# Stereoselectivity of Galactosylation Reactions *In Vacuo*

**DOI:** 10.1021/jacs.6c09131

**Published:** 2026-08-05

**Authors:** Chun-Wei Chang, Andrei Zviagin, Dana Wehner, Niklas Geue, Jacob S. Jordan, Ana Zidar, Ariane R. Wieseke, Kim Greis, Gaël M. Vos, Michael Götze, Oleg Boyarkin, Kevin Pagel

**Affiliations:** † Freie Universität Berlin, Institute of Chemistry and Biochemistry, Altensteinstraße 23A, Berlin, Germany 14195; ‡ Fritz Haber Institute of the Max Planck Society, Department of Molecular Physics, Faradayweg 4-6, Berlin, Germany 14195; § SCI-SB-RB, ISIC, École Polytechnique Fédérale de Lausanne, Station 6, CH-1015 Lausanne, Switzerland

## Abstract

Controlling the stereoselective outcome of glycosidic bond formation remains a major challenge in chemical synthesis. This process typically proceeds along a complex S_N_1-S_N_2 mechanistic continuum. In solution, the key contributions to these mechanisms are difficult to determine due to influence by structural and environmental variables, such as solvents, counterions or temperature. To isolate the intrinsic factors driving steroselectivity in glycosylations, we investigate the reactions for the monosaccharide galactose in the gas phase. We perform ion–molecule galactosylation reactions in complete isolation inside an ion trap of a mass spectrometer and directly probe the stereochemistry of the formed products using mass-resolved ultraviolet cold ion spectroscopy. The results show that α-selectivity is significantly weaker in the gas phase than in solution, and that the product stereochemistry might be influenced by the nucleophile’s electron density rather than its overall nucleophilic strength.

## Graphical abstract

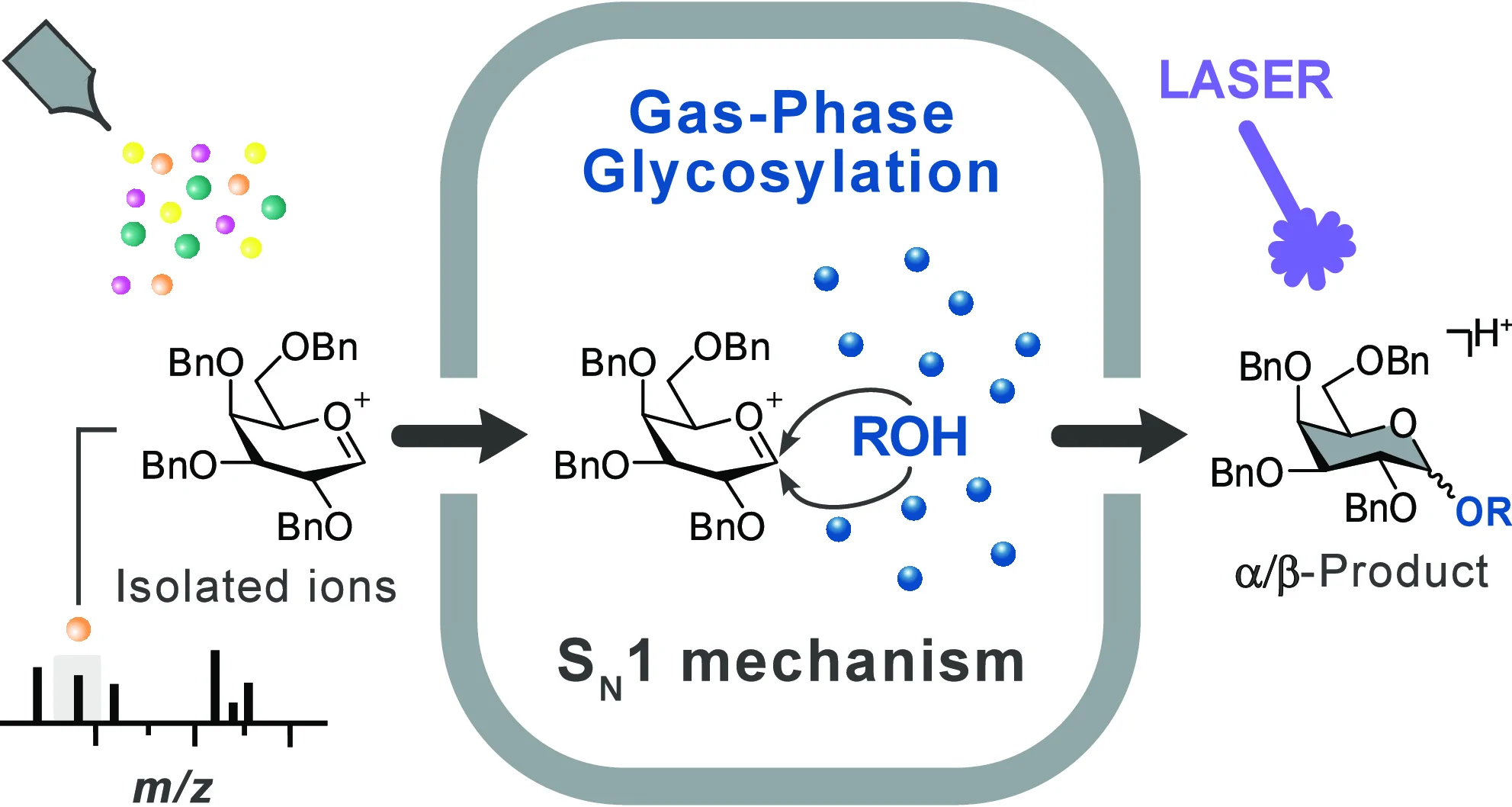

Glycosylation is a fundamental reaction for building complex oligosaccharides in nature.^1,2^ It links glycosylating agents and nucleophiles to form new glycosidic bonds. The reaction forms a new chiral center at the anomeric position. This in turn, generates two possible stereoisomeric products: α- and β-glycosides.^3^ Traditionally, the reaction does not proceed via a single, discrete mechanism. Instead, the mechanism lies along a continuum between two pathways: the unimolecular S_N_1 and bimolecular S_N_2 pathways (Figure 1a).^4,5^ While kinetic isotope effect experiments are often employed to probe these pathways, the boundary between these two extremes remains elusive.^6–8^ This uncertainty arises because changes in temperature, solvent, or the structure of the building blocks easily shift the reaction pathway.^7,9,10^ Consequently, quantifying the individual contributions of S_N_1 and S_N_2 throughout the reaction remains a significant analytical hurdle.

**Figure 1:**
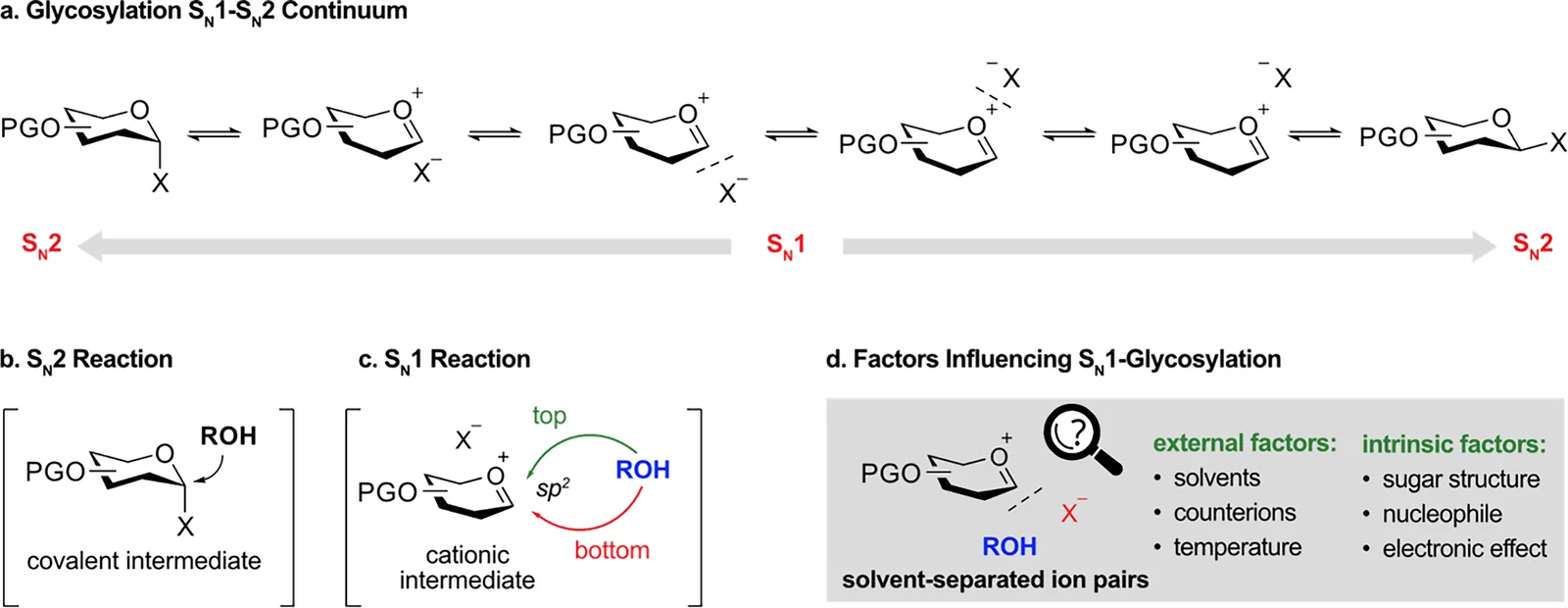
Challenges in the stereoselective installation of glycosidic bonds. (a) The glycosylation mechanism exists on an S_N_1-S_N_2 continuum. The reaction often results in a mixture of α- and β-anomers. (b) The S_N_2 pathway proceeds with stereoinversion at the anomeric center. It allows for predictable product formation. (c) In the S_N_1 pathway, a cationic intermediate is formed. Nucleophiles can attack the intermediate from either the top or bottom, creating α- or β-glycosides as respective products. (d) Various factors influence the stereochemical outcome of S_N_1-type glycosylations, ranging from external environmental conditions (solvent, temperature) to the intrinsic properties of the building blocks.

S_N_2 glycosylation involves covalent glycosyl intermediates and leads to stereoinverted products (Figure 1b). Nuclear magnetic resonance (NMR) spectroscopy has provided detailed characterization of this process.^11–13^ Specifically, exchange NMR has enabled the observation of dynamic exchange between glycosyl triflate and the counterions in solution.^14,15^ Under this framework, the final stereochemical outcome was found to be governed by the Curtin-Hammett kinetic principle,^12,15,16^ with the product ratio depending on the relative energy barriers of the competing transition states.

In contrast, the understanding of the S_N_1 mechanism remains limited. The reaction is governed by highly reactive species, known as glycosyl cations (Figure 1c).^17^ The supposed oxocarbenium ions feature an *sp* ^2^-hybridized anomeric carbon.^13,18^ Their planar geometry allows the nucleophile to attack from either the top (β) or bottom (α) face, ultimately dictating the product stereoselectivity. To understand the origin of stereoselectivity, it is essential to characterize the three-dimensional structure of glycosyl cations. However, these cations are highly transient with extremely short lifetimes, posing a significant analytical challenge.^13,18,19^ Various spectroscopic techniques have been developed to address this.^13,18–20^ For instance, NMR-based methods analyze these cations by stabilizing them with superacids.^13,21^ Alternatively, gas-phase infrared (IR) spectroscopy has emerged as a powerful tool for the direct structural analysis of glycosyl cations.^22–25^ It provides unique vibrational fingerprints that allow the assignment of ion structures, when compared with theoretical spectra simulated from density functional theory (DFT) calculations.

Despite effective methods for characterizing intermediates, the coupling step in glycosylation reactions remains elusive. Its complexity stems from the fact that it involves a bimolecular reaction with a nucleophile. Besides its role as a reactant, the nucleophile alters the sugar conformation to minimize steric hindrance, thereby ensuring an approach along the lowest energy pathway.^26–28^ In solution, this process is further complicated by external factors (Figure 1d).^29,30^ The solvent, for example, orients the cationic intermediate relative to the nucleophile^31^ and dictates the reactivity of solvent-separated ion pairs (SSIPs).^32,33^ Ultimately, the dynamic equilibria between these intermediates and the solvent govern the stereoselectivity of the glycosylation. Another key parameter for tuning sugar reactivity and controlling donor activation is temperature, which dictates both the overall yield and the stereoselective outcome of the glycosylation.^34^

Because these intrinsic and external factors are strongly interconnected in solution, it remains a challenge to determine the true driver of stereoselectivity. It is often unclear whether the outcome depends primarily on the sugar building blocks, the nucleophile, or external solvent effects. This is particularly relevant for galactose, whose building blocks are often highly reactive and exceptionally sensitive to their environment. Even small changes in temperature or solvent can completely alter the stereochemical outcome.^29^ Therefore, without observing the glycosyl cations and nucleophiles together in a controlled environment, these individual contributions cannot be easily distinguished. This lack of clarity represents a major hurdle in predicting the formation of challenging linkages, such as 1,2-*cis*-glycosidic bonds.

To obtain this controlled environment for S_N_1-glycosylations, we performed galactosylation reactions in complete isolation inside a mass spectrometer (MS). This allows us to study the reactions free from the complicating factors in solution. Technically, this is achieved by trapping the galactosyl cations in an ion trap, where they are brought into reaction with volatile nucleophiles to form a new glycosidic bond. The resulting products are monitored *in situ* using gas-phase cold ion UV fragmentation spectroscopy (Figure S1). The results reveal that the inherent chemical properties of the reactants have a considerably smaller impact on the S_N_1-glycosylation than was previously expected from solution-phase experiments.

To conduct galactosylation reactions in the gas phase (Figure 2a), a benzyl-protected thiogalactoside building block was chosen as precursor. Thiols are commonly used as leaving groups in carbohydrate synthesis and the benzyl-protecting groups prevent unwanted side reactions. Thiogalactoside precursor ions were generated from an organic solution using nanoelectrospray ionization (n-ESI). On the way into MS, the ions undergo controlled *in-source* dissociation via collisions with ambient air molecules to produce galactosyl cations. Finally, the ions are transferred into MS and stored in a dedicated reaction ion trap at room temperature and a residual gas pressure of 10^–4^ mbar. While these intermediates are highly reactive in solution, they remain stable in the gas phase under this low pressure due to the inherent lack of nucleophiles *in vacuo*. Bleeding low-pressure vapor of a nucleophile into the trap initiates a gas-phase galactosylation reaction (Figure 2b and Figure S2).

**Figure 2:**
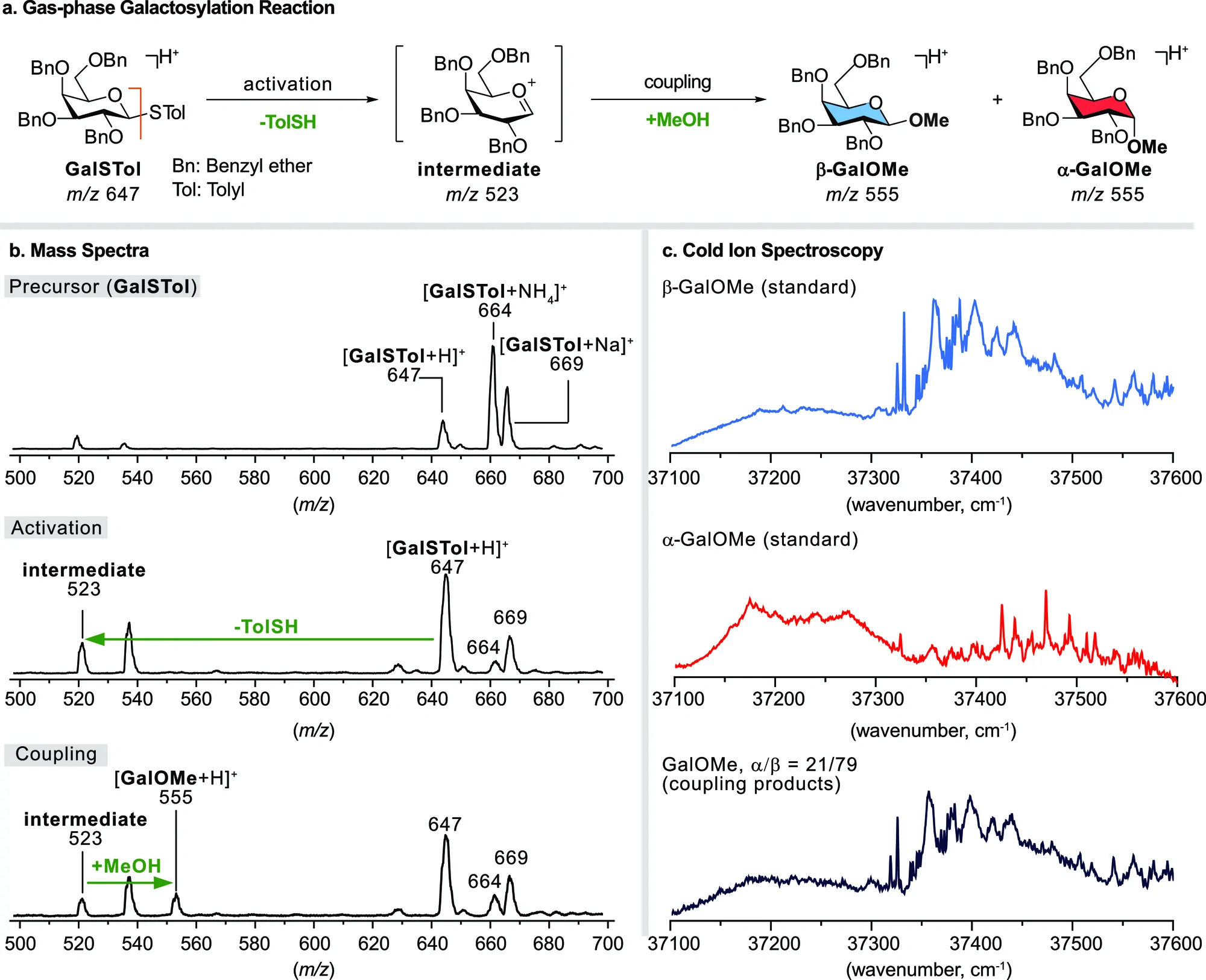
Galactosylation reaction in the gas phase. (a) A galactosyl cation is generated through *in-source* collision induced dissociation. Methanol vapor reacts with the galactosyl cation producing an α/β mixture of the protonated GalOMe via an S_N_1 mechanism. (b) Mass spectrometry is used to monitor the gas-phase galactosylation reaction. Electrospray ionization of the thiogalactoside precursor yields protonated, ammonium and sodium adducts. In-source fragmentation of the protonated precursor at *m*/*z* 647 yields galactosyl cations at *m*/*z* 523. The gas-phase reaction with methanol vapor results in GalOMe product ions at *m*/*z* 555. (c) Reference UV fragmentation spectra of the anomerically pure standards of β-GalOMe (blue), α-GalOMe (red), and of the gas-phase galactosylation product (black). The α/β ratio of the *in situ* product is derived from the best fit of the black trace by a linear combination of the blue and red ones. This analysis relies on distinct spectral fingerprints. A broad feature between 37100 cm^–1^ and 37300 cm^–1^ characterizes the α-anomer, whereas distinct bands from 37300 cm^–1^ and 37600 cm^–1^ are related to the β-anomer.

A galactosylation reaction yields either α- or β-galactoside anomers. The α/β anomeric ratio of the products formed in solution was determined using NMR and liquid chromatography. To determine the anomeric ratio for the products of the reaction in the gas phase, we employed UV photofragmentation cold ion spectroscopy, which is a well-established method for the analysis of isomeric biomolecules.^35–38^ It involves measuring UV spectra of the anomerically mixed products and of two anomerically pure α/β-galactoside standards. The mixed spectrum is then numerically decomposed into a linear combination of the two standard reference spectra (Supporting Information, section 3), yielding relative concentrations of the individual anomers in the product mixture. To measure a UV spectrum of the product ions, they are *m*/*z*-isolated and transferred from the reaction ion trap into a cryogenic ion trap, where they are collisionally cooled to ∼ 10 K. Next, the ions undergo photofragmentation by a UV laser pulse. Spectra are recorded by monitoring the abundance of fragment ions as a function of laser wavelength. UV spectra of the anomerically pure synthetic α/β-galactosides were recorded similarly, except that the ESI-generated ions were not subjected to *in-source* fragmentation and no nucleophile vapor was bled into the reaction ion trap.

Isolation of the reactants in the gas phase at low background pressure essentially removes all external interferences present in solution. This allows a precise examination of the stereoselective contribution of the reactants in S_N_1-glycosylation reactions (Figure 2c). Spectral differences arise because the orientation of the anomeric *O*-methyl substituent alters the inductive polarization of the sugar, which in turn shifts the electronic and vibrational energy levels in the UV spectra.^39^

The obtained spectral difference reveals a relative concentration of α-GalOMe produced in the gas-phase of 21 ± 6% (Figures S3 and S4). This is nearly half of the 42% obtained in solution, as determined by NMR (section 8 in Supporting Information). The product ratio reflects the stereoselectivity that arises in a pure S_N_1-type glycosylation reaction. To assess the stability of the glycosyl products, NMR spectroscopy (in solution) and ion mobility mass spectrometry (in the gas phase) were employed (Figures S5 and S6). These data and literature precedents indicate that the anomeric center is locked, which suppresses low-energy mutarotation pathways.^40–43^ This suggests that both solution- and gas-phase ratios are meaningful and can be analyzed quantitatively.

To systematically assess the effect of different steric and electronic factors in S_N_1-glycosylation, we investigated gas-phase galactosylations with different nucleophiles under otherwise identical conditions (Figures S7–S10). The anomeric composition of the gas-phase products at 298 K was compared to that of the solution reactions (Figure 3). In solution, the nonparticipating solvent dichloromethane was used to generate conditions that facilitate the S_N_1 mechanism. Consistently with previous reports,^44,45^ the solution galactosylation reactions show an inverse relationship between α-selectivity and nucleophile strength (gray bars), such that a higher abundance of the α-product is observed for the weaker nucleophiles (Figure S11).

**Figure 3:**
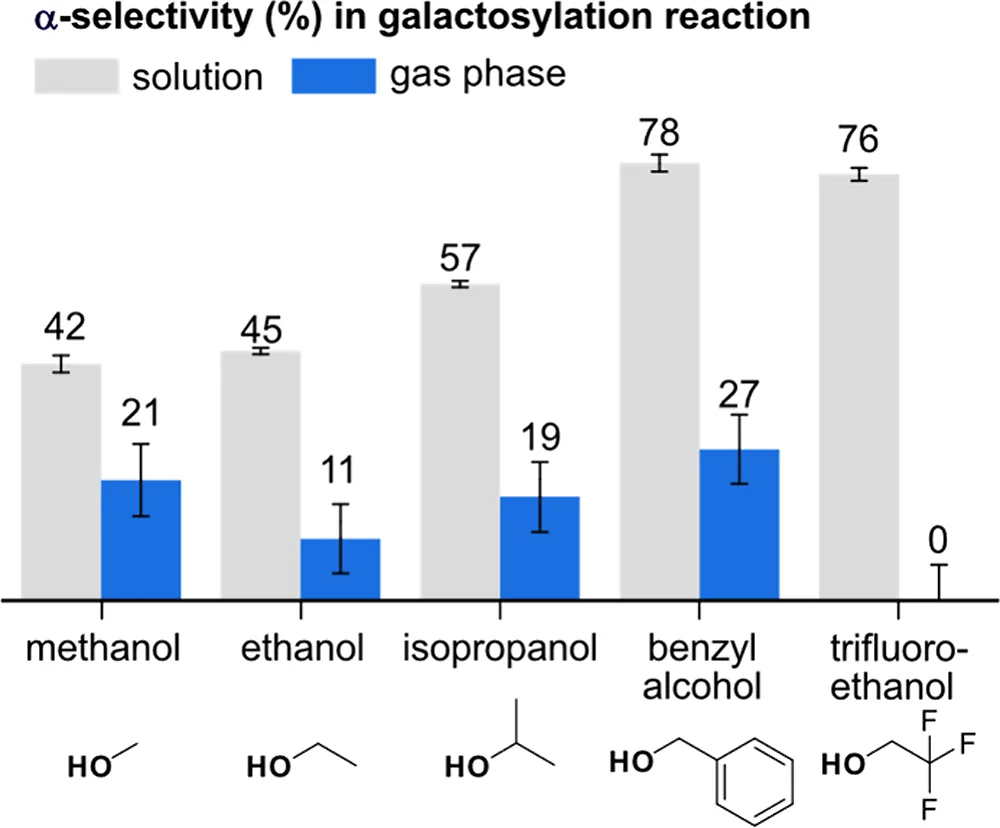
Stereoselectivity in solution and gas-phase galactosylation reactions at 25 °C. In the solution phase, α-product formation correlates with the strength of the nucleophile. In contrast, the α-content is significantly reduced in the gas phase and appears to be largely independent of nucleophilic strength.

This general trend stands in a stark contrast to what is observed when the influence of the solution is removed. First, for all the studied nucleophiles, α-selectivity is significantly reduced in gas-phase galactosylation reactions, with a minimum reduction of 1.9 times measured for the methanol substituent. The reaction with the weakest tested nucleophile, trifluoroethanol, exhibits a particularly striking difference between the abundance of the α-product in solution (76%) and in the gas phase (0%). Interestingly, the relative selectivity in the gas phase might be influenced by electronic effects. Electron-rich nucleophiles, such as benzyl alcohol, show a slightly higher α-content than electron-poor nucleophiles such as trifluoroethanol. This data aligns with earlier theoretical calculations suggesting that α-galactoside formation is predominantly driven by electrostatic interactions *in vacuo*.^46,47^ Analogous *in vacuo* glycosylation reactions were also attempted with glucose and mannose (Figures S12–S17); however, these yielded inconclusive results. Future studies will investigate how general the observed effects are for other monosaccharides.

Another fundamental question is how the structure of the intermediate correlates with the observed herein stereoselectivities, and how these correlations differ between solution and gas phase. Previously, we characterized the structure of the perbenzylated galactosyl cation using cryogenic gas-phase IR spectroscopy, suggesting interactions of the C6-OBn group with the positively charged anomeric center (C1).^48^ Here, we extend this study with a detailed conformational analysis of the galactosyl cation (Figure S20). The computational results show that, besides previously reported conformations that facilitate C6–C1 interactions and hence α-selectivity,^48^ other structures occur at marginally higher energies that would prefereably lead to β-products.^49,50^ This might rationalize our experimentally observed gas-phase β-selectivity, however this warrants further investigations.

In conclusion, we present herein the first direct evaluation of galactosylation reactions by removing interference from external factors, such as solvent and counterions. This is achieved by performing ion–molecule reactions of glycosyl cations in the gas phase and subsequent *in situ* sensing of the formed products with mass-selective UV cold ion spectroscopy. Compared to galactosylation reactions in solution, the intrinsic gas-phase α-selectivity is significantly reduced and appears to be independent of the nucleophilic strength. Instead, α-glycosylation appears to be enhanced for electron-donating nucleophiles. These results illustrate how glycosylation reactions are heavily influenced by the surrounding environment. Ultimately, isolating these intrinsic reactivity factors will provide datasets that can be used to train machine learning models in the future to predict solution-phase stereoselectivities. Combining *in vacuo* insights with solution-phase data and predictive AI could enable the rational design of glycosylation strategies.

## Supplementary Material





## Notes

The data reported in this paper are available in the main text or the Supporting Information. The Supporting Information is available free of charge at https://pubs.acs.org/doi/10.1021/jacs.6c09131.

Deconvolution (ZIP)
Additional information as detailed in the text (PDF)

## References

[1] Yang Y., Zhang X., Yu B. (2015). *O*-Glycosylation
Methods in the Total Synthesis of Complex Natural Glycosides. Nat. Prod. Rep..

[2] Yang Y., Yu B. (2017). Recent Advances in the Chemical Synthesis of C-Glycosides. Chem. Rev..

[3] Leng W.-L., Yao H., He J.-X., Liu X.-W. (2018). Venturing
beyond donor-controlled
glycosylation: new perspectives toward anomeric selectivity. Acc. Chem. Res..

[4] Adero P. O., Amarasekara H., Wen P., Bohé L., Crich D. (2018). The Experimental Evidence in Support of Glycosylation Mechanisms
at the S_N_1–S_N_2 interface. Chem. Rev..

[5] Crich D. (2010). Mechanism
of a Chemical Glycosylation Reaction. Acc. Chem.
Res..

[6] Zhuo M. H., Wilbur D. J., Kwan E. E., Bennett C. S. (2019). Matching Glycosyl
Donor Reactivity to Sulfonate Leaving Group Ability Permits S_N_2 Glycosylations. J. Am. Chem. Soc..

[7] Huang M., Garrett G. E., Birlirakis N., Bohé L., Pratt D. A., Crich D. (2012). Dissecting the Mechanisms
of a Class
of Chemical Glycosylation using Primary ^13^C Kinetic Isotope
Effects. Nat. Chem..

[8] Yu F., Li J., DeMent P. M., Tu Y.-J., Schlegel H. B., Nguyen H. M. (2019). Phenanthroline-catalyzed
Stereoretentive Glycosylations. Angew. Chem.,
Int. Ed..

[9] van
Hengst J. M. A., Hellemons R. J. C., Remmerswaal W. A., van de Vrande K. N. A., Hansen T., van der Vorm S., Overkleeft H. S., van der Marel G. A., Codée J. D. C. (2023). Mapping
the Effect of Configuration and Protecting Group Pattern on Glycosyl
Acceptor Reactivity. Chem. Sci..

[10] Chang C.-W., Lin M.-H., Chan C.-K., Su K.-Y., Wu C.-H., Lo W.-C., Lam S., Cheng Y.-T., Liao P.-H., Wong C.-H., Wang C.-C. (2021). Automated
Quantification of Hydroxyl
Reactivities: Prediction of Glycosylation Reactions. Angew. Chem., Int. Ed..

[11] Chang C.-W., Lin M.-H., Chiang T.-Y., Wu C.-H., Lin T.-C., Wang C.-C. (2023). Unraveling the Promoter Effect and the Roles of Counterion
Exchange in Glycosylation Reaction. Sci. Adv..

[12] Santana A. G., Montalvillo-Jimenez L., Diaz-Casado L., Corzana F., Merino P., Canada F. J., Jimenez-Oses G., Jimenez-Barbero J., Gomez A. M., Asensio J. L. (2020). Dissecting
the Essential Role of
Anomeric b-Triflates in Glycosylation Reactions. J. Am. Chem. Soc..

[13] Franconetti A., Ardá A., Asensio J. L., Blériot Y., Thibaudeau S., Jiménez-Barbero J. (2021). Glycosyl Oxocarbenium
Ions: Structure, Conformation, Reactivity, and Interactions. Acc. Chem. Res..

[14] de
Kleijne F. F. J., Moons P. H., Ter Braak F., Almizori H. R., Jakobs L. J. H., Houthuijs K. J., Berden G., Martens J., Oomens J., Rutjes F., White P. B., Boltje T. J. (2025). Mechanism of C-3 Acyl Neighboring
Group Participation in Mannuronic Acid Glycosyl Donors. J. Am. Chem. Soc..

[15] Moons P. H., de Kleijne F. F. J., van Wieringen T., ter Braak F., Almizori H. R., Jakobs L. J. H., de Kleijne W. P. M., Rutjes F. P. J. T., Martens J., Oomens J., Korevaar P. A., White P. B., Boltje T. J. (2025). Elucidating the
Curtin–Hammett Principle in Glycosylation Reactions: The Decisive
Role of Equatorial Glycosyl Triflates. J. Am.
Chem. Soc..

[16] Remmerswaal W. A., Elferink H., Houthuijs K. J., Hansen T., ter Braak F., Berden G., van der Vorm S., Martens J., Oomens J., van der Marel G. A., Boltje T. J., Codée J. D. C. (2024). Anomeric
Triflates versus Dioxanium Ions: Different Product-Forming Intermediates
from 3-Acyl Benzylidene Mannosyl and Glucosyl Donors. J. Org. Chem..

[17] Bohé L., Crich D. (2015). A Propos of Glycosyl Cations and the Mechanism of Chemical Glycosylation;
The Current State of the Art. Carbohydr. Res..

[18] Braak F. t., Elferink H., Houthuijs K. J., Oomens J., Martens J., Boltje T. J. (2022). Characterization
of Elusive Reaction Intermediates
Using Infrared Ion Spectroscopy: Application to the Experimental Characterization
of Glycosyl Cations. Acc. Chem. Res..

[19] Chang C. W., Wehner D., Prabhu G. R. D., Moon E., Safferthal M., Bechtella L., Osterlund N., Vos G. M., Pagel K. (2025). Elucidating
reactive sugar-intermediates by mass spectrometry. Commun. Chem..

[20] Geue N., Safferthal M., Pagel K. (2025). Collision-Induced Fragmentation of
Oligosaccharides: Mechanistic Insights for Mass Spectrometry-Based
Glycomics. Angew. Chem., Int. Ed..

[21] Martin A., Arda A., Désiré J., Martin-Mingot A., Probst N., Sinaÿ P., Jiménez-Barbero J., Thibaudeau S., Blériot Y. (2016). Catching elusive glycosyl cations
in a condensed phase with HF/SbF_5_ superacid. Nat. Chem..

[22] Mucha E., Marianski M., Xu F. F., Thomas D. A., Meijer G., von Helden G., Seeberger P. H., Pagel K. (2018). Unravelling the Structure
of Glycosyl Cations via Cold-ion Infrared Spectroscopy. Nat. Commun..

[23] Chang C.-W., Greis K., Prabhu G. R. D., Wehner D., Kirschbaum C., Ober K., Torres-Boy A. Y., Leichnitz S., Meijer G., von Helden G., Seeberger P. H., Pagel K. (2024). Mechanistic insight into benzylidene-directed
glycosylation reactions
using cryogenic infrared spectroscopy. Nat.
Synth..

[24] Geue N., Greis K., Omoregbee-Leichnitz S., Kirschbaum C., Chang C. W., Meijer G., von Helden G., Seeberger P. H., Pagel K. (2025). Influence of Levulinoyl Protecting
Groups on Glycosylation Stereoselectivity and Glycosyl Cation Structure. Eur. J. Org. Chem..

[25] Geue N., Greis K., Omoregbee-Leichnitz S., Kirschbaum C., Meijer G., von Helden G., Seeberger P. H., Marianski M., Pagel K. (2026). Evaluating Participation Modes in
Peracetylated Glycosyl Cations. Org. Lett..

[26] Lucero C. G., Woerpel K. A. (2006). Stereoselective
C-Glycosylation Reactions of Pyranoses:The
Conformational Preference and Reactions of the Mannosyl Cation. J. Org. Chem..

[27] Hansen T., Lebedel L., Remmerswaal W. A., van der Vorm S., Wander D. P. A., Somers M., Overkleeft H. S., Filippov D. V., Desire J., Mingot A., Bleriot Y., van der Marel G. A., Thibaudeau S., Codée J. D. C. (2019). Defining
the S_N_1 Side of Glycosylation Reactions: Stereoselectivity
of Glycopyranosyl Cations. ACS Cent. Sci..

[28] Remmerswaal W. A., Hoogers D., Schoenmakers J., Bickelhaupt F. M., Hansen T., Codee J. D. (2026). Beyond the Two-Conformer
Model: The
Role of Boat Conformers in the Stereoselectivity of SN1-Type Glycosylations. Chem. Sci..

[29] Chatterjee S., Moon S., Hentschel F., Gilmore K., Seeberger P. H. (2018). An Empirical
Understanding of the Glycosylation Reaction. J. Am. Chem. Soc..

[30] Andreana P. R., Crich D. (2021). Guidelines for *O*-Glycoside Formation from First
Principles. ACS Cent. Sci..

[31] Dorst K. M., Engström O., Angles d’Ortoli T., Mobarak H., Ebrahemi A., Fagerberg U., Whitfield D. M., Widmalm G. (2024). On the influence of
solvent on the stereoselectivity
of glycosylation reactions. Carbohydr. Res..

[32] Crich D. (2021). En Route to
the Transformation of Glycoscience: A Chemist’s Perspective
on Internal and External Crossroads in Glycochemistry. J. Am. Chem. Soc..

[33] Hettikankanamalage A. A., Lassfolk R., Ekholm F. S., Leino R., Crich D. (2020). Mechanisms
of Stereodirecting Participation and Ester Migration from Near and
Far in Glycosylation and Related Reactions. Chem. Rev..

[34] Lin M. H., Kuo Y. T., Danglad-Flores J., Sletten E. T., Seeberger P. H. (2024). Parametric
Analysis of Donor Activation for Glycosylation Reactions. Chem. Eur. J..

[35] Kopysov V., Makarov A., Boyarkin O. V. (2015). Colors for molecular masses: fusion
of spectroscopy and mass spectrometry for identification of biomolecules. Anal. Chem..

[36] Kopysov V., Makarov A., Boyarkin O. V. (2017). Identification of
Isomeric Ephedrines
by Cold Ion UV Spectroscopy: Toward Practical Implementation. Anal. Chem..

[37] Saparbaev E., Kopysov V., Aladinskaia V., Ferrieres V., Legentil L., Boyarkin O. V. (2020). Identification and
Quantification
of Any Isoforms of Carbohydrates by 2D UV-MS Fingerprinting of Cold
Ions. Anal. Chem..

[38] Saparbaev E., Yamaletdinov R., Boyarkin O. V. (2021). Identification of Isomeric Lipids
by UV Spectroscopy of Noncovalent Complexes with Aromatic Molecules. Anal. Chem..

[39] Saparbaev E., Aladinskaia V., Yamaletdinov R., Pereverzev A. Y., Boyarkin O. V. (2020). Revealing Single-Bond Anomeric Selectivity
in Carbohydrate–Protein
Interactions. J. Phys. Chem. Lett..

[40] Habibi S. C., Nagy G. (2023). General Method to Obtain
Collision Cross-Section Values in Multipass
High-Resolution Cyclic Ion Mobility Separations. Anal. Chem..

[41] Cui H., Zheng D., Chu H., Li Y., Li G. (2026). Exploring
the Mutarotation Mechanism of Glucose in Solution Using Deep Learning
Potential. J. Phys. Chem. B.

[42] Pellegrinelli R. P., Yue L., Carrascosa E., Warnke S., Ben Faleh A., Rizzo T. R. (2020). How General Is Anomeric
Retention during Collision-Induced
Dissociation of Glycans?. J. Am. Chem. Soc..

[43] Greis K., Mucha E., Lettow M., Thomas D. A., Kirschbaum C., Moon S., Pardo-Vargas A., von Helden G., Meijer G., Gilmore K., Seeberger P. H., Pagel K. (2020). The Impact of Leaving Group Anomericity on the Structure of Glycosyl
Cations of Protected Galactosides. ChemPhysChem.

[44] van
der Vorm S., Hansen T., Overkleeft H. S., van der Marel G. A., Codée J. D. C. (2017). The Influence of Acceptor Nucleophilicity
on the Glycosylation Reaction Mechanism. Chem.
Sci..

[45] van
der Vorm S., Hansen T., van Hengst J. M. A., Overkleeft H. S., van der Marel G. A., Codée J. D. C. (2019). Acceptor Reactivity in Glycosylation Reactions. Chem. Soc. Rev..

[46] Mo Y. (2010). Computational
Evidence That Hyperconjugative Interactions Are Not Responsible For
The Anomeric Effect. Nat. Chem..

[47] Alabugin I. V., Kuhn L., Krivoshchapov N. V., Mehaffy P., Medvedev M. G. (2021). Anomeric
Effect, Hyperconjugation and Electrostatics: Lessons From Complexity
in a Classic Stereoelectronic Phenomenon. Chem.
Soc. Rev..

[48] Marianski M., Mucha E., Greis K., Moon S., Pardo A., Kirschbaum C., Thomas D. A., Meijer G., von Helden G., Gilmore K., Seeberger P. H., Pagel K. (2020). Remote Participation
during Glycosylation Reactions of Galactose Building Blocks: Direct
Evidence from Cryogenic Vibrational Spectroscopy. Angew. Chem., Int. Ed..

[49] Siyabalapitiya
Arachchige S., Crich D. (2022). Side Chain Conformation and Its Influence
on Glycosylation Selectivity in Hexo- and Higher Carbon Furanosides. J. Org. Chem..

[50] Dharuman S., Amarasekara H., Crich D. (2018). Interplay of Protecting Groups and
Side Chain Conformation in Glycopyranosides. Modulation of the Influence
of Remote Substituents on Glycosylation?. J.
Org. Chem..

