# Supplementary material for: Stereoselectivity of Galactosylation Reactions In Vacuo
Source: J Am Chem Soc. 2026 Aug 5;148(32):34045–50. doi: 10.1021/jacs.6c09131 (PMC13495751; doi:10.1021/jacs.6c09131)
Supplement: Supplementary file 1 [file ja6c09131_si_002.pdf]

Supplementary Materials for

Stereoselectivity of Galactosylation Reactions in *vacuo*

Chun-Wei Chang[1,2,†], Andrei Zviagin[3,†], Dana Wehner[1,2], Niklas Geue[1,2], Jacob S. Jordan[1,2], Ana Zidar[1,2], Ariane R. Wieseke[1,2], Kim Greis[1,2], Gaël M. Vos[1,2], Michael Götze[1,2], Oleg Boyarkin[3,*] and Kevin Pagel[1,2,*]

[†] These authors equally contributed to this work.

[1] Freie Universität Berlin, Institute of Chemistry and Biochemistry, Altensteinstraße 23A, Berlin, Germany 14195

[2] Fritz Haber Institute of the Max Planck Society, Department of Molecular Physics, Faradayweg 4-6, Berlin, Germany 14195

[3]SCI-SB-RB, ISIC, École Polytechnique Fédérale de Lausanne, Station 6, CH-1015 Lausanne, Switzerland

***Corresponding Authors**

Prof. Dr. Kevin Pagel

E-Mail: kevin.pagel@fu-berlin.de

Prof. Dr. Oleg Boyarkin

E-Mail: oleg.boiarkin@epfl.ch

1 General Information 3
2 Materials and conditions..........4
2.1 General Procedure for Glycosylation Reaction in the Gas Phase and Solutions..........4
2.2 Procedure for Gas-phase Glycosylation Reaction and Cold Ion Spectroscopy..........6
2.3 Characterization of Gas-phase Glycosyl Product using Cold Ion Spectroscopy on Methyl Galactoside..........9
3. Error Estimation For Stereoselectivity in Gas-phase Glycosylation Reactions 11
4. Stability Test of Glycosyl Product..........13
5. Characterization of Gas-phase Glycosyl Product using Cold Ion Spectroscopy and NMR on Galactoside with Different Nucleophiles..........15
6. Studying the Gas-phase Glycosylations on Mannose and Glucoside..........20
7. Structure Analysis of Oxocarbenium Ion 25
8. General Procedure for Chemical Glycosylation Reaction..........27
8.1 Characterization of Glycosyl Product in Solution Synthesis..........29
Ethyl 2,3,4,6-Tetra-O-benzyl-D-galactopyranoside 36
iso-Propyl 2,3,4,6-Tetra-O-benzyl-D-galactopyranoside 40
Trifluoroethyl 2,3,4,6-Tetra-O-benzyl-D-galactopyranoside 48
1,2,3,4,6-Penta-O-benzyl-D-galactopyranoside 54
Ethyl 2,3,4,6-Tetra-O-benzyl-D-glucopyranoside 60
iso-Propyl 2,3,4,6-Tetra-O-benzyl-D-glucopyranoside 66
Ethyl 2,3,4,6-Tetra-O-benzyl-D-mannopyranoside..........72
iso-Propyl 2,3,4,6-Tetra-O-benzyl-D-mannopyranoside..........75
9. Reference 78

1 General Information

All **chemicals** were reagent grade and used as supplied unless otherwise noted. All **solvents** for chemical reactions were commercially purchased in p.a. quality. For liquid chromatography, solvents with corresponding quality were used.

Reaction completion, identity, and purity of all compounds were determined by thin-layer chromatography (**TLC**) and/or nuclear magnetic resonance (**NMR**). TLC was performed on ALUGRAM Xtra SIL G/UV_{245} pre-coated TLC sheets (0.20 mm) from Macherey-Nagel GmbH. Compounds were visualized by UV irradiation (254 nm) and stained by spraying with a solution of $Ce(SO_4)_2$, $(NH_4)_6Mo_7O_2$, and H_2SO_4 in water and subsequently heating on a hot plate. Compounds were purified using CombiFlash NextGen 300+ flash chromatography system from Teledyne Labs and RediSep Gold High Performance 24g HP Silica columns. ^1H, ^{13}C, COSY and HSQC **NMR** spectra were recorded in parts per million (δ) relative to the resonance of the solvent on a Spektrometer ECP500 (500 MHz) or Spektrometer ECZ600 S (600 MHz) spectrometer. Assignments were supported by COSY and HSQC experiments. Electrospray ionization mass spectra (**ESI-MS**) were obtained using UPLC-Synapt G2-S HDMS mass spectrometer (Waters) and Ionspec QFT-7 (Agilent/Varian).

2 Materials and conditions

2.1 General Procedure for Glycosylation Reaction in the Gas Phase and Solutions

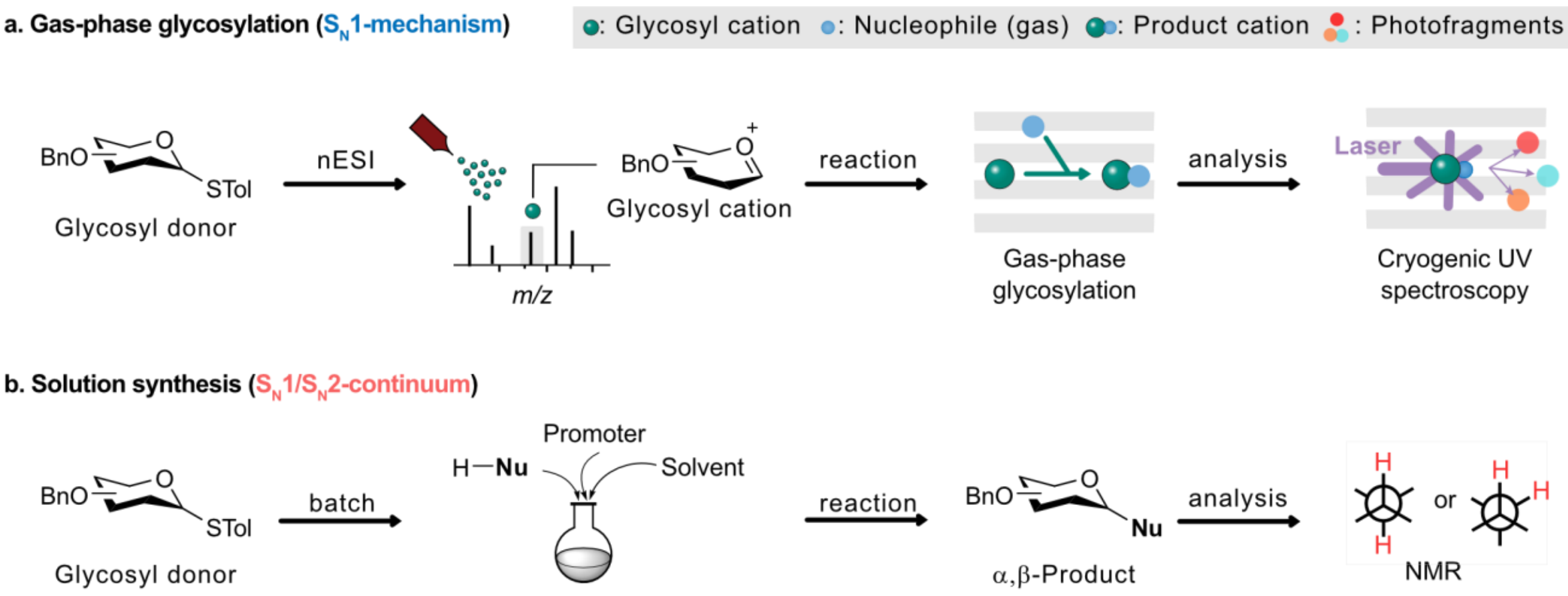

Figure S1. Dual approach investigation of the intrinsic stereoselective glycosylation in S_N1 mechanism. a) Gas-phase synthesis. Glycosyl cation is formed through in-source fragmentation, followed by gas-phase glycosylation in the ion trap filled with nucleophile vapor. The formation of glycosyl product and α/β selectivity is measured by cryogenic UV spectroscopy. b) Solution Synthesis: Chemical glycosylation reactions are performed in batch reactions, where the mechanism spans the continuum between S_N1 and S_N2.

To isolate the S_N1 component of the glycosylation reaction we first generate glycosyl cations during infusion in the apparatus through collision-induced dissociation (CID) in the source region using a nano-electrospray ionization (n-ESI) source. In positive ion mode, CID on activated glycosyl donors results in the efficient removal of the leaving group, generating the glycosyl cations of interest. The activated glycosides are transferred to a temperature-controlled octupole ion trap (pre-trap) filled with the vapor of a nucleophilic acceptor supplied through a leak valve. The generated ions are contained and allowed to react in the pre-trap for 50 ms and subsequently guided through a quadrupole mass filter to select for ions corresponding with the *m/z* of the glycosylation product. In this manner, S_N2-type reactions cannot occur and we observed pure S_N1-type glycosylation reactions in the gas phase. Ions of interest are transmitted to a cryogenic octupole ion trap (CIT) kept at 6 K, cooled by pulsed helium. The thermalized glycosylation products are then probed in the CIT with a UV laser pulse with tunable photon energy. Fragmentation induced by photon absorption events is monitored to produce high-resolution

cryogenic UV photodissociation spectra (UVPD). A laser-free cycle measures the signal baseline of the product ions in the cold trap, which is used to normalize the fragment signal. Each data point represents an average of 10 measurements and the complete spectrum is recorded at least three times. The resulting parent and fragment ions are released and detected by a quadrupole mass filter equipped with a channeltron detector.

Compared to the gas-phase experiments, a generally higher α-content is observed in solution. This difference in anomeric preference results from a continuum of S_N1 and S_N2 reactions mediated by external factors such as solvent,[1-3] activators,[4,5] counter ions,[6-8] and covalent species, which are not present in the gas phase.[4,9-11]

The choice of UV spectroscopy over IR was primarily dictated by experimental and instrumental constraints. In our setup, the relatively low intensity of the available IR OPO systems necessitates messenger-tagging spectroscopy to obtain high-quality vibrational spectra. Typically, this tagging is performed within a cryogenic ion pre-trap to ensure background-free IR data.

In the present study, however, this pre-trap is dedicated to the gas-phase glycosylation reaction, which must be conducted at room temperature. Because the pre-trap cannot simultaneously maintain room temperature for the reaction and cryogenic temperatures for messenger tagging, IR spectroscopy was not viable for this specific experimental design. In contrast, UV photofragmentation spectroscopy provided a robust and reliable alternative, allowing for the characterization of reaction products directly under these instrumental conditions.

2.2 Procedure for Gas-phase Glycosylation Reaction and Cold Ion Spectroscopy

Glycosyl donor and synthetic standards were dissolved in the acetonitrile/water (9/1) mixture with 0.2 vol% of acetic acid to the final concentrations of 100 µM and 50 µM, respectively. All solvents were of HPLC grade. The ions were produced by a nano-electrospray ionization (n-ESI) source and transferred through a metal capillary and three consecutive inline molecular skimmers into a temperature-controlled octupole ion trap (pre-trap) kept at room temperature. Glycosyl cations were formed via in-source fragmentation. To perform the glycosylation reaction, the pre-trap was filled with the vapor of a nucleophilic acceptor (either MeOH, EtOH, iPrOH, BnOH, or TFE), which was continuously introduced through a leak valve. The vapor pressure was adjusted to maximize glycosyl product. In each cycle of the measurements the ionized synthetic standards were trapped and accumulated during 50 ms in the pre-trap filled with N_2 for the glycosylation reaction. Subsequently, the ions were released from the pre-trap, the product of the reaction or synthetic standard was mass-selected by a quadrupole mass filter and guided into a cryogenic octupole ion trap (CIT), which was kept at 6 K. The arrived ions were trapped and cooled in CIT by collisions with He gas, which was pulsed into the trap through a solenoid pulsed valve, approximately, one ms before arrival of the ions. In about 10 ms the already cold ions were interrogated by a UV laser pulse of a wavelength-tunable UV laser. The remaining parent and the appearing fragment ions were, finally, released from CIT and detected by a second quadrupole mass filter equipped with a channeltron detector. To increase the detected number of fragment ions the resolution of the second quadrupole mass filter was reduced and its central *m/z* value was selected such that to detect all different highly abundant fragment ions in a wide *m/z* range, while fully discriminating the transmittance of the heavier parent ones. The whole cold ion mass spectrometer operates in a 20 Hz cycle, while the laser is fired at 10 Hz repetition rate to organize the alternative laser-on/off cycles of the measurements. In the laser-off cycles of the measurements the second quadrupole mass-filter is switched from the broadband detection of the fragments to the detection of the parent ions only. The measured number of parent ions is then used for normalization of the fragment ion signal to account for instability of the ion source.

For UV spectroscopy, the UV pulses of 5-6 ns duration were produced by frequency-doubling the output of a tunable dye laser, which, in turn, was pumped by the frequency-tripled output of a Nd:YAG laser (355 nm.) The pulse energy, typically, remains within the 2-2.5 mJ interval throughout the entire scanned range of the dye laser; the average power of UV light was

recorded by a broadband pyroelectric detector along with the fragment ion signal for its further power normalization. The UV light has a 0.15 cm^{-1} spectral linewidth; the wavenumber of the dye laser is continuously measured by a wavemeter with ±0.05 cm^{-1} accuracy. Each data point was averaged over 10 measurements, and each spectrum was recorded, at least, three times to ensure its reproducibility.

The library-based quantification of the two anomers in the product of the glycosylation reaction involves a preliminary measurement of UV spectra of each of the two anomerically pure species, and a spectrum of the unknown product of the glycosylation reaction. This spectrum then is decomposed into a linear combination of the two reference spectra with two variable non-negative coefficients, normalized to 1. The coefficients of the decomposition that results in the minimum residual error from the experimental spectrum give the relative concentrations of the anomers.

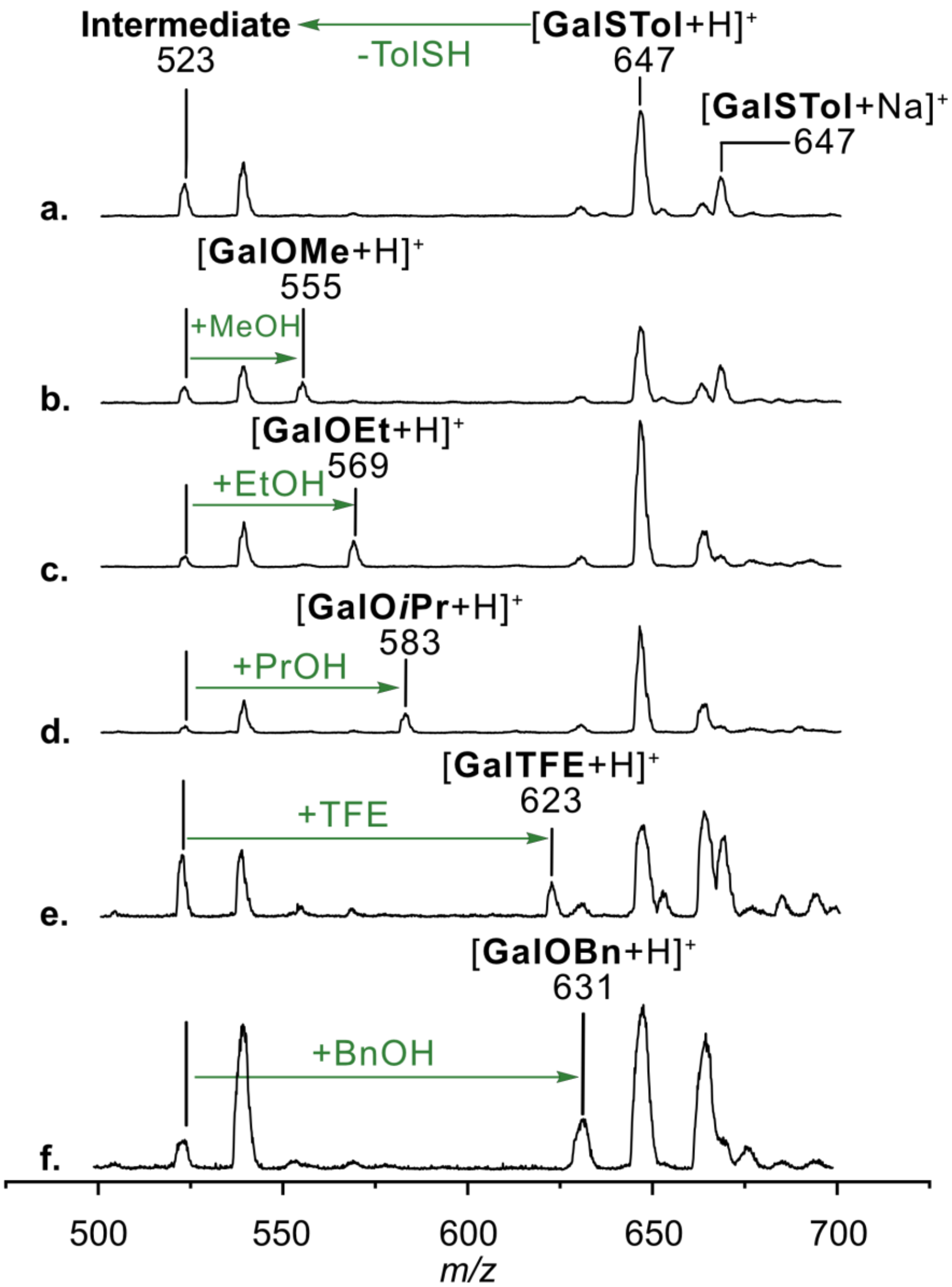

Figure S2. Mass spectra of (a) formation of glycosyl cation intermediate via in source fragmentation of the **GalSTol** precursor and product of gas-phase glycosylation reaction (b) methyl galactoside (**GalOMe**), (c) ethyl galactoside (**GalOEt**), (d) isopropyl galactoside (**GalOPr**), (e) 2,2,2,-trifluoroethyl galactoside (**GalTFE**), and (f) benzyl galactoside (**GalOBn**).

2.3 Characterization of Gas-phase Glycosyl Product using Cold Ion Spectroscopy on Methyl Galactoside

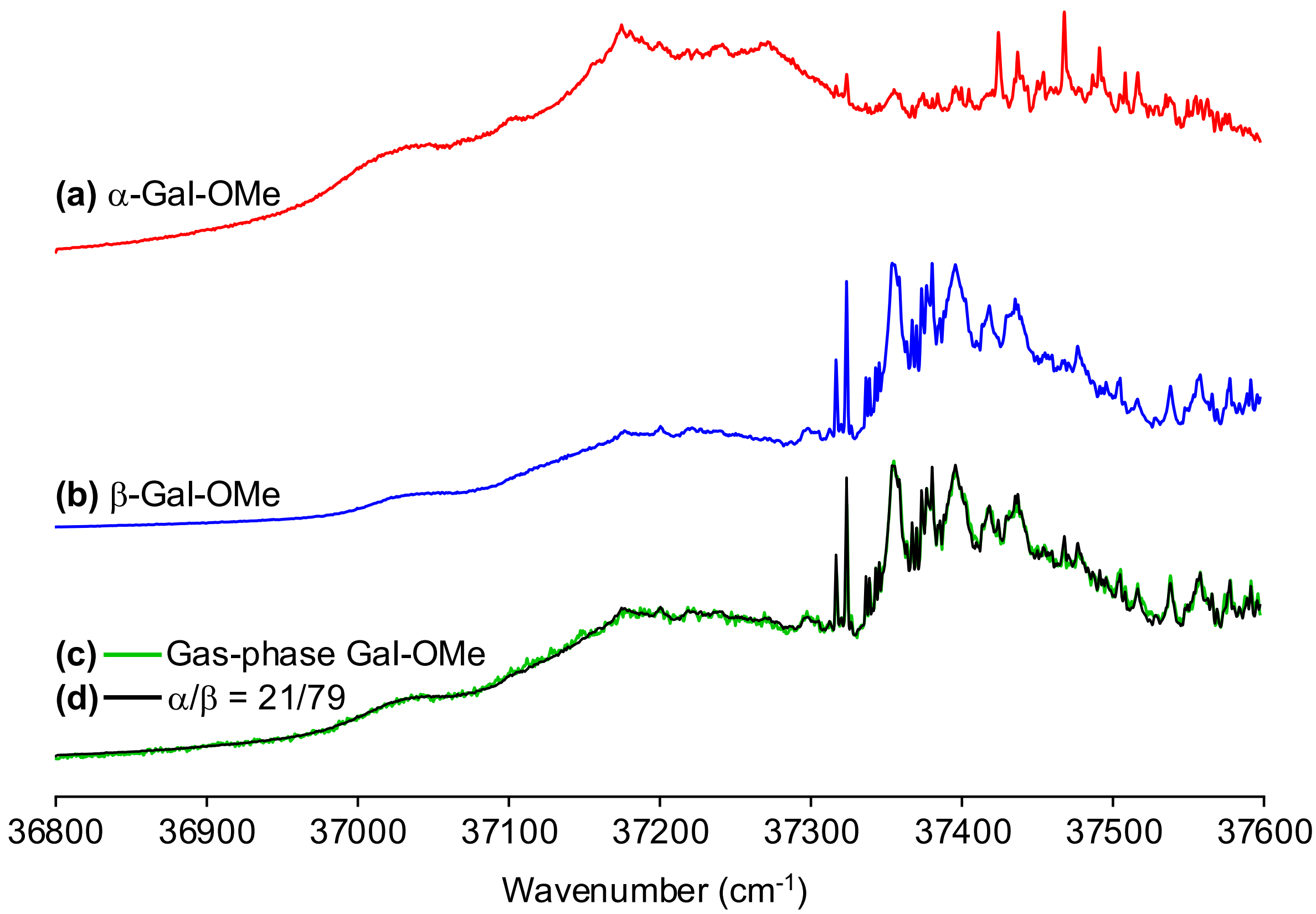

Figure S3. UVPD spectra of (a) α- and (b) β-methyl glycoside (GalOMe) synthetic standards and (c) the product of gas-phase glycosylation. The spectrum of the product of gas-phase glycosylation is fitted (d) by the sum of spectra in (a) and (b) with the coefficients (α/β = 21/79) derived by the numerical decomposition. The stereoselective outcome of the gas-phase glycosylation reaction was determined by fitting the UVPD spectrum with a linear combination of UVPD spectra acquired from synthetic α-GalOMe and β-GalOMe standards. The UVPD spectra of the pure standards show distinct and diagnostic bands in the spectral range from 37100 to 37600 cm^{-1}. Specifically, two sharp absorptions at 37316 and 37323 cm^{-1} and a complex signature at 37356 cm^{-1} comprise the characteristic pattern of the β-anomer (blue). The α-anomer (red) is

characterized by a broad signal that extends from 37100 to 37300 cm^{-1} and sharper bands at 37424 and 37468 cm^{-1}. These spectral signatures serve as precise fingerprints to fit the spectrum of the gas-phase glycosylation products (black). A linear regression fit reveals the α/β ratio of 21/79 in gas-phase glycosylation, indicating a high β-selectivity at room temperature. Details regarding experimental procedures are provided in Sections 2.1 and 2.2. Methodologies for error analysis are described in Section 3, and the Python-based deconvolution script is provided in the Supporting Information (File: 'deconvolution script').

3. Error Estimation For Stereoselectivity in Gas-phase Glycosylation Reactions

In library-based quantification of the relative concentration of mixed in solution isomers, we usually assess the accuracy by performing measurements of 2D UV-MS (or UV only) fingerprints for a statistically large set of solution mixtures of isomers of known prepared concentrations, each time calculating the relative concentrations of the isomers.[12-19] The statistical uncertainty of concentrations for a considered number of isomers in question is then reported as root mean square deviation (RMSD) for the (calculated-mixed) differences.[12-19] Herein, this was not possible to perform due to, except for one pair of isomers, a lack of synthetic standards with known relative concentrations. Instead, the likely error in the determined relative concentration of isomers in the experiments herein was estimated based on three factors: (i) signal-to-noise (S/N) ratio in the measured fingerprints, (ii) fit quality, quantified by error for the liner fit coefficients with these fingerprints, (iii) difference between UV spectra of ions in each pair of isomers. We then compared these factors for the studied herein isomeric pairs with the numerous data obtained in our earlier measurements for different molecules.[14,16-19] The comparisons suggest that, for the quality of the reported herein spectra, the inaccuracy of fit coefficients is, by far, a less important factor than the spectral similarity. Our analysis of the previous data for pairs of isomers, whose spectral difference is similar to that of the studied herein molecules, yields RMSD values in the range of 1.5 % to 4.3 %, with a statistically averaged value of 3.1%. We, therefore, take 6.2 % as a doubled RMSD (95% confidence level) value for the determined herein relative concentrations of the isomers. This is consistent with the accuracy of ± 3.62 %, which was directly determined for the only available pair of isomers with known relative concentrations (Figure S8). It is worth noting that, the evaluated above ± 6.2% statistical accuracy is by far higher than the threshold ± 20 % error that, potentially, could affect the conclusions of this work.

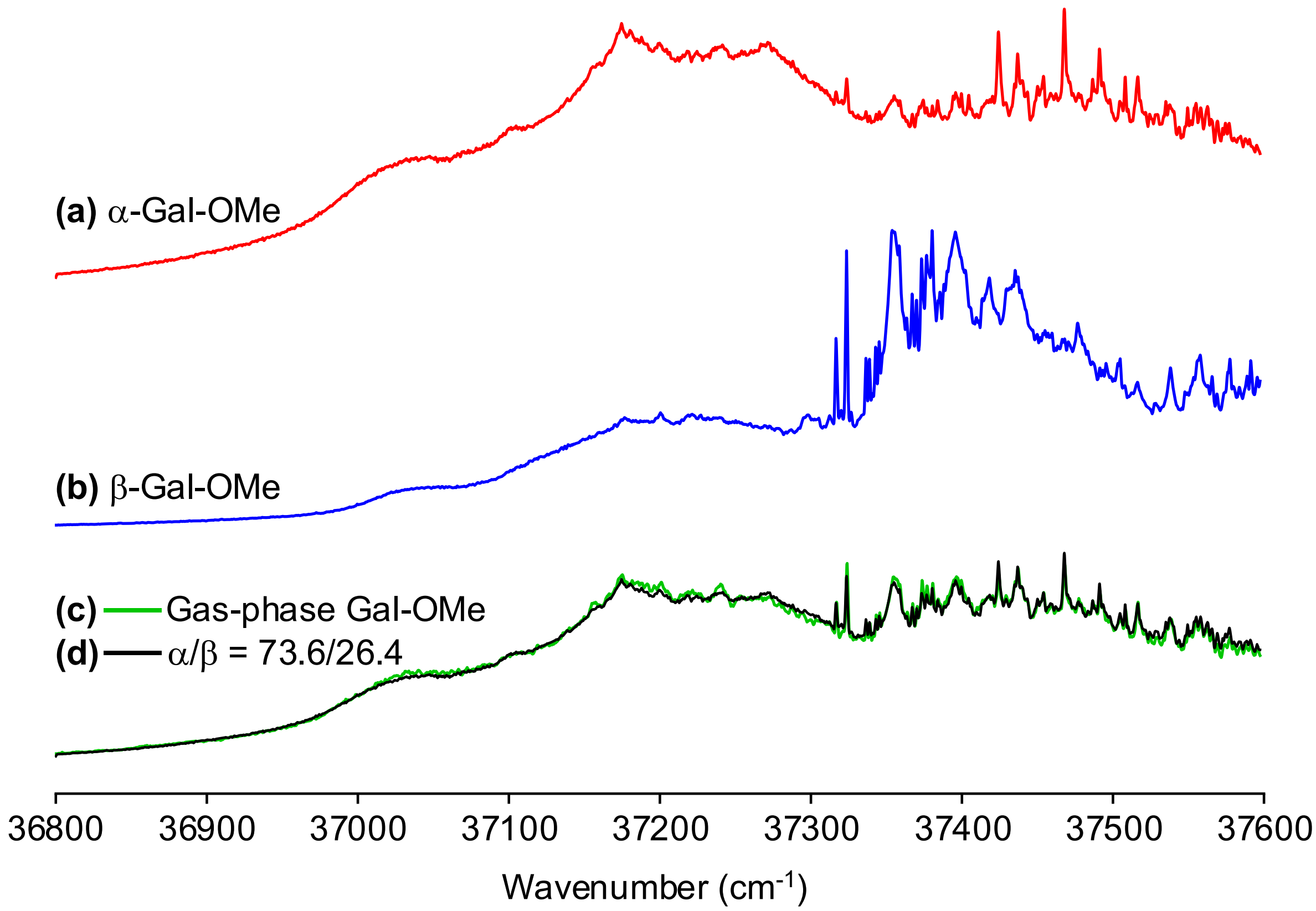

Figure S4. To validate the accuracy of the UV deconvolution method, a mixture of α- and β-methyl glycoside was manually prepared in a 70/30 ratio, as determined by NMR. It displays the UVPD spectra of (a) the α-methyl glycoside standard, (b) the β-methyl glycoside standard, and (c) their 70/30 mixture. The spectrum of the mixture is fitted using a weighted sum of the standards from (a) and (b). Numerical decomposition yielded a ratio of α/β = 73.6/26.4, demonstrating the reliability of the UVPD-based fitting compared to the NMR-validated benchmark. Details regarding experimental procedures are provided in Sections 2.1 and 2.2. Methodologies for error analysis are described in Section 3, and the Python-based deconvolution script is provided in the Supporting Information (File: 'deconvolution script').

4. Stability Test of Glycosyl Product

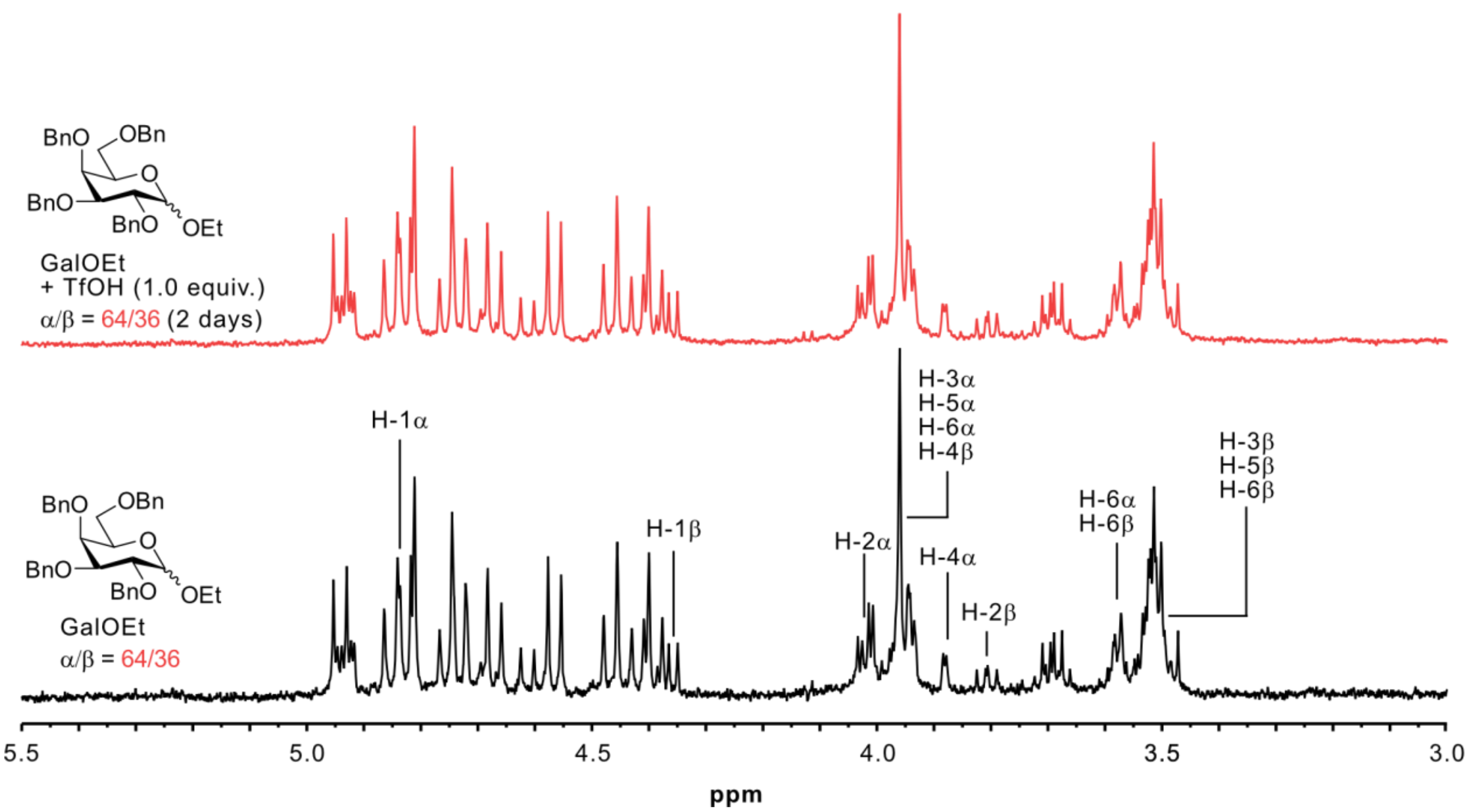

Figure S5. Stability of ethyl galactoside under acidic conditions. In the NMR analysis, ethyl galactoside was first dissolved in d-chloroform. The ratio of α- to β-glycoside was determined to be 64/36. After treatment with triflic acid (TfOH), this ratio remained unchanged for two days. These results indicate that both α- and β-glycosides are stable, with no anomerization/equilibrium occurring in the solution.

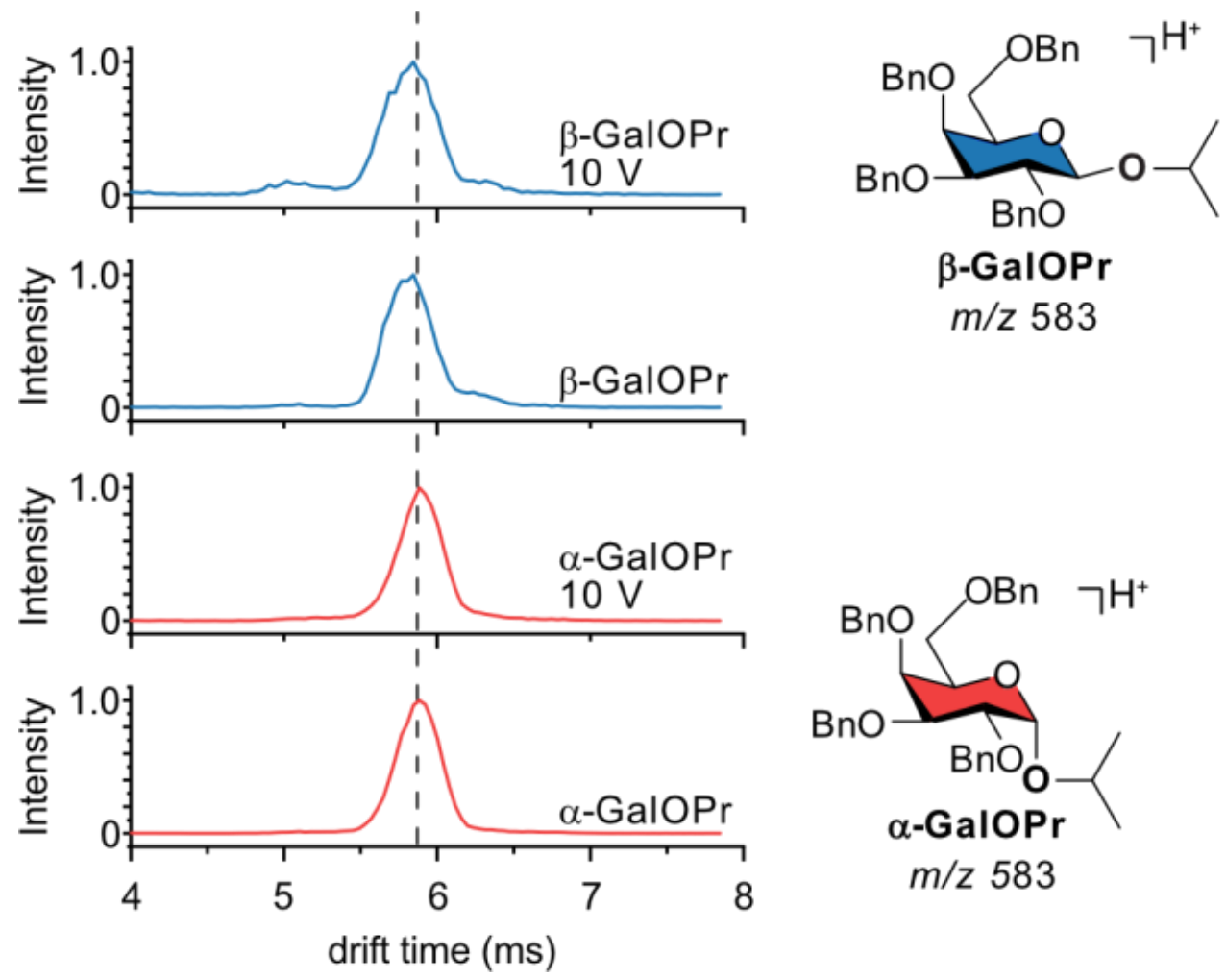

Figure S6. Stability test of the isopropyl glycoside in the gas phase. Traveling-wave ion mobility mass spectrometry (TWIM-MS) was applied to evaluate the gas-phase stability of the glycoside product. Although the resolving power of the instrument likely could not resolve these from a 50:50 mixture, the mobiligram suggests that the α- and β-anomers can be roughly distinguished based on their distinct mobiligram shapes and the centroid of peaks in their arrival time distributions. In the activation step, the collision voltage was ramped beyond 10 V; however, no significant peak shifting or broadening was observed. Instead, a strict decrease in the intensity of the protonated precursor ion occurred. The data suggests that α- and β-anomerization is likely minor. This is supported by literature precedent,[20,21] α- and β-equilibration requires a free, unprotected anomeric hydroxyl group (-OH) to proceed via a low-energy, ring-opening/ring-closing mechanism (mutarotation). Because the anomeric center of GalOPr is locked as an isopropyl group, the hemiacetal framework is highly stable. Consequently, increasing the collision shifts the reaction threshold toward glycosidic bond cleavage, yielding glycosyl cation intermediates rather than anomeric interconversion.[22,23]

5. Characterization of Gas-phase Glycosyl Product using Cold Ion Spectroscopy and NMR on Galactoside with Different Nucleophiles

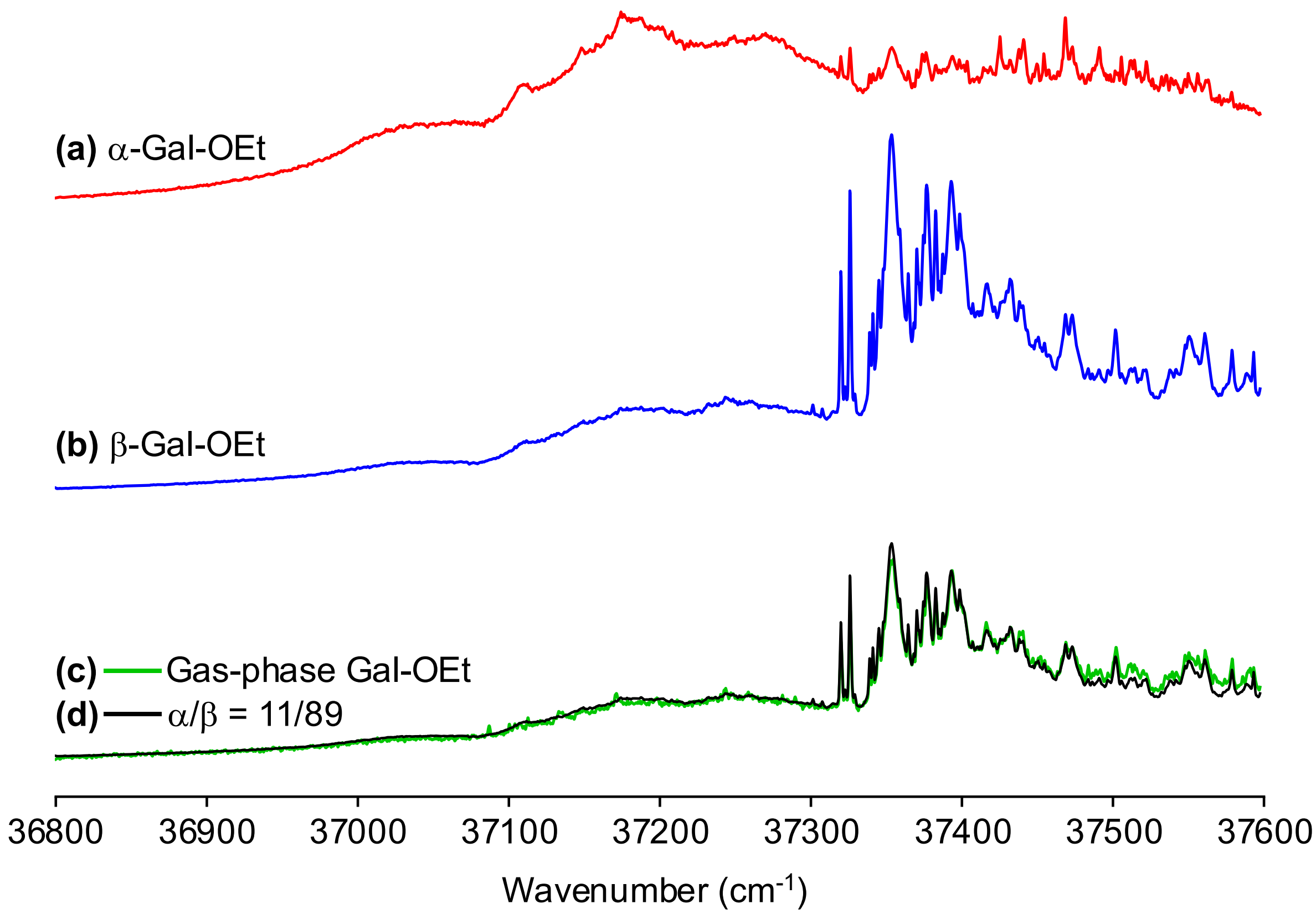

Figure S7. UVPD spectra of (a) α- and (b) β-ethyl glycoside (GalOEt) synthetic standards and (c) the product of gas-phase glycosylation. The spectrum of the product of gas-phase glycosylation is fitted (d) by the sum of spectra in (a) and (b) with the coefficients (α/β = 11/89) derived by the numerical decomposition. Details regarding experimental procedures are provided in Sections 2.1 and 2.2. Methodologies for error analysis are described in Section 3, and the Python-based deconvolution script is provided in the Supporting Information (File: 'deconvolution script').

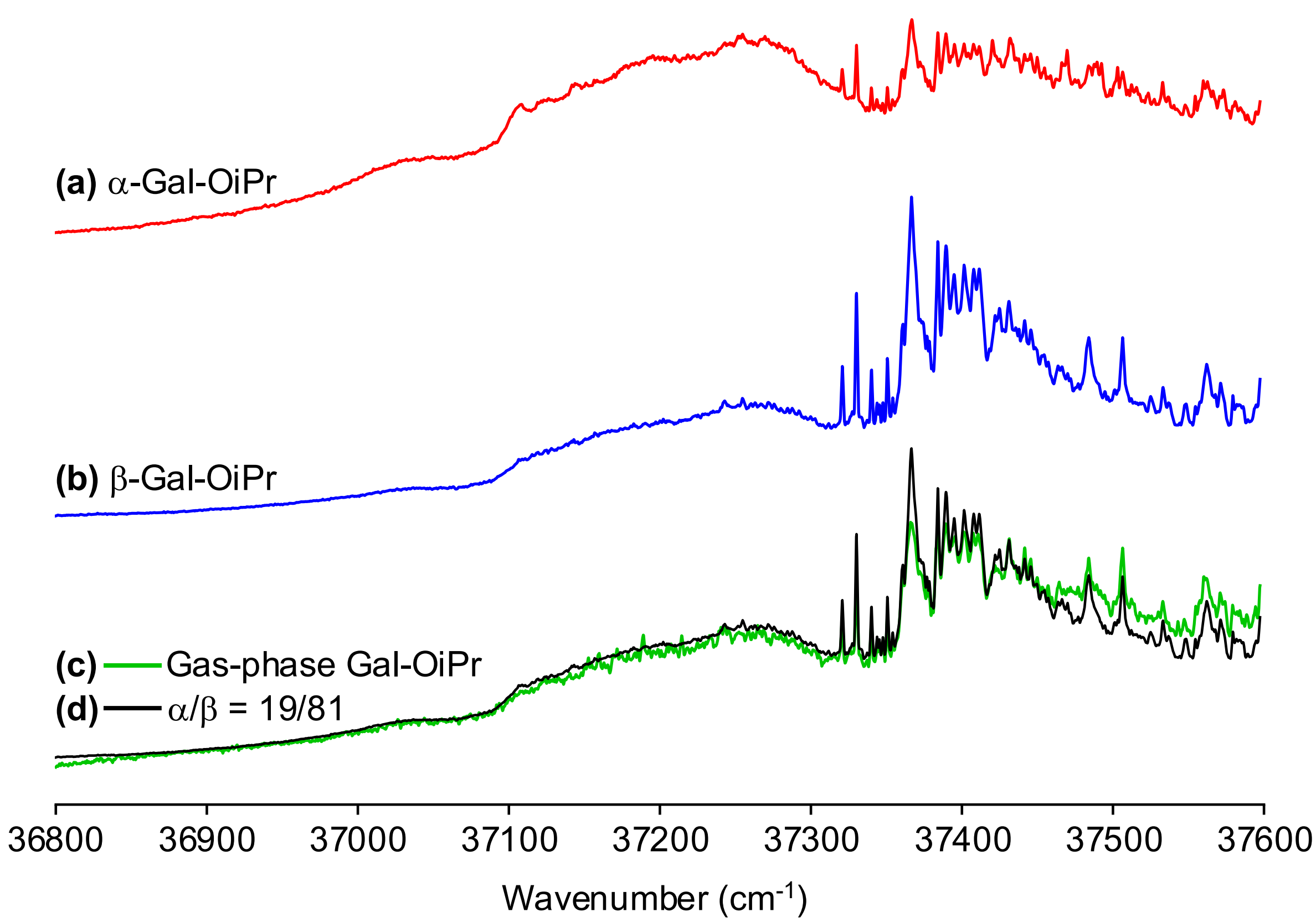

Figure S8. UVPD spectra of (a) α- and (b) β-isopropyl glycoside (GalOPr) synthetic standards and (c) the product of gas-phase glycosylation. The spectrum of the product of gas-phase glycosylation is fitted (d) by the sum of spectra in (a) and (b) with the coefficients (α/β =19/81) derived by the numerical decomposition. Details regarding experimental procedures are provided in Sections 2.1 and 2.2. Methodologies for error analysis are described in Section 3, and the Python-based deconvolution script is provided in the Supporting Information (File: 'deconvolution script').

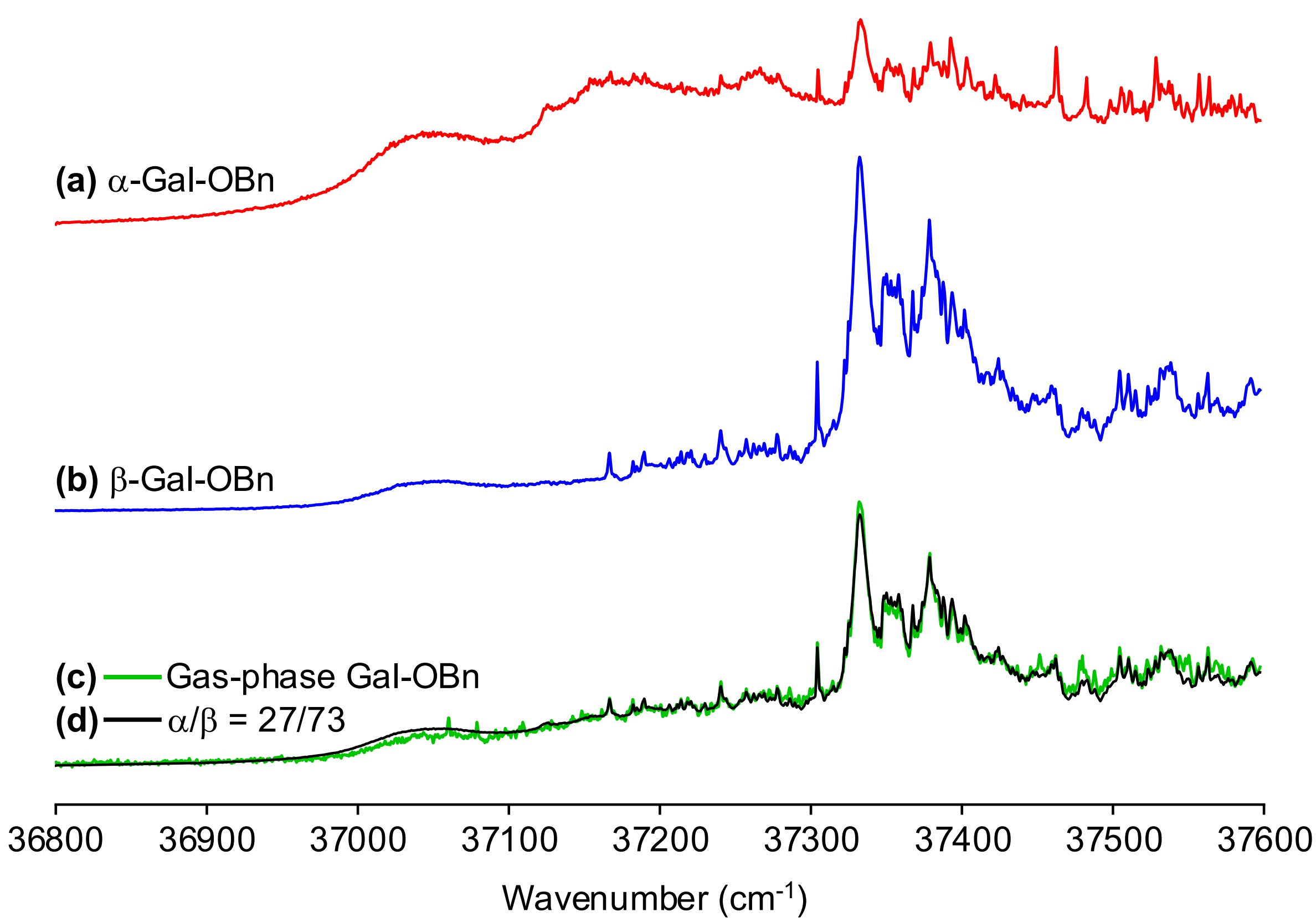

Figure S9. UVPD spectra of (a) α- and (b) β-GalOBn synthetic standards and (c) the product of gas-phase glycosylation. The spectrum of the product of gas-phase glycosylation is fitted (d) by the sum of spectra in (a) and (b) with the coefficients (α/β = 27/73) derived by the numerical decomposition. Details regarding experimental procedures are provided in Sections 2.1 and 2.2. Methodologies for error analysis are described in Section 3, and the Python-based deconvolution script is provided in the Supporting Information (File: 'deconvolution script').

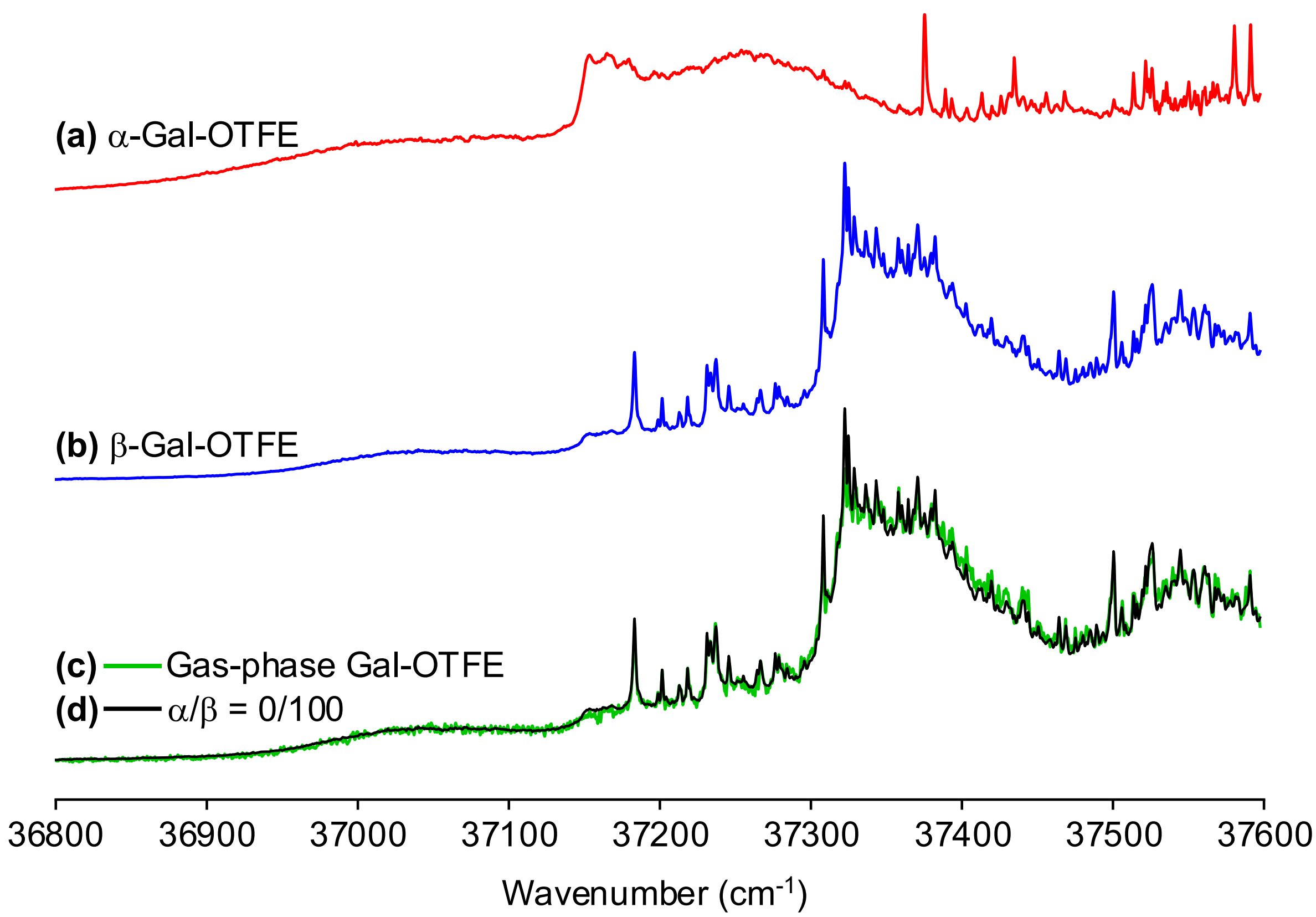

Figure S10. UVPD spectra of (a) α- and (b) β-GalTFE synthetic standards and (c) the product of gas-phase glycosylation. The spectrum of the product of gas-phase glycosylation is fitted (d) by the sum of spectra in (a) and (b) with the coefficients (α/β = 0/100) derived by the numerical decomposition. Details regarding experimental procedures are provided in Sections 2.1 and 2.2. Methodologies for error analysis are described in Section 3, and the Python-based deconvolution script is provided in the Supporting Information (File: 'deconvolution script').

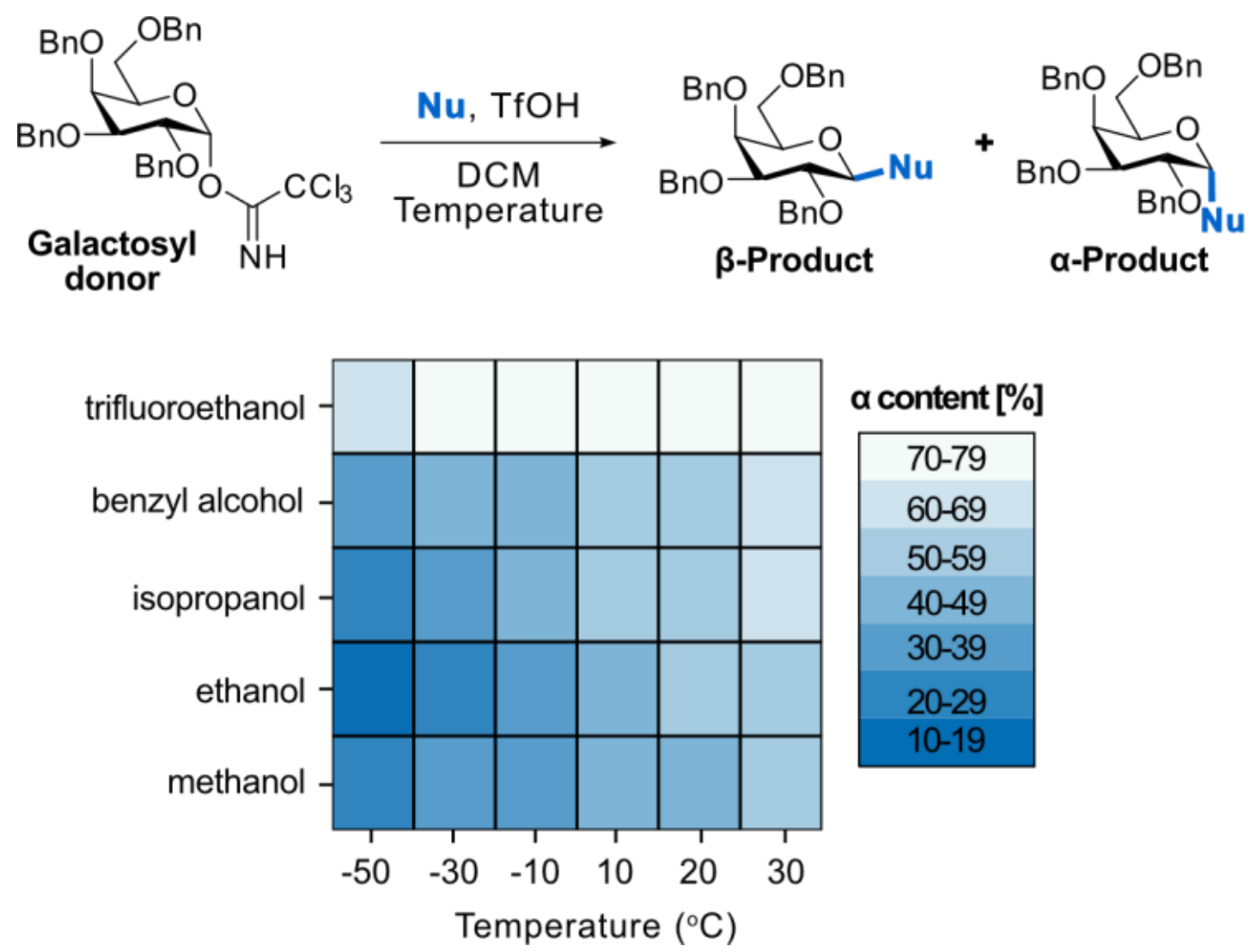

Figure S11. Chemical tests on glycosylation reactions. The stereoselective outcomes were probed using a range of nucleophiles and temperatures. The data revealed a transition from β- to α-selectivity with increasing temperature and weaker nucleophiles. This α-selectivity can be rationalized by a mechanistic shift from an S_N2 pathway toward S_N1,[24,25]. While these trends are evident, the fundamental contributions driving these mechanisms remain difficult to define; they are often obscured by structural and environmental variables, such as solvent effects. Consequently, relying solely on solution-phase selectivity can be misleading. To address this, we employ gas-phase reactions as a "clean room" model, enabling us to probe intrinsic stereoselectivity in an environment free from external perturbations.

6. Studying the Gas-phase Glycosylations on Mannose and Glucoside

For two other monosaccharides, namely mannose and glucose, analogous reactions were performed. For glucosides, the α- and β-anomer standards yielded indistinguishable cold UV spectra. For mannosides, the UV spectra of the detected ions do not match the standard references. Both observations hint at the formation of non-covalent cation-nucleophile complexes, making the corresponding spectra difficult to analyse.

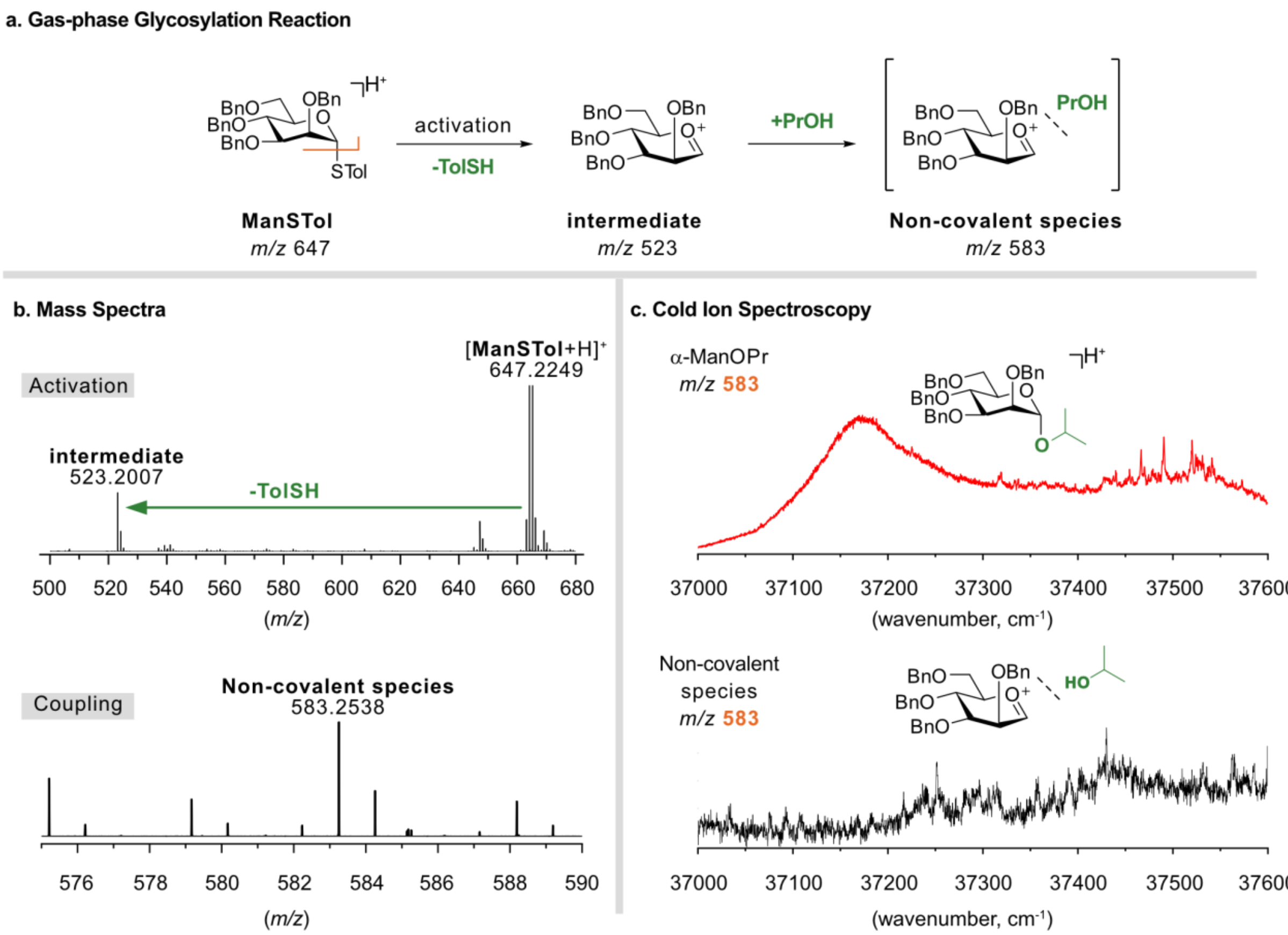

Figure S12. Gas-phase glycosylation reactions of mannose. The mannosyl cation (*m/z* 523) is generated from the thiomannoside precursor (*m/z* 647). Upon introduction of isopropanol, a new signal corresponding to the product ion (*m/z* 583) appears. However, cold UV spectroscopy reveals that the spectrum of this ion is distinct from that of the actual product ion. We therefore hypothesize that this ion represents a non-covalent complex that coincidentally has the same *m/z* as the product.

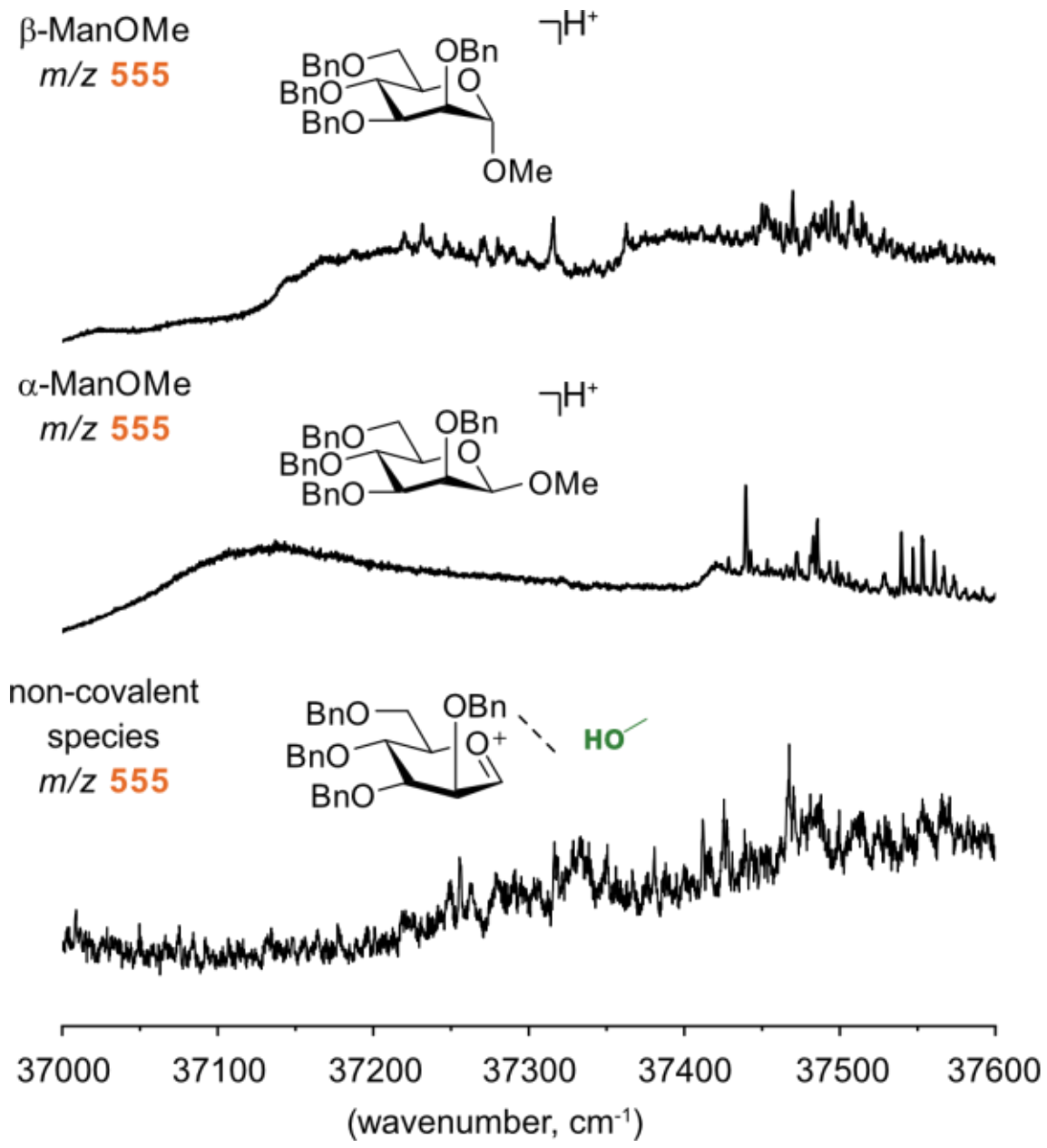

Figure S13. Gas-phase glycosylation reactions of mannoside and methanol. Activation of the thiomannoside generates the mannosyl cation, which subsequently interacts with methanol. The experiment yields a signal corresponding to the product ion (*m/z* 555). However, since the cold UV spectroscopy differs from that of the product ion prepared from the standard, we hypothesize that this signal arises from a non-covalent complex rather than a covalent product.

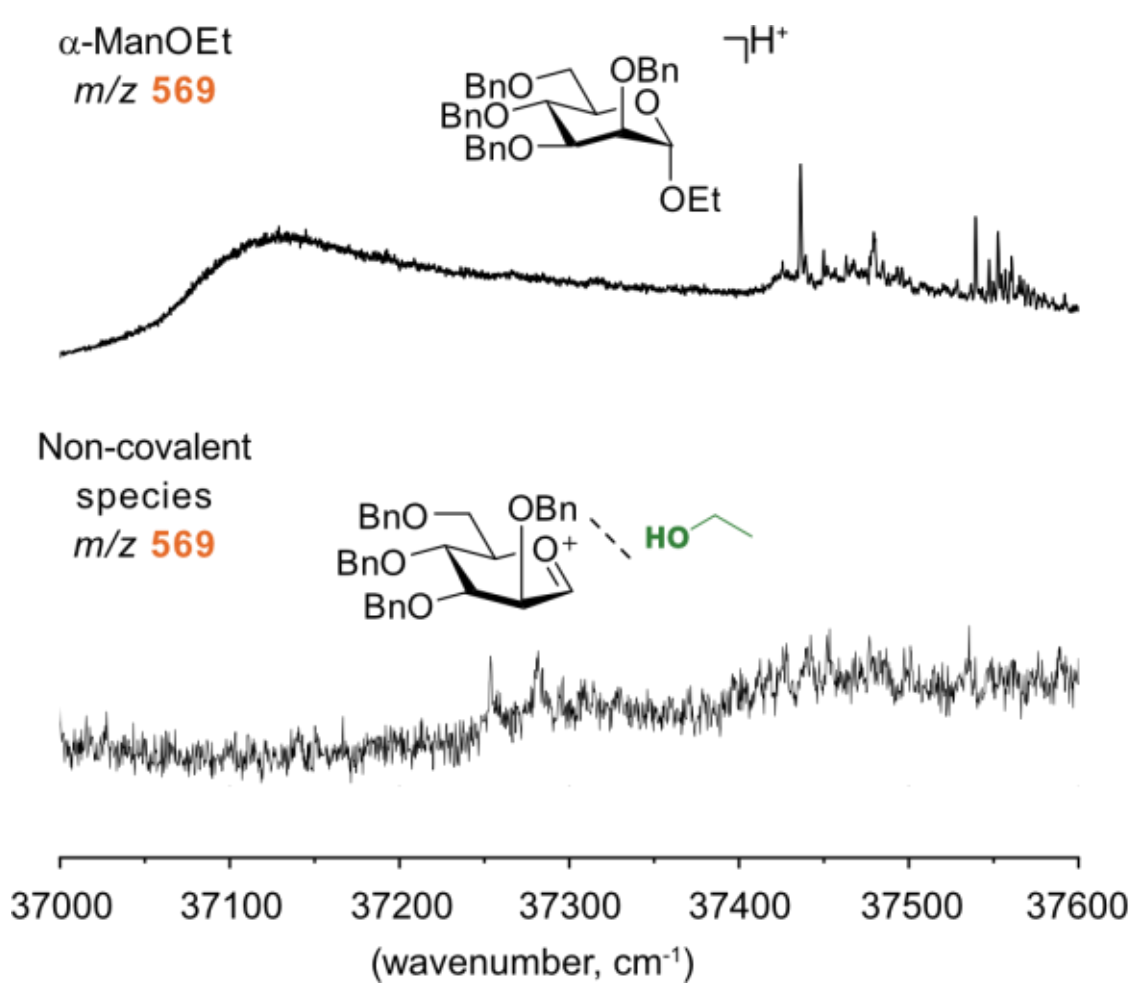

Figure S14. Gas-phase glycosylation reactions of mannoside and ethanol. Activation of the thiomannoside generates the mannosyl cation, which subsequently interacts with methanol. The experiment yields a signal corresponding to the product ion (*m/z* 569). However, since the cold UV spectroscopy differs from that of the product ion prepared from the standard, we hypothesize that this signal arises from a non-covalent complex rather than a covalent product.

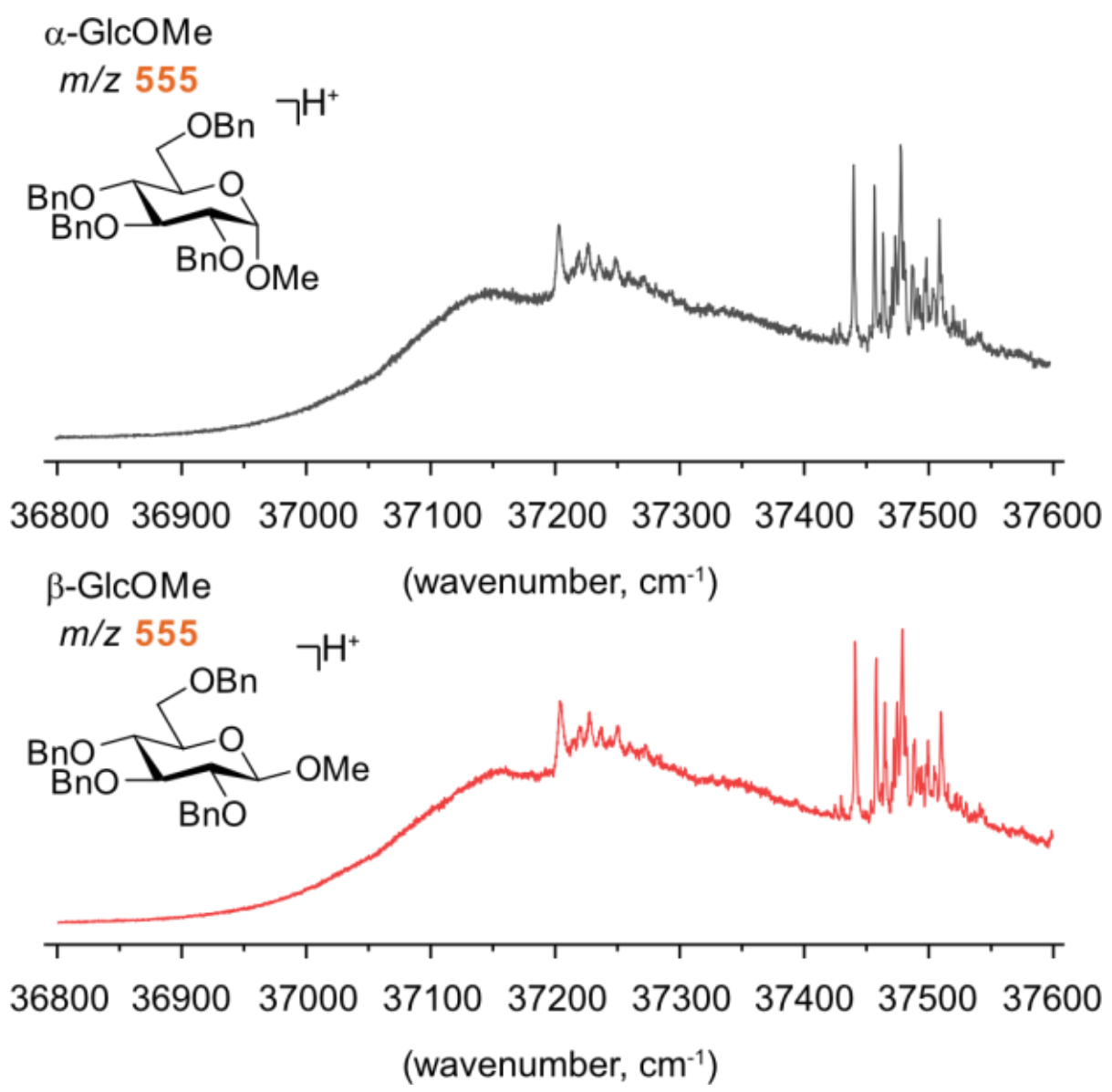

Figure S15. Cold UV spectroscopy of methyl glucoside (GlcOMe). Both α-anomer (α-GlcOMe) and β-anomer (β-GlcOMe) standards were prepared by chemical synthesis. Because the two anomers cannot be distinguished under these conditions, this system is ineffective for determining the in situ α/β ratio of the product from the ion–molecule reaction.

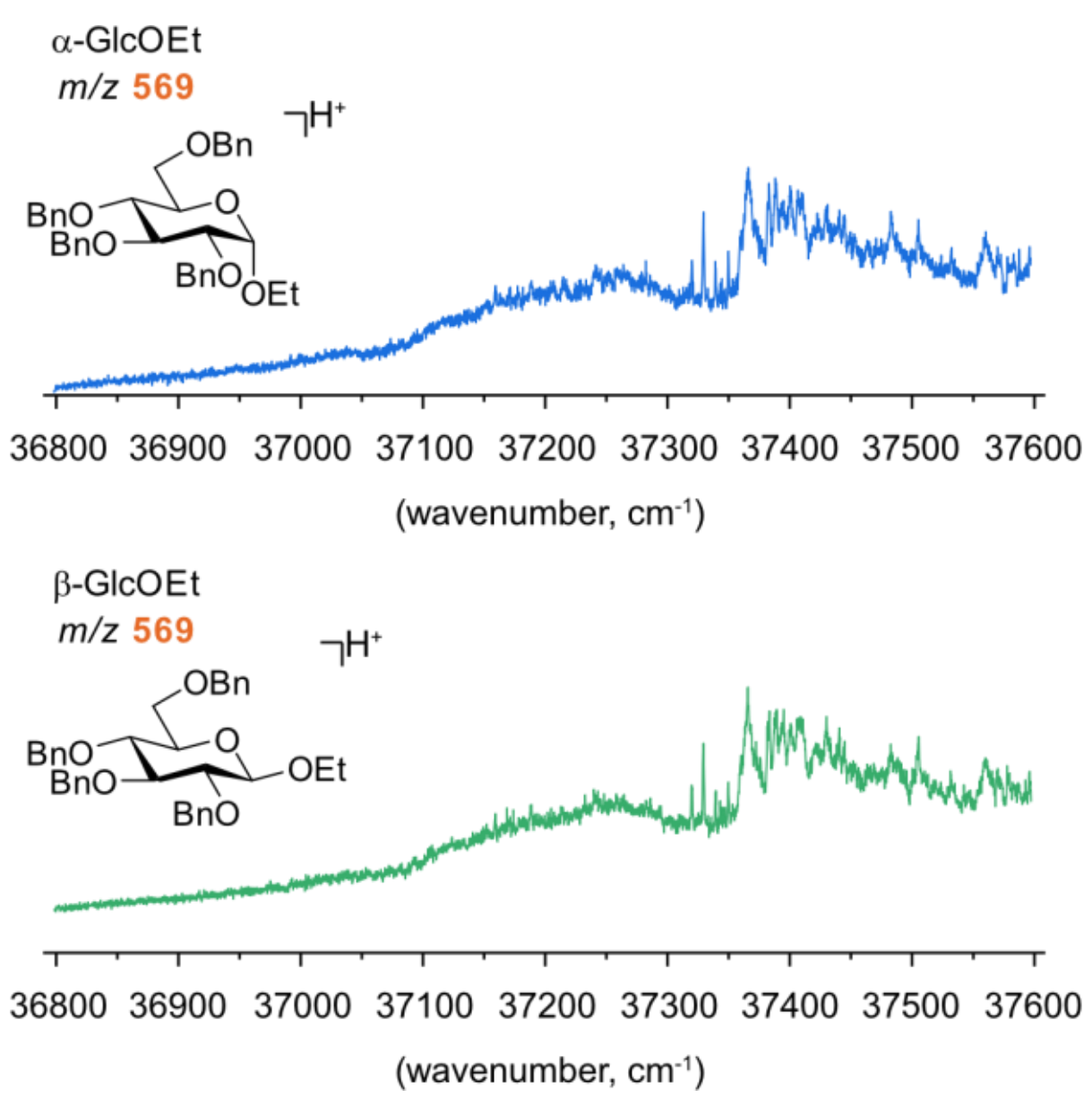

Figure S16. Cold UV spectroscopy of ethyl glucoside (GlcOEt). Both α-anomer (α-GlcOEt) and β-anomer (β-GlcOEt) standards were prepared by chemical synthesis. Because the two anomers cannot be distinguished under these conditions, this system is ineffective for determining the in situ α/β ratio of the product from the ion–molecule reaction.

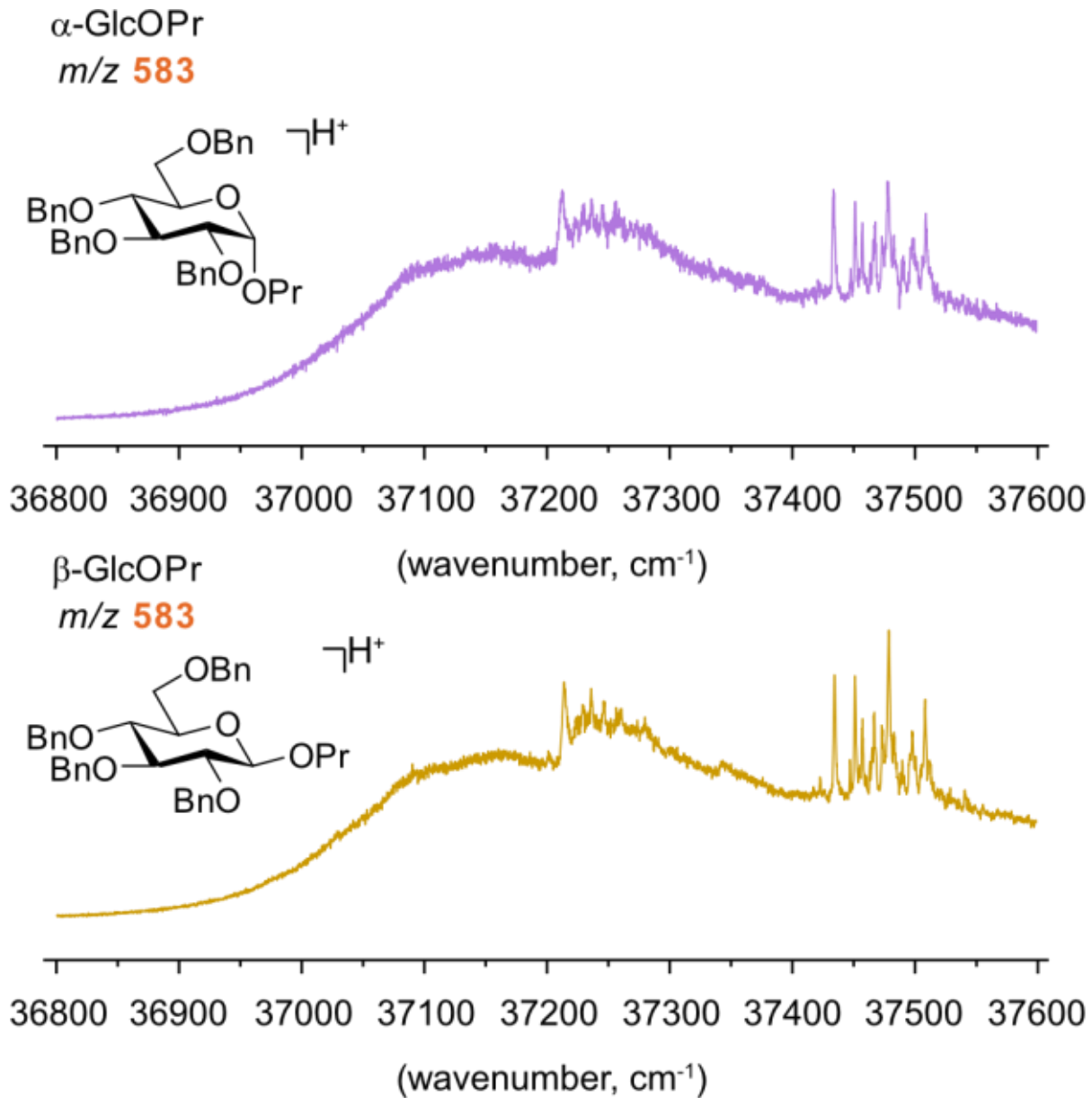

Figure S17. Cold UV spectroscopy of isopropyl glucoside (GlcOPr). Both α-anomer (α-GlcOPr) and β-anomer (β-GlcOPr) standards were prepared by chemical synthesis. Because the two anomers cannot be distinguished under these conditions, this system is ineffective for determining the in situ α/β ratio of the product from the ion–molecule reaction.

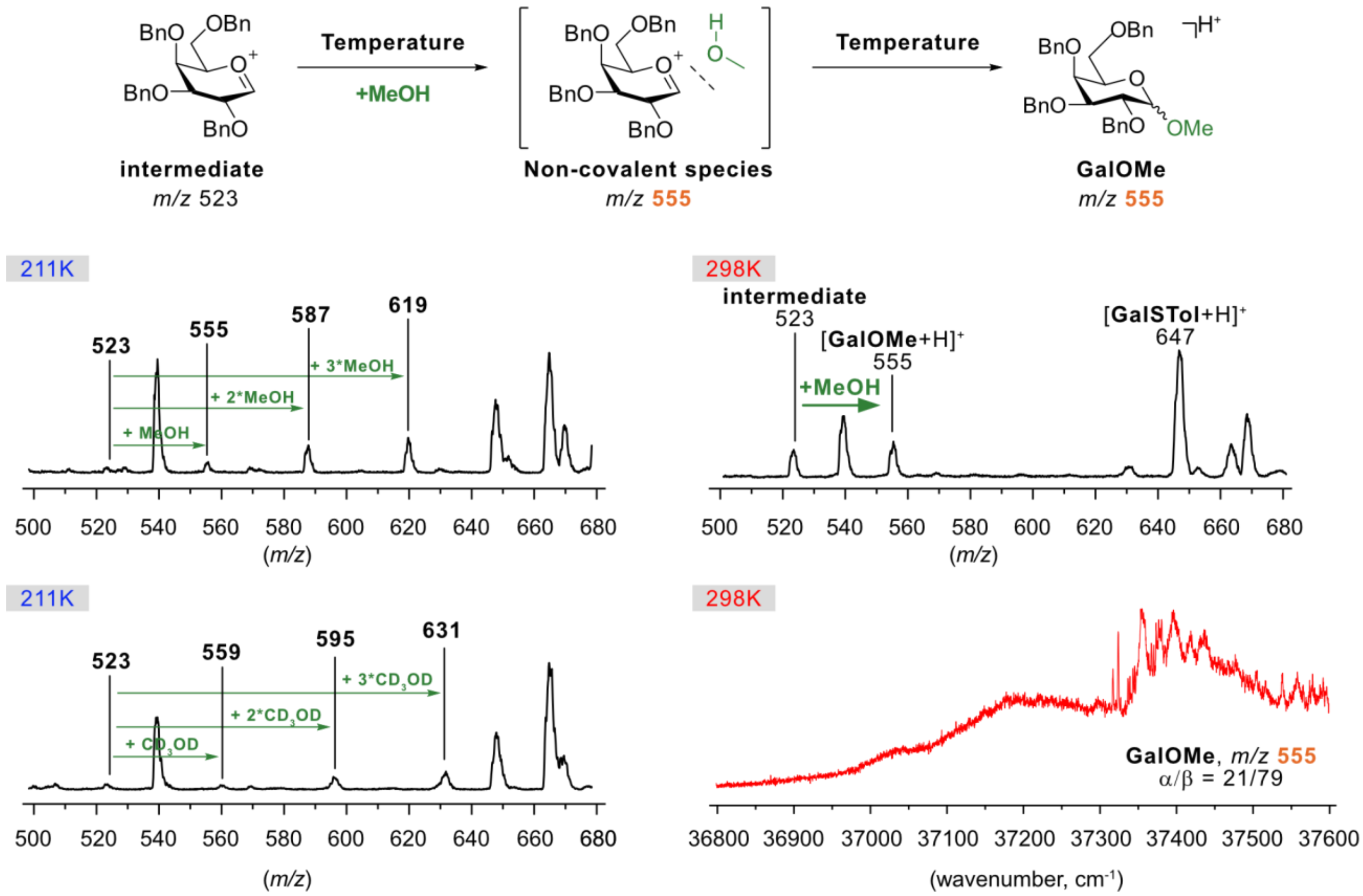

Figure S18. Studying the temperature effect on gas-phase ion reactions. The reaction proceeds through a two-step S_N1-like pathway. First, the oxocarbenium ion forms a discrete and associated adduct with methanol to form a non-covalent species. Secondly, this pair of species forms a covalent bond at the anomeric position, yielding a glycosyl product. The outcome is strongly temperature dependent. At 298 K, the protonated product GalOMe is formed at *m/z* 555, and linear regression of the cold UV spectrum indicates an α/β ratio of 21/79. At 211 K, the ion at *m/z* 555 was isolated and probed by cold UV spectroscopy; however, its spectrum differs from that of the authentic product prepared from a standard. These results suggest that at 211 K, glycosylation does not proceed to covalent bond formation. Instead, one to three methanol molecules may condense and associate non-covalently with the glycosyl cation. Furthermore, when deuterated methanol was used, a corresponding mass shift of +4 Da was observed. Based on these data, we propose that at low temperature, a non-covalent species is formed through cation–dipole interactions between the glycosyl cation and the nucleophile (alcohol).

7. Structure Analysis of Oxocarbenium Ion

Method Description

Initial geometries of oxocarbenium ion were constructed by chemical intuition using GaussView 6.[26] Conformational search were performed using CREST[27] with the semiempirical method GFN2-xTB[28] using default settings. The selected structures are reoptimized and their harmonic frequencies are computed at the PBE1PBE/6-311+G(d,p) EmpiricalDispersion=GD3BJ[29,30] level of theory using Gaussian 16.[26] Relative free energies at 90 K (approximate temperature of the ion trap) were extracted from the frequency calculation.

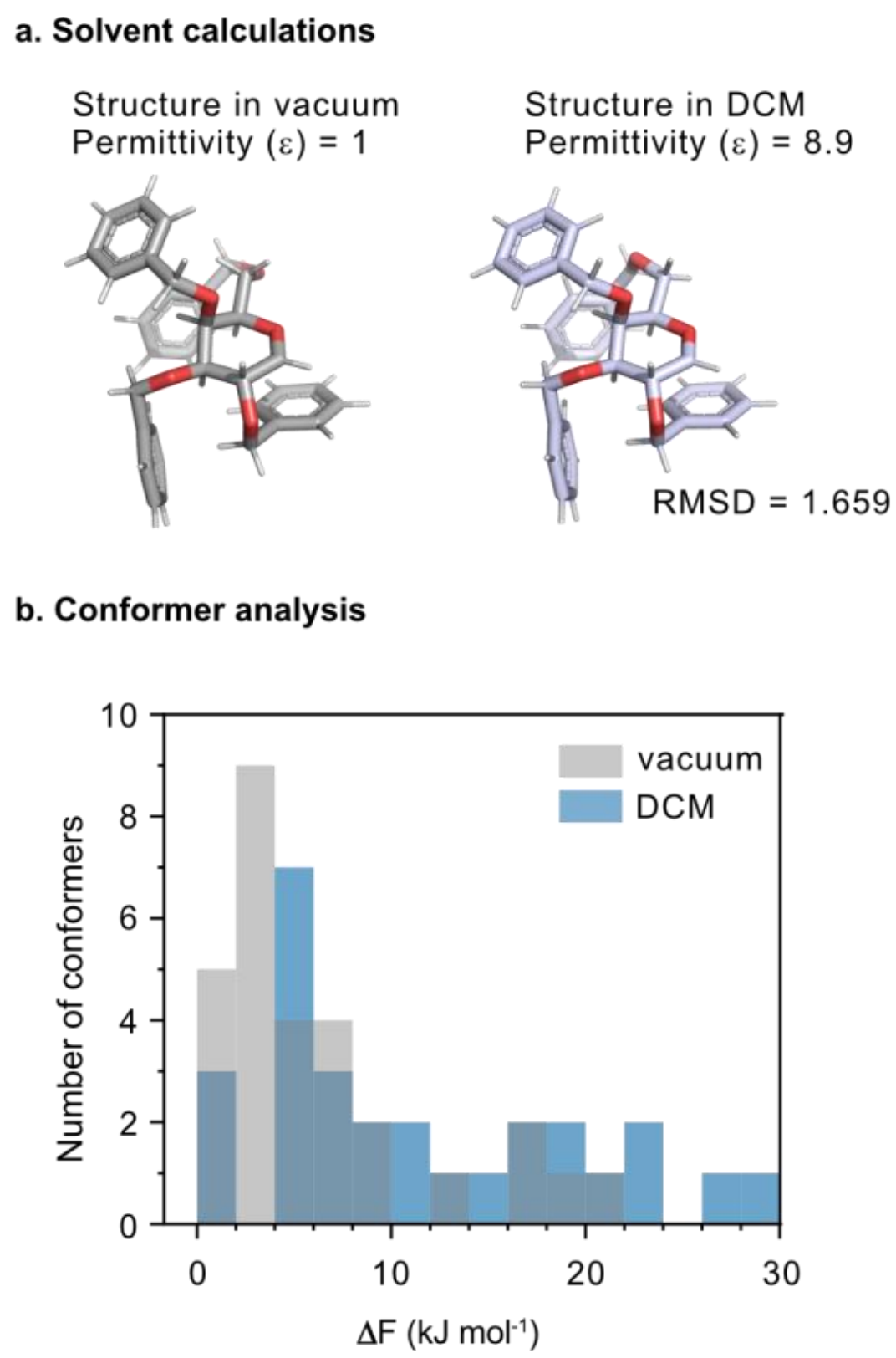

Figure S19. Structure analysis of the oxocarbenium ion in vacuo and DCM. Conformational analysis of the galactosyl cation. a) Conformer sampling. Thirty conformers were computed using a solvent model, and the most stable one was selected. The lowest-energy structure in DCM is similar to the gas-phase structure, revealing an RMSD of 1.659. b) Conformational distribution. In vacuum, more low-energy conformers are present, whereas in DCM the distribution shifts toward higher-energy conformers. This agrees with earlier results, showing that intermediates are stabilized in solvents with higher permittivity.[31]

Conformation of glycosyl cation:

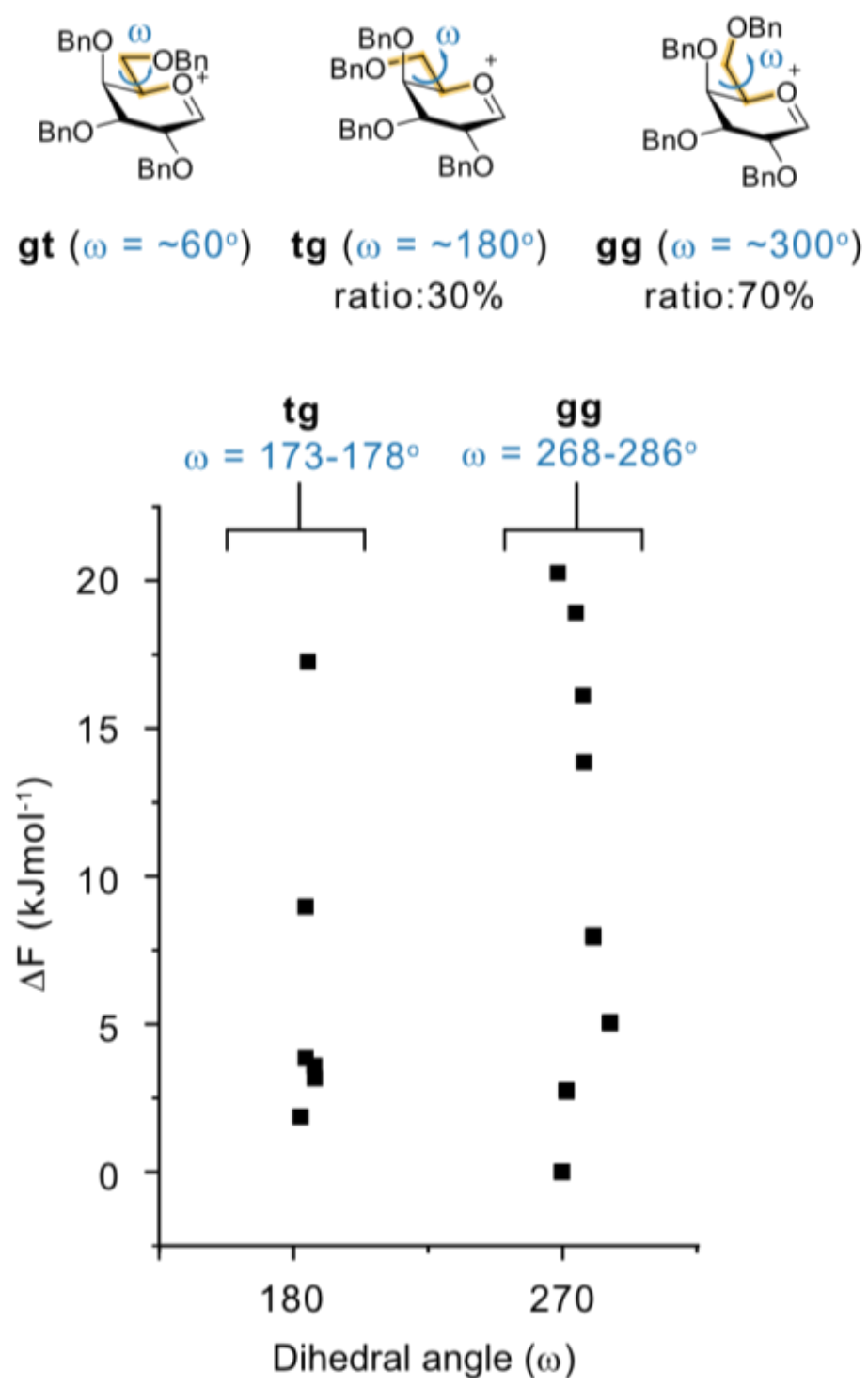

Figure S20. Conformation analysis of the galactosyl cation. Gas-phase structures of perbenzylated galactosyl cations have been previously studied using cryogenic infrared ion spectroscopy, suggesting C6-OBn participation in the intermediate structure.[32] Analysis of the C5-C6 dihedral angle reveals a dynamic conformational equilibrium between the gauche-gauche (*gg,* 70%) and trans-gauche (*tg,* 30%) conformers, which lie within a narrow free energy window of 0-20 kJ mol^{-1}. This rapid interconversion governs high-energy oxocarbenium ion formation, aligning with Crich's established rotamer models.[33,34] Specifically, the *tg* conformation favors nucleophile attacking at the β-face, and this β-selectivity is driven by an intramolecular hydrogen bond and a low-energy transition state.[33,34] While *tg* conformations occur less abundant and at slightly higher energies than *gg* conformations, the energetic differences are minor, and the influence of the *tg* conformations might hence explain the experimentally observed gas-phase β-selectivity. In contrast, the energetically preferred *gg* conformation features a shorter C1-O6 distance, inducing C6-OBn participation. The resulting C6-cyclic cations, as found in our previous work, shield the β-face and account for α-selectivity.[32] Further investigations are warranted to decipher the influence of oxocarbenium structure and conformation on stereoselectivity *in vacuo* and solution, for example by taking kinetic barriers into account.

8. General Procedure for Chemical Glycosylation Reaction

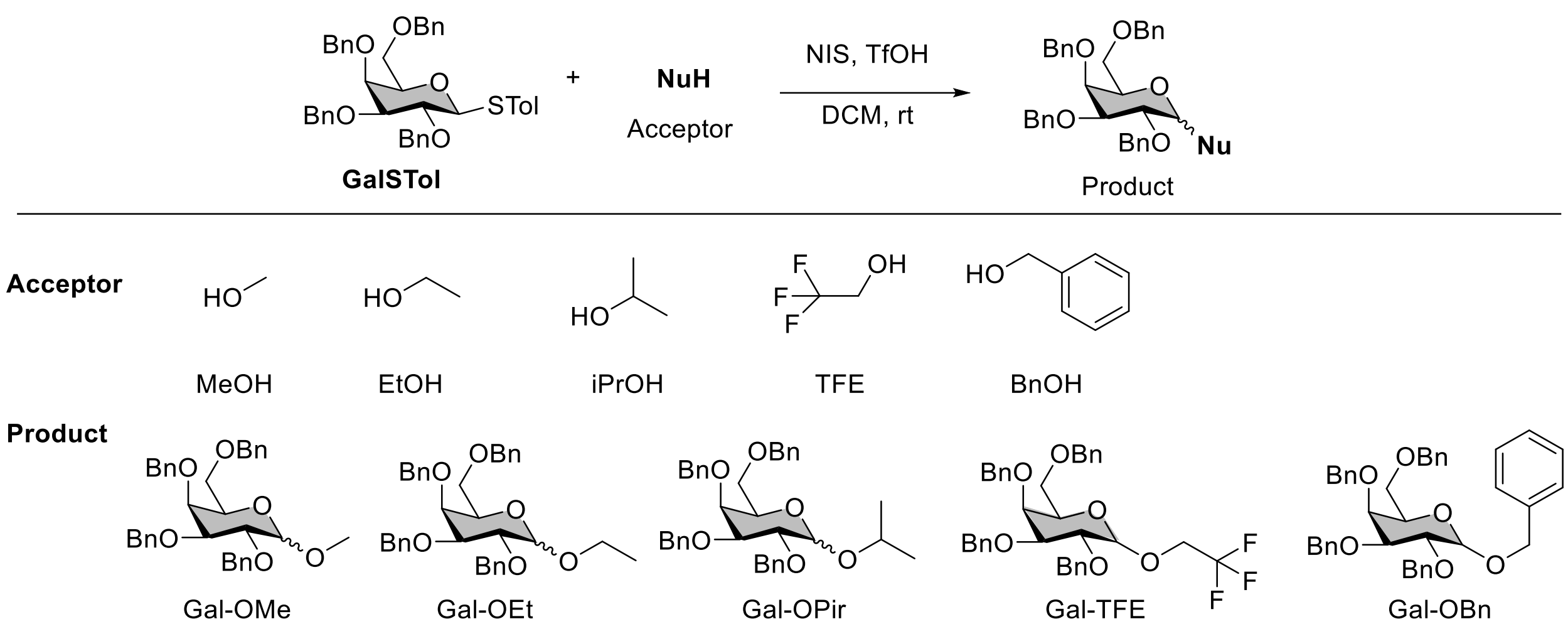

Thioglycoside building blocks are widely used in oligosaccharide synthesis due to their chemical stability during storage and high reactivity in solution-phase reactions.[35] Unwanted side reactions can be prevented by adding protecting groups that mask reactive functional groups, enabling precise control over the reaction site. To study the stereoselective glycosylation in unperturbed form, it is crucial to use non-participating protecting groups that do not interfere with the structure of the glycosyl cation, such as benzyl ethers. In addition, benzyl groups are resistant to modifications at low pH, eliminating potential artifacts that may arise as a result of acidification during ionization.[36-38] The electron-donating effect of the benzyl group further yields glycosyl donors with high reactivity in glycosylation reactions.[35] Due to these beneficial properties, per-*O*-benzyl-protected thiogalactoside (**GalSTol**) was selected as a suitable candidate to probe the stereoselective glycosylation t in gas-phase reactions.

In the gas-phase experiments, the stereoselectivity of a pure S_N1 mechanism is determined. Glycosylation reactions in solution, on the other hand, usually span a continuum of S_N1/S_N2 mechanisms.[4,10,39-41] To compare, solution-phase glycosylations were performed using the same nucleophiles and carefully controlled conditions. In solution, solvent molecules often provide more than simple solvation. A common hypothesis is that nitrile solvents form quasi-stable α-nitrilium ions that undergo S_N2 displacement to promote the β-product. Similarly, 1,4-dioxane stabilizes the glycosyl cation by forming a β-oxonium ion, which enhances α-selectivity.[42] Because such solvent participations complicate the study of pure S_N1 contributions, we utilized the non-participating solvent dichloromethane to isolate the S_N1 mechanism. Dichloromethane with a low relative

permittivity (εr) of 8.9 also closely resembles the vacuum conditions within the mass spectrometer (εr = 1).

Donor (64.6 mg, 0.1 mmol, 1.0 equiv.) and NIS (34 mg, 64.6 mg, 0.15 mmol, 1.5 equiv.) were added to a dry flask under nitrogen atmosphere. Dry DCM (1 mL) was added and when donor and NIS dissolved, acceptor (1.5 equiv.) was added. TfOH (1.76µL, 0.02 mmol, 0.2 equiv.) was added to the reaction mixture. The mixture was stirred 30 minutes at room temperature (25 ºC), then reaction completion was checked by TLC. The reaction mixture was extracted with EA and distilled water (3 x 30 mL) and concentrated. The residue was purified by HPLC, and the structure was characterized by NMR.

8.1 Characterization of Glycosyl Product in Solution Synthesis

Preparative NP-HPLC for α/β ratio determination

The α/β ratio of the glycosyl product was determined using HPLC. However, if HPLC could not separate the anomers, the ratio was determined from the integral values in the ^{1}H NMR spectrum.

*Methyl 2,3,4,6-Tetra-O-*benzyl-*D-galactopyranoside*

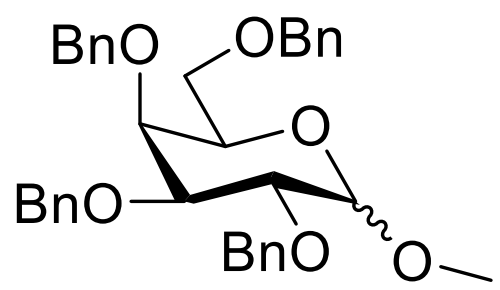

Gal-OMe

Normal Phase HPLC of **Gal-OMe**: The α/β ratio of the product is 44/56. Both the α- and β-anomers were isolated, and their identities were further confirmed via NMR spectroscopy. The detailed NMR assignments for both species are provided below.

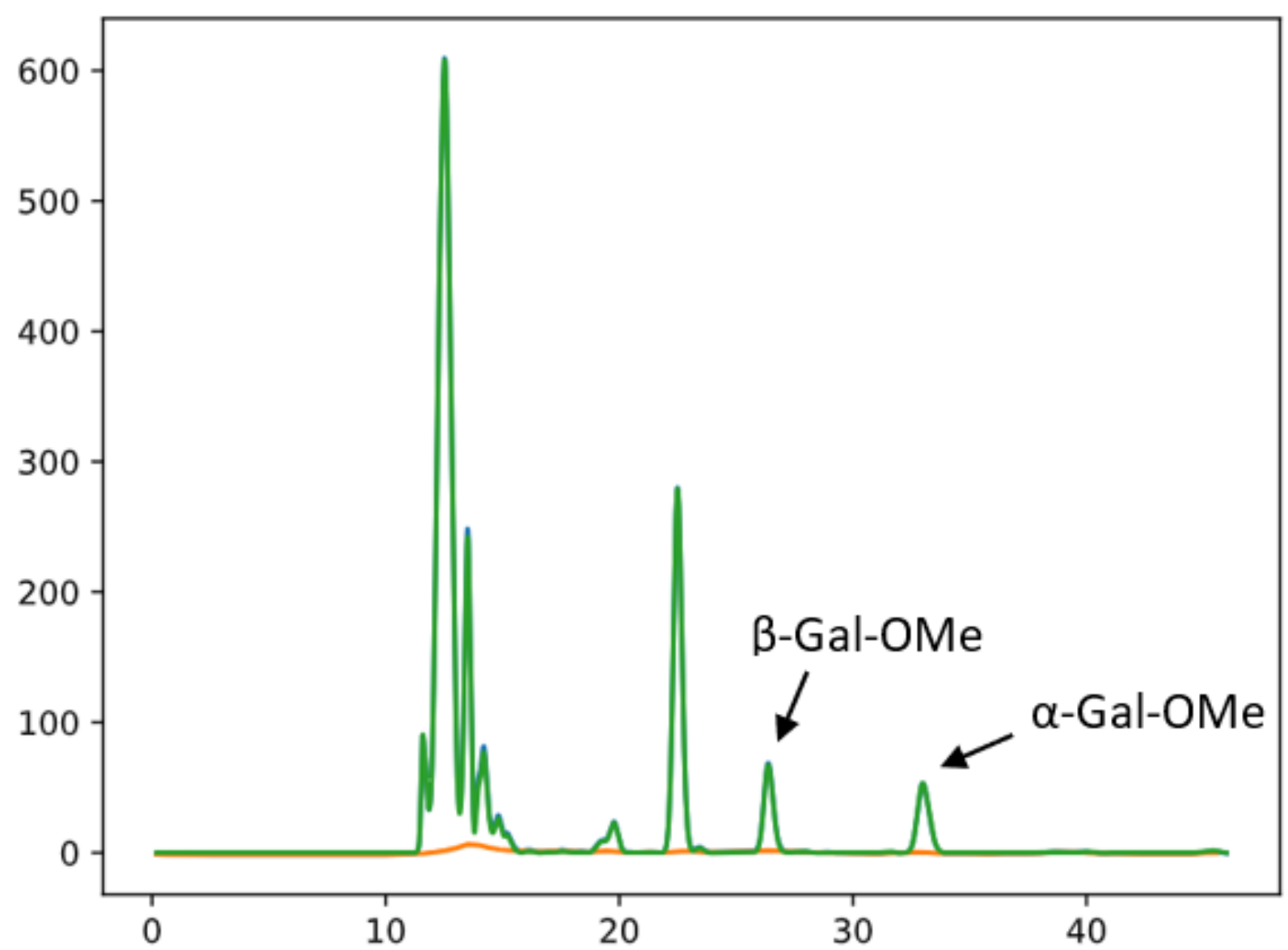

α-anomer of GalOMe:

^{1}H NMR (600 MHz, $CDCl_3$) δ 7.39 – 7.26 (m, 17H), 7.26 – 7.20 (m, 5H), 4.93 (d, *J* = 11.4 Hz, 1H), 4.82 (dd, *J* = 11.9, 9.2 Hz, 2H), 4.72 (d, *J* = 11.7 Hz, 1H), 4.69 – 4.64 (m, 2H), 4.55 (d, *J* = 11.4 Hz, 1H), 4.46 (d, *J* = 11.7 Hz, 1H), 4.38 (d, *J* = 11.8 Hz, 1H), 4.05 – 3.99 (m, 1H), 3.94 – 3.85 (m, 3H), 3.55 – 3.47 (m, 2H), 3.35 (s, 3H).**^{13}C NMR** (151 MHz, $CDCl_3$) δ 138.83, 138.65, 138.52, 137.98, 128.41, 128.38, 128.36, 128.32, 128.25, 128.20, 128.14, 128.09, 127.88, 127.75, 127.69, 127.67, 127.56, 127.48, 98.79, 79.11, 76.44, 75.16, 74.72, 73.56, 73.47, 73.28, 69.22, 69.07, 55.34 ppm. **HRMS (ESI)** calcd for $C_{35}H_{38}O_6Na$ $[M + Na]^+$ 577.2566, found 577.2535.

^{1}H NMR (600 MHz, $CDCl_3$) of **α-anomer GalOMe**:

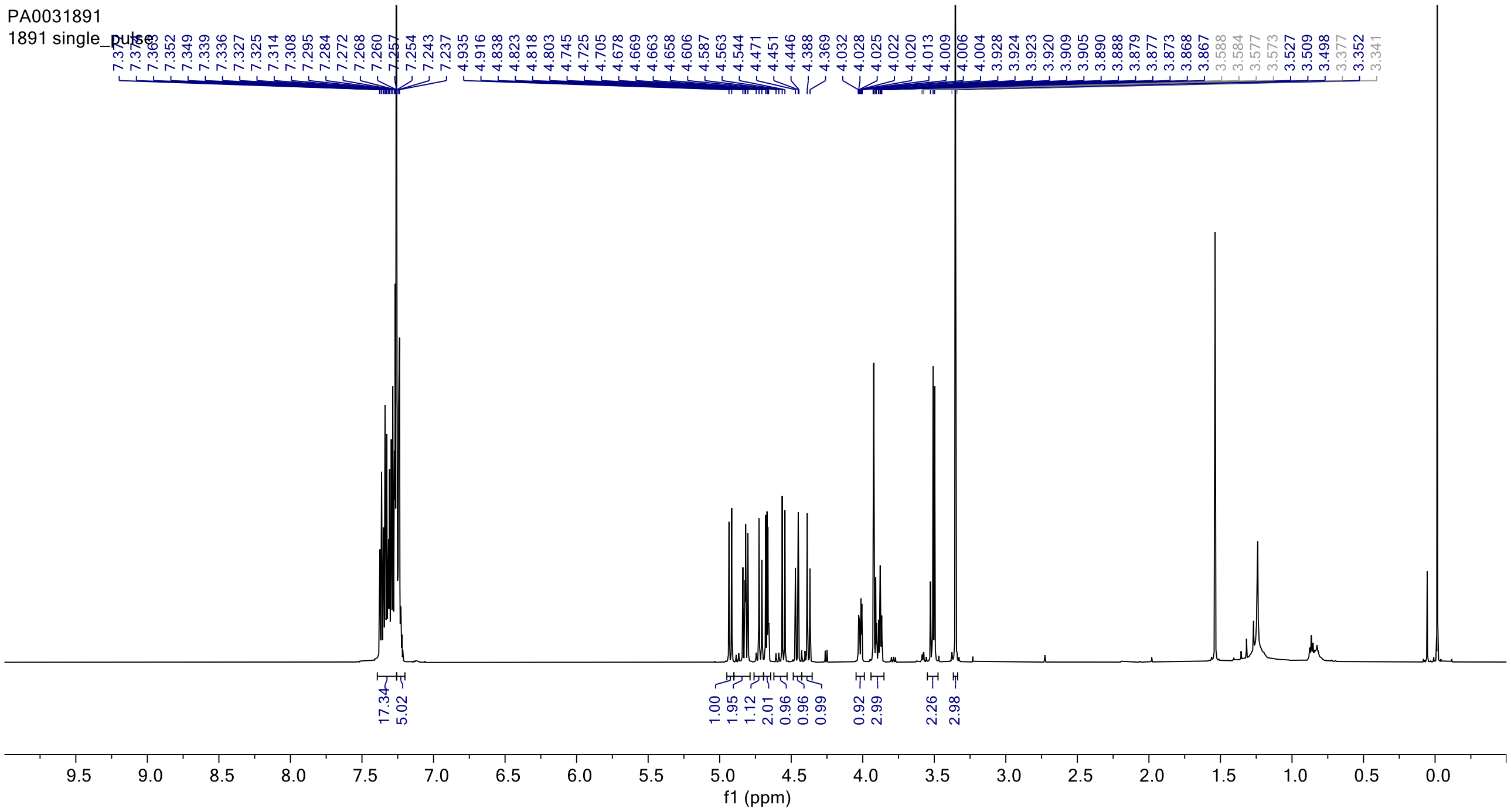

2D-HSQC (^{1}H, ^{13}C) (151 MHz, $CDCl_3$) of **α-anomer GalOMe**:

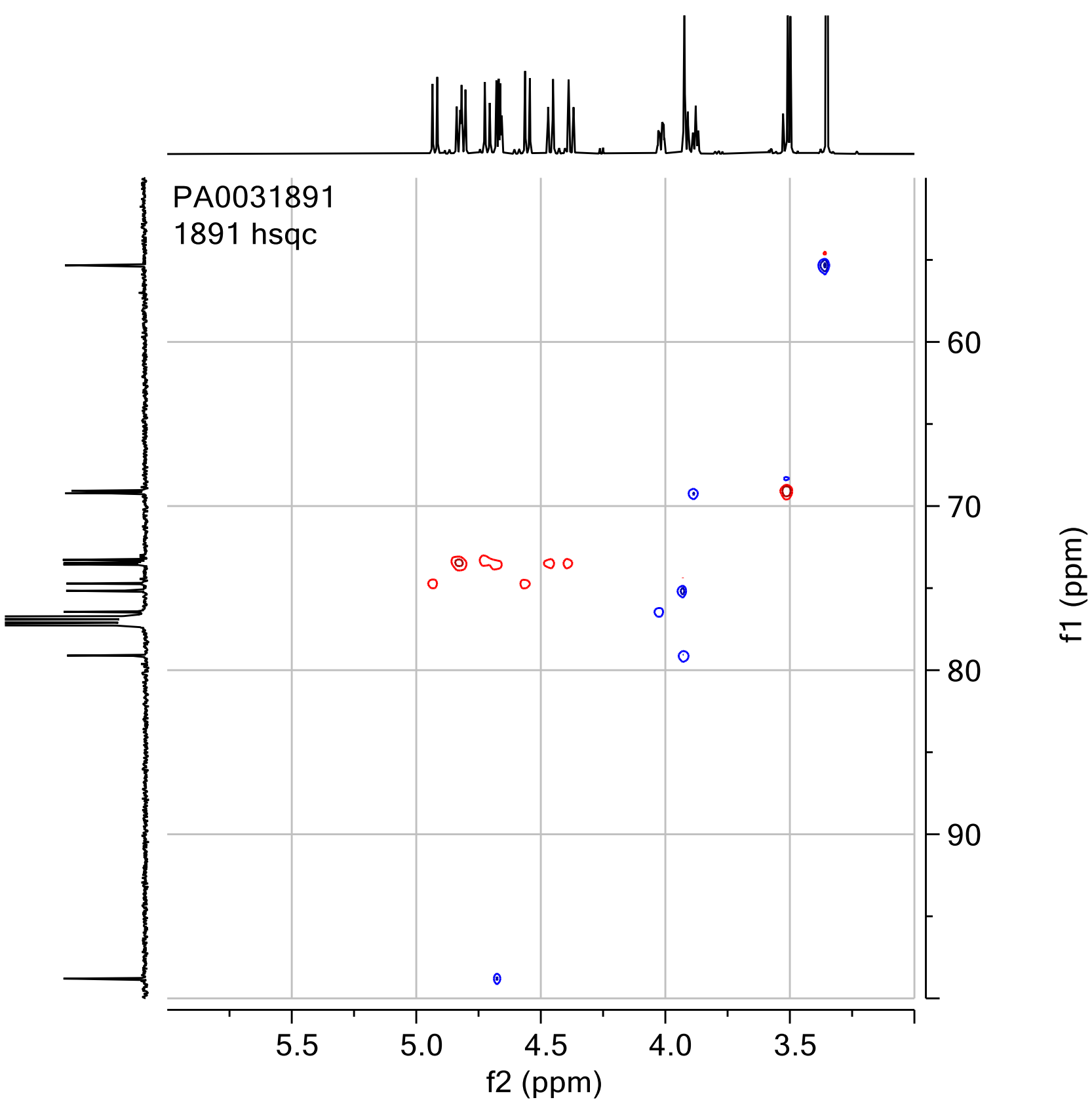

^{13}C NMR (151 MHz, $CDCl_3$) of **α-anomer GalOMe**:

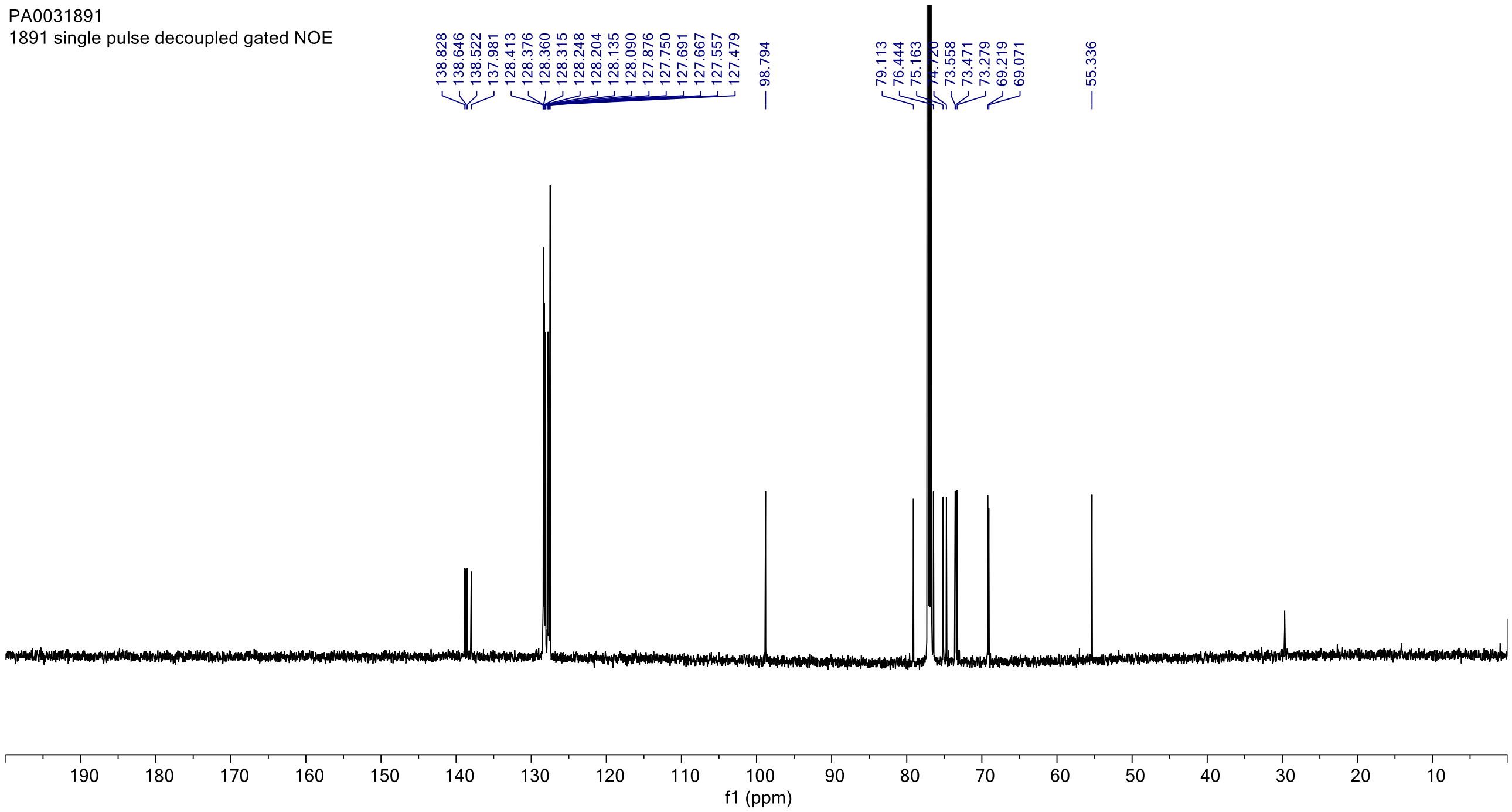

DEPT135 NMR (151 MHz, $CDCl_3$) of **α-anomer GalOMe**:

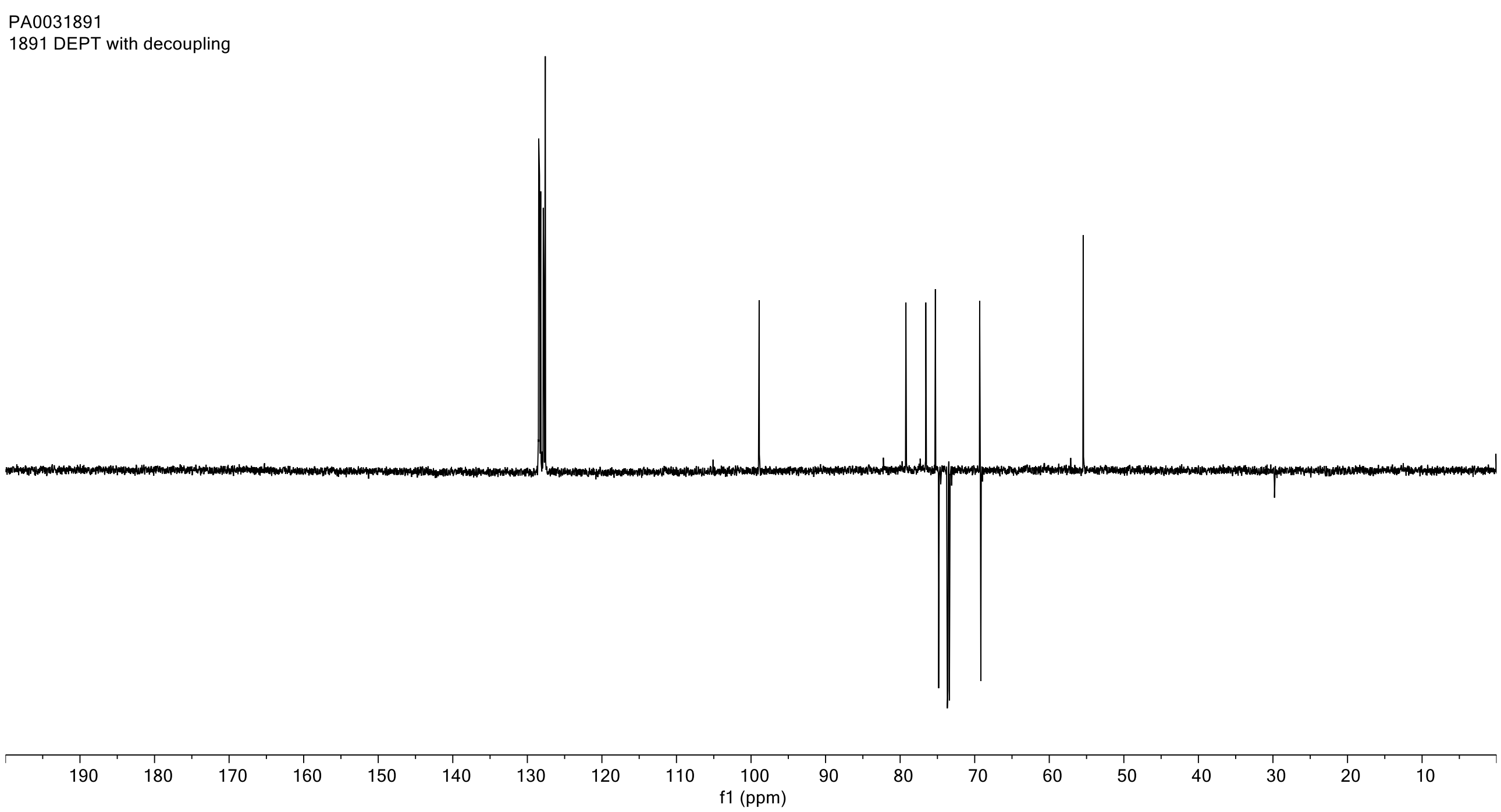

ESI-MS of **α-anomer GalOMe**:

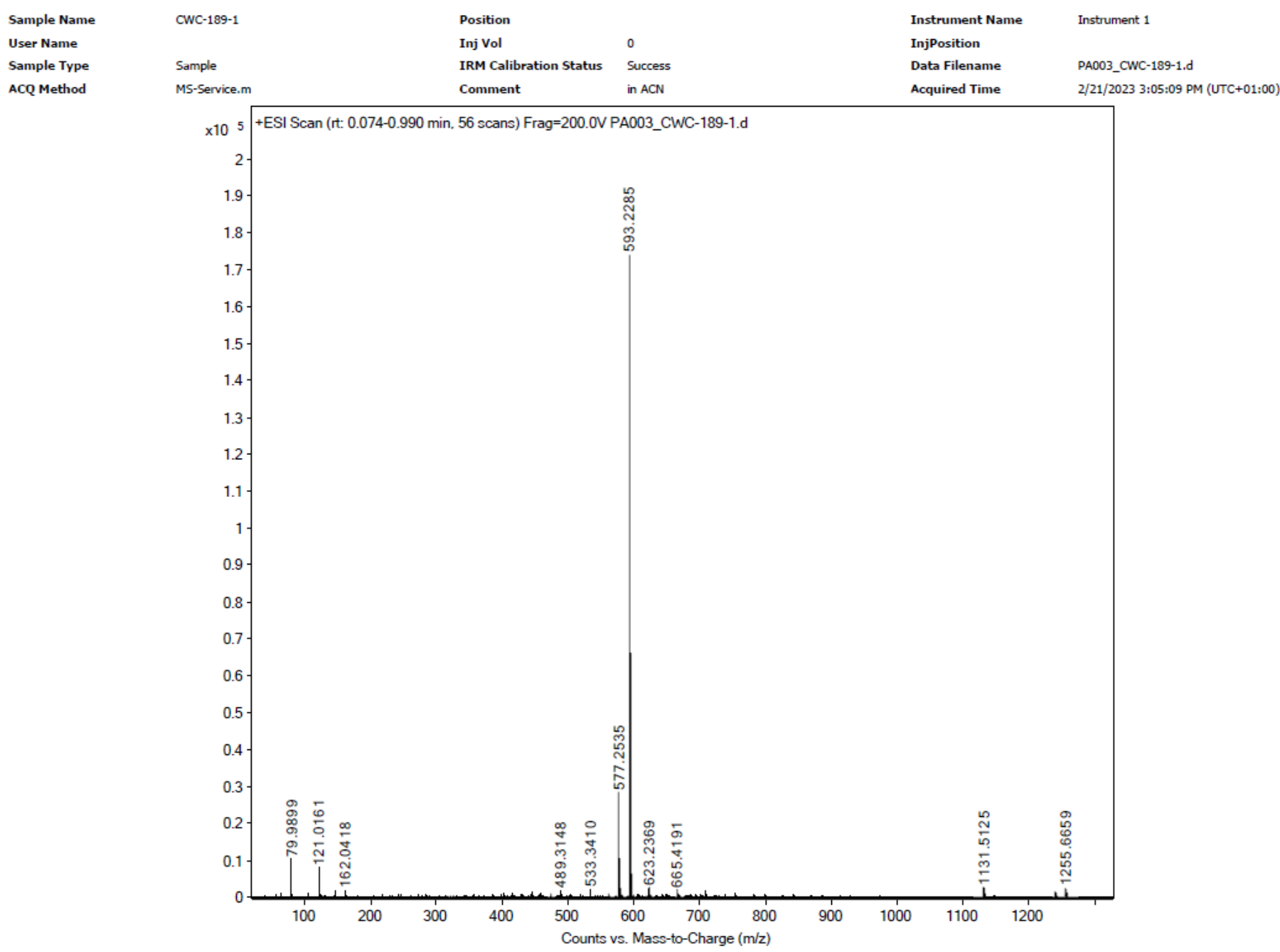

β-anomer of GalOMe:

^{1}H NMR (600 MHz, $CDCl_3$) δ 7.36 – 7.21 (m, 22H), 4.90 (dd, *J* = 27.1, 11.3 Hz, 2H), 4.76 – 4.66 (m, 3H), 4.59 (d, *J* = 11.7 Hz, 1H), 4.46 – 4.37 (m, 2H), 3.87 (d, *J* = 2.9 Hz, 1H), 3.78 (dd, *J* = 9.8, 7.7 Hz, 1H), 3.58 (dd, *J* = 6.3, 2.4 Hz, 2H), 3.54 – 3.47 (m, 5H). **^{13}C NMR** (151 MHz, $CDCl_3$) δ 138.81, 138.63, 138.49, 137.90, 128.41, 128.33, 128.25, 128.13, 128.09, 127.87, 127.76, 127.53, 127.51, 127.48, 104.97, 82.13, 79.61, 75.12, 74.43, 73.53, 73.42, 73.35, 72.98, 68.84, 57.00 ppm. **HRMS (ESI)** calcd for $C_{35}H_{38}O_6Na$ $[M + Na]^+$ 577.2566, found 577.2532.

^{1}H NMR (600 MHz, $CDCl_3$) of **β-anomer GalOMe**:

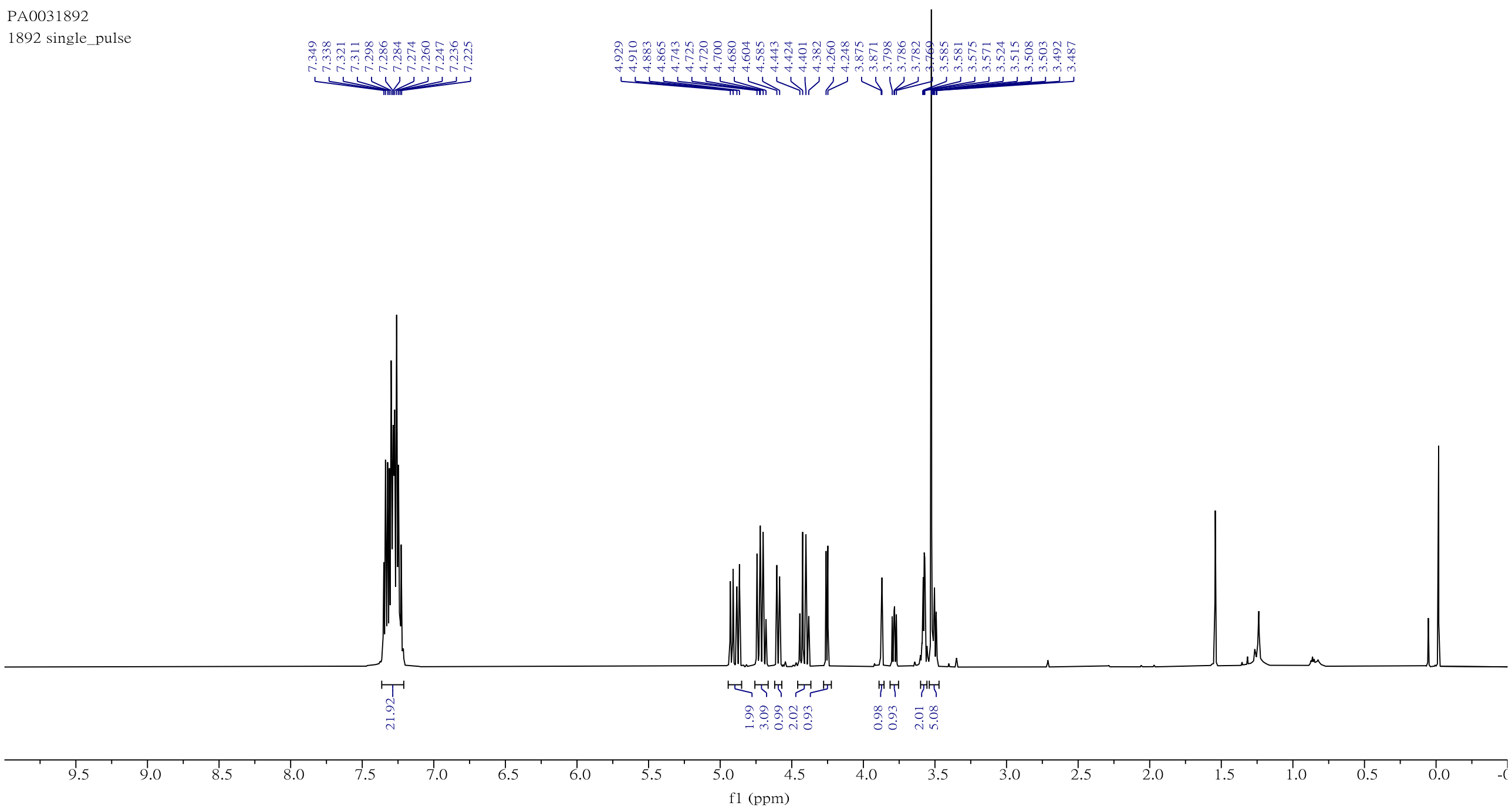

2D-HSQC (^{1}H, ^{13}C) (151 MHz, $CDCl_3$) of **β-anomer GalOMe**:

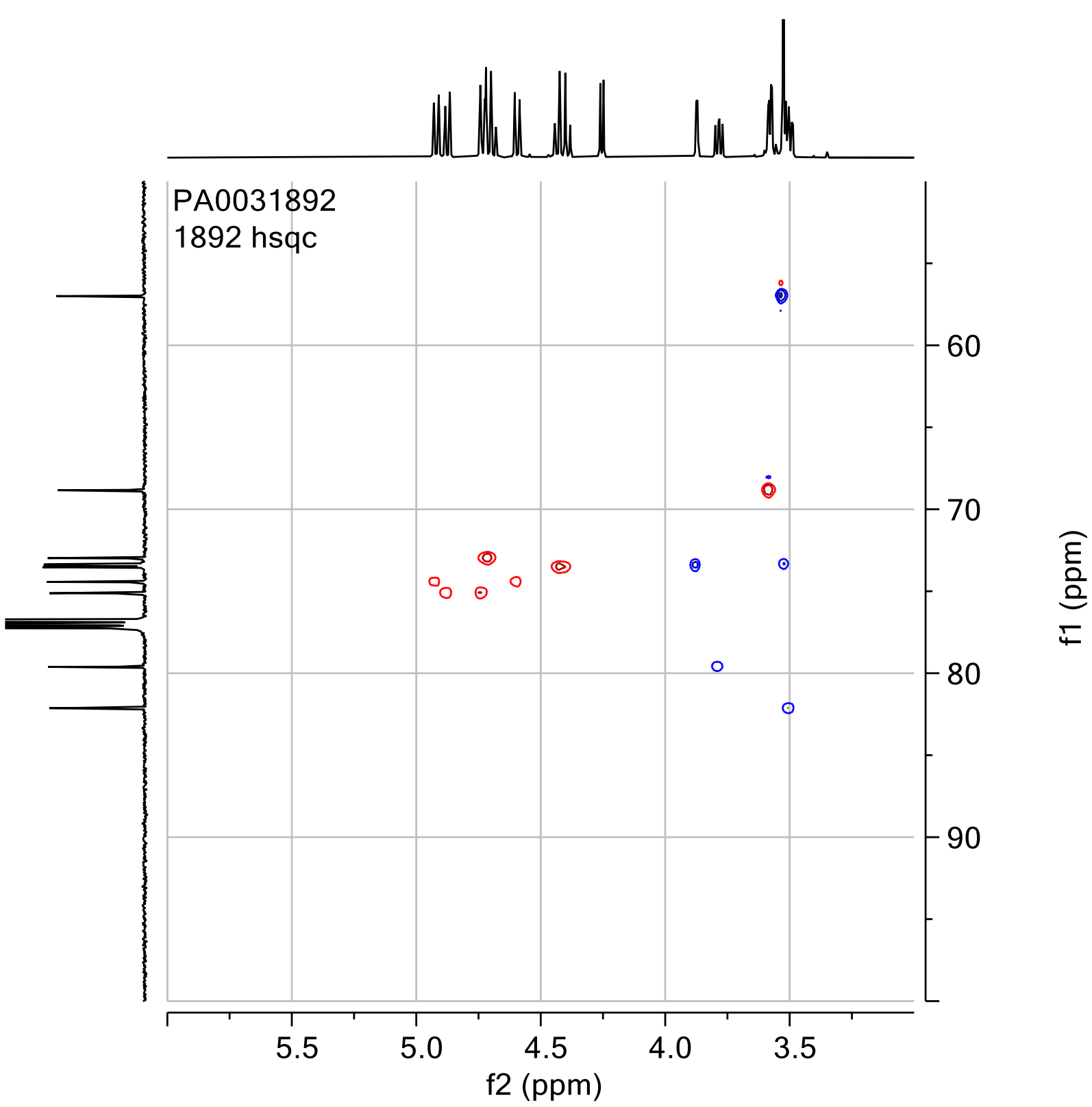

^{13}C NMR (151 MHz, $CDCl_3$) of **β-anomer GalOMe**:

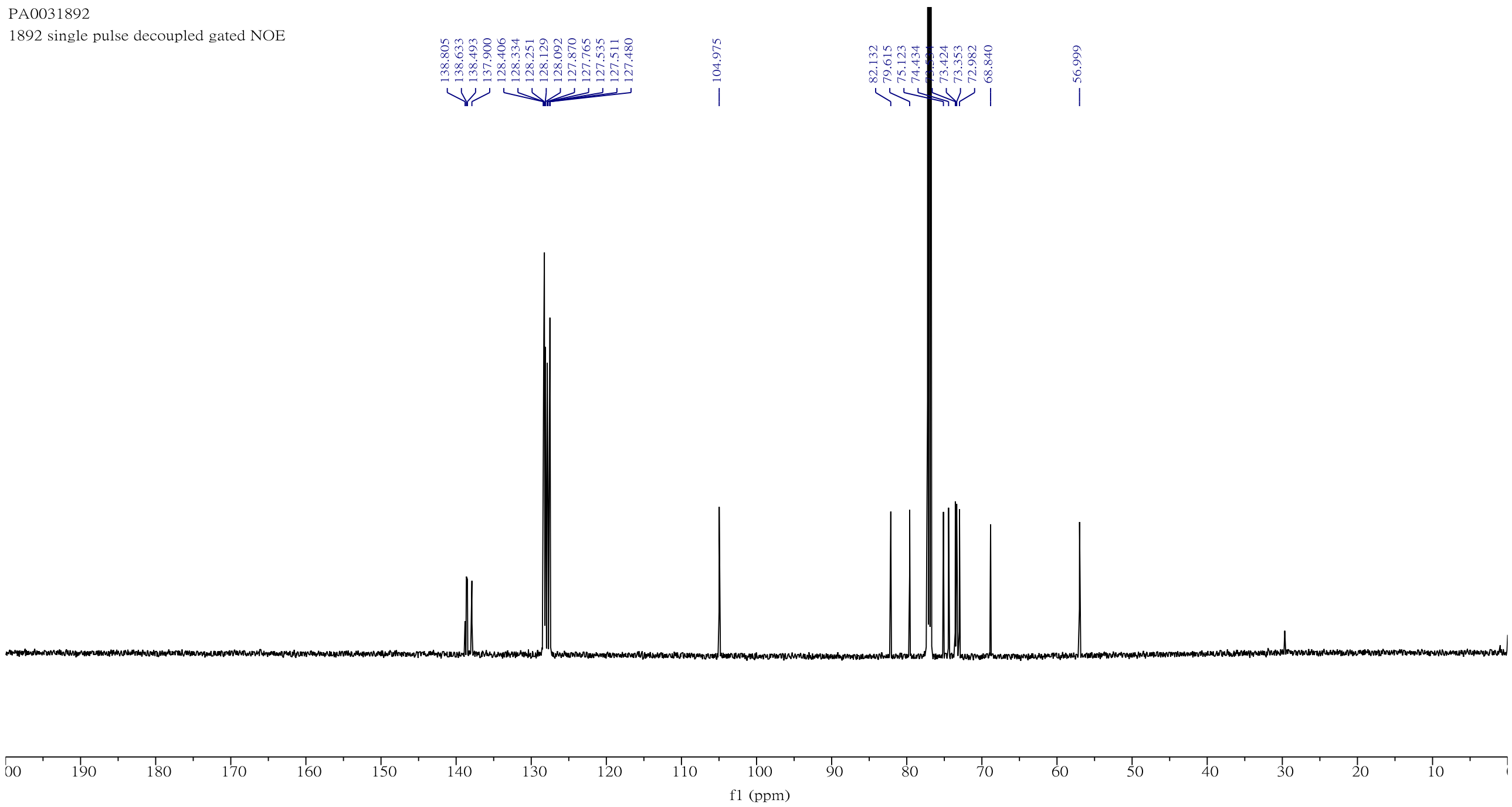

DEPT135 NMR (151 MHz, $CDCl_3$) of **β-anomer GalOMe**:

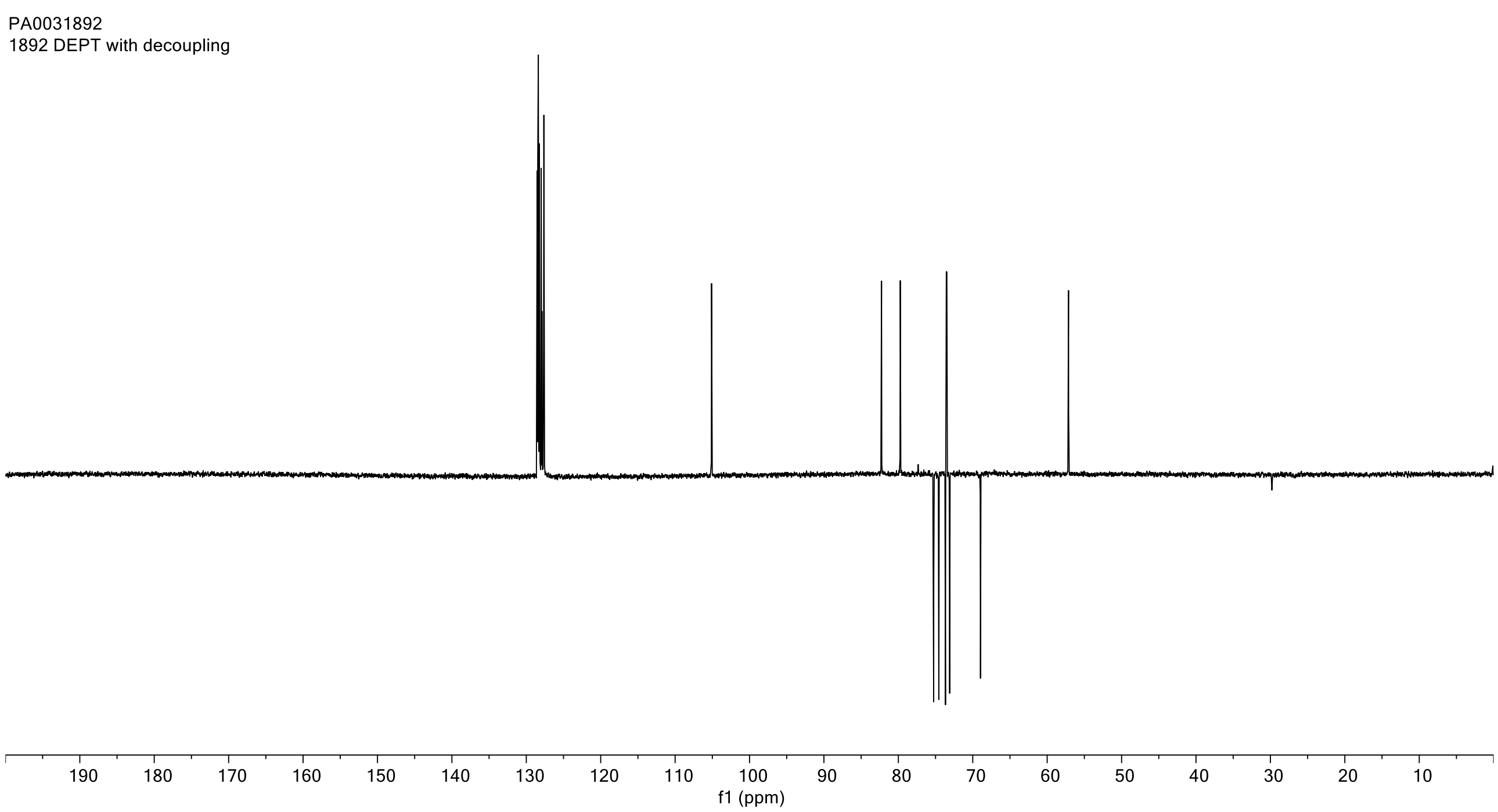

ESI-MS of **β-anomer GalOMe**:

**Sample Name**	CWC-189-2	**Position**		**Instrument Name**	Instrument 1
**User Name**		**Inj Vol**	0	**InjPosition**	
**Sample Type**	Sample	**IRM Calibration Status**	Success	**Data Filename**	PA003_CWC-189-2.d
**ACQ Method**	MS-Service.m	**Comment**	in ACN	**Acquired Time**	2/21/2023 3:09:38 PM (UTC+01:00)

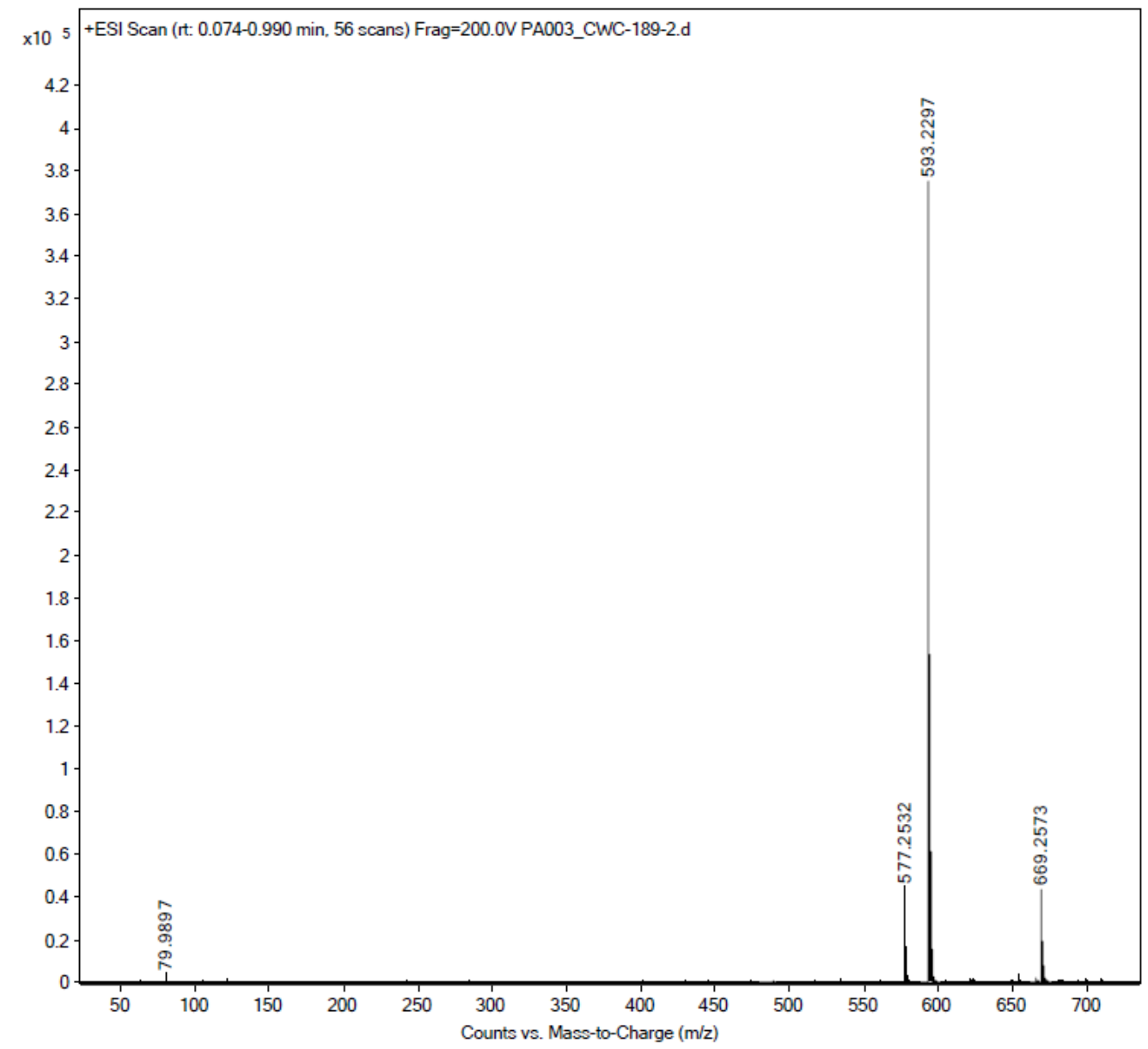

Ethyl 2,3,4,6-Tetra-O-benzyl-D-galactopyranoside

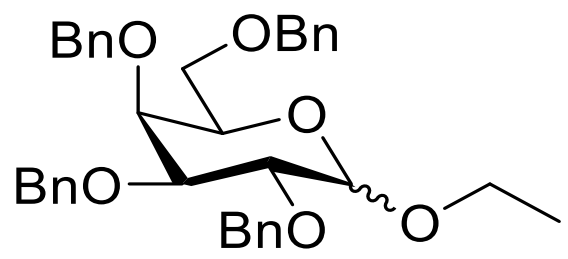

Gal-OEt

Normal Phase HPLC of **Gal-OEt**: The α/β anomers could not be separated by HPLC; therefore, their ratio was determined by NMR. The products were isolated, and their α- and β-anomers were further confirmed via NMR spectroscopy. The detailed NMR assignments for both species are provided below.

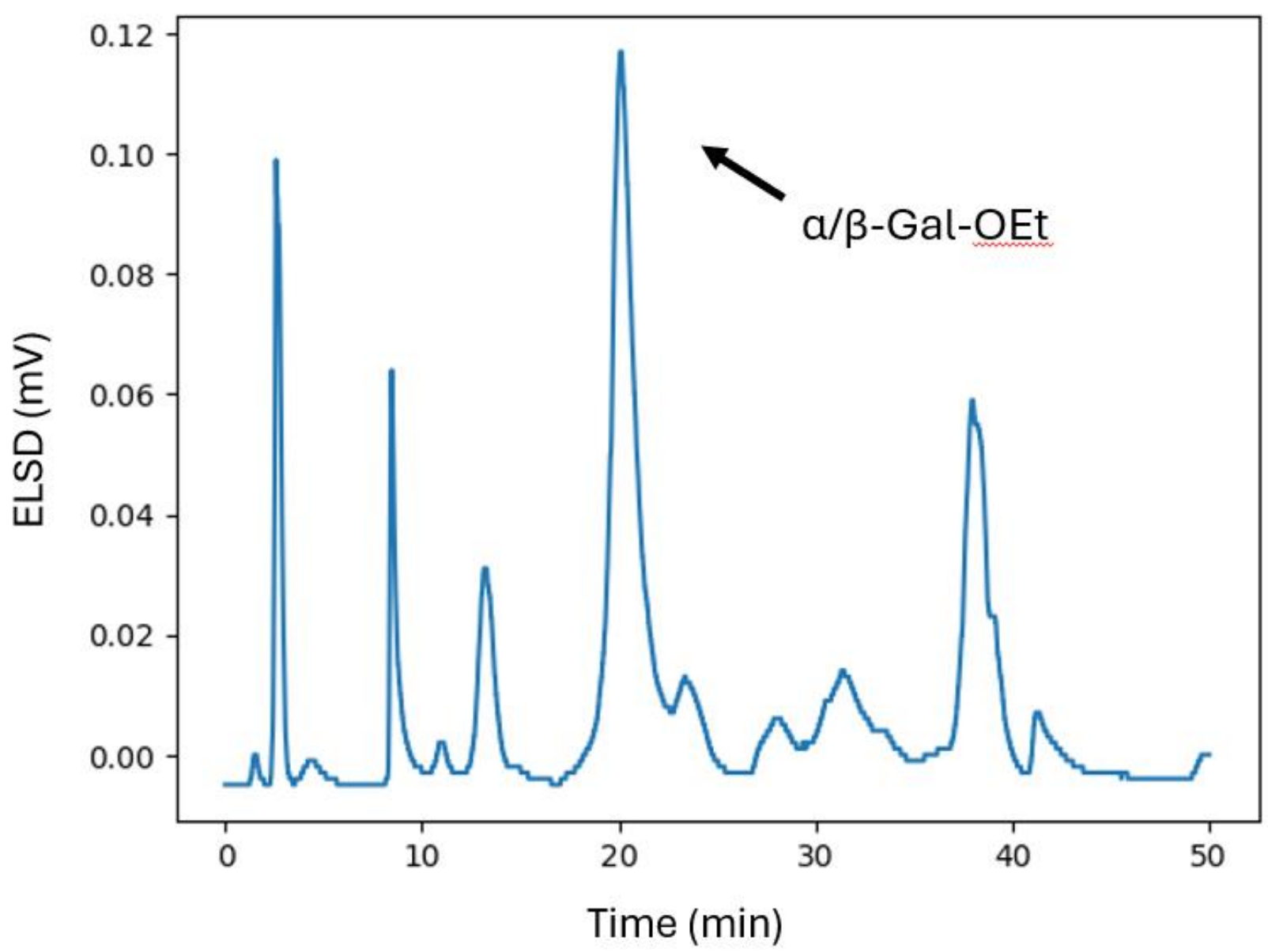

^{1}H NMR (700 MHz, $CDCl_3$) of **GalOEt**, region shown: 1.5 - 0.9 ppm. The α/β ratio of the **GalOEt** was determined from the integral values of the CH_3 protons on the reducing end (O-ethyl group) in the ^{1}H NMR spectrum. The CH_3 signal of the α-anomer appears at δ 1.29 (t, *J* = 7.1 Hz, 3H), while the CH_3 signal of the β-anomer appears at δ 1.27 (t, *J* = 7.1 Hz, 3H). The integral ratio of 1.35:1 corresponds to 57% α-anomer.

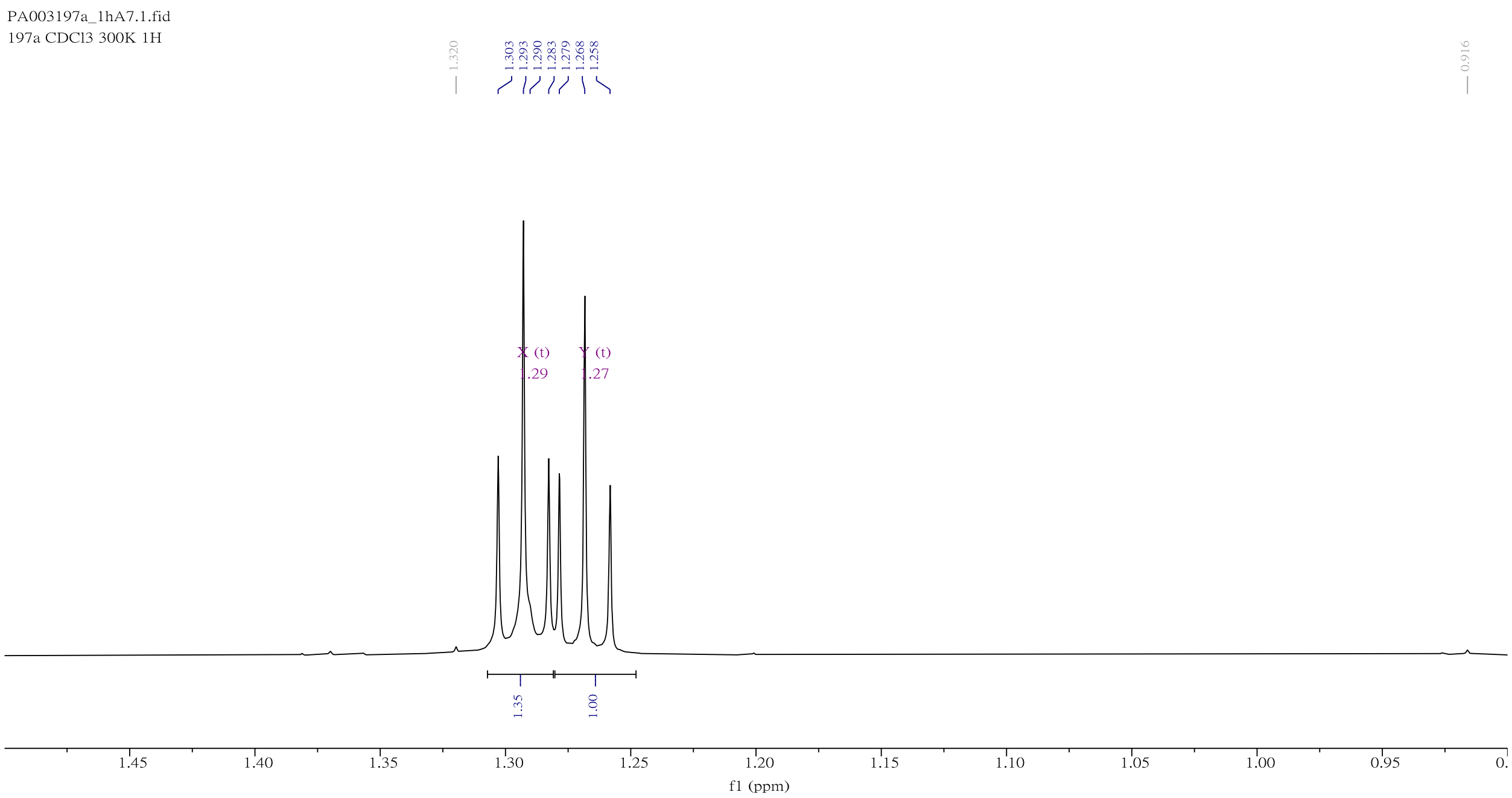

^{1}H NMR (700 MHz, $CDCl_3$) δ 7.43 – 7.28 (m, 34H), 7.28 – 7.26 (m, 1H), 5.00 – 4.95 (m, 3H), 4.90 – 4.87 (m, 1H), 4.85 (dd, *J* = 7.9, 4.2 Hz, 2H), 4.80 (d, *J* = 3.0 Hz, 1H), 4.79 – 4.77 (m, 2H), 4.77 – 4.72 (m, 1H), 4.71 (d, *J* = 12.1 Hz, 1H), 4.65 (d, *J* = 11.6 Hz, 1H), 4.60 (d, *J* = 11.5 Hz, 1H), 4.52 – 4.48 (m, 1H), 4.47 (s, 1H), 4.44 (d, *J* = 6.4 Hz, 1H), 4.43 (d, *J* = 6.3 Hz, 1H), 4.39 (d, *J* = 7.7 Hz, 1H), 4.06 (dd, *J* = 9.5, 3.7 Hz, 1H), 4.04 – 3.96 (m, 3H), 3.92 (dd, *J* = 2.9, 1.1 Hz, 1H), 3.84 (dd, *J* = 9.8, 7.7 Hz, 1H), 3.73 (dq, *J* = 10.1, 7.1 Hz, 1H), 3.65 – 3.59 (m, 3H), 3.59 – 3.52 (m, 4H), 1.29 (t, *J* = 7.1 Hz, 3H), 1.27 (t, *J* = 7.1 Hz, 3H). **^{13}C NMR** (176 MHz, CDCl3) δ 138.97, 138.89, 138.76, 138.72, 138.61, 138.05, 137.98, 128.43, 128.40, 128.36, 128.32, 128.28, 128.25, 128.21, 128.15, 128.04, 127.90, 127.78, 127.71, 127.63, 127.54, 127.50, 127.47, 127.45, 103.82, 97.18, 82.24, 79.66, 79.22, 77.22, 77.04, 76.86, 76.60, 75.24, 75.20, 74.78, 74.48, 73.56, 73.50, 73.39, 73.29, 73.06, 69.23, 69.08, 69.00, 68.95, 65.48, 63.34, 15.32, 15.01. **HRMS (ESI)** calcd for $C_{36}H_{46}O_6Na$ $[M + Na]^+$ 691.2727, found 691.2782.

^{1}H NMR (700 MHz, $CDCl_3$) of **GalOEt**:

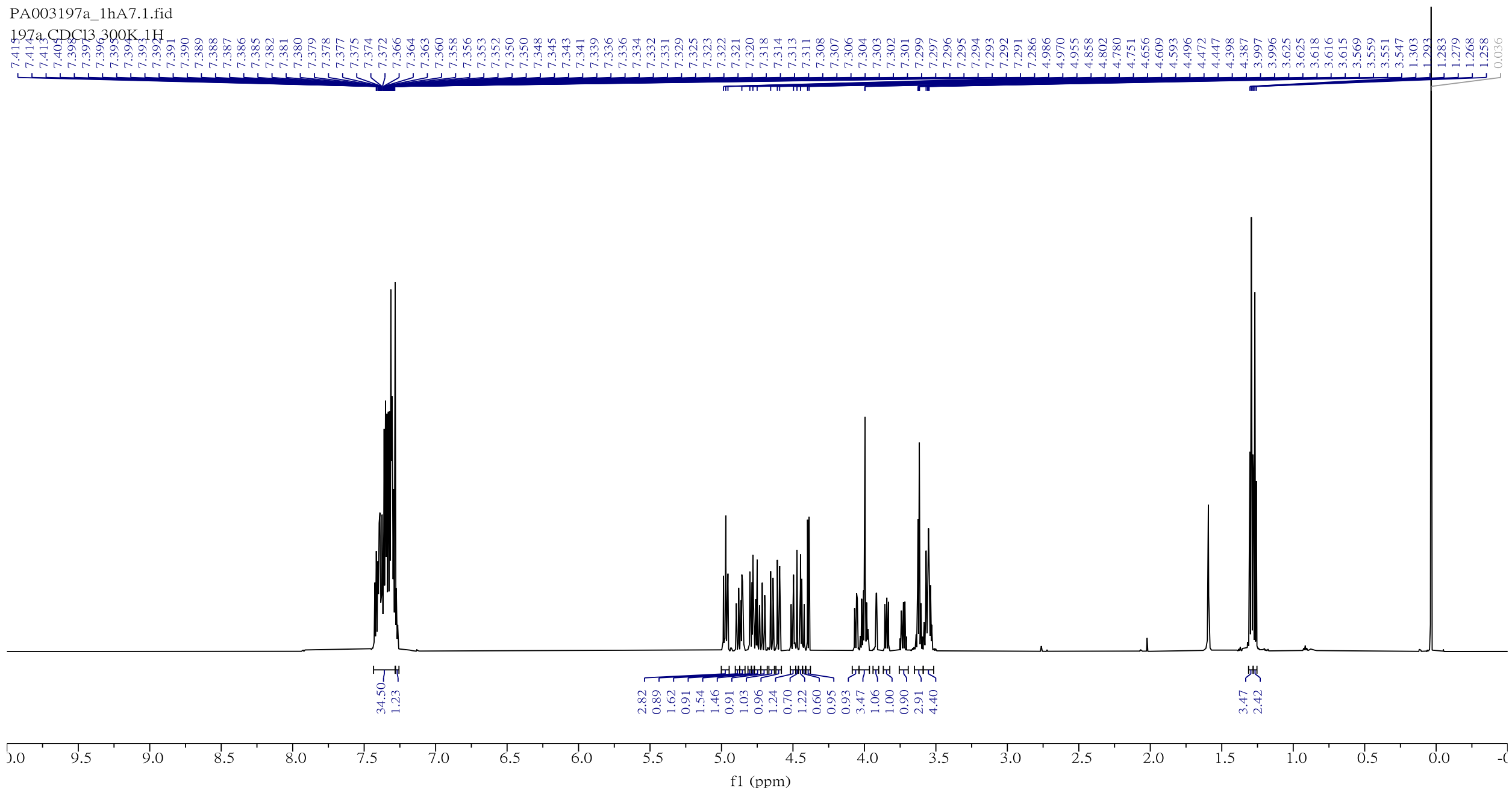

^{13}C NMR (176 MHz, $CDCl_3$) of **GalOEt**:

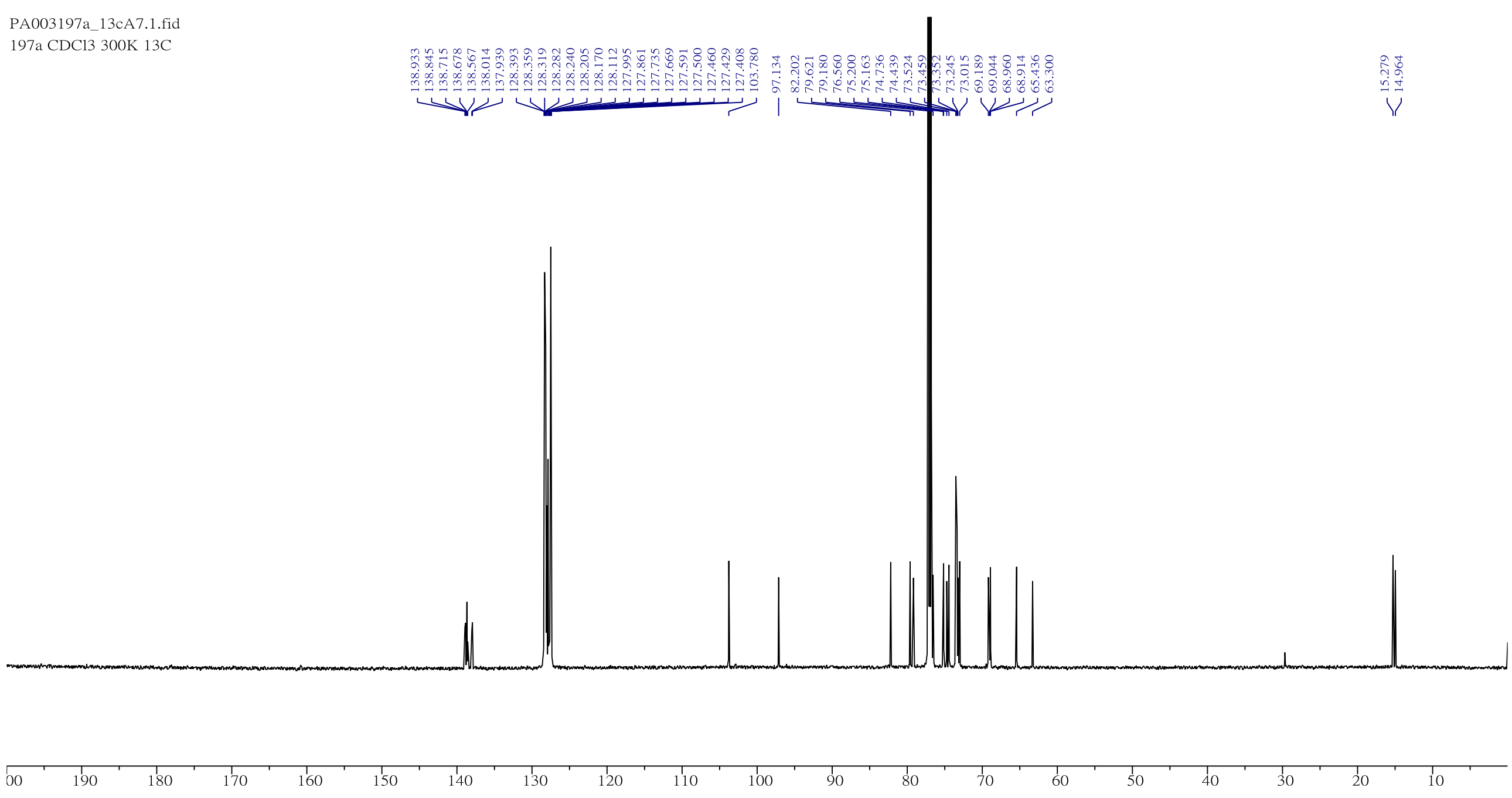

DEPT135 NMR (151 MHz, $CDCl_3$) of **GalOEt**:

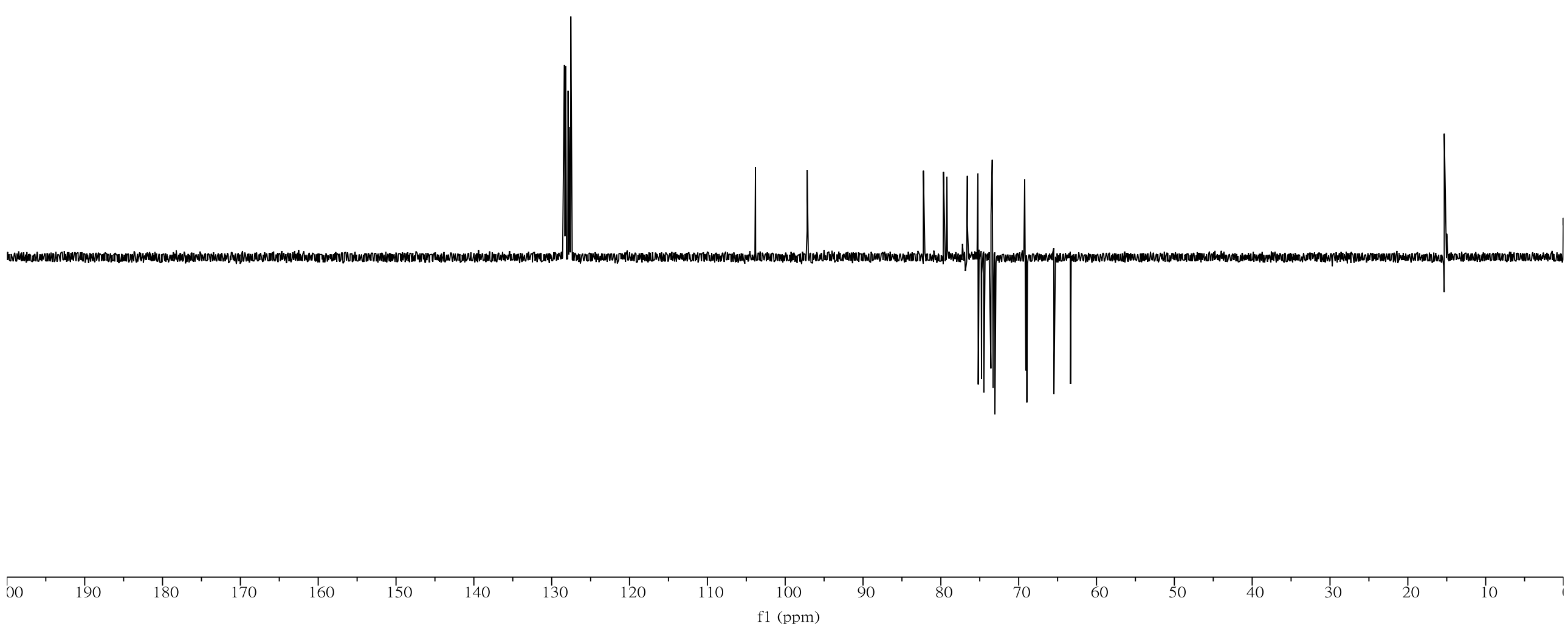

2D-HSQC (^{1}H, ^{13}C) (176 MHz, $CDCl_3$) of **GalOEt**:

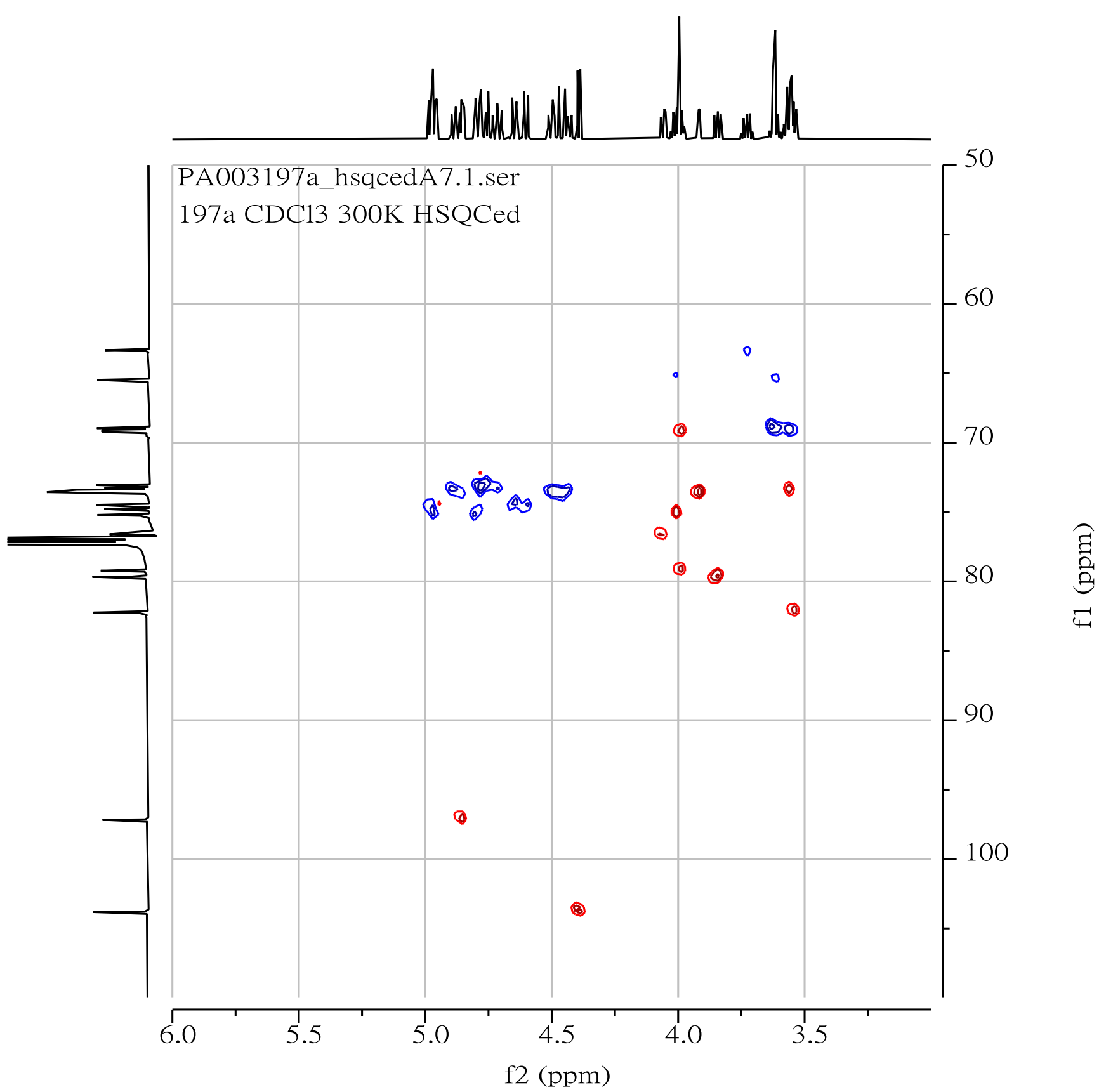

ESI-MS of **GalOEt**:

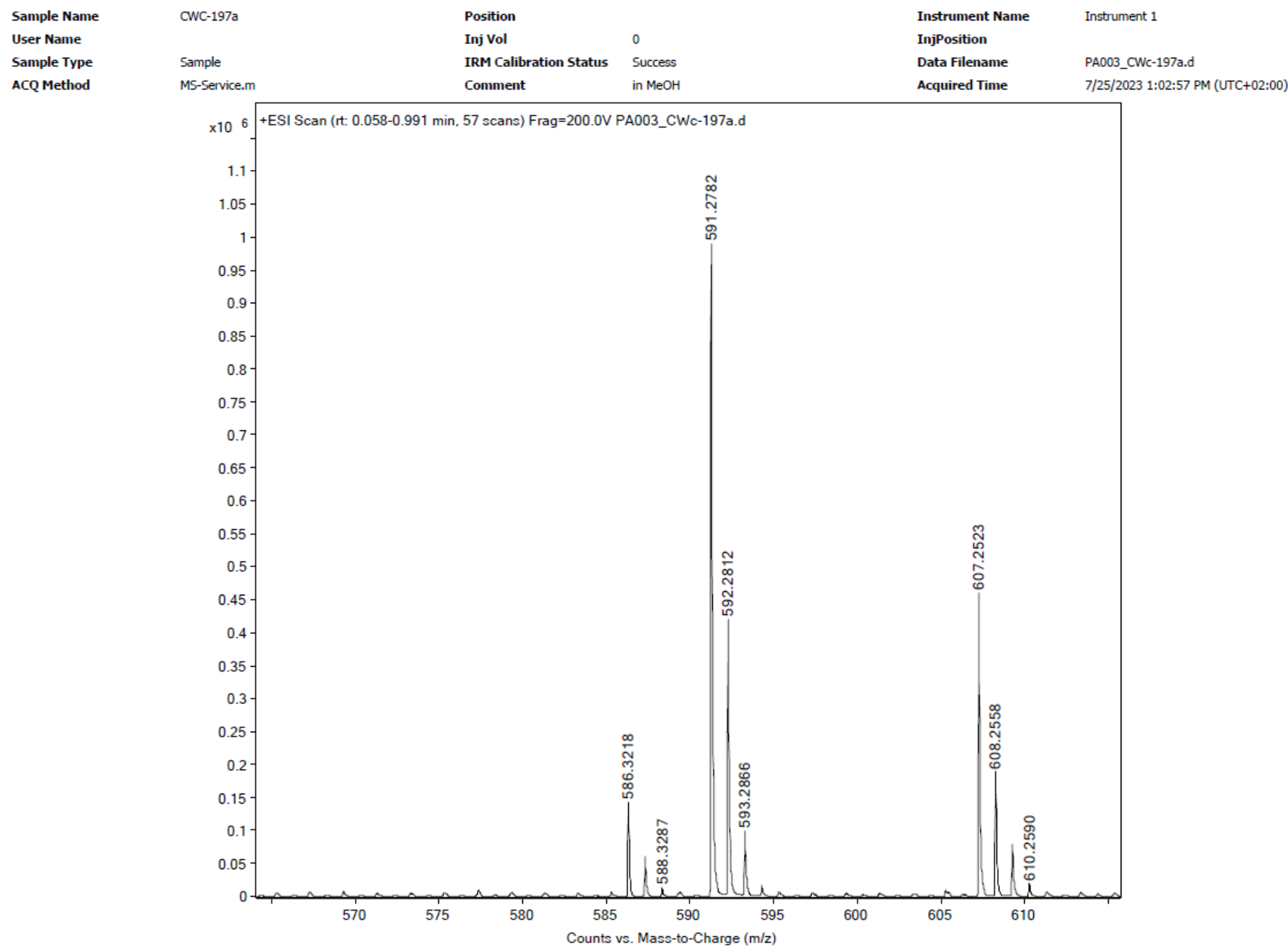

iso-Propyl 2,3,4,6-Tetra-O-benzyl-D-galactopyranoside

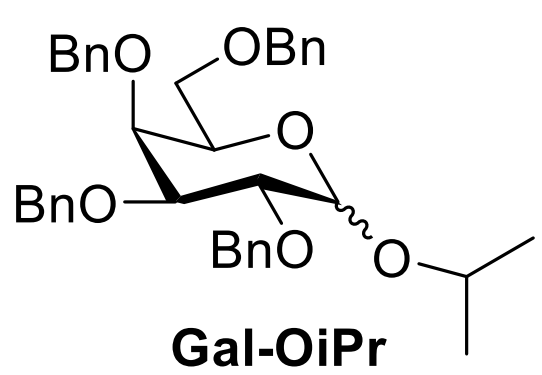

Gal-OiPr

Normal Phase HPLC of **Gal-OiPr**: The α/β ratio of the product is 57/43. Both the α- and β-anomers were isolated, and their identities were further confirmed via NMR spectroscopy. The detailed NMR assignments for both species are provided below.

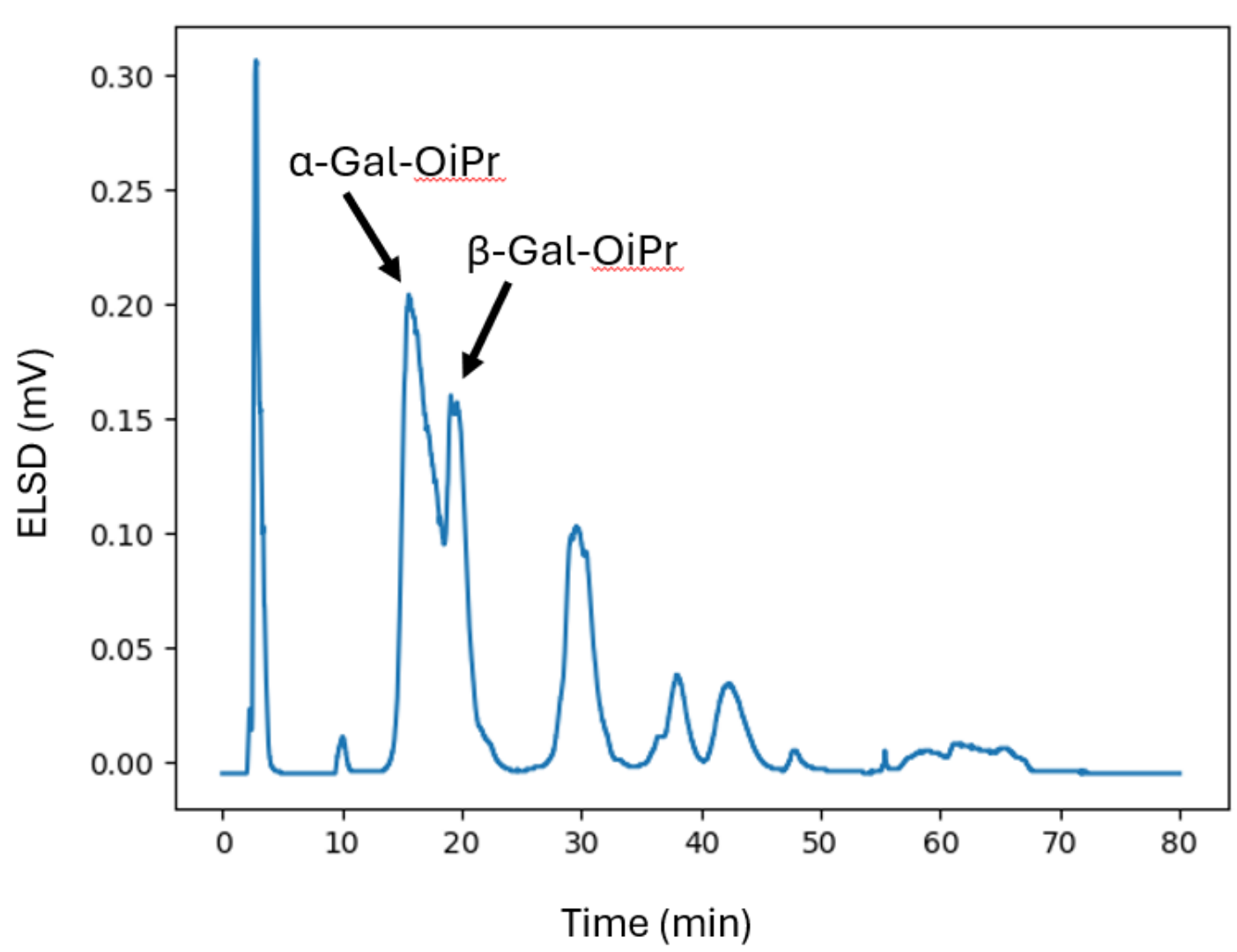

α-anomer of GalOPr:

^{1}H NMR (600 MHz, $CDCl_3$) δ 7.41 – 7.27 (m, 19H), 7.27 – 7.24 (m, 3H), 4.98 – 4.92 (m, 2H), 4.86 (d, *J* = 11.6 Hz, 1H), 4.81 (d, *J* = 12.0 Hz, 1H), 4.74 (d, *J* = 11.6 Hz, 1H), 4.68 (d, *J* = 12.0 Hz, 1H), 4.58 (d, *J* = 11.5 Hz, 1H), 4.48 (d, *J* = 11.8 Hz, 1H), 4.41 (d, *J* = 11.8 Hz, 1H), 4.05 – 4.01 (m, 2H), 3.99 (dd, *J* = 3.0, 1.2 Hz, 1H), 3.98 – 3.86 (m, 2H), 3.58 – 3.49 (m, 2H), 1.23 (d, *J* = 6.3 Hz, 3H), 1.18 (d, *J* = 6.1 Hz, 3H). **^{13}C NMR** ^{13}C NMR (151 MHz, $CDCl_3$) δ 138.98, 138.76, 138.70, 138.06, 128.35, 128.30, 128.28, 128.20, 128.17, 127.98, 127.68, 127.65, 127.57, 127.48, 127.41, 127.38, 95.50, 79.23, 76.50, 75.16, 74.72, 73.41, 73.22, 73.17, 69.12, 69.10, 69.04, 23.18, 21.24 ppm. **HRMS (ESI)** calcd for $C_{37}H_{42}O_6Na$ $[M + Na]^+$ 605.2879, found 605.2970.

^{1}H NMR (600 MHz, $CDCl_3$) of **α-anomer GalOPr**:

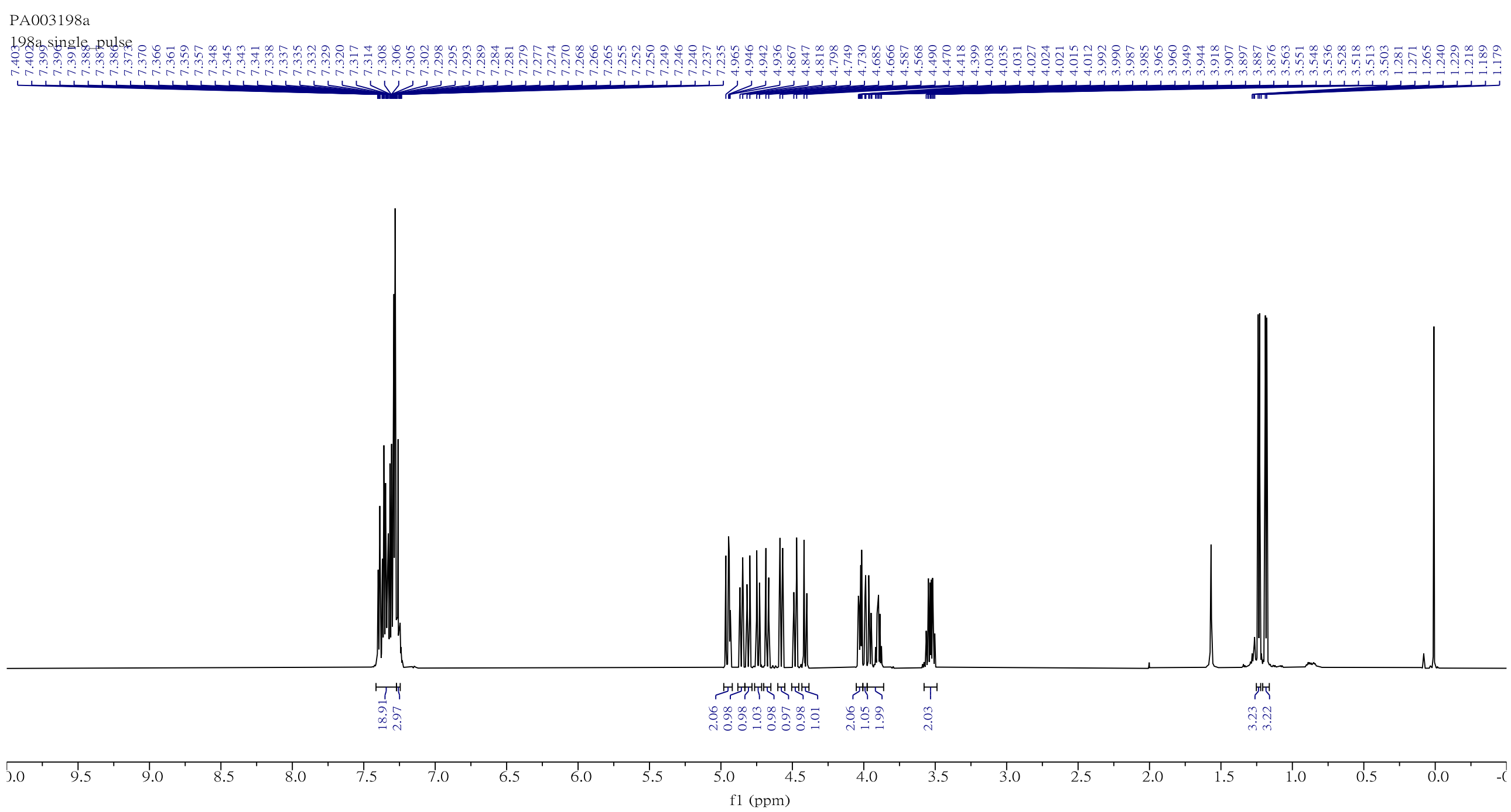

2D-HSQC (^{1}H, ^{13}C) (151 MHz, $CDCl_3$) of **α-anomer GalOPr**:

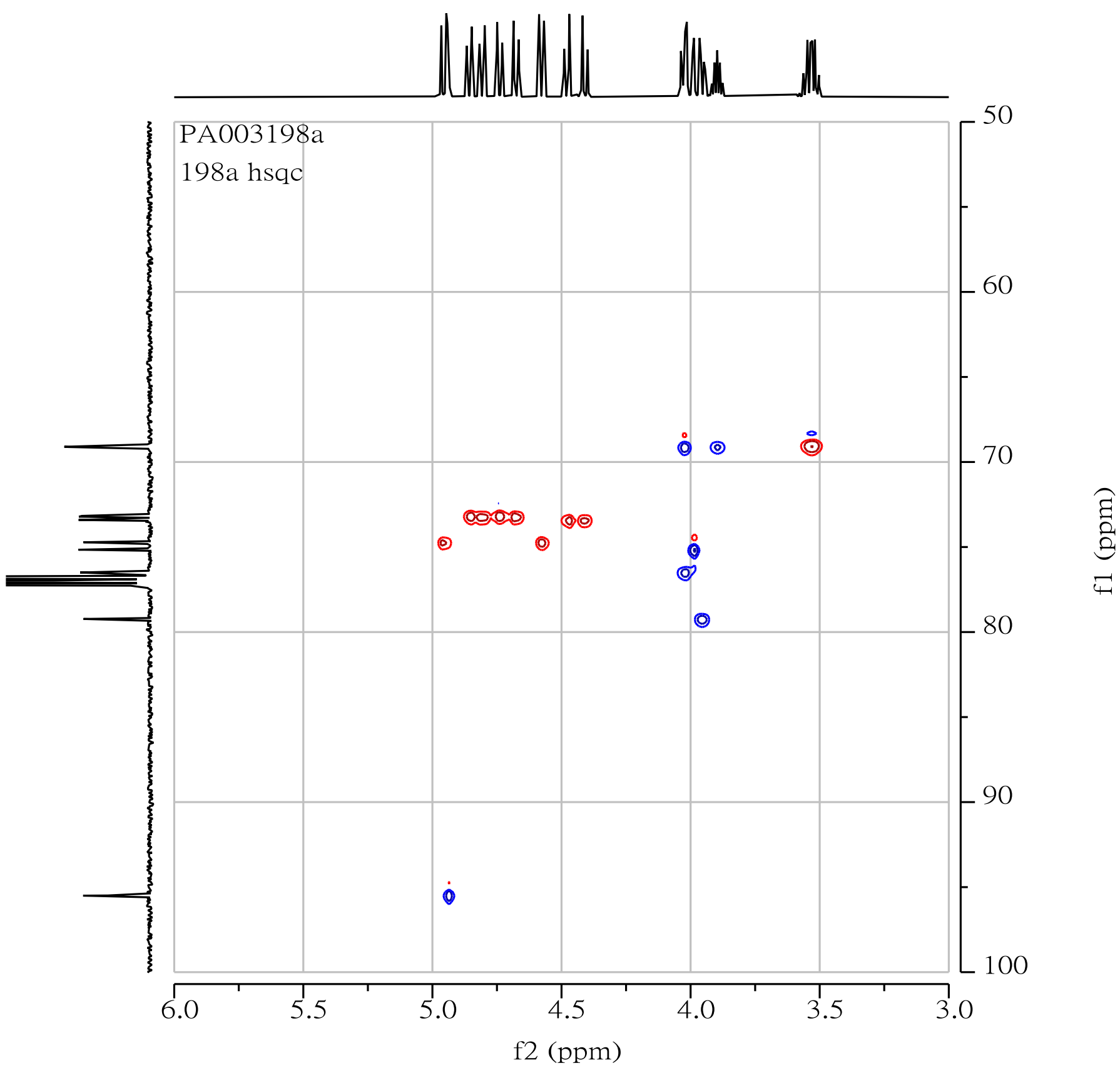

^{13}C NMR (151 MHz, $CDCl_3$) of **α-anomer GalOPr**:

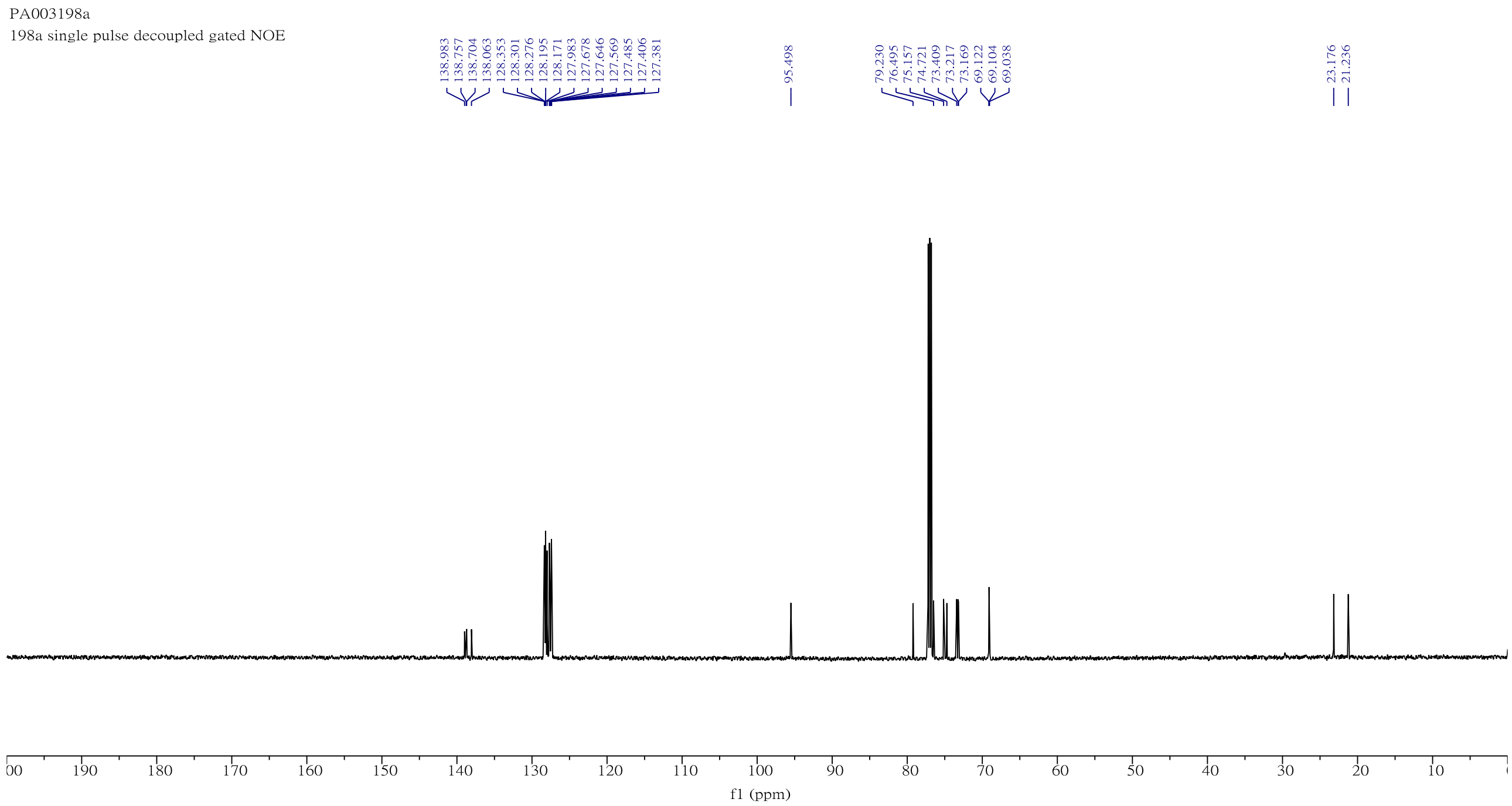

DEPT135 NMR (151 MHz, $CDCl_3$) of **α-anomer GalOPr**:

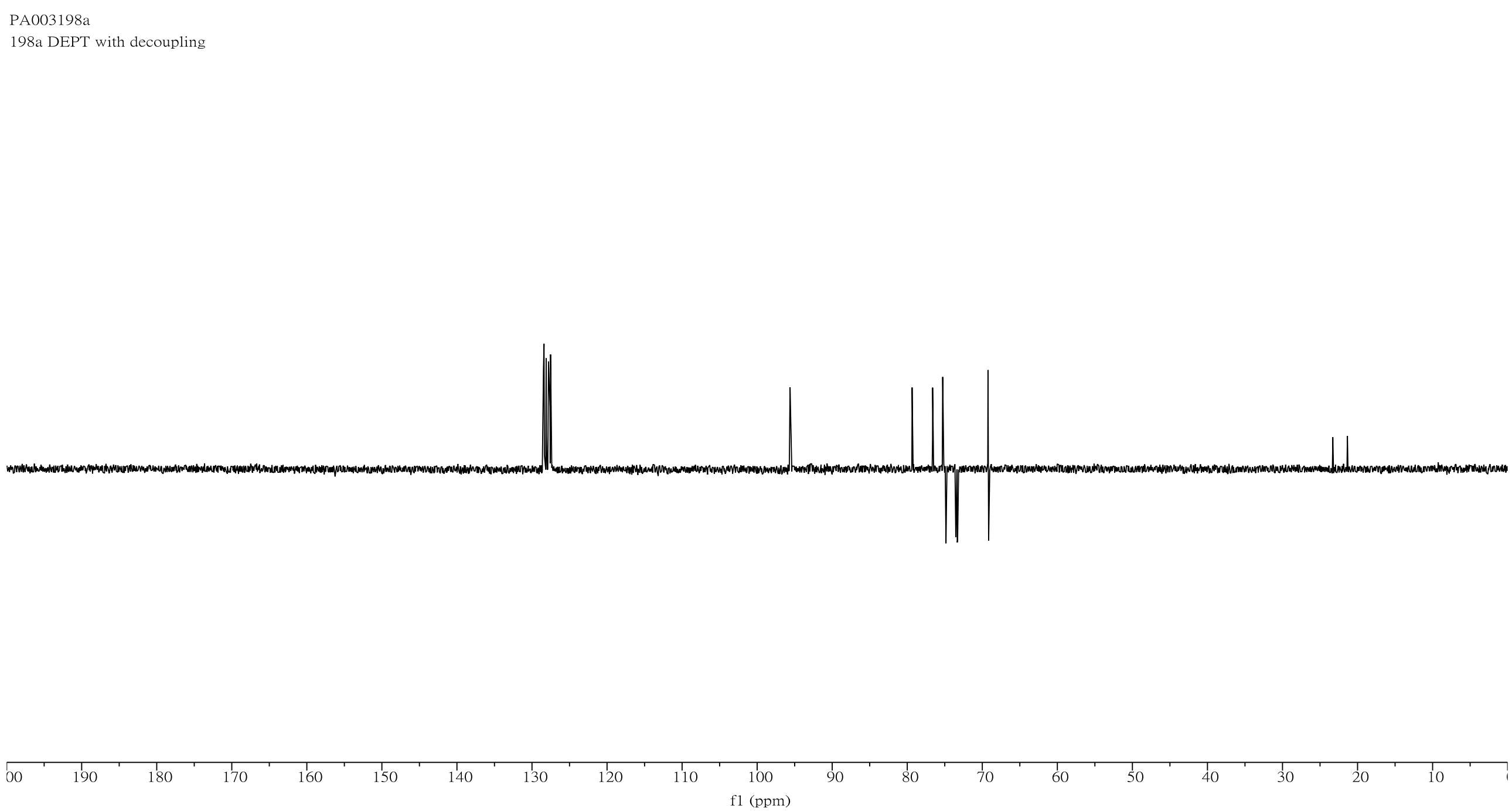

ESI-MS of **α-anomer GalOPr**:

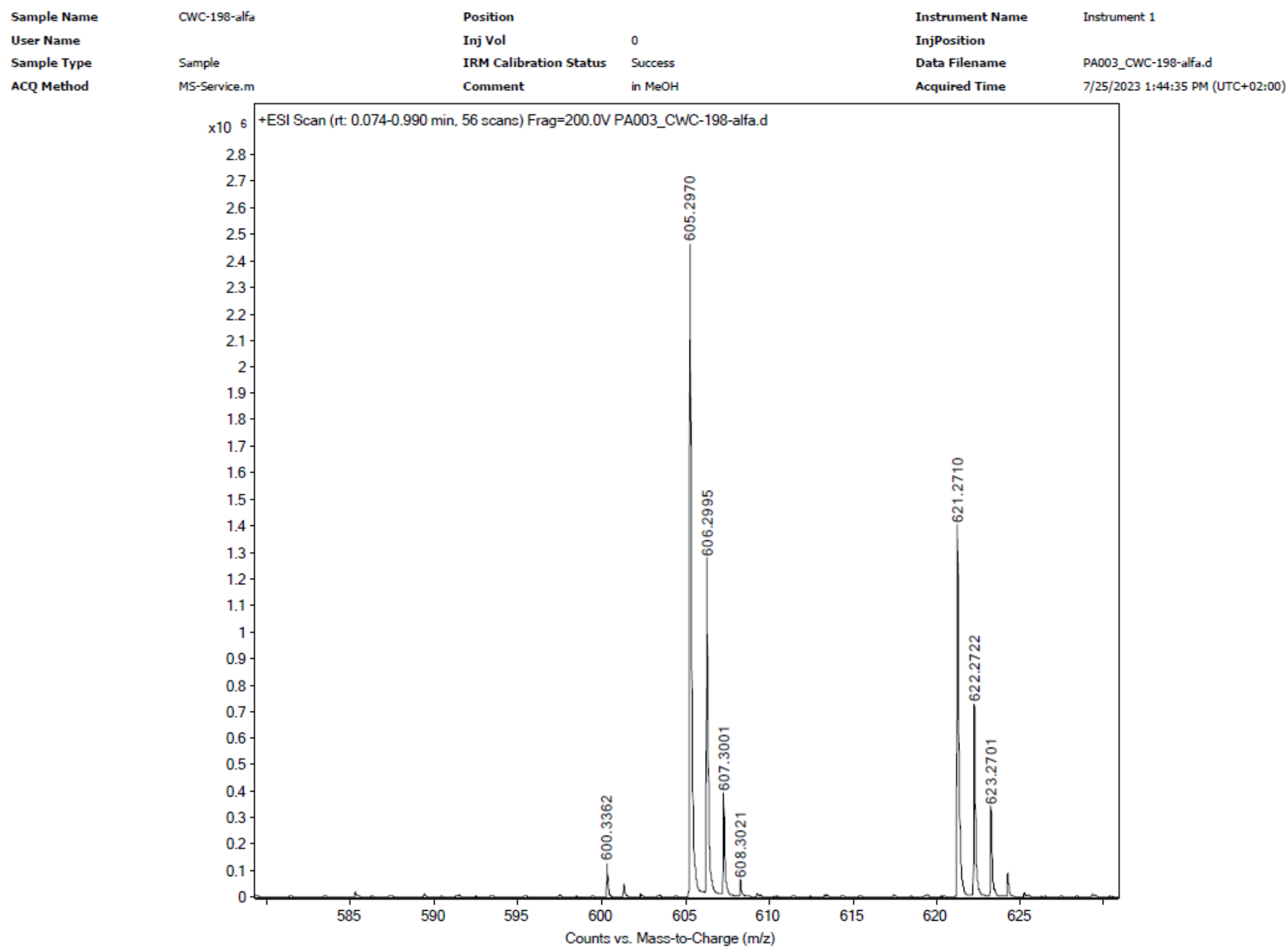

β-anomer of GalOPr:

^{1}H NMR (700 MHz, $CDCl_3$) δ 7.40 – 7.26 (m, 24H), 7.26 – 7.21 (m, 1H), 4.97 – 4.91 (m, 3H), 4.85 (d, *J* = 11.6 Hz, 1H), 4.80 (d, *J* = 12.0 Hz, 1H), 4.78 – 4.65 (m, 3H), 4.62 (d, *J* = 11.7 Hz, 1H), 4.57 (d, *J* = 11.5 Hz, 1H), 4.50 – 4.38 (m, 3H), 4.04 – 4.02 (m, 1H), 4.02 – 3.93 (m, 3H), 3.93 – 3.85 (m, 2H), 3.58 (d, *J* = 6.5 Hz, 1H), 3.56 – 3.48 (m, 3H), 1.23 (d, *J* = 6.3 Hz, 3H), 1.18 (d, *J* = 6.3 Hz, 3H). **^{13}C NMR** (151 MHz, $CDCl_3$) δ 138.84, 138.70, 138.62, 137.99, 128.39, 128.36, 128.32, 128.30, 128.28, 128.24, 128.20, 128.18, 128.12, 127.99, 127.84, 127.73, 127.68, 127.65, 127.51, 127.49, 127.41, 127.38, 102.44, 82.42, 79.62, 76.50, 75.16, 74.72, 74.41, 73.54, 73.50, 73.41, 73.36, 73.22, 73.17, 73.10, 72.09, 69.11, 69.06, 23.64, 22.12 ppm. **HRMS (ESI)** calcd for $C_{37}H_{42}O_6Na$ $[M + Na]^+$ 605.2879, found 605.2945.

^{1}H NMR (700 MHz, $CDCl_3$) of **β-anomer GalOPr**:

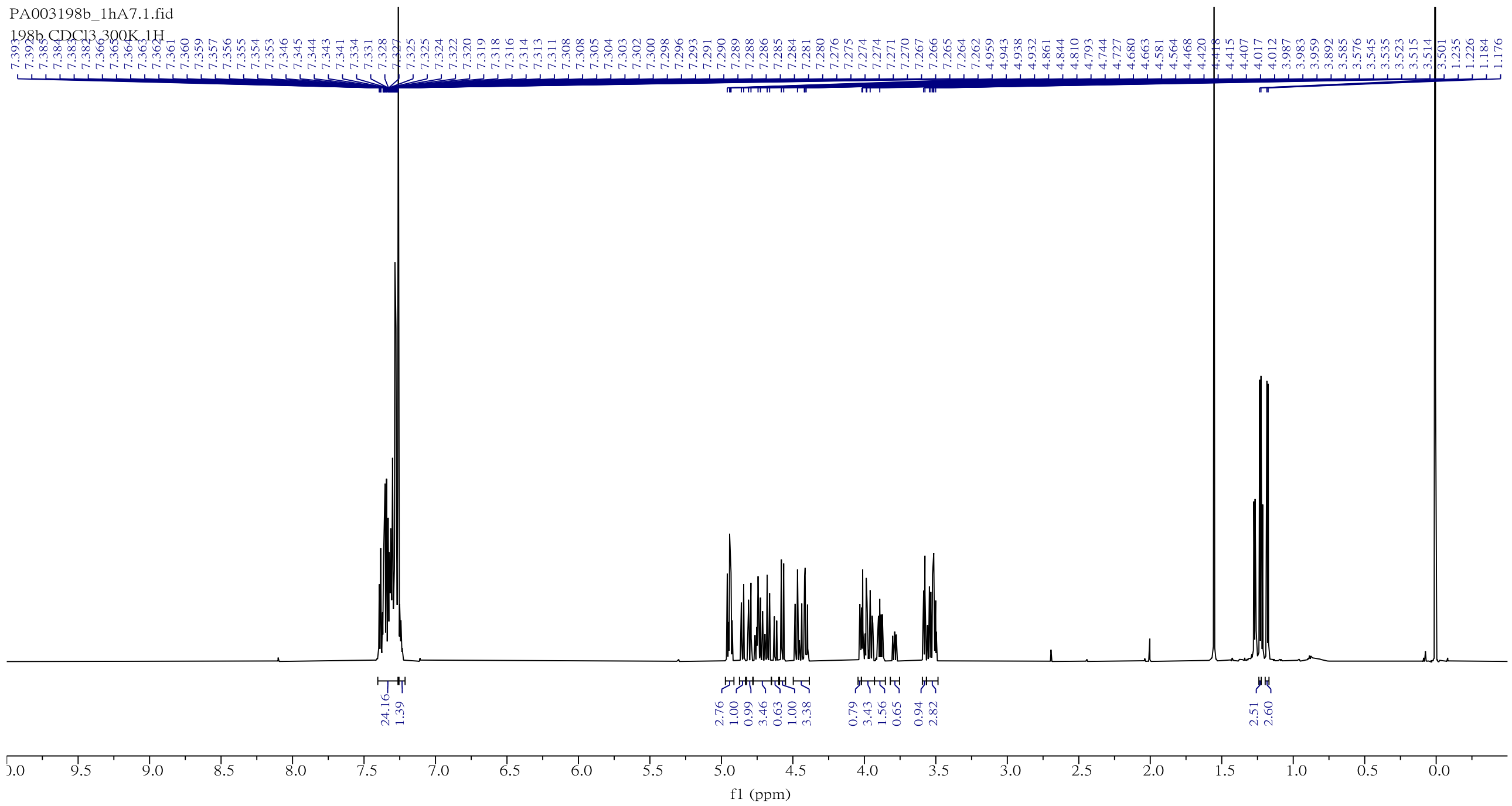

2D-HSQC (^{1}H, ^{13}C) (151 MHz, $CDCl_3$) of **β-anomer GalOPr**:

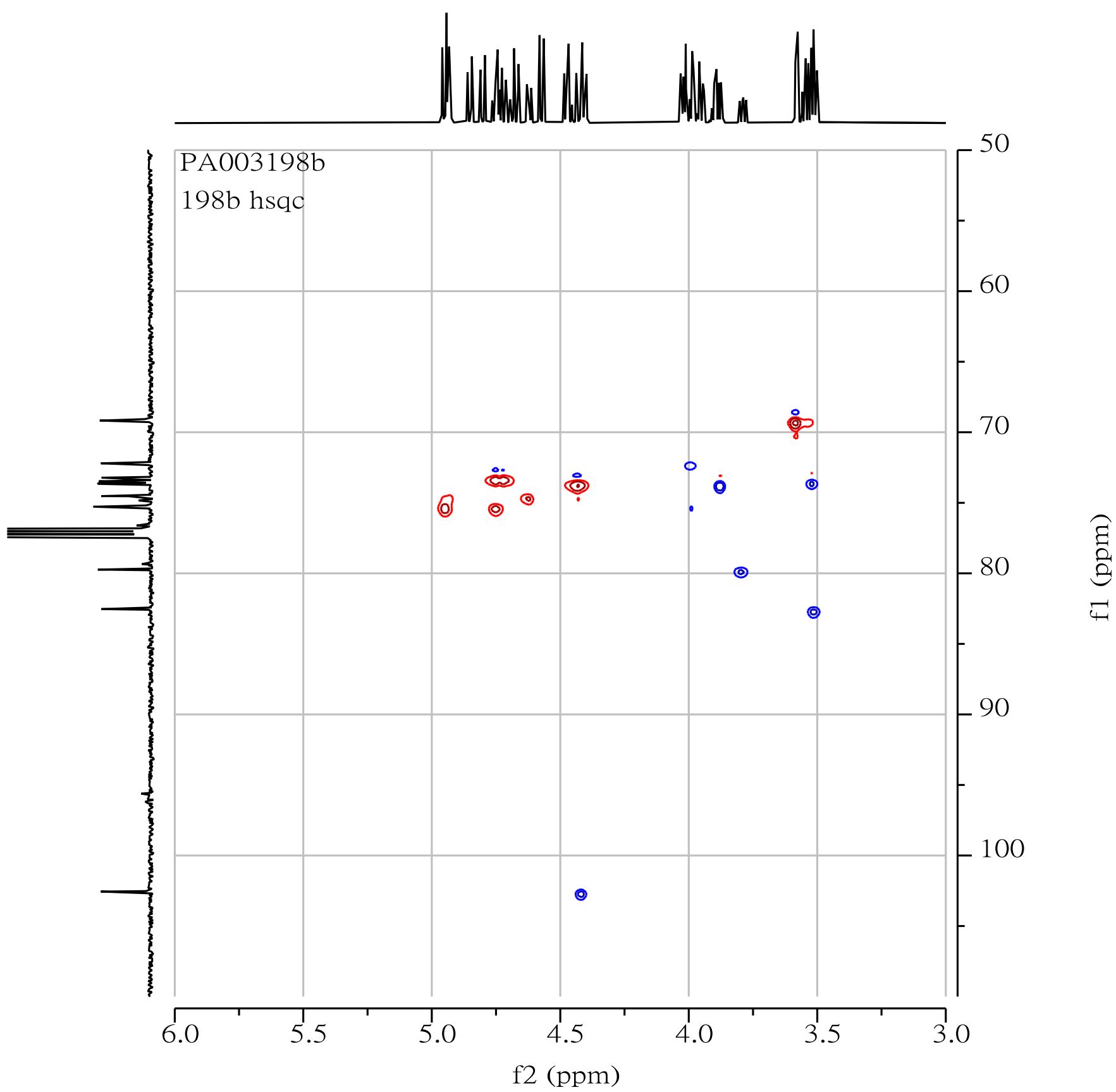

^{13}C NMR (151 MHz, $CDCl_3$) of **β-anomer GalOPr**:

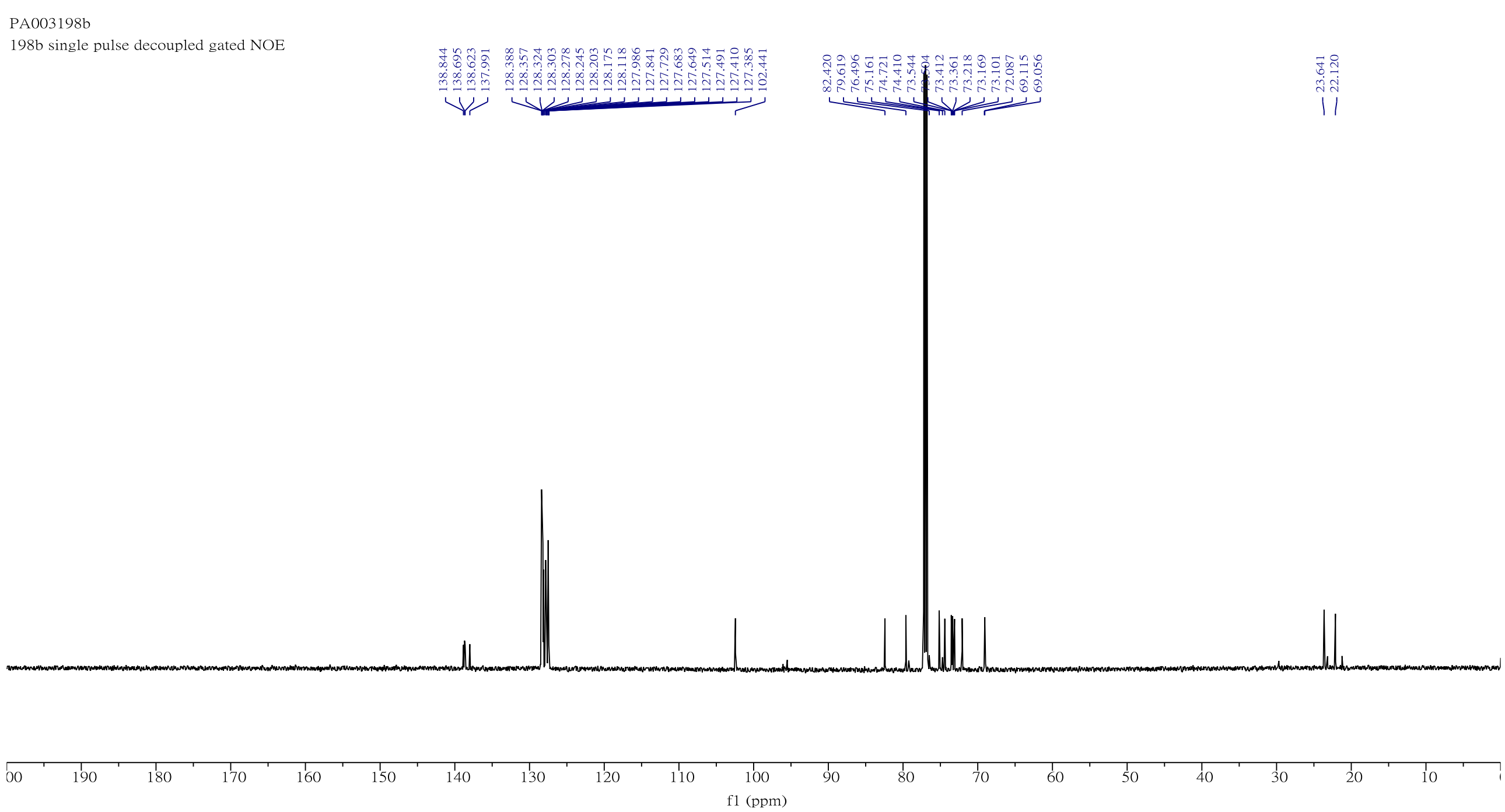

DEPT135 NMR (151 MHz, $CDCl_3$) of **β-anomer GalOPr**:

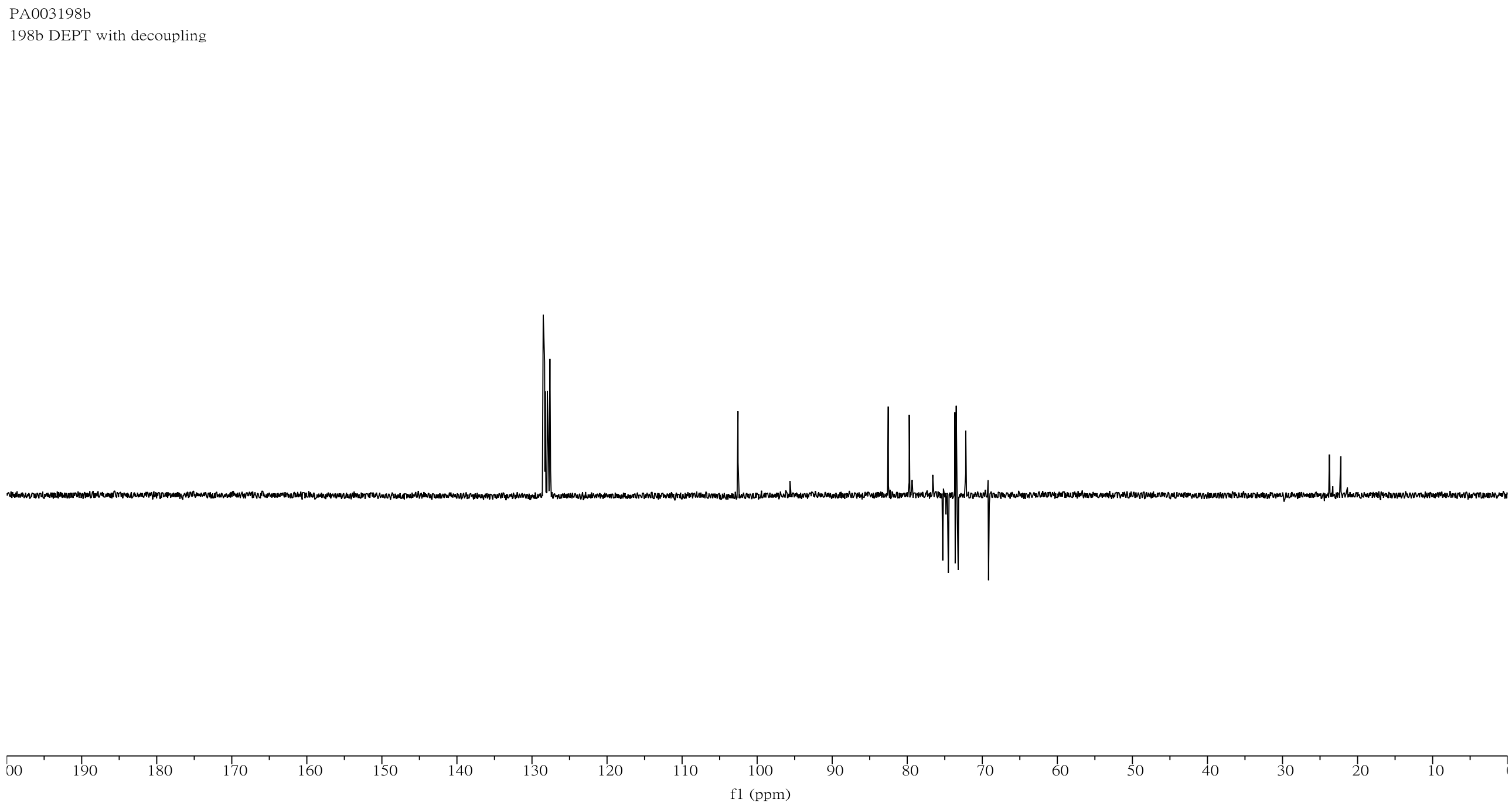

ESI-MS of **β-anomer GaliOPr**:

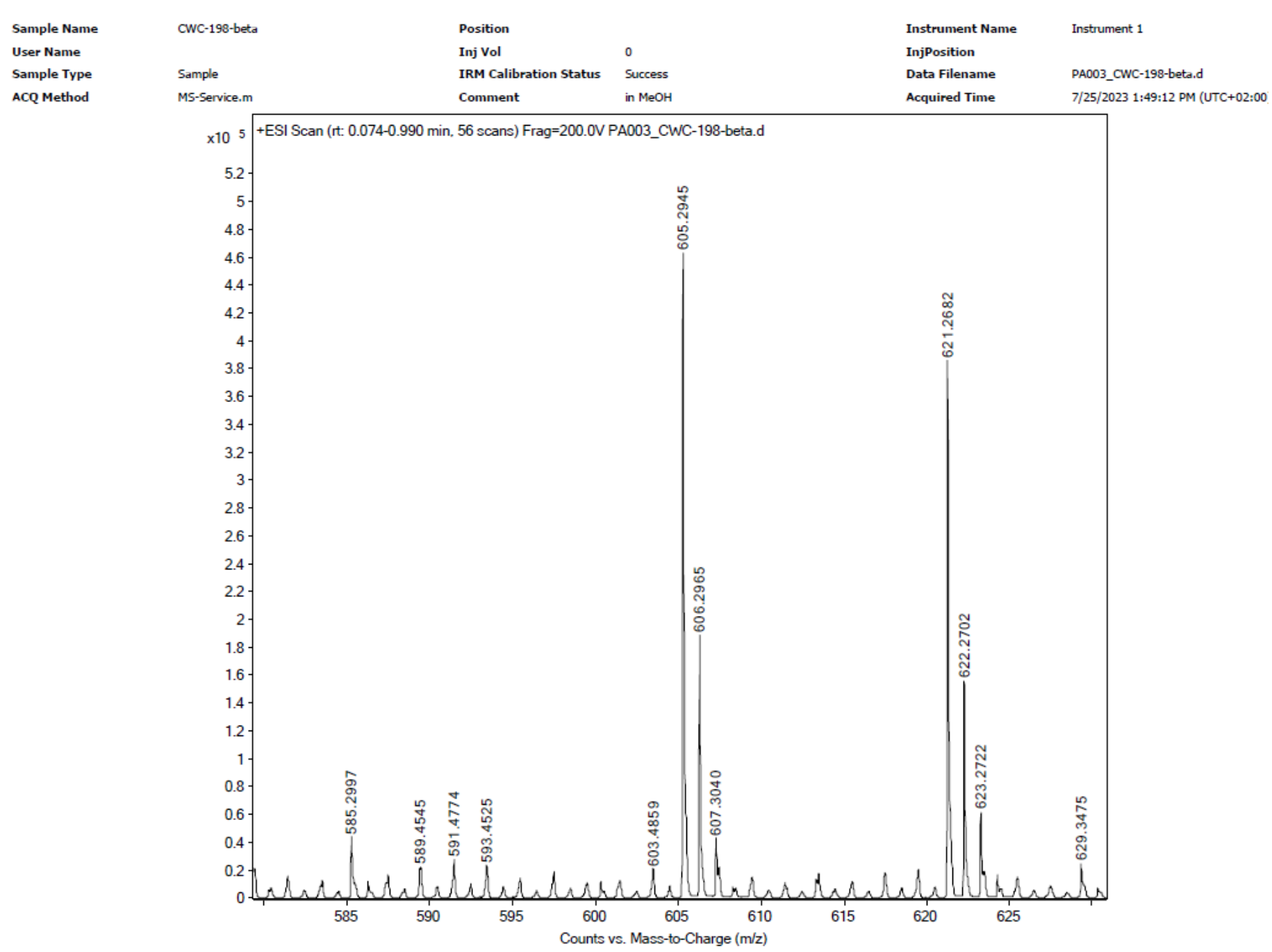

Trifluoroethyl 2,3,4,6-Tetra-O*-benzyl-*D-galactopyranoside

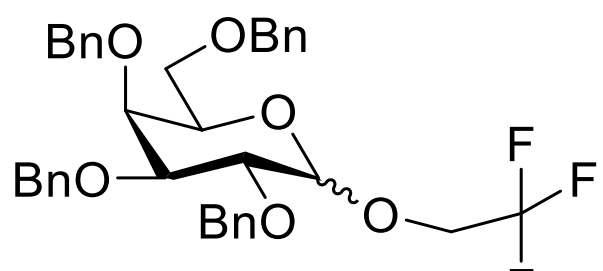

Gal-TFE

Normal Phase HPLC of **Gal-TFE**: The α/β ratio of the product is 76/24. Both the α- and β-anomers were isolated, and their identities were further confirmed via NMR spectroscopy. The detailed NMR assignments for both species are provided below.

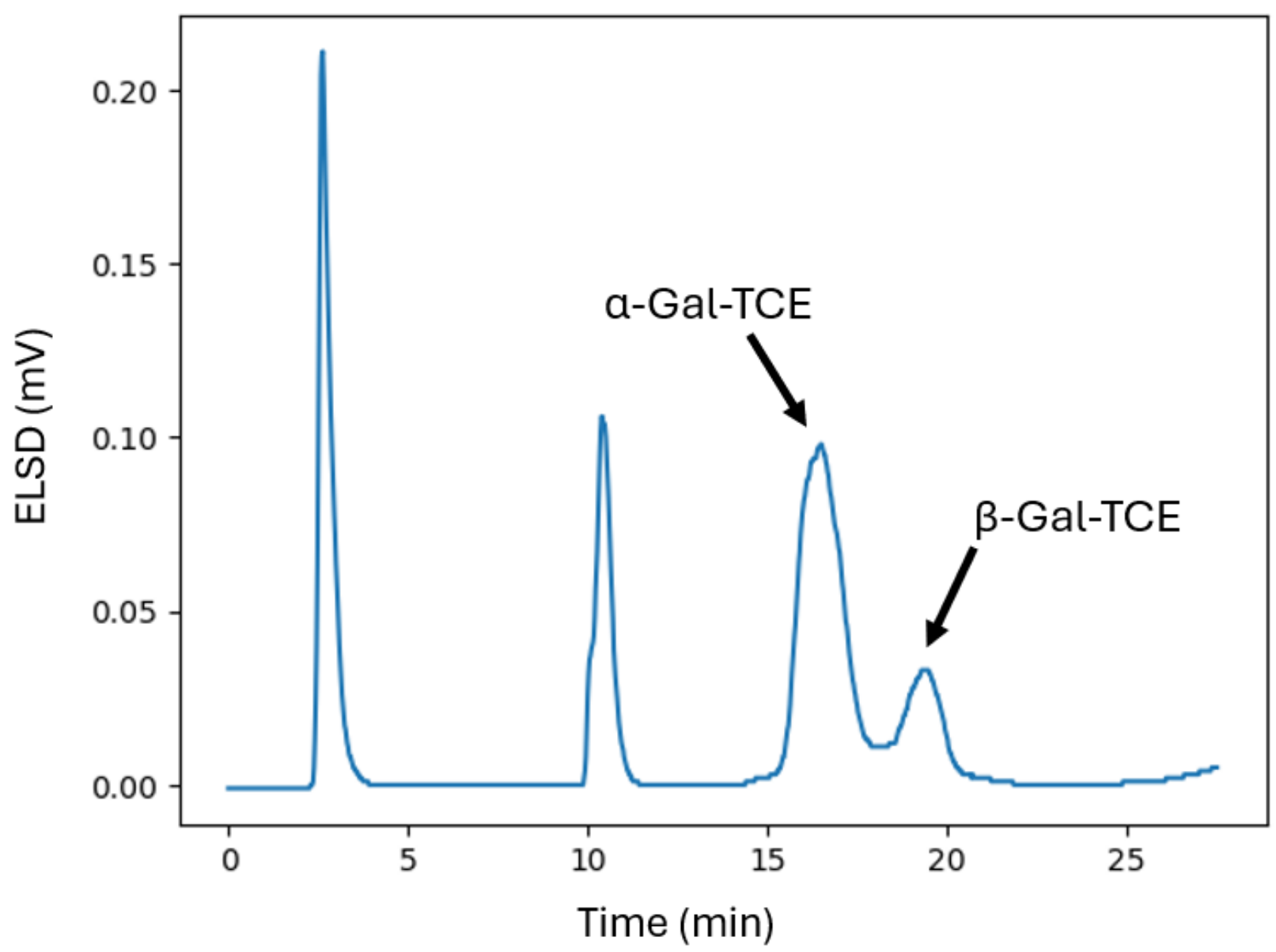

α-anomer of GalTFE:

^{1}H NMR (600 MHz, $CDCl_3$) δ 7.40 – 7.28 (m, 17H), 7.28 – 7.22 (m, 8H), 4.94 (d, *J* = 11.4 Hz, 1H), 4.88 (d, *J* = 3.7 Hz, 1H), 4.83 (dd, *J* = 21.3, 11.8 Hz, 2H), 4.73 (d, *J* = 11.6 Hz, 1H), 4.66 (d, *J* = 11.9 Hz, 1H), 4.56 (d, *J* = 11.4 Hz, 1H), 4.46 (d, *J* = 11.8 Hz, 1H), 4.40 (d, *J* = 11.8 Hz, 1H), 4.07 (dd, *J* = 10.0, 3.7 Hz, 1H), 3.99 – 3.83 (m, 5H), 3.50 (d, *J* = 6.4 Hz, 2H). **^{13}C NMR** (151 MHz, $CDCl_3$) δ 138.70, 138.47, 138.43, 137.85, 128.41, 128.36, 128.25, 127.95, 127.76, 127.72, 127.65, 127.52, 127.46, 98.21, 78.55, 76.15, 74.80, 73.46, 73.38, 73.31, 70.03, 68.73, 64.61, 64.38, 29.69, 29.35 ppm. **HRMS (ESI)** calcd for $C_{36}H_{37}F_3O_6Na$ $[M + Na]^+$ 645,2440, found 645.2419; $C_{36}H_{37}F_3O_6K$ $[M + K]^+$ 661.2179, found 661.2180.

^{1}H NMR (600 MHz, $CDCl_3$) of **α-anomer GalTFE**:

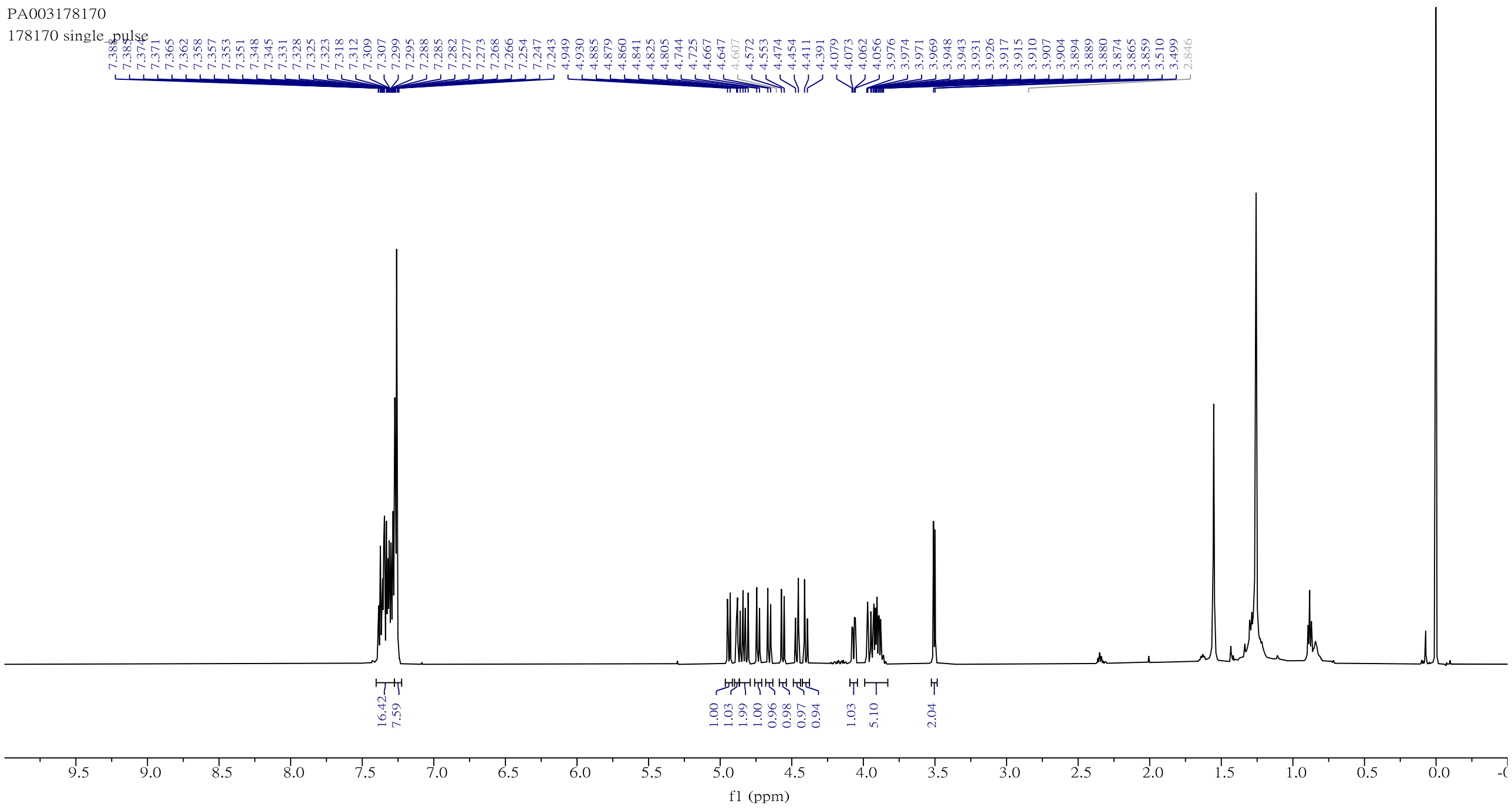

^{13}C NMR (151 MHz, $CDCl_3$) of **α-anomer GalTFE**:

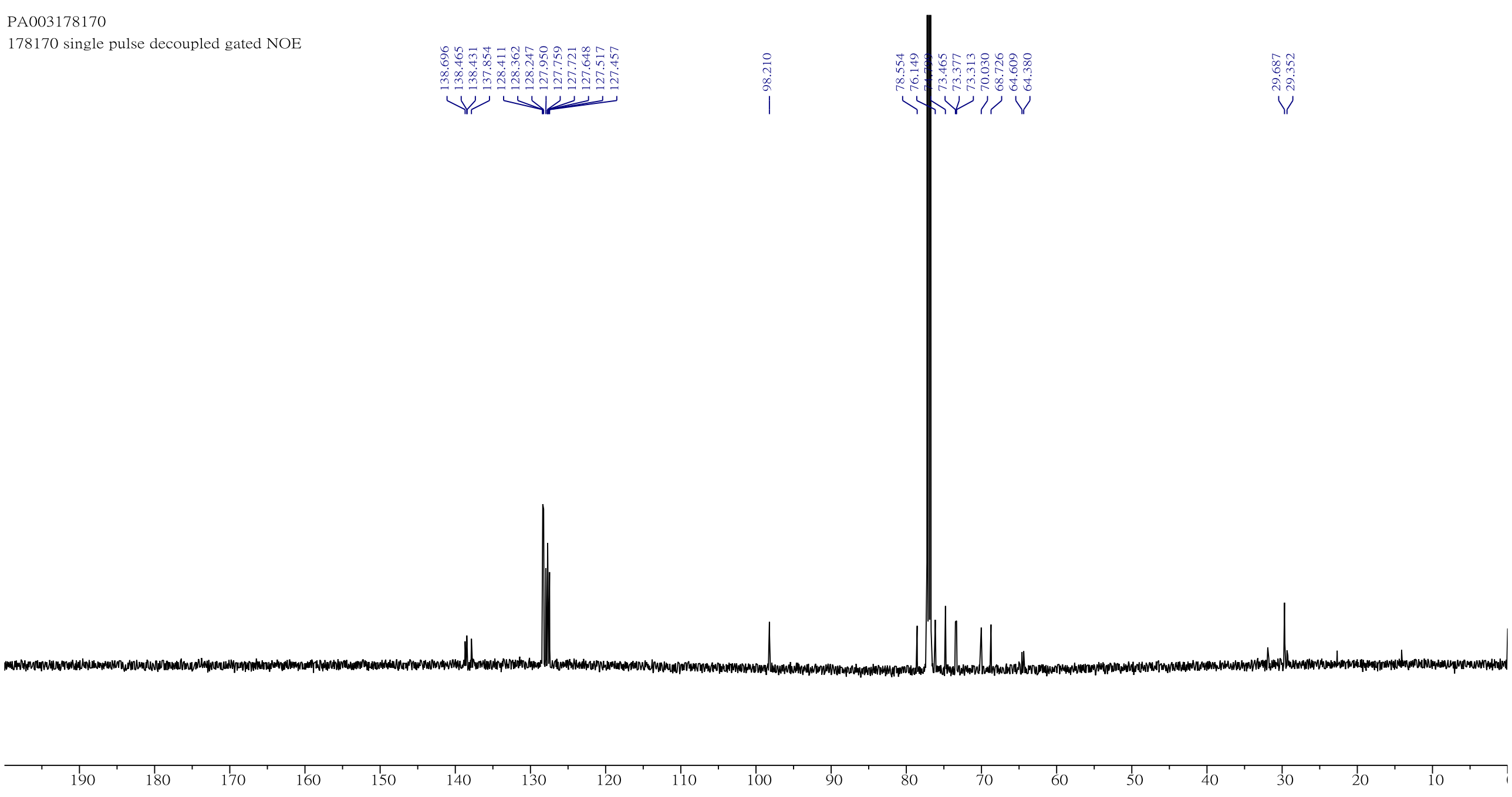

DEPT135 NMR (151 MHz, $CDCl_3$) of **α-anomer GalTFE**:

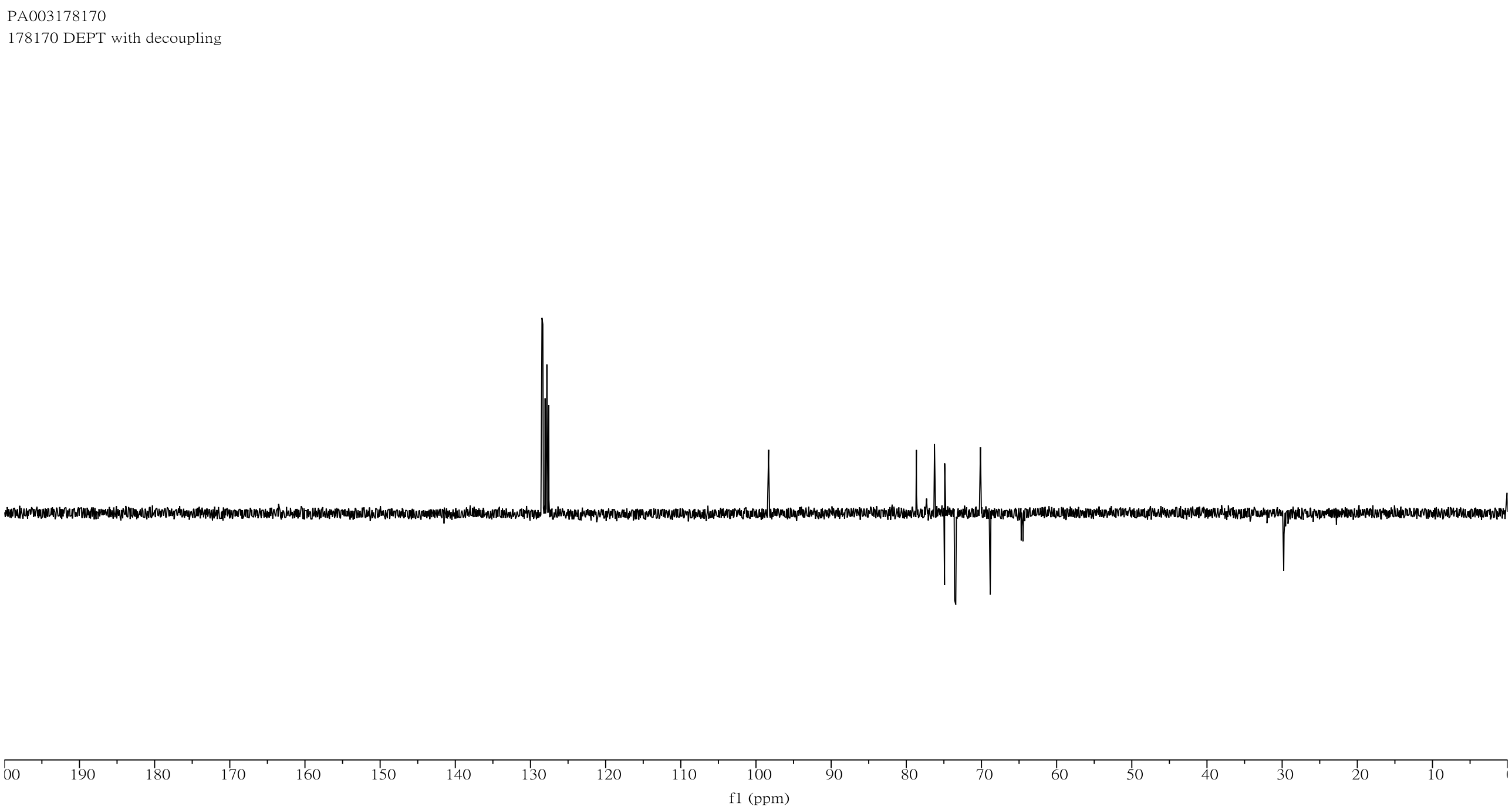

2D-HSQC (^{1}H, ^{13}C) (151 MHz, $CDCl_3$) of **α-anomer GalTFE**:

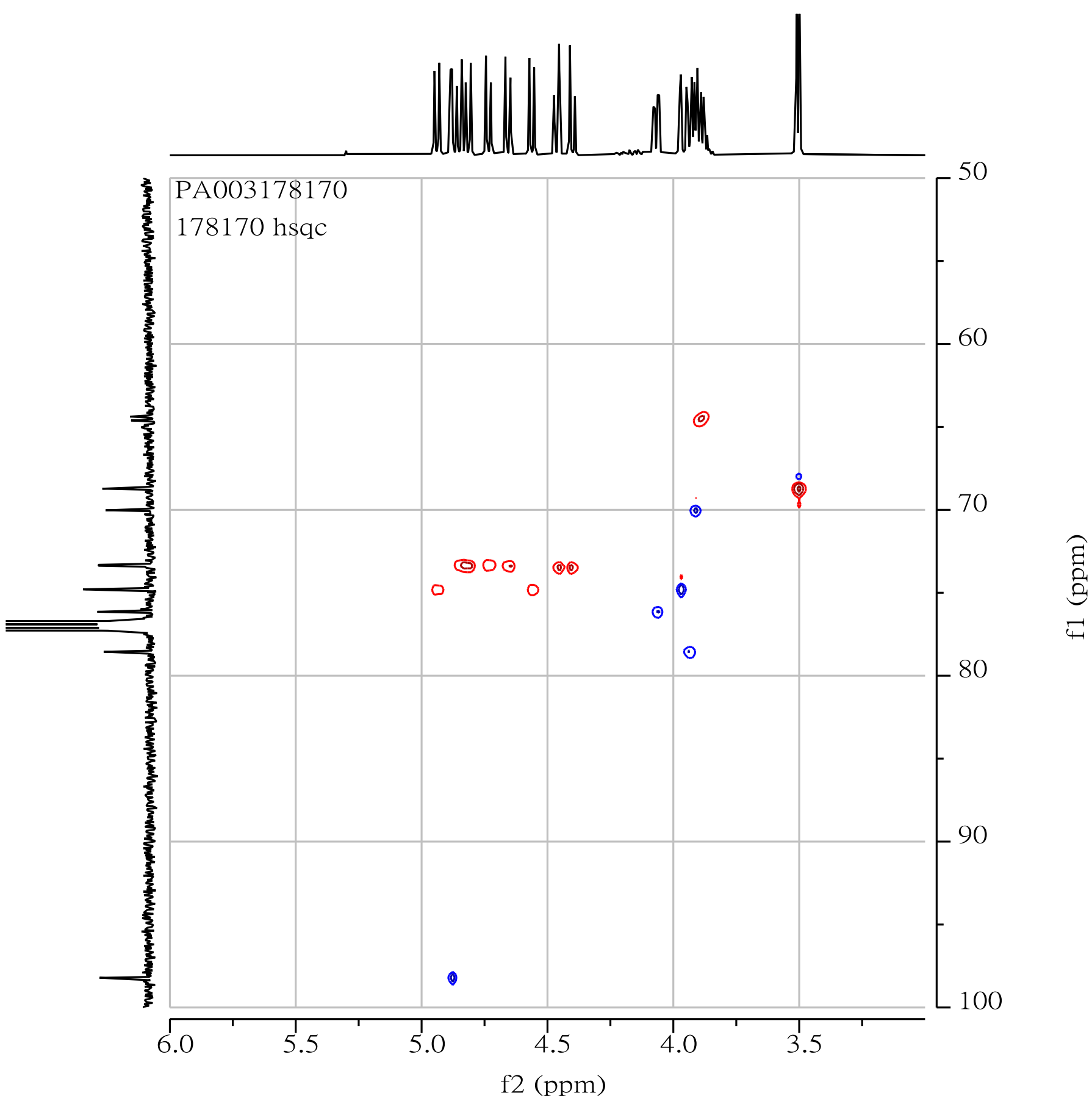

ESI-MS of **α-anomer GalTFE**:

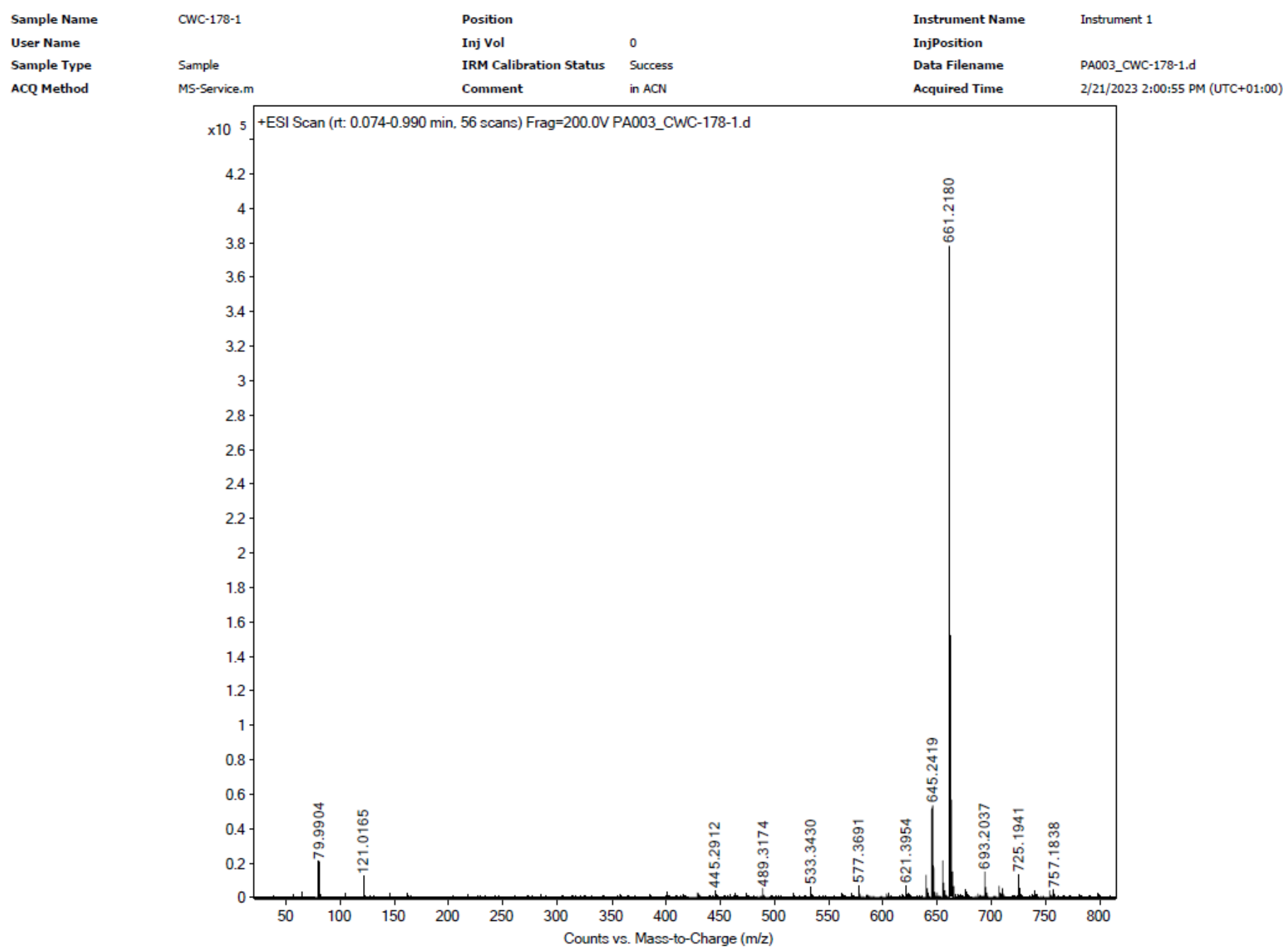

β-anomer of GalTFE:

^{1}H NMR (600 MHz, $CDCl_3$) δ7.38 – 7.26 (m, 24H), 7.25 (s, 1H), 4.97 – 4.88 (m, 2H), 4.78 – 4.69 (m, 3H), 4.63 – 4.59 (m, 1H), 4.50 – 4.43 (m, 1H), 4.42 (d, J = 5.1 Hz, 1H), 4.23 – 4.12 (m, 1H), 3.98 – 3.91 (m, 1H), 3.89 – 3.86 (m, 1H), 3.86 – 3.82 (m, 1H), 3.64 – 3.49 (m, 3H). **^{13}C NMR** (151 MHz, $CDCl_3$) δ 38.70, 138.47, 138.43, 137.85, 128.41, 128.36, 128.25, 127.95, 127.76, 127.72, 127.65, 127.52, 127.46, 98.21, 78.55, 76.15, 74.80, 73.46, 73.38, 73.31, 70.03, 68.73, 64.61, 64.38, 29.69, 29.35 ppm. **HRMS (ESI)** calcd for $C_{36}H_{37}F_3O_6K$ $[M + K]^+$ 661.2179, found 661.2108.

^{1}H NMR (600 MHz, $CDCl_3$) of **β-anomer GalTFE**:

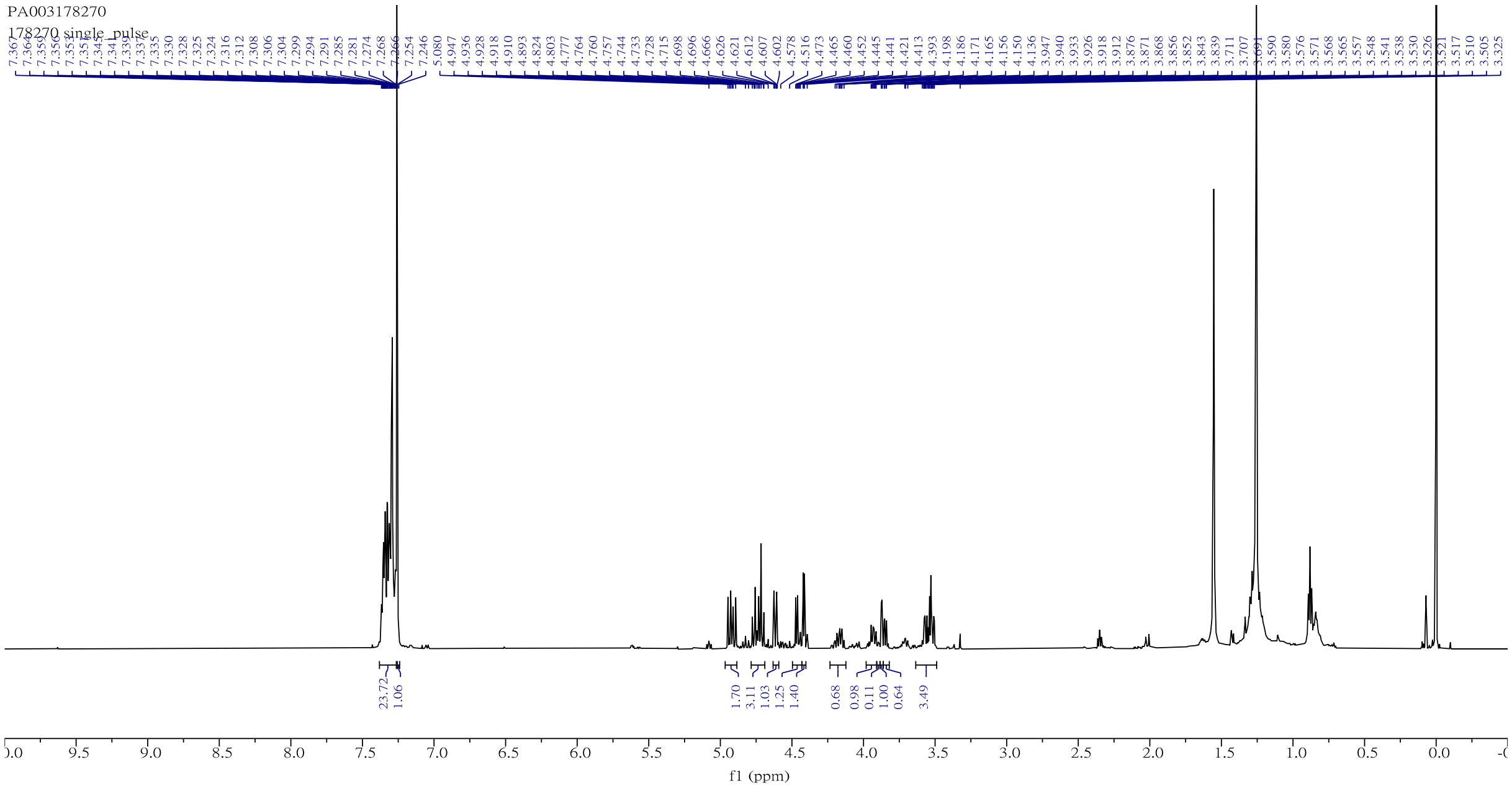

^{13}C NMR (151 MHz, $CDCl_3$) of **β-anomer GalTFE**:

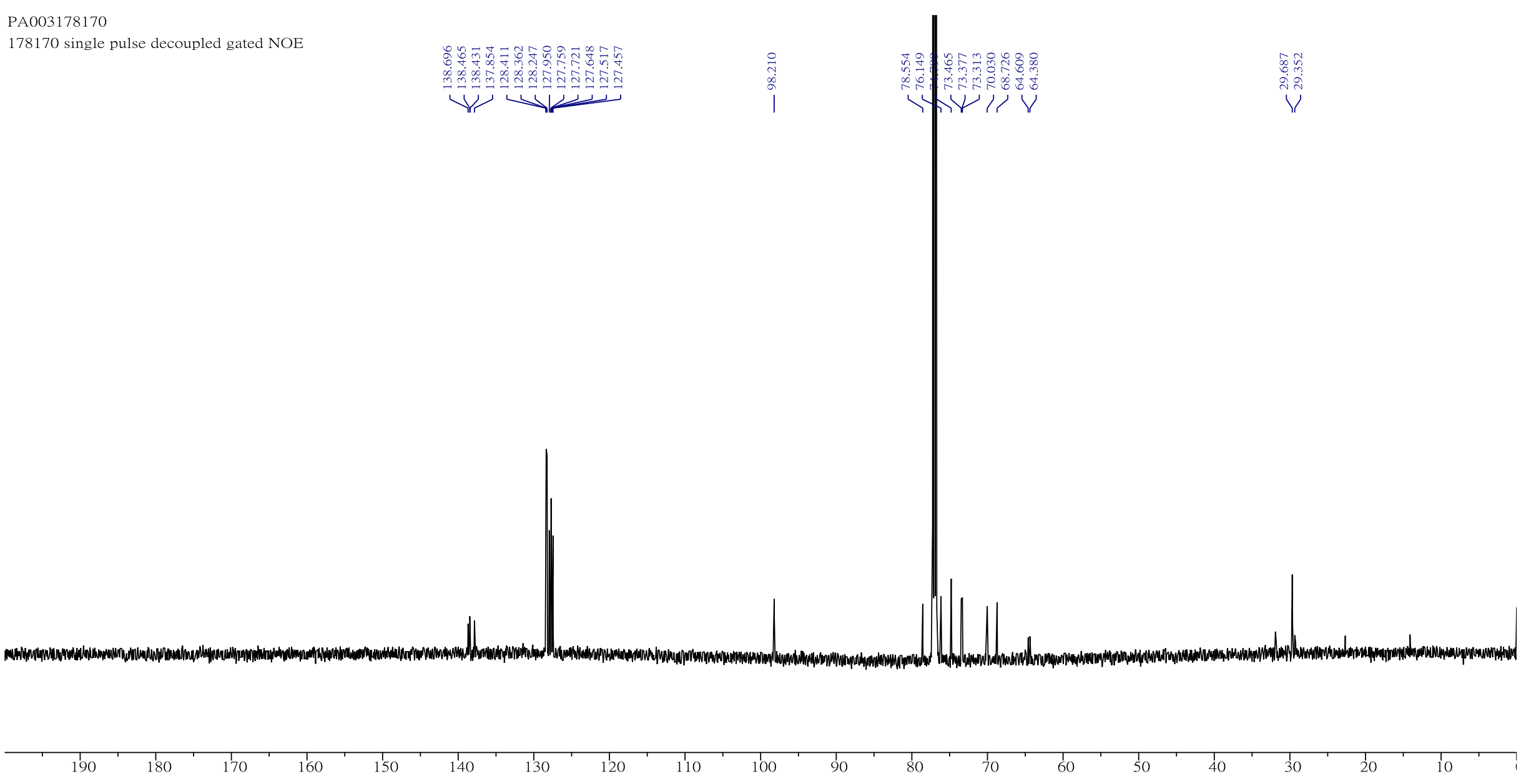

2D-HSQC (^{1}H, ^{13}C) (151 MHz, $CDCl_3$) of **β-anomer GalTFE**:

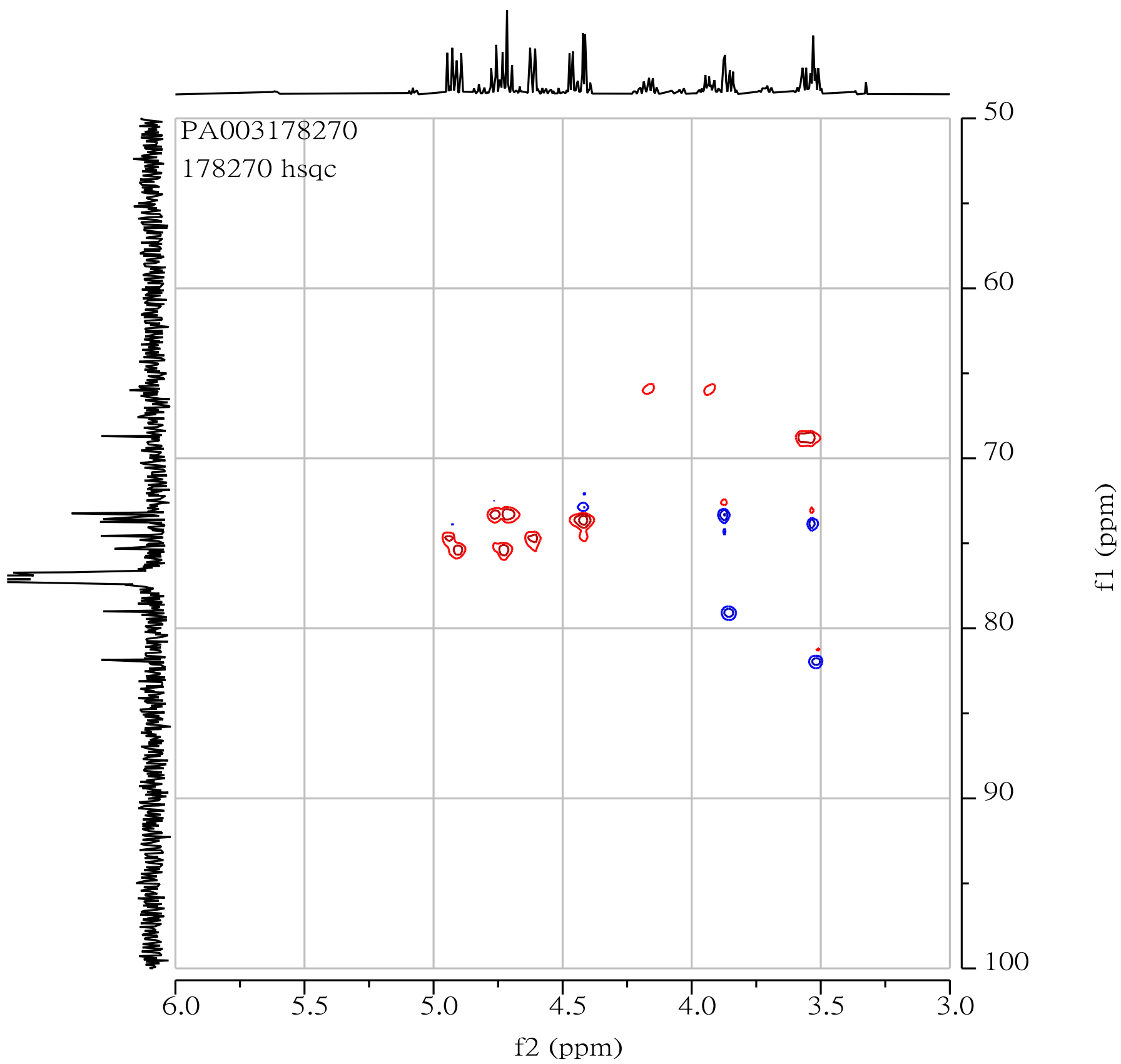

ESI-MS of **β-anomer GalTFE**:

**Sample Name**	CWC-178-2	**Position**		**Instrument Name**	Instrument 1
**User Name**		**Inj Vol**	0	**InjPosition**	
**Sample Type**	Sample	**IRM Calibration Status**	Success	**Data Filename**	PA003_CWX-178-2.d
**ACQ Method**	MS-Service.m	**Comment**	in ACN	**Acquired Time**	2/21/2023 2:19:05 PM (UTC+01:00)

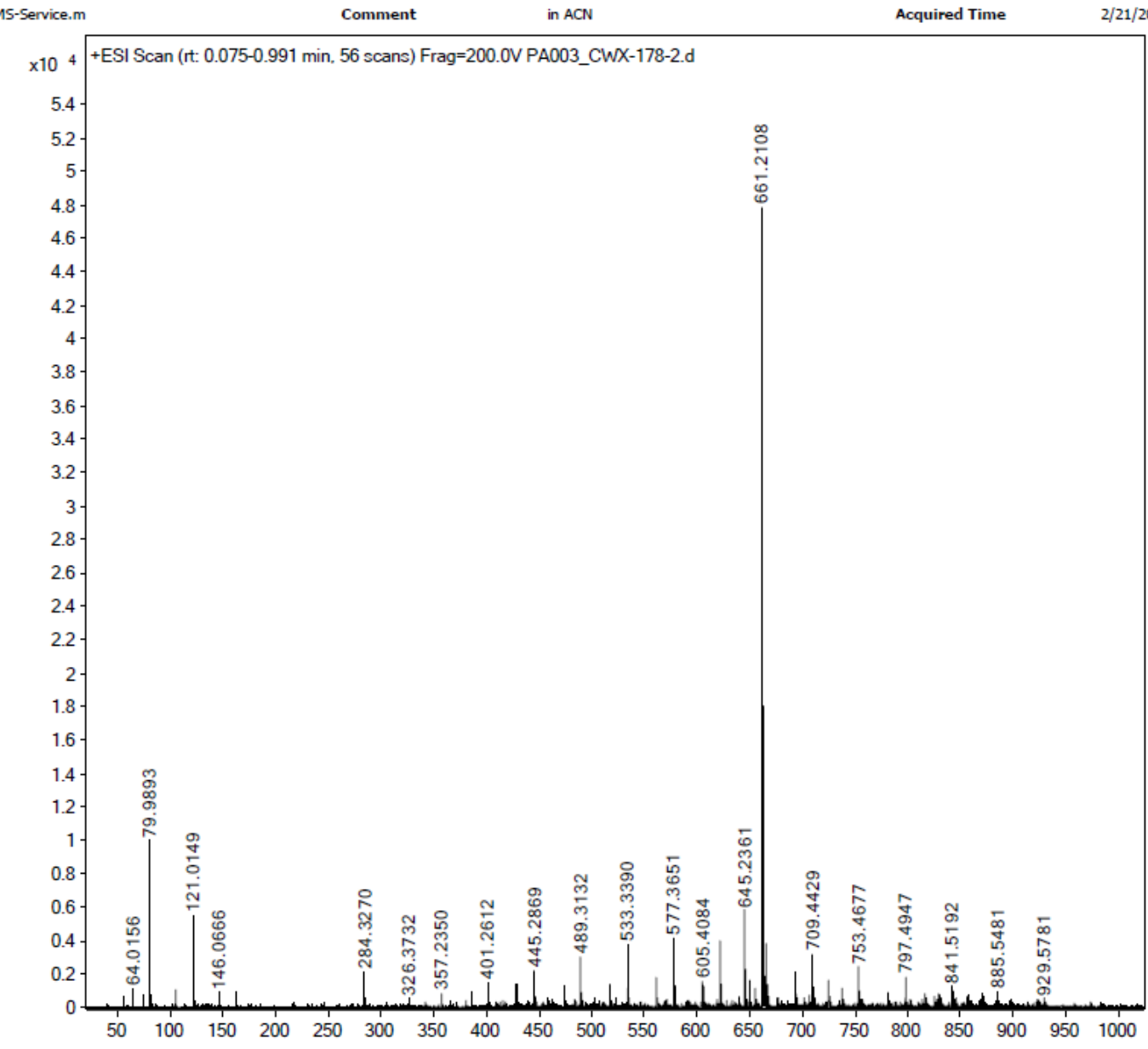

1,2,3,4,6-Penta-O-*benzyl-*D-galactopyranoside

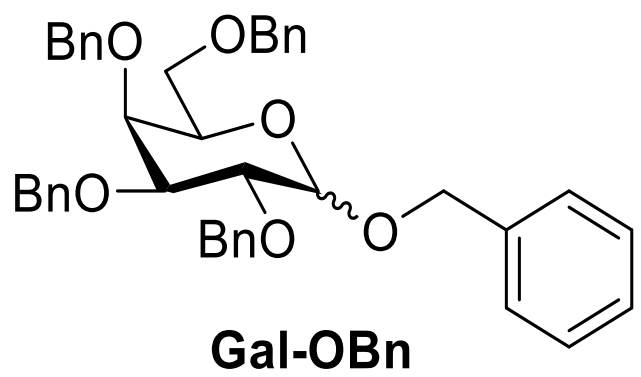

Gal-OBn
(α/β = 78/22)

Normal Phase HPLC of **α/β-Gal-OBn**: The α/β anomers could not be separated by HPLC; therefore, their ratio was determined by NMR. The products were isolated, and their α- and β-anomers were further confirmed via NMR spectroscopy. The detailed NMR assignments for both species are provided below.

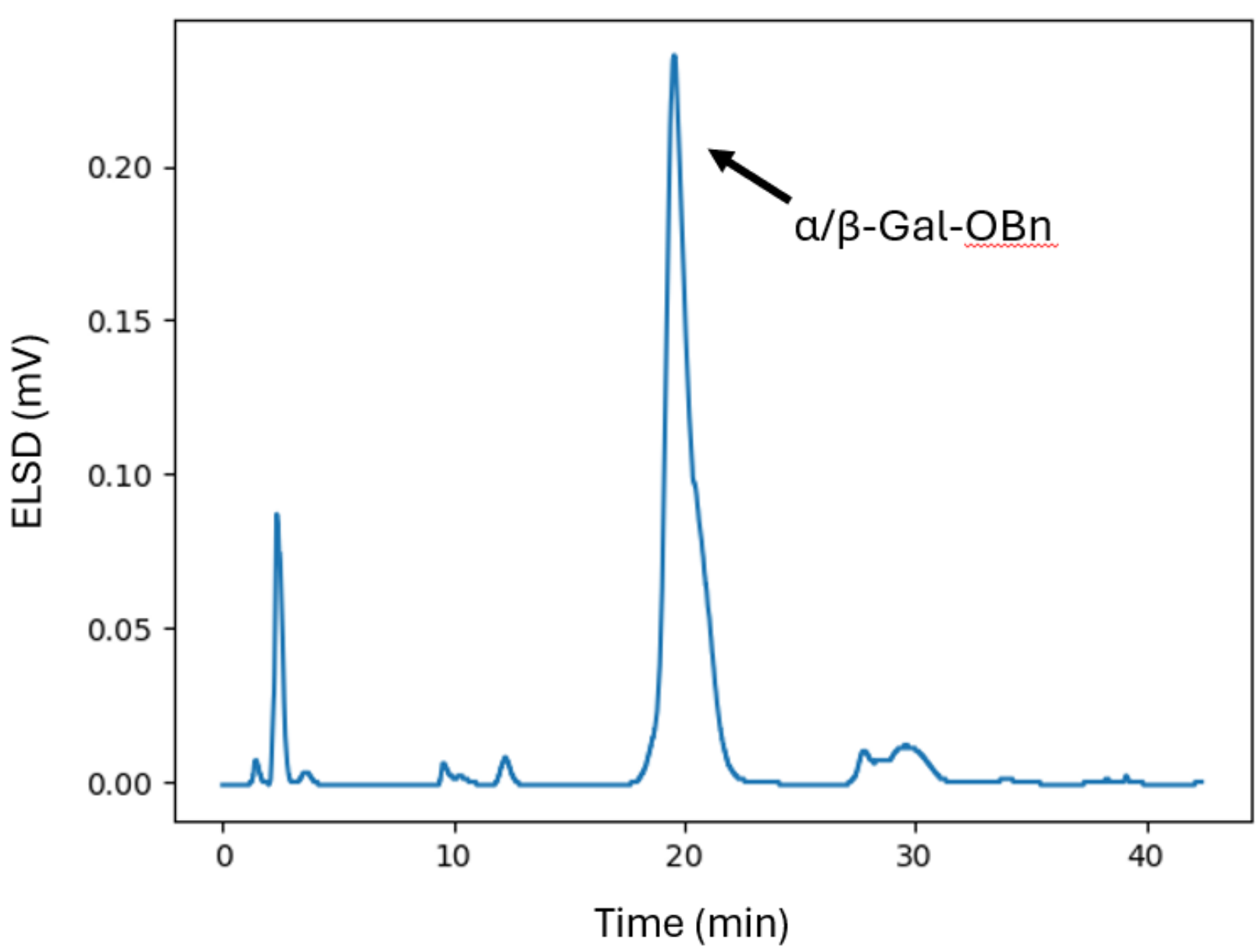

^{1}H NMR (700 MHz, $CDCl_3$) of **GalOBn**, region shown: 5.1-4.5 ppm. For the α-product, the diagnostic peak appears at δ 4.90 (d, *J* = 3.5 Hz, 1H), corresponding to the anomeric proton. For the β-product, the diagnostic peak appears at δ 4.64 (dd, *J* = 11.9, 3.5, 2H), corresponding to the benzyl methylene (CH_2) protons. The α/β ratio was determined from the integral values of the α-anomeric proton and the β-methylene signals. The ratio 1: (0.61/2) corresponds to approximately 77% α-anomer.

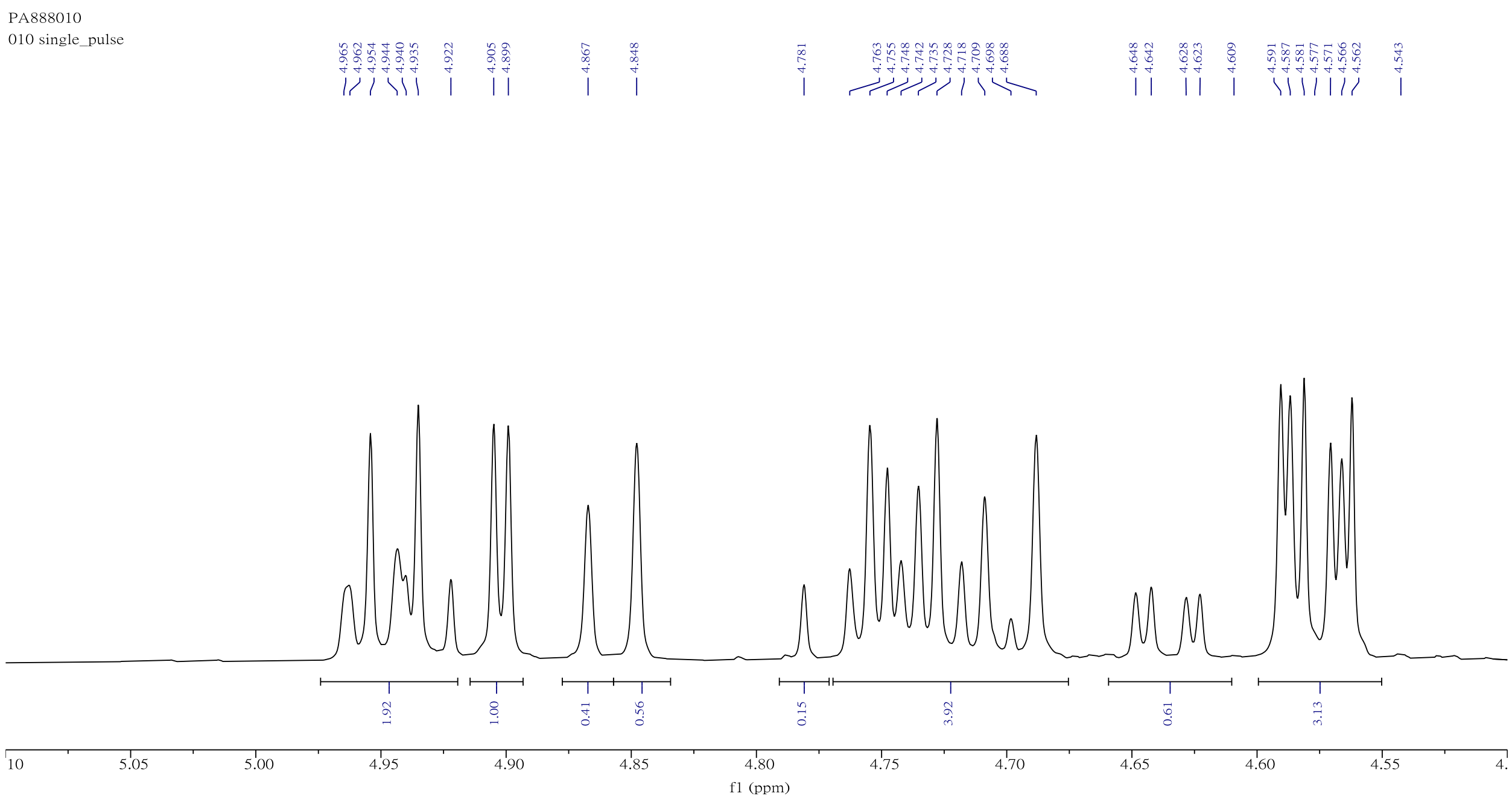

^{1}H NMR (600 MHz, $CDCl_3$) δ 7.41 – 7.37 (m, 4H), 7.36 – 7.26 (m, 27H), 7.26 – 7.23 (m, 3H), 4.97 – 4.92 (m, 2H), 4.90 (d, *J* = 3.5 Hz, 1H), 4.85 (s, 1H), 4.77 – 4.68 (m, 4H), 4.64 (dd, *J* = 11.9, 3.5 Hz, 1H), 4.60 – 4.55 (m, 3H), 4.49 – 4.43 (m, 2H), 4.40 (d, *J* = 11.8 Hz, 1H), 4.02 (dd, *J* = 10.7, 3.0 Hz, 2H), 4.00 – 3.95 (m, 2H), 3.92 – 3.86 (m, 1H), 3.54 – 3.50 (m, 1H), 3.48 (d, *J* = 5.9 Hz, 1H). **^{13}C NMR** (151 MHz, $CDCl_3$) 138.88, 138.68, 138.58, 138.02, 137.39, 128.38, 128.36, 128.32, 128.30, 128.23, 128.19, 127.87, 127.85, 127.74, 127.71, 127.69, 127.51, 127.44, 102.82, 96.06, 79.58, 79.17, 77.21, 77.00, 76.79, 76.49, 75.06, 74.71, 73.47, 73.44, 73.21, 73.16, 70.88, 69.47, 69.01, 68.95 ppm. **HRMS (ESI)** calcd for $C_{41}H_{42}O_6Na$ $[M + Na]^+$ 653.2879, found 653.2907.

^{1}H NMR (600 MHz, $CDCl_3$) of **GalOBn**:

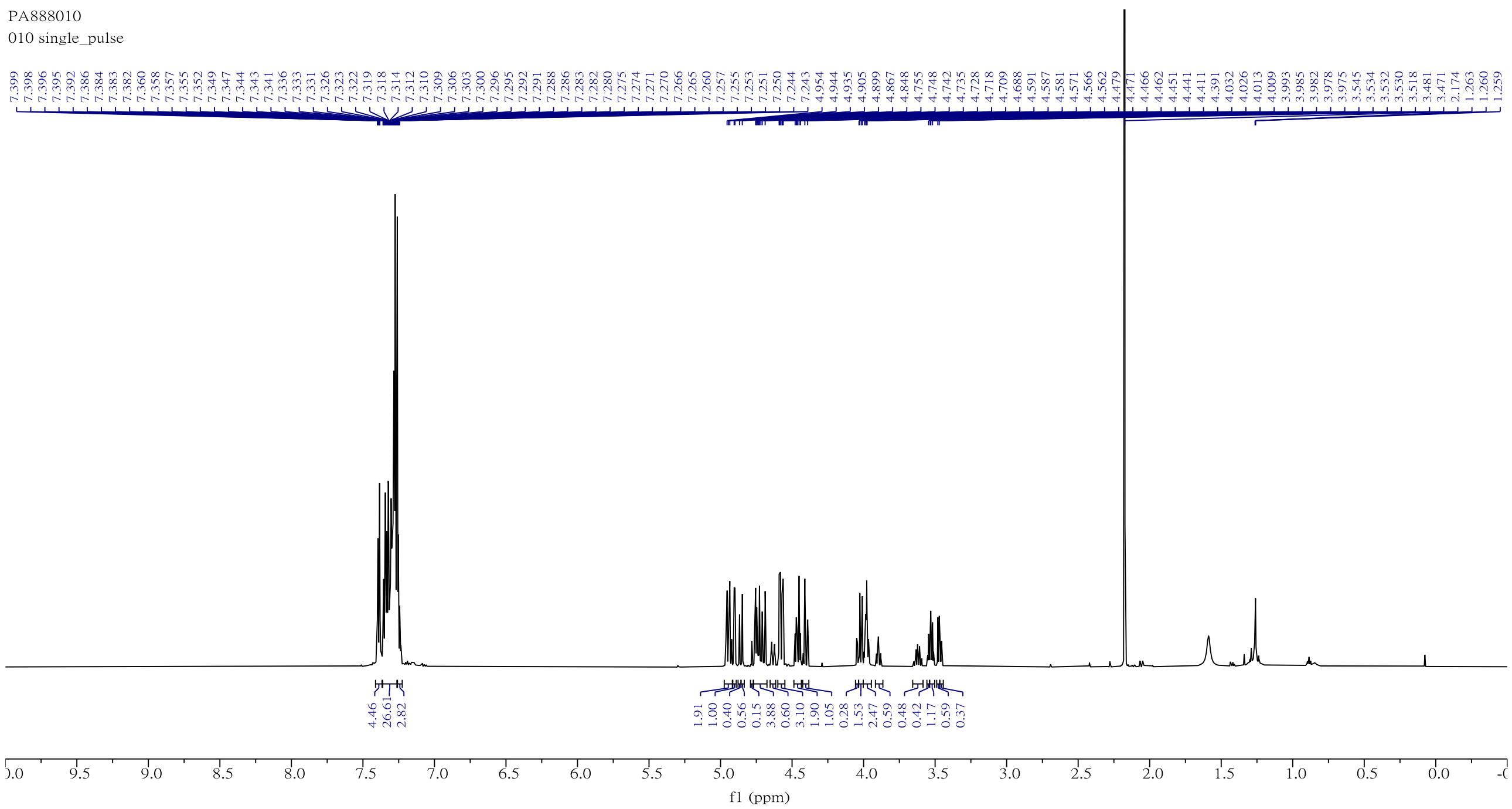

2D-HSQC (^{1}H, ^{13}C) (151 MHz, $CDCl_3$) of **GalOBn**:

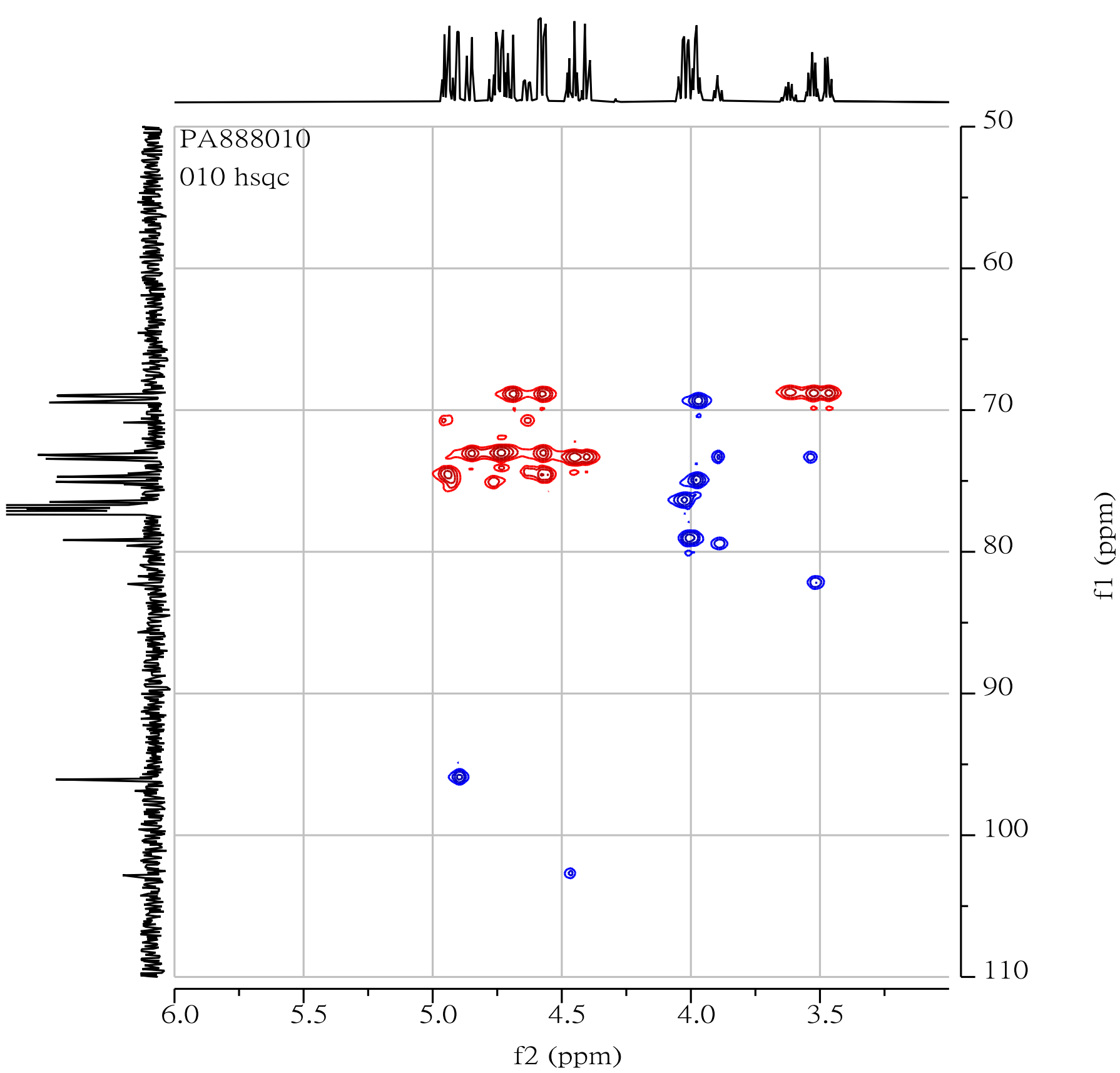

^{13}C NMR (151 MHz, $CDCl_3$) of **GalOBn**:

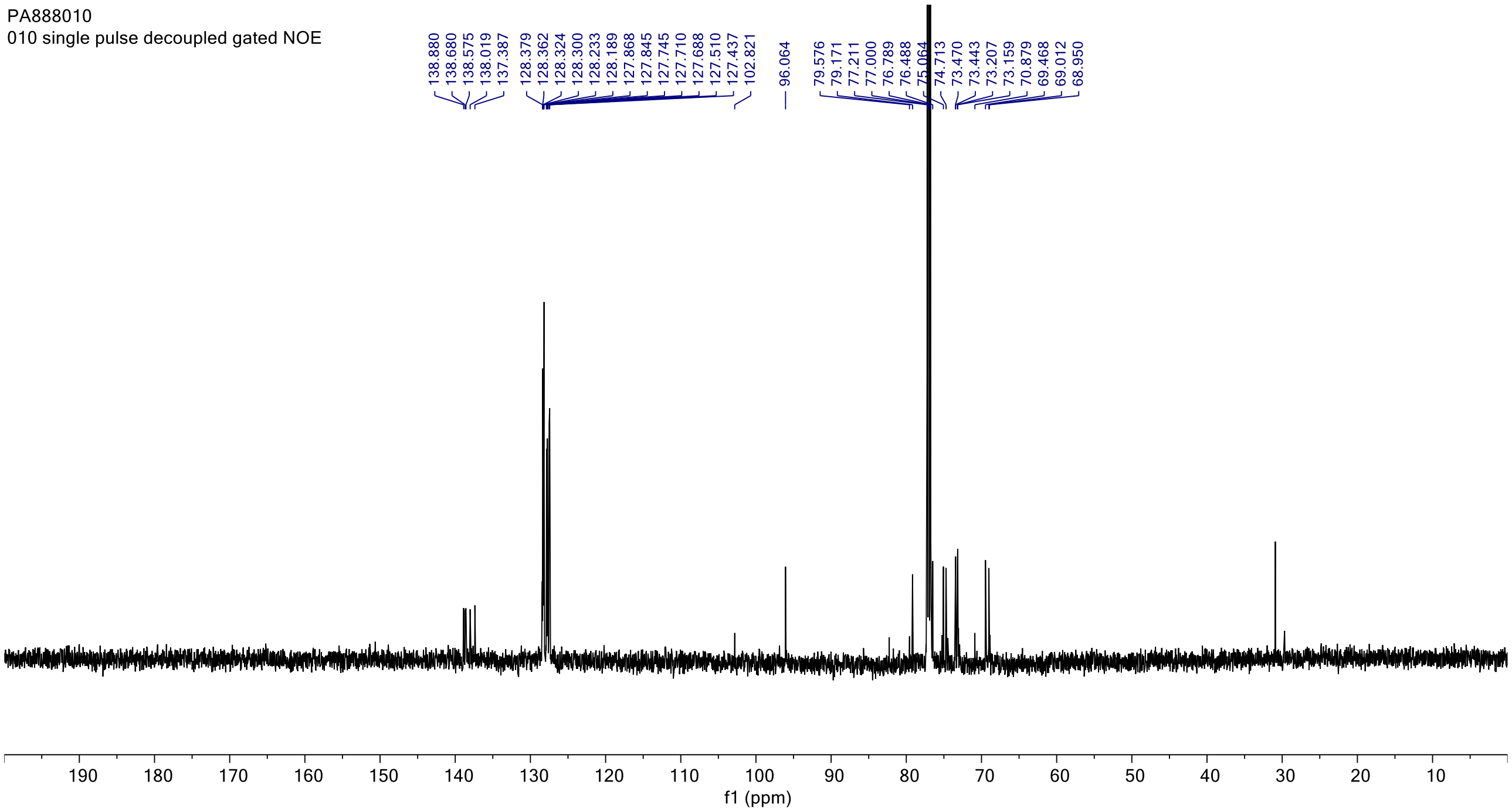

DEPT135 NMR (151 MHz, $CDCl_3$) of **GalOBn**:

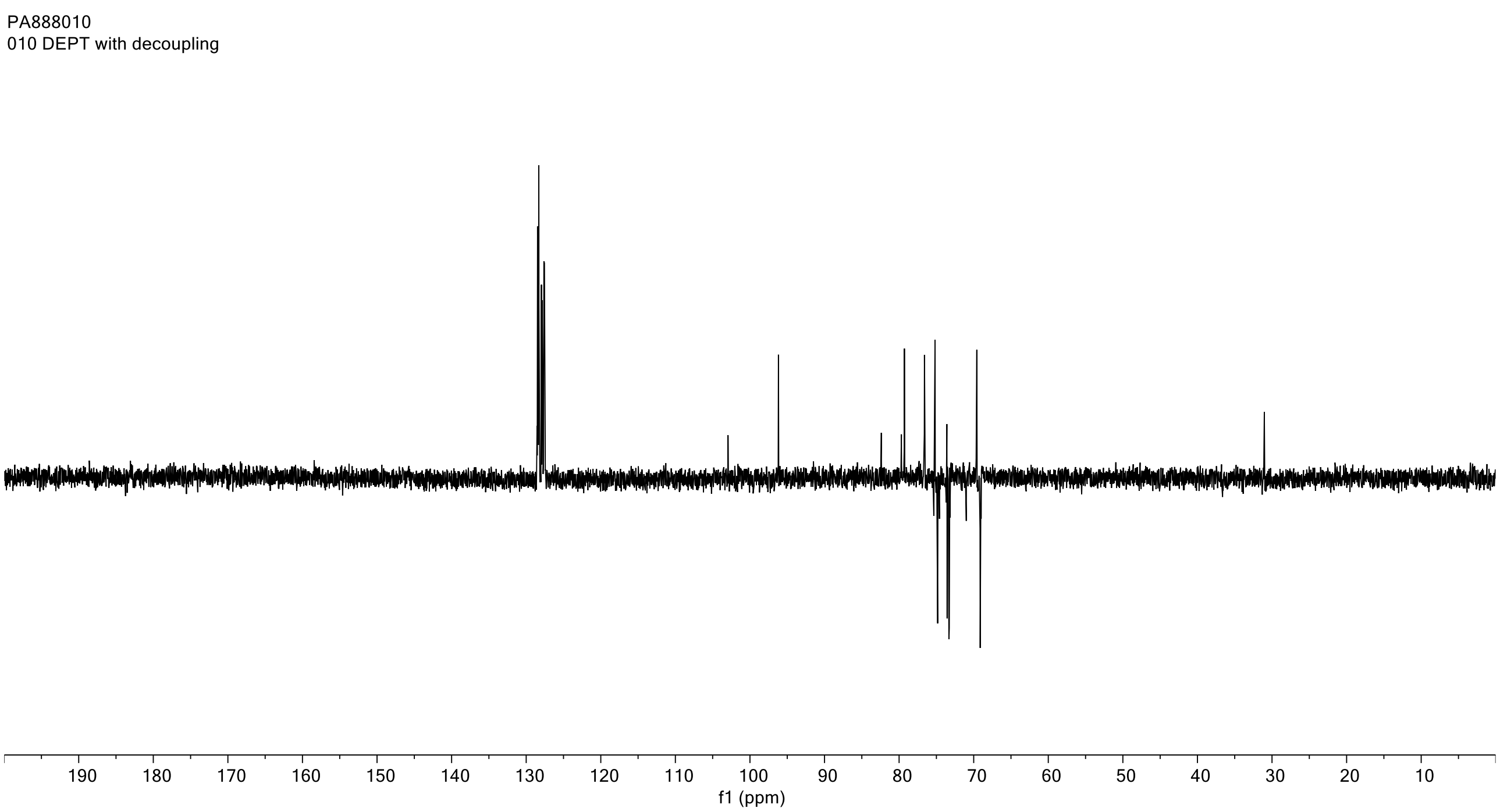

ESI-MS of **GalOBn**:

**Sample Name**	010	**Position**		**Instrument Name**	Instrument 1
**User Name**		**Inj Vol**	0	**InjPosition**	
**Sample Type**	Sample	**IRM Calibration Status**	Success	**Data Filename**	PA888_42900.d
**ACQ Method**	MS-Service.m	**Comment**	in DCM/MeOH	**Acquired Time**	10/12/2023 12:24:30 PM (UTC+02:00)

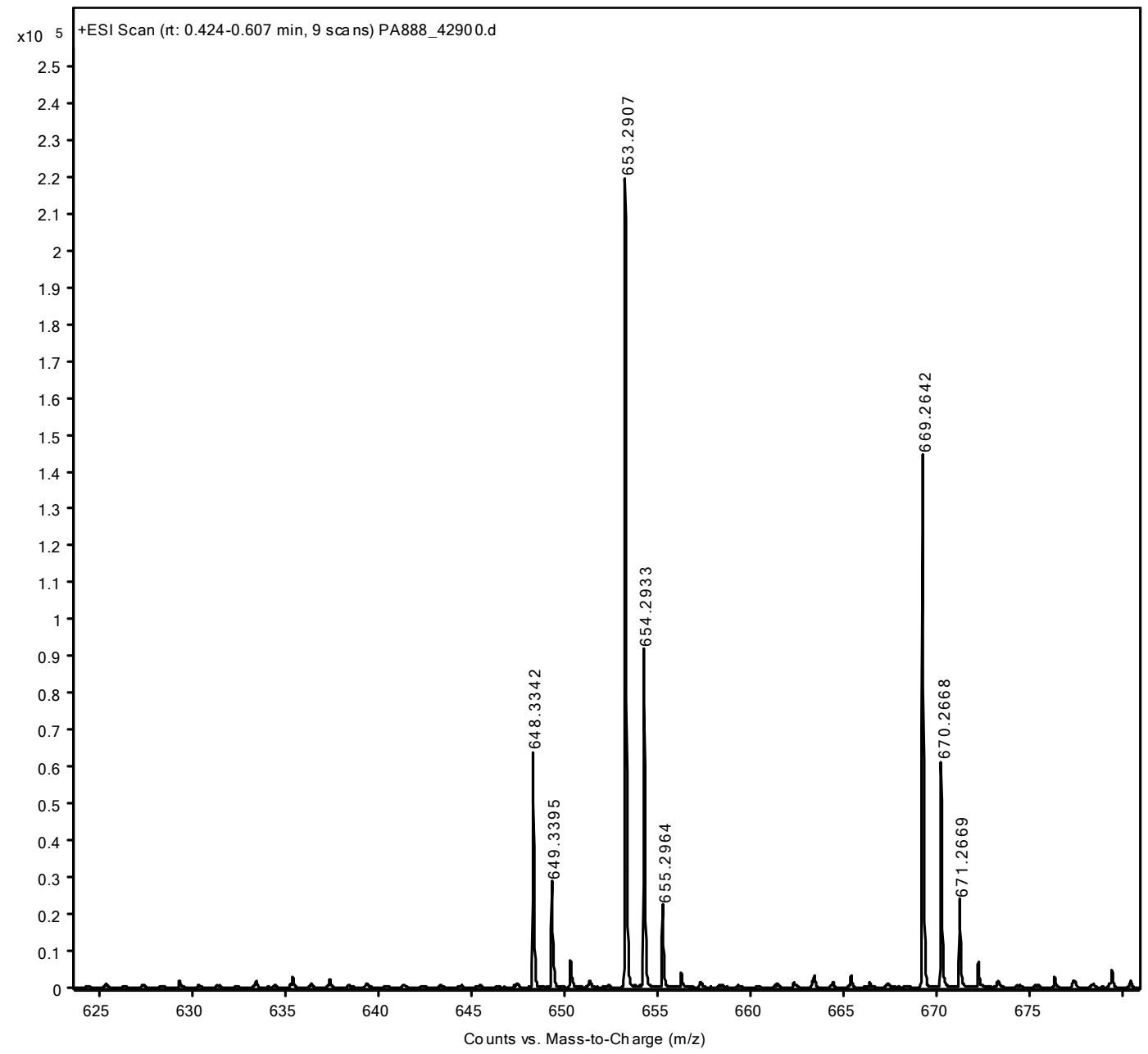

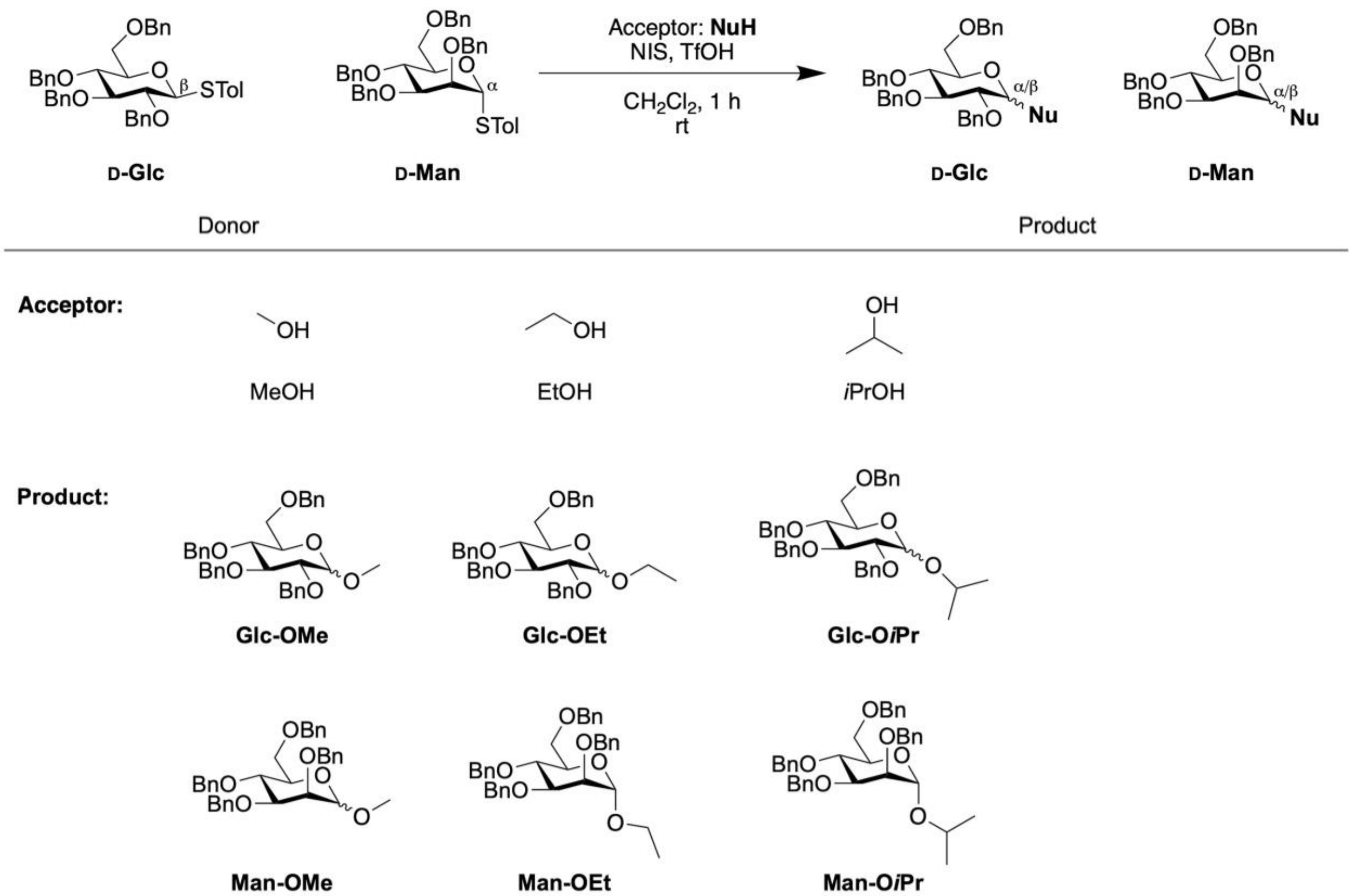

The donor (64.4 mg, 0.100 mmol, 1.0 eq.) was added and dried in a flask. Afterward, NIS (33.7 mg, 0.150 mmol, 1.5 eq.) was added, and the reaction mixture was dissolved in dry CH_2Cl_2 (1 mL). The acceptor (1.5 eq.) was added. One minute later, TfOH (17.5 µL, 0.200 mmol, 0.20 eq.) was added, and the reaction mixture was stirred for 1 h at room temperature. The reaction was monitored by TLC (SiO_2, Hex/EtOAc), quenched with Et_3N, and concentrated under reduced pressure. The crude product was purified by column chromatography (MPLC, SiO_2, Hex/EtOAC) and characterized by NMR. The product could be obtained as α and β isomers.

The NMR and mass spectral data of the products Glc-OMe and Man-OMe are consistent with those reported in the literature.[11,43]

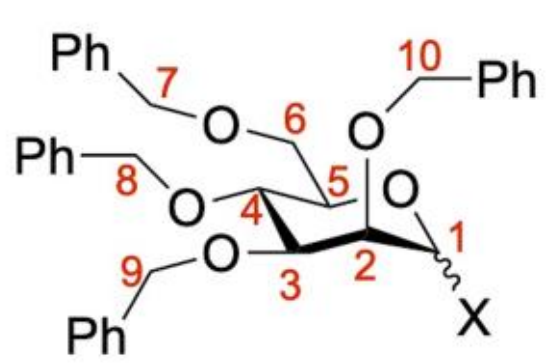

Ethyl 2,3,4,6-Tetra-O-benzyl-D-glucopyranoside

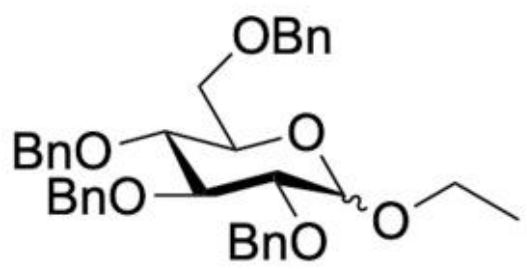

Glc-OEt

***a*-anomer of Glc-OEt:**

^{1}H NMR (500 MHz, $CDCl_3$) δ 7.39 – 7.21 (m, 20H, Ph-H), 5.00 (d, *J* = 10.8 Hz, 1H, H-9a), 4.83 (d, *J* = 10.7 Hz, 1H, H-8a), 4.82 (d, *J* = 10.6 Hz ,1H, H-9b), 4.80 (d, *J* = 12.1 Hz ,1H, H-10a), 4.75 (d, *J* = 3.6 Hz, 1H, H-1), 4.65 (d, *J* = 12.2 Hz, 1H, H-10b), 4.61 (d, *J* = 12.2 Hz, 1H, H-7a), 4.47 (d, *J* = 12.1 Hz, 1H, H-7b), 4.45 (d, *J* = 10.7 Hz, 1H, H-8b), 4.00 (dd, *J* = 9.7, 8.9 Hz, 1H, H-3), 3.78 (ddd, *J* = 9.9, 3.5, 2.0 Hz, 1H, H-5), 3.74 – 3.71 (m, 1H, H-6a), 3.71 – 3.66 (m, 1H, GlcOC*H*H), 3.66 – 3.63 (m, 1H, H-4), 3.63 – 3.60 (m, 1H, H-6b), 3.55 (dd, *J* = 9.7, 3.6 Hz, 1H, H-2), 3.53 – 3.49 (m, 1H, GlcOCH*H*), 1.31 – 1.19 (t, 3H, CH_3). **^{13}C NMR (176 MHz, $CDCl_3$)** δ 139.1 (Ph-C), 138.5 (Ph-C), 138.4 (Ph-C), 138.1 (Ph-C), 128.6 (Ph-C), 128.5 (Ph-C), 128.5 (Ph-C), 128.2 (Ph-C), 128.1 (Ph-C), 128.0 (Ph-C), 128.0 (Ph-C), 127.8 (Ph-C), 127.8 (Ph-C), 127.7 (Ph-C), 96.7 (C-1), 82.3 (C-3), 80.1 (C-2), 77.9 (C-4), 75.8 (C-9), 75.2 (C-8), 73.6 (C-7), 73.4 (C-10), 70.2 (C-5), 68.7 (C-6), 63.4 (GlcO*C*HH), 31.1 (CH_3). **HRMS (ESI)** calcd for $C_{36}H_{40}O_6$ $[M+Na]^+$: 591.2723, found: 591.2747

^{1}H NMR (500 MHz, $CDCl_3$) of ***a*-anomer of Glc-OEt**:

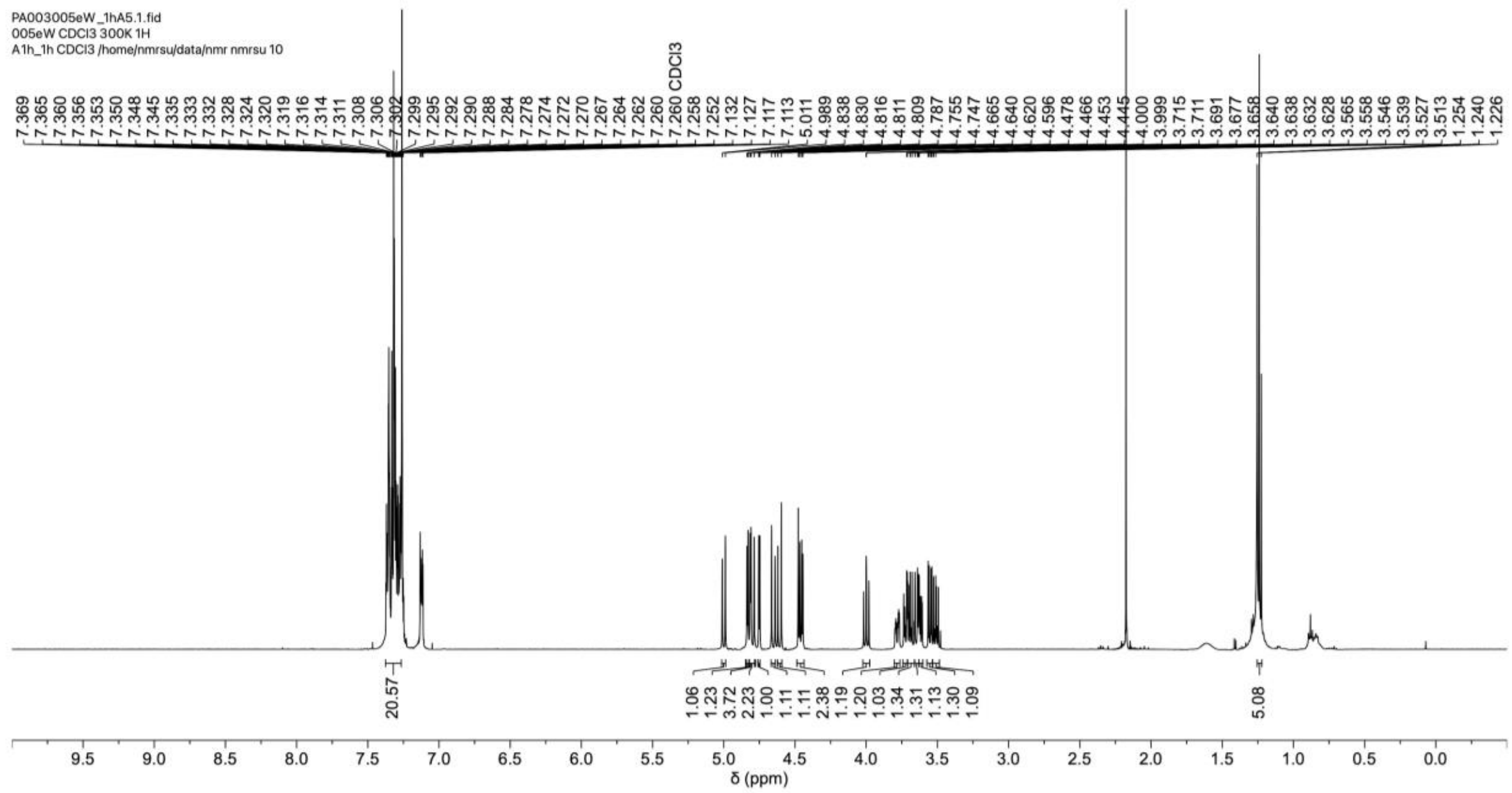

2D-HSQC (^{1}H, ^{13}C) (176 MHz, $CDCl_3$) of ***a*-anomer of Glc-OEt**:

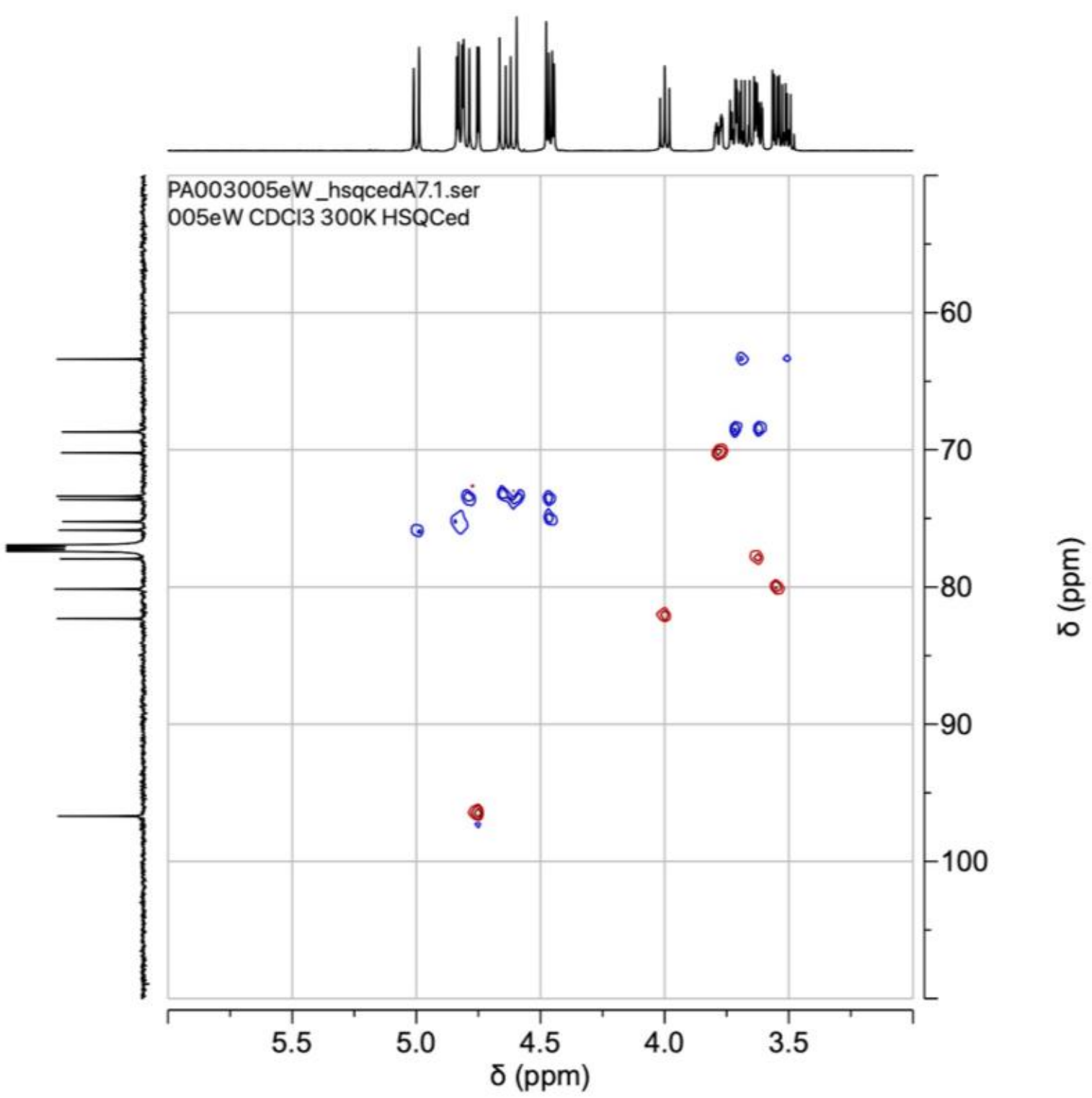

^{13}C NMR (176 MHz, $CDCl_3$) of ***a*-anomer of Glc-OEt**:

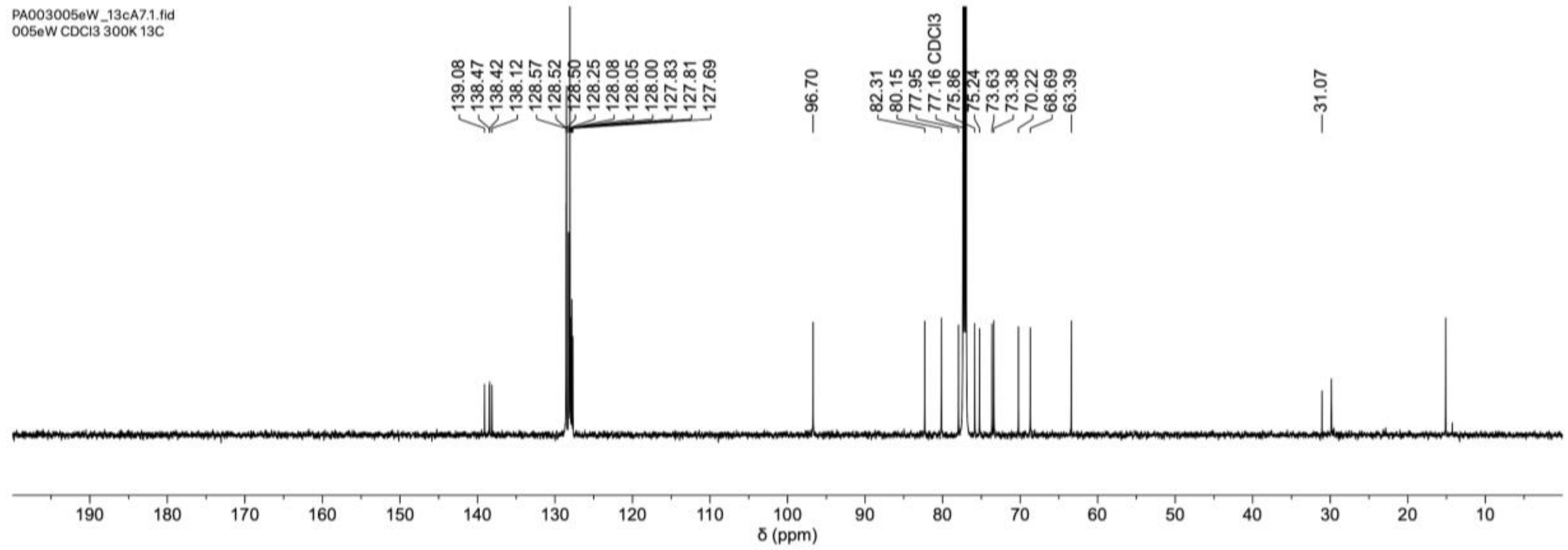

DEPT135 NMR (176 MHz, $CDCl_3$) of ***a*-anomer of Glc-OEt**:

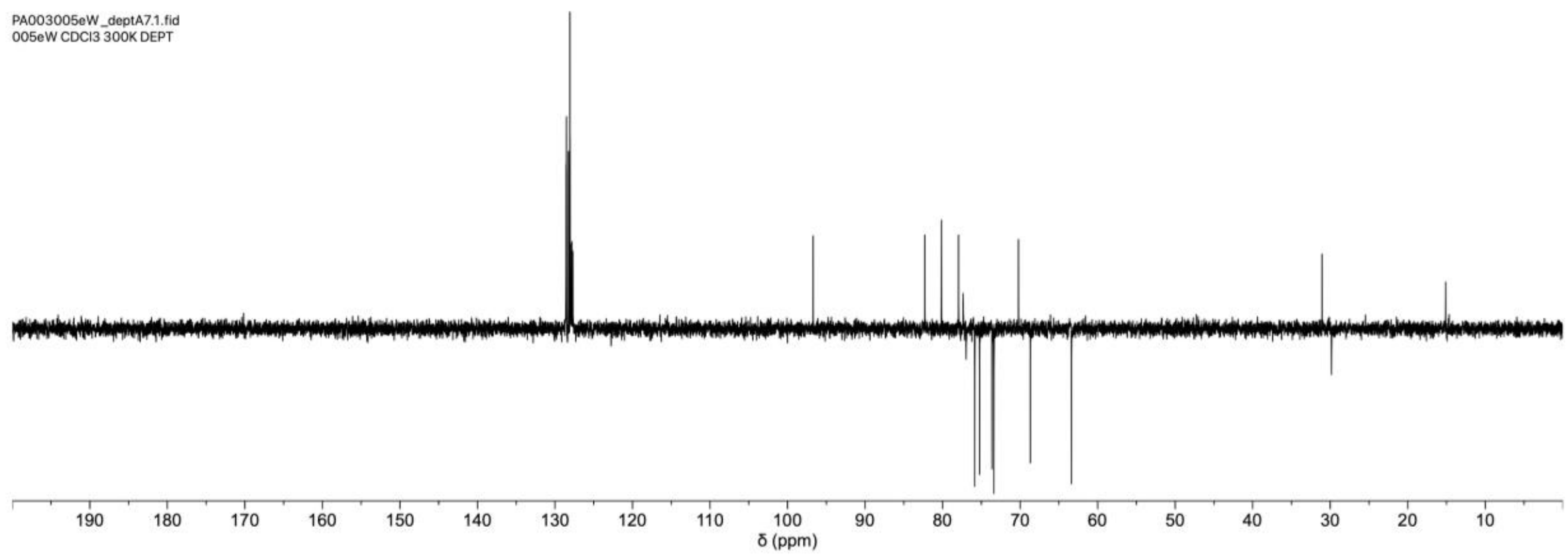

ESI-MS of ***α*-anomer of Glc-OEt**:

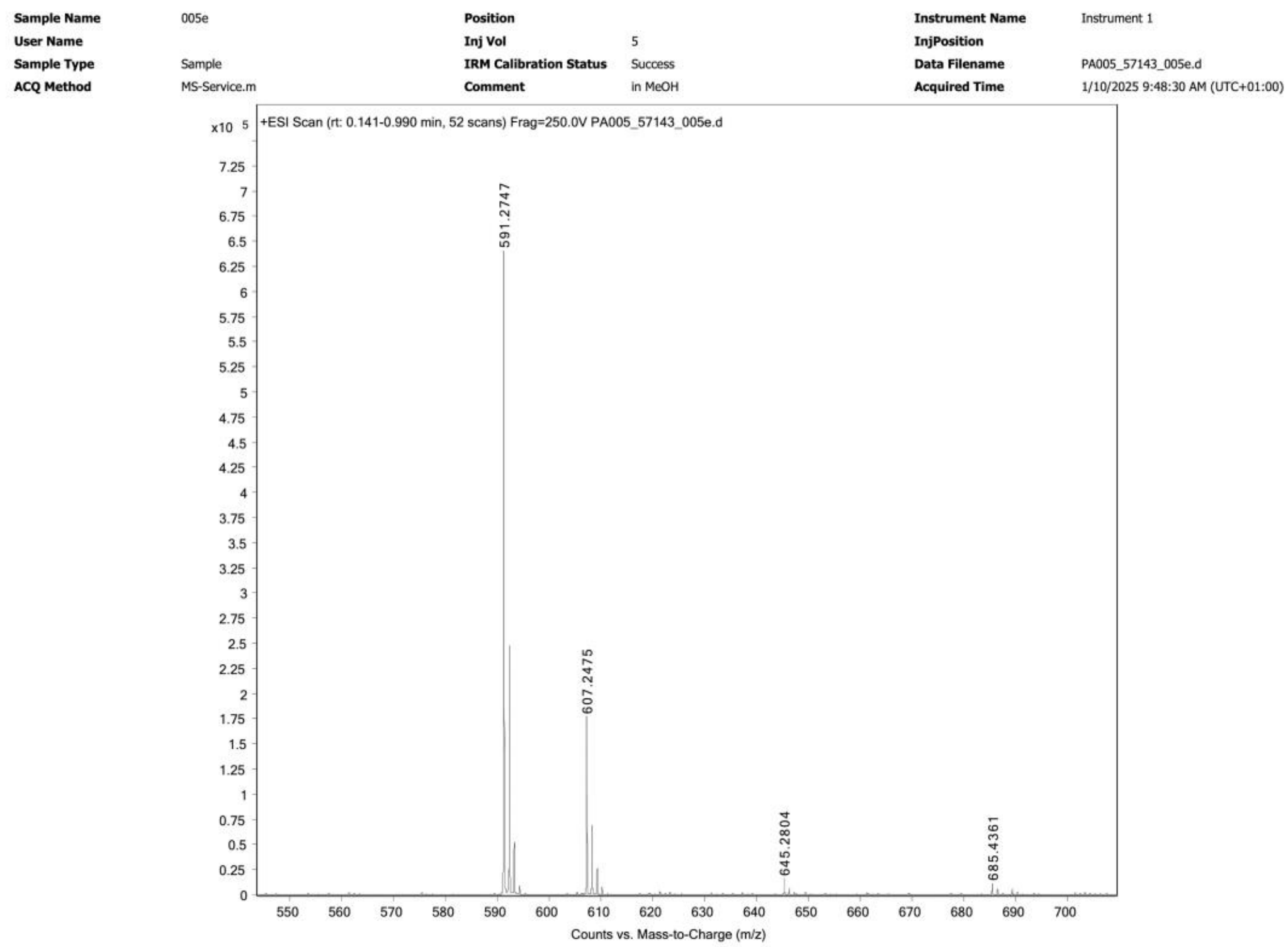

***β*-anomer of Glc-OEt:**

^{1}H NMR (500 MHz, $CDCl_3$) δ 7.39 – 7.22 (m, 20H, Ph-H), 4.96 (d, *J* = 10.9 Hz, 1H, H-10a), 4.93 (d, *J* = 11.0 Hz, 1H, H-9a), 4.82 (d, *J* = 10.8 Hz, 1H, H-8a), 4.79 (d, *J* = 10.9 Hz, 1H, H-9b), 4.72 (d, *J* = 10.9 Hz, 1H, H-10b), 4.62 (d, *J* = 12.2 Hz, 1H, H-7a), 4.56 (d, *J* = 12.2 Hz, 1H,H-7b), 4.52 (d, *J* = 10.8 Hz, 1H, H-8b), 4.41 (d, *J* = 7.8 Hz, 1H, H-1), 4.02 (dq, *J* = 9.5, 7.1 Hz, 1H, GlcOC*H*H), 3.75 (dd, *J* = 10.7, 2.0 Hz, 1H, H-6a), 3.69 – 3.66 (m, 1H, H-6b), 3.66 – 3.60 (m, 2H, H-3, GlcOCH*H*), 3.58 (dd, *J* = 9.7, 8.9 Hz, 1H, H-4), 3.49 – 3.46 (m, 1H, H-5), 3.47 – 3.43 (m, 1H, H-2), 1.30 (t, *J* = 7.1 Hz, 3H, CH_3). **^{13}C NMR (176 MHz, $CDCl_3$)** δ 138.99 (Ph-C), 138.9 (Ph-C), 138.5 (Ph-C), 138.4 (Ph-C), 128.7 (Ph-C), 128.7 (Ph-C), 128.7 (Ph-C), 128.5 (Ph-C), 128.3 (Ph-C), 128.2 (Ph-C), 128.1 (Ph-C), 128.1 (Ph-C), 128.0 (Ph-C), 127.9 (Ph-C), 103.8 (C-1), 85.0 (C-3), 82.6 (C-2), 78.3 (C-4), 76.0 (C-9), 75.3 (C-8), 75.2 (C-5), 75.1 (C-10), 73.8 (C-7), 69.4 (C-6), 65.9 (GlcO*C*HH), 31.2 (CH_3). **HRMS (ESI)** calcd for $C_{36}H_{40}O_6$ $[M+Na]^+$: 591.2723, found: 591.2744

^{1}H NMR (500 MHz, $CDCl_3$) of ***β-anomer* of Glc-OEt**:

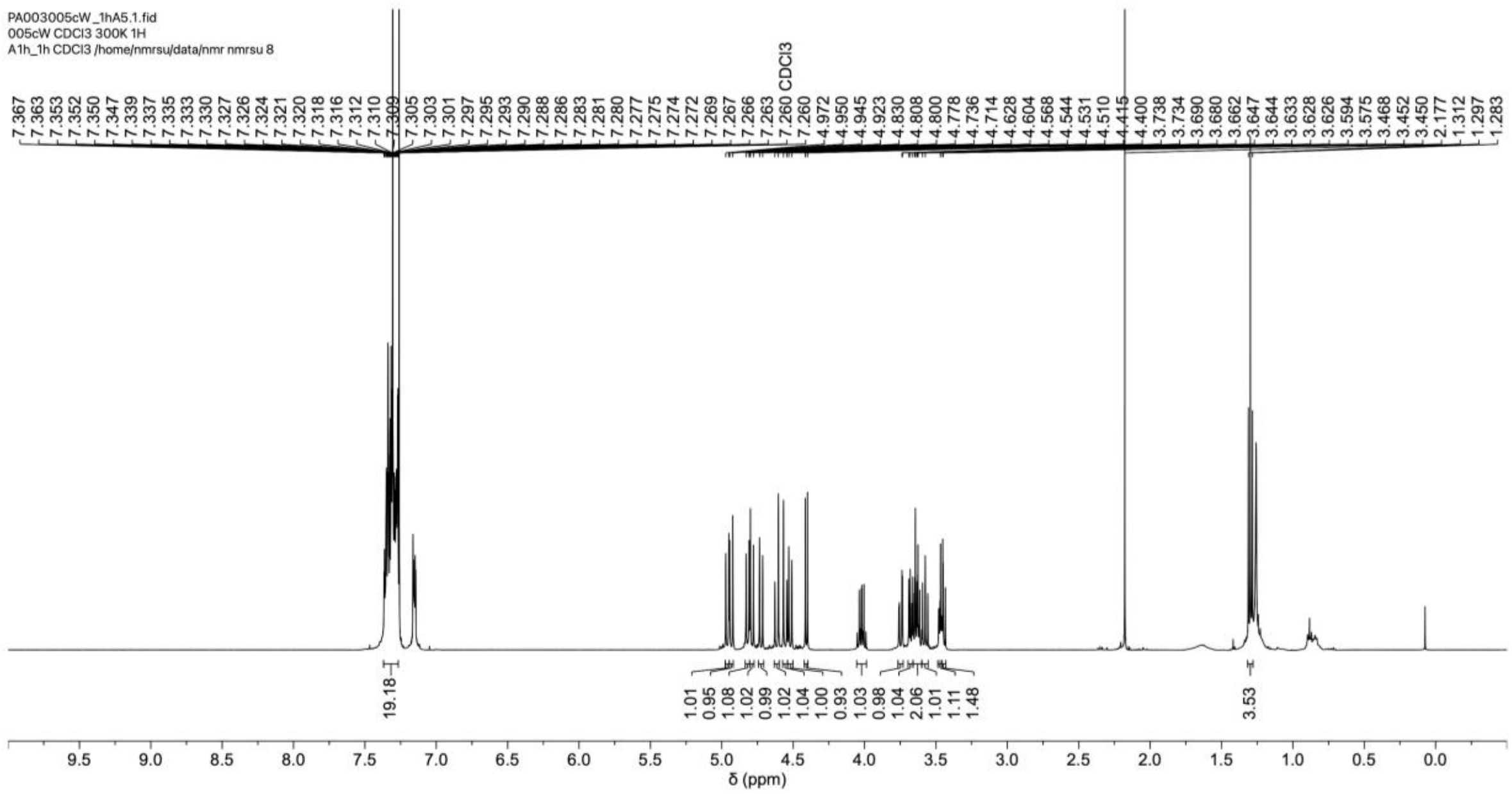

2D-HSQC (^{1}H, ^{13}C) (176 MHz, $CDCl_3$) of ***β*-anomer of Glc-OEt**:

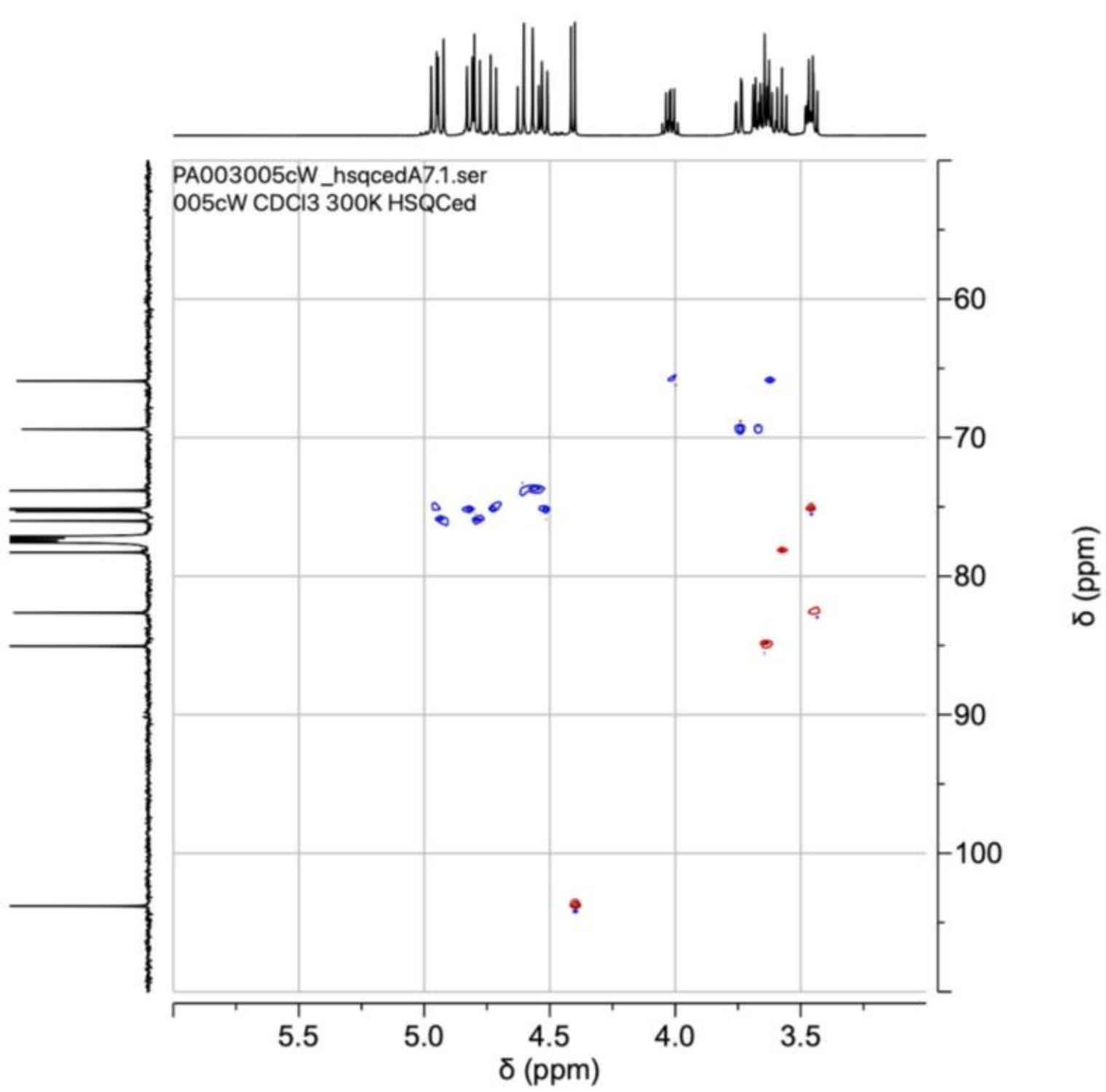

^{13}C NMR (176 MHz, $CDCl_3$) of ***β-anomer* of Glc-OEt**:

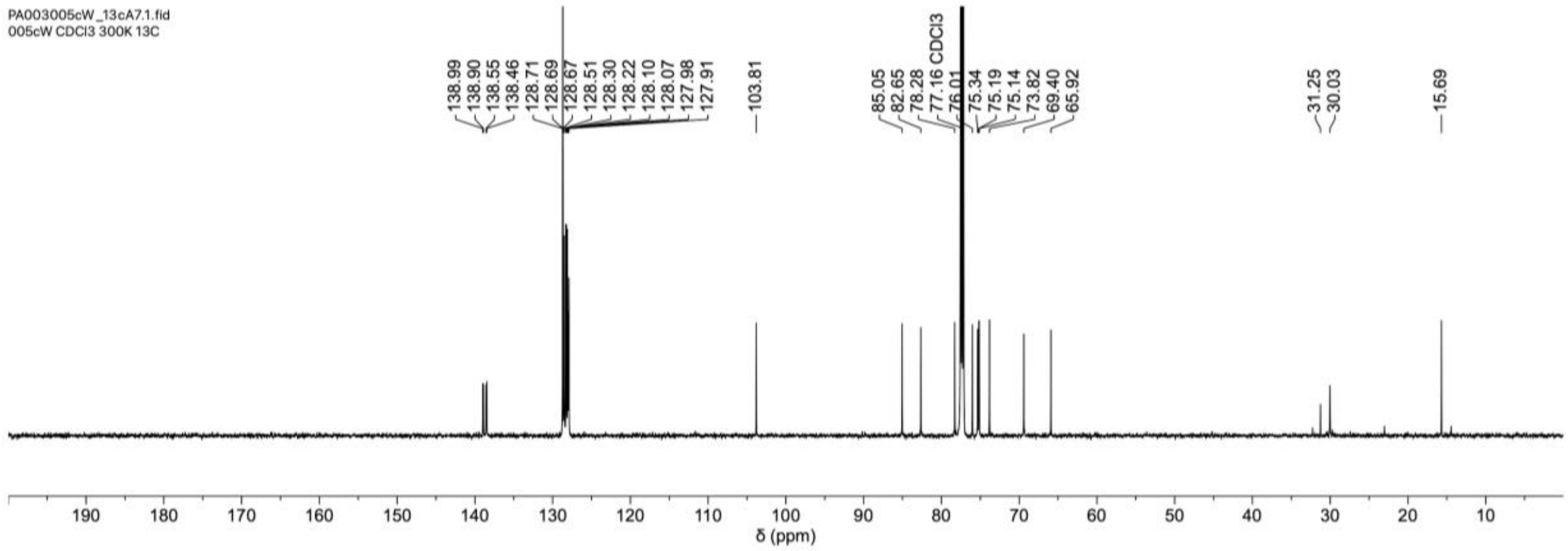

DEPT135 NMR (176 MHz, $CDCl_3$) of ***β*-anomer of Glc-OEt**:

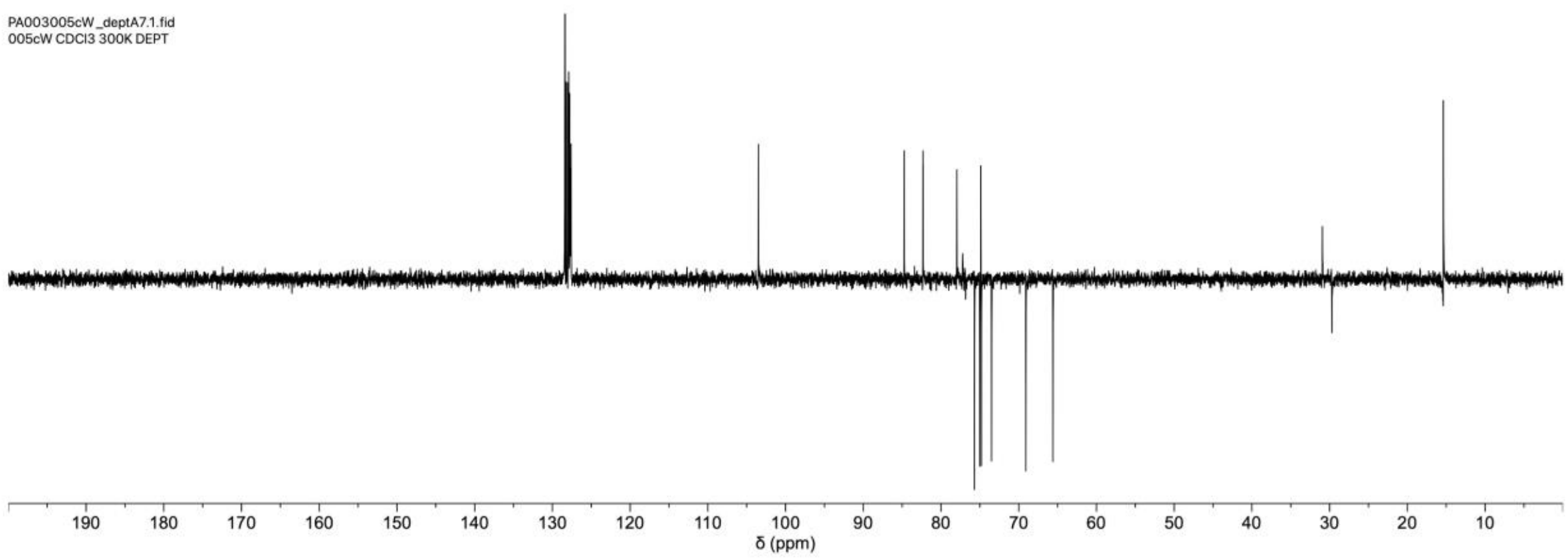

ESI-MS of ***β*-anomer of Glc-OEt**:

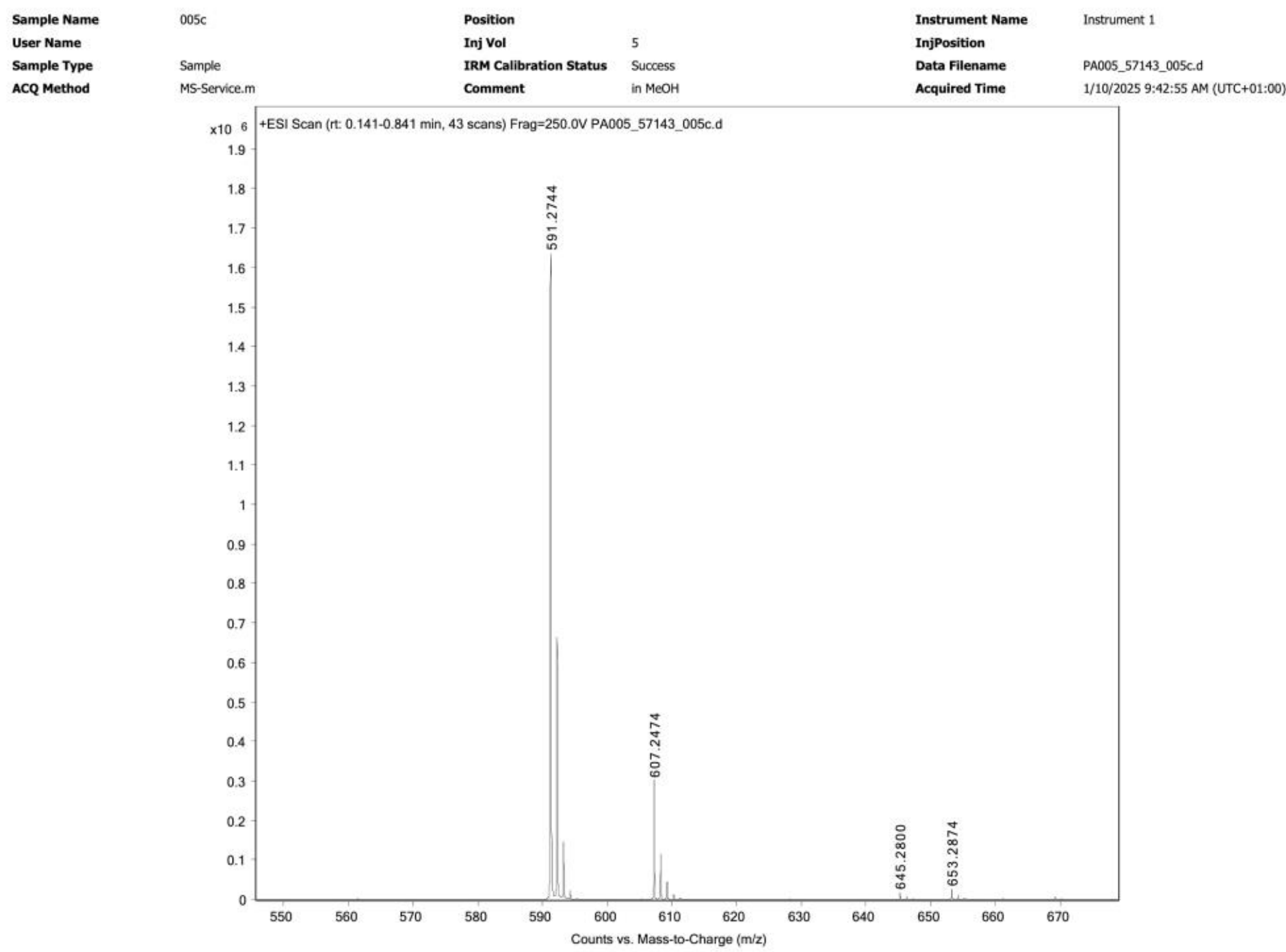

iso-Propyl 2,3,4,6-Tetra-O-benzyl-D-glucopyranoside

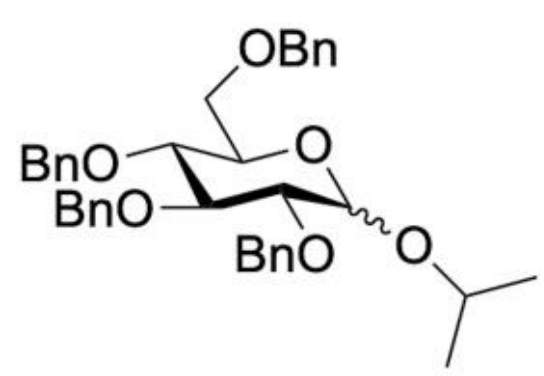

Glc-O*i*Pr

***a*-anomer of Glc-OPr:**

^{1}H NMR (700 MHz, $CDCl_3$) δ 7.42 – 7.26 (m, 18H, Ph-H), 7.19 – 7.11 (m, 2H, Ph-H), 5.00 (d, *J* = 10.8 Hz, 1H, H-9a), 4.88 (d, *J* = 3.7 Hz, 1H, H-1), 4.82 (t, *J* = 10.6 Hz, 2H, H-9b, H-8a), 4.77 (d, *J* = 11.9 Hz, 1H, H-10a), 4.65 (d, *J* = 12.0 Hz, 1H, H-10b), 4.61 (d, *J* = 12.1 Hz, 1H, H-7a), 4.47 (d, *J* = 10.6 Hz, 1H, H-8b), 4.47 (d, *J* = 12.3, 1H, H-7b), 4.01 – 3.97 (m, 1H, H-3), 3.90 (h, *J* = 6.2 Hz, 1H, GlcOC*H*), 3.84 (ddd, *J* = 10.0, 3.7, 2.1 Hz, 1H, H-5), 3.73 (dd, *J* = 10.6, 3.7 Hz, 1H, H-6a), 3.65 – 3.63 (m, 9.7, 9.3 Hz, 1H, H-4), 3.63 – 3.61 (m, 1H, H-6b), 3.55 (dd, *J* = 9.7, 3.7 Hz, 1H, H-2), 1.22 (d, *J* = 6.3 Hz, 3H, CH_3'), 1.18 (d, *J* = 6.1 Hz, 3H, CH_3''). **^{13}C NMR (176 MHz, $CDCl_3$)** δ 138.9 (Ph-C), 138.2 (Ph-C), 128.4 (Ph-C), 128.3 (Ph-C), 128.3 (Ph-C), 128.1 (Ph-C), 127.9 (Ph-C), 127.9 (Ph-C), 127.7 (Ph-C), 127.6 (Ph-C), 127.5 (Ph-C), 94.7 (C-1), 82.1

(C-3), 79.9 (C-2), 77.9 (C-4), 75.6 (C-9), 75.1 (C-8), 73.4 (C-7), 73.1 (C-10), 70.0 (C-5), 69.0 (GlcO*C*H), 68.5 (C-6), 23.1 (CH$_3$'), 22.9 (CH$_3$''). **HRMS (ESI)** calcd for $C_{37}H_{42}O_6$ [M+Na]$^+$: 605.2879, found: 605.2909

^{1}H NMR (700 MHz, CDCl$_3$) of ***a*-anomer of Glc-OPr**:

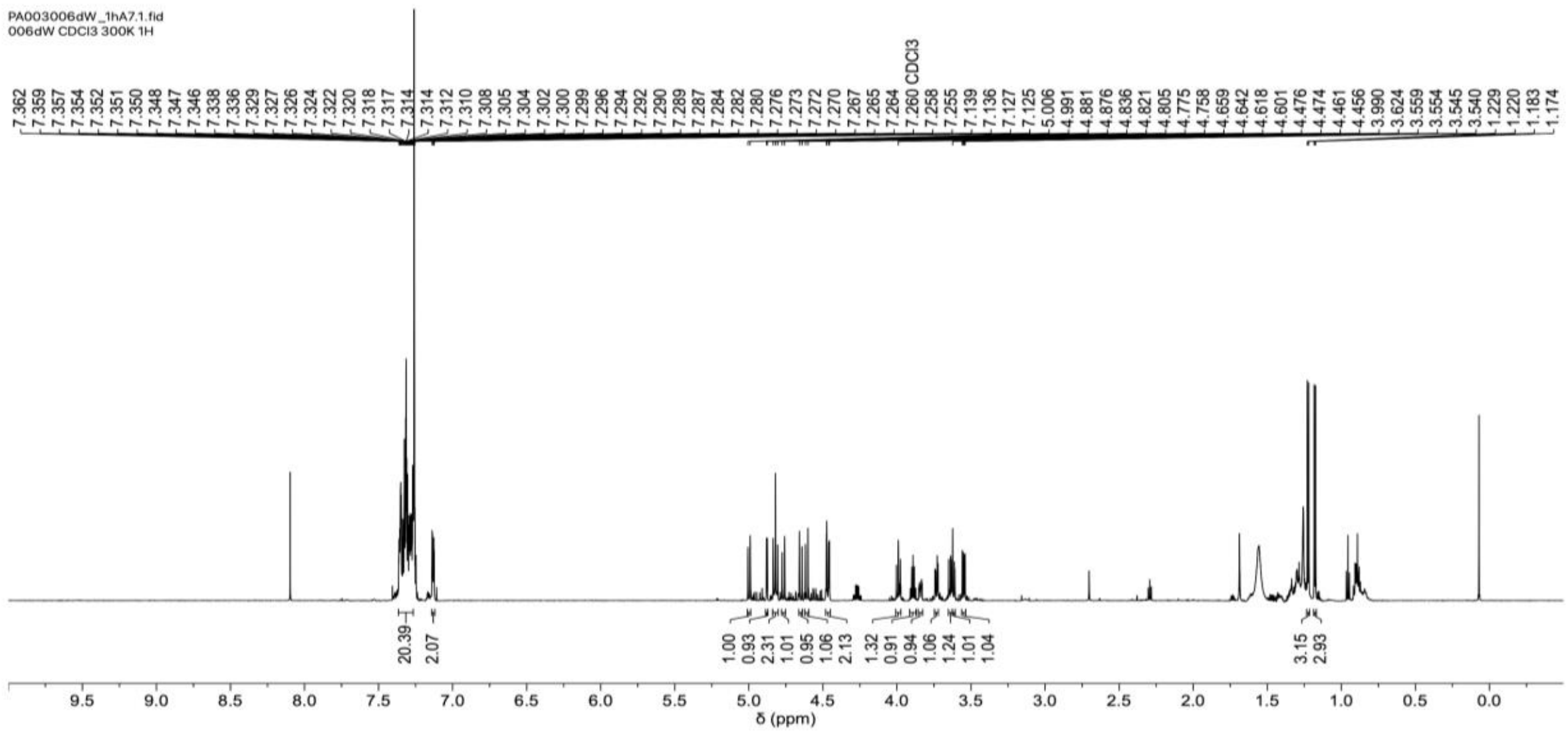

2D-HSQC (^{1}H, ^{13}C) (151 MHz, CDCl$_3$) of ***a*-anomer of Glc-OPr**:

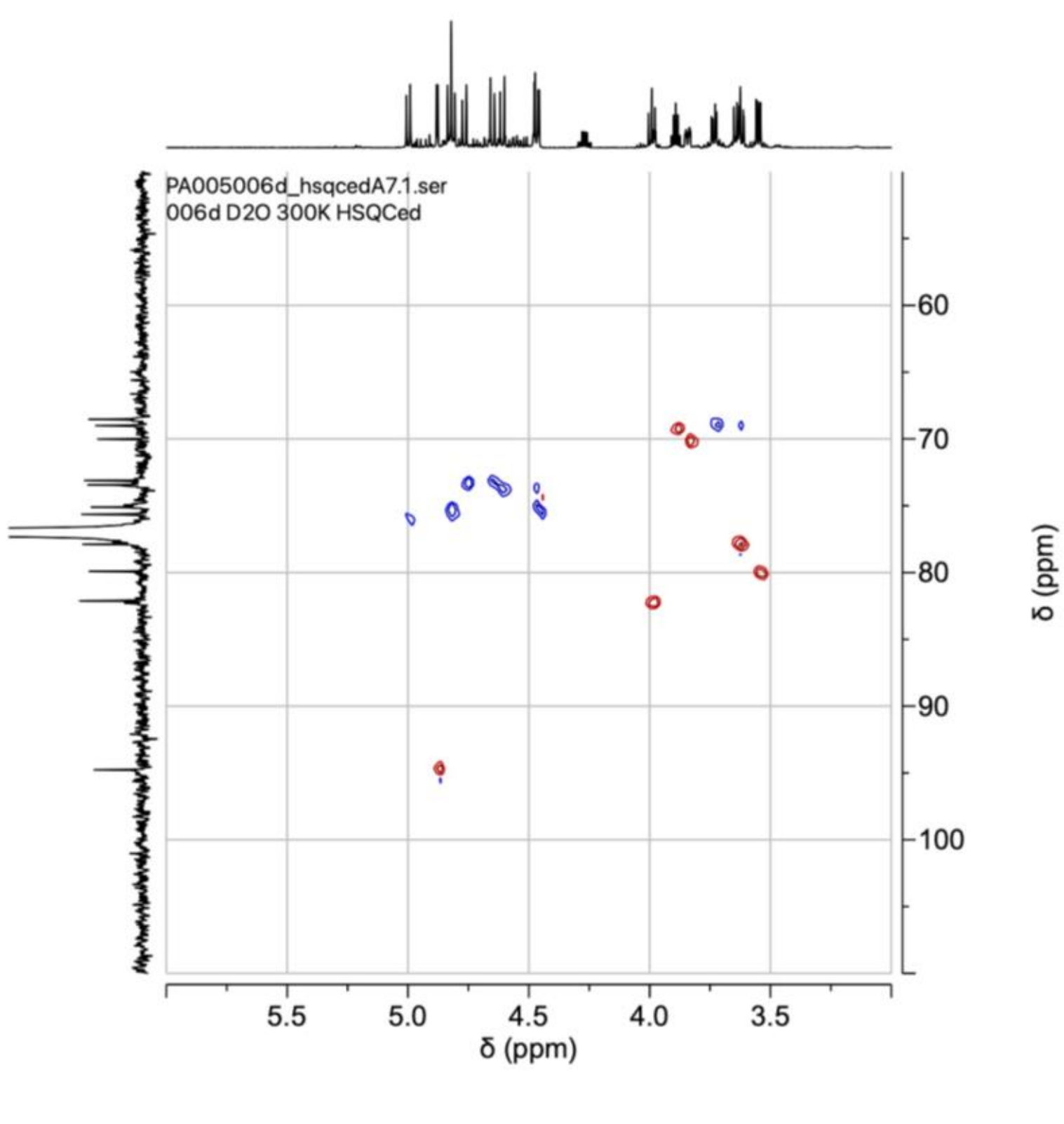

^{13}C NMR (151 MHz, $CDCl_3$) of ***a*-anomer of Glc-OPr**:

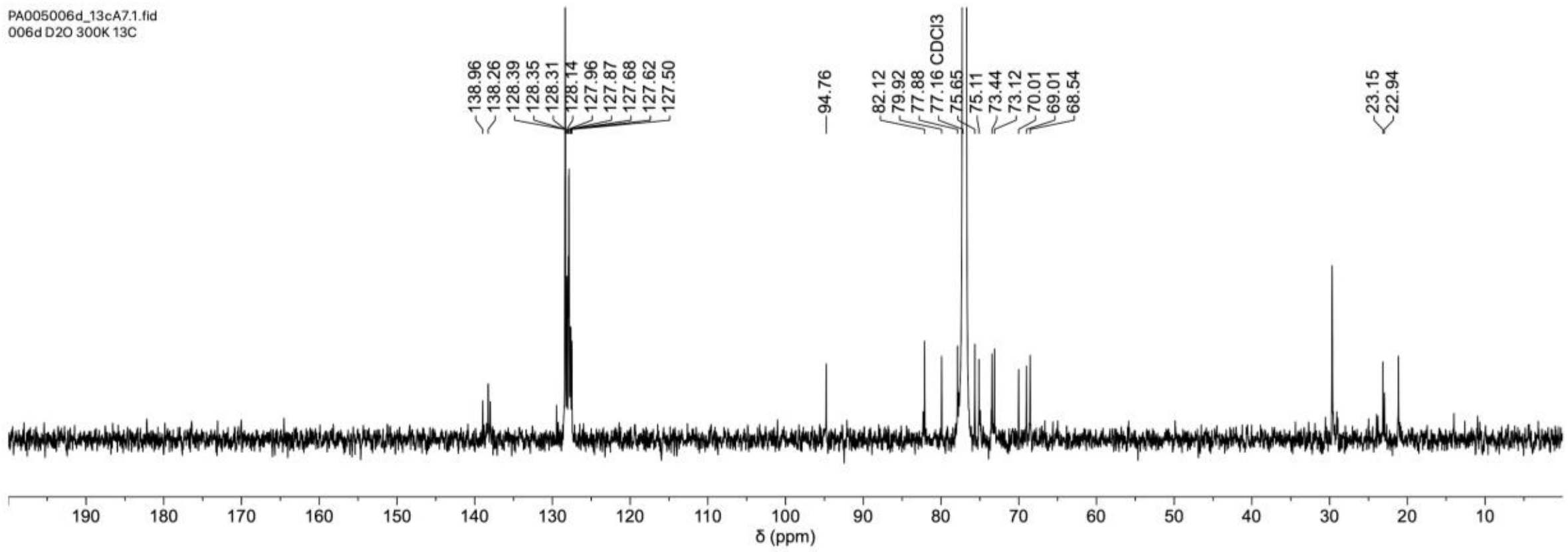

DEPT135 NMR (151 MHz, $CDCl_3$) of ***a*-anomer of Glc-OPr**:

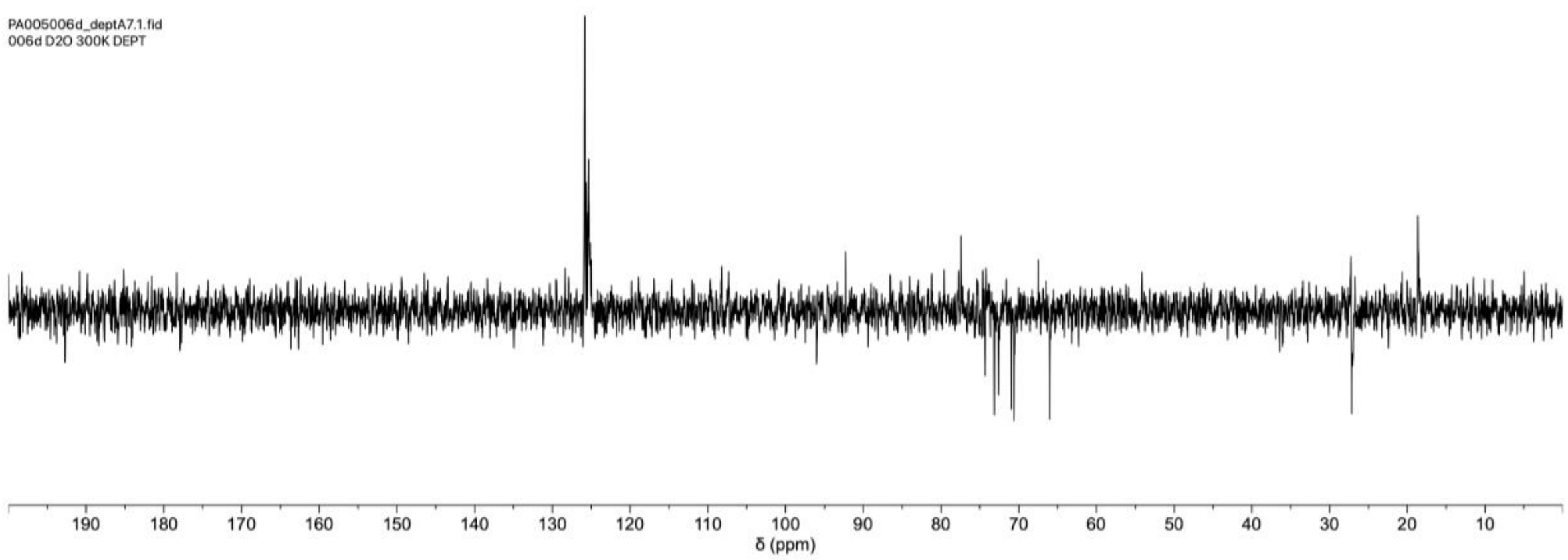

ESI-MS of ***a*-anomer of Glc-OPr**:

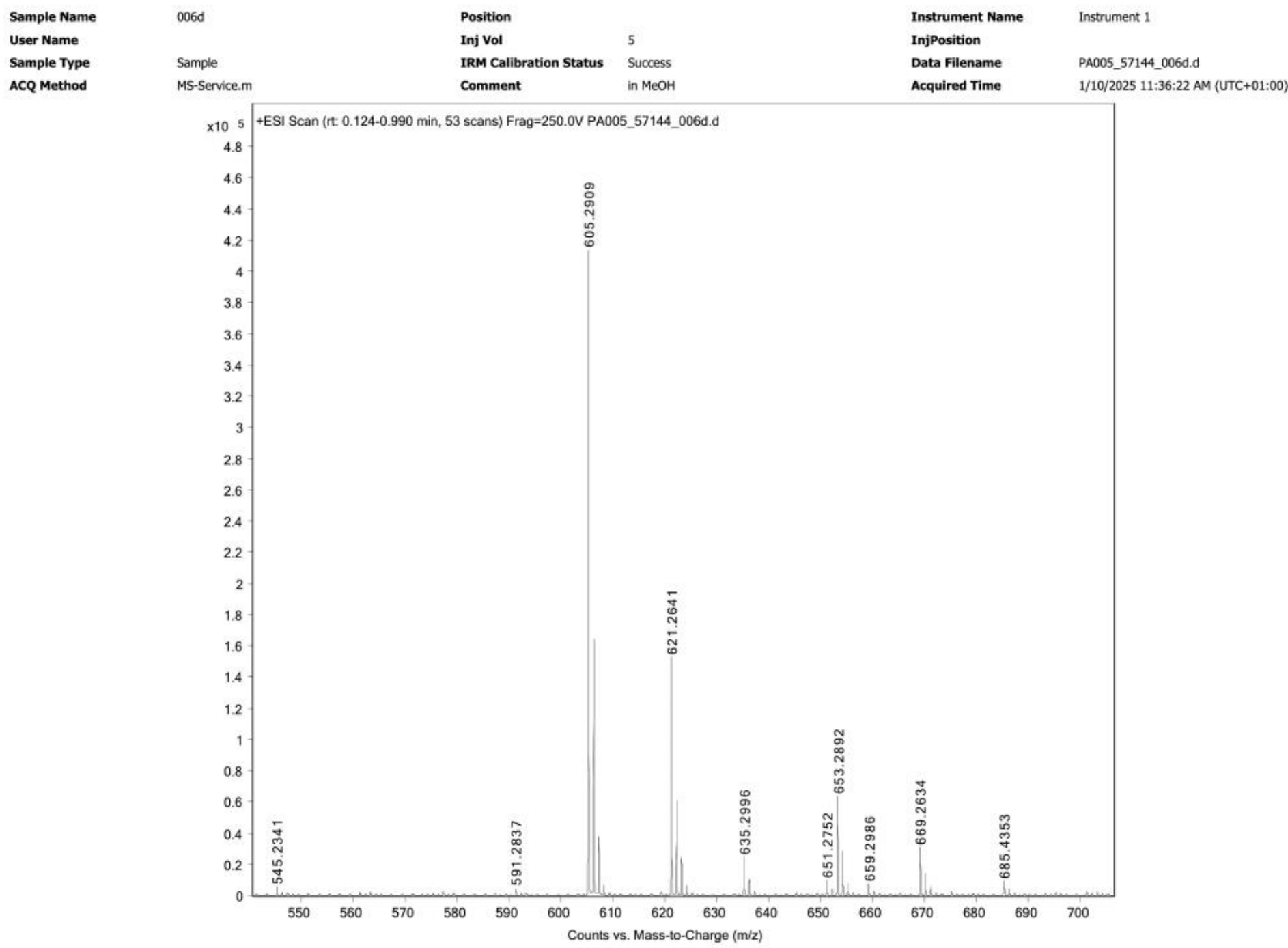

***β*-anomer of Glc-OPr:**

^{1}H NMR (700 MHz, CDCl$_3$) δ 7.37 – 7.26 (m, 18H, Ph-H), 7.19 – 7.15 (m, 2H, Ph-H), 4.97 (d, *J* = 10.9 Hz, 1H, H-10a), 4.92 (d, *J* = 10.9 Hz, 1H, H-9a), 4.82 (d, *J* = 10.8 Hz, 1H, H-8a), 4.78 (d, *J* = 11.0 Hz, 1H, H-9b), 4.71 (d, *J* = 10.9 Hz, 1H, H-10b), 4.61 (d, *J* = 12.3 Hz, 1H, H-7a), 4.57 – 4.55 (d, *J* = 12.3 Hz, 1H, H-7b), 4.54 (d, *J* = 10.9 Hz, 1H, H-8b), 4.47 (d, *J* = 7.8 Hz, 1H, H-1), 4.02 (dq, *J* = 12.4, 6.1 Hz, 1H, GlcOC*H*), 3.74 (dd, *J* = 10.8, 2.0 Hz, 1H, H-6a), 3.66 (dd, *J* = 10.8, 5.7 Hz, 1H, H-6b), 3.63 (t, *J* = 9.3 Hz, 1H, H-3), 3.57 – 3.53 (m, 1H, H-4), 3.46 (ddd, *J* = 9.8, 5.3, 2.0 Hz, 1H, H-5), 3.43 (dd, *J* = 9.2, 7.8 Hz, 1H, H-2), 1.32 (d, *J* = 6.3 Hz, 3H, CH$_3$'), 1.25 (d, *J* = 6.1 Hz, 3H, CH$_3$''). **^{13}C NMR (176 MHz, CDCl$_3$)** δ 139.0 (Ph-C), 138.9 (Ph-C), 138.6 (Ph-C), 138.5 (Ph-C), 128.7 (Ph-C), 128.5 (Ph-C), 128.3 (Ph-C), 128.2 (Ph-C), 128.0 (Ph-C), 128.0 (Ph-C), 128.0 (Ph-C), 127.9 (Ph-C), 127.7 (Ph-C), 102.5 (C-1), 85.2 (C-3), 82.7 (C-2), 78.3 (C-4), 76.0 (C-9), 75.3 (C-8), 75.2 (C-5, C-10), 73.8 (C-7), 72.7 (GlcO*C*H), 69.5 (C-6), 24.1 (CH$_3$'), 22.6 (CH$_3$''). **HRMS (ESI)** calcd for $C_{37}H_{42}O_6$ [M+Na]$^+$: 605.2879, found: 605.2908

^{1}H NMR (700 MHz, $CDCl_3$) of ***β-anomer*** **of GlcOPr**:

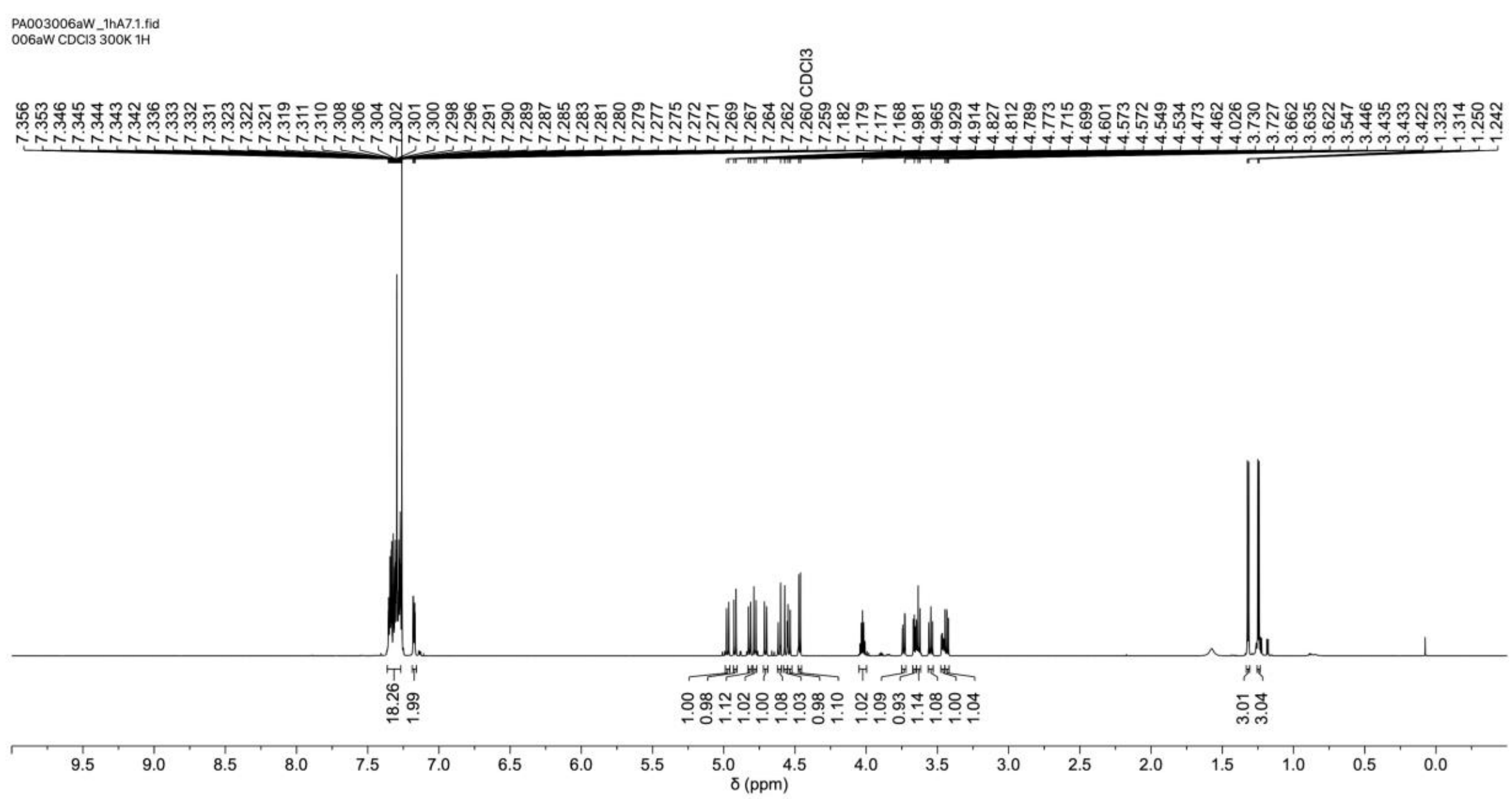

2D-HSQC (^{1}H, ^{13}C) (151 MHz, $CDCl_3$) of ***β*-anomer of GlcOPr**:

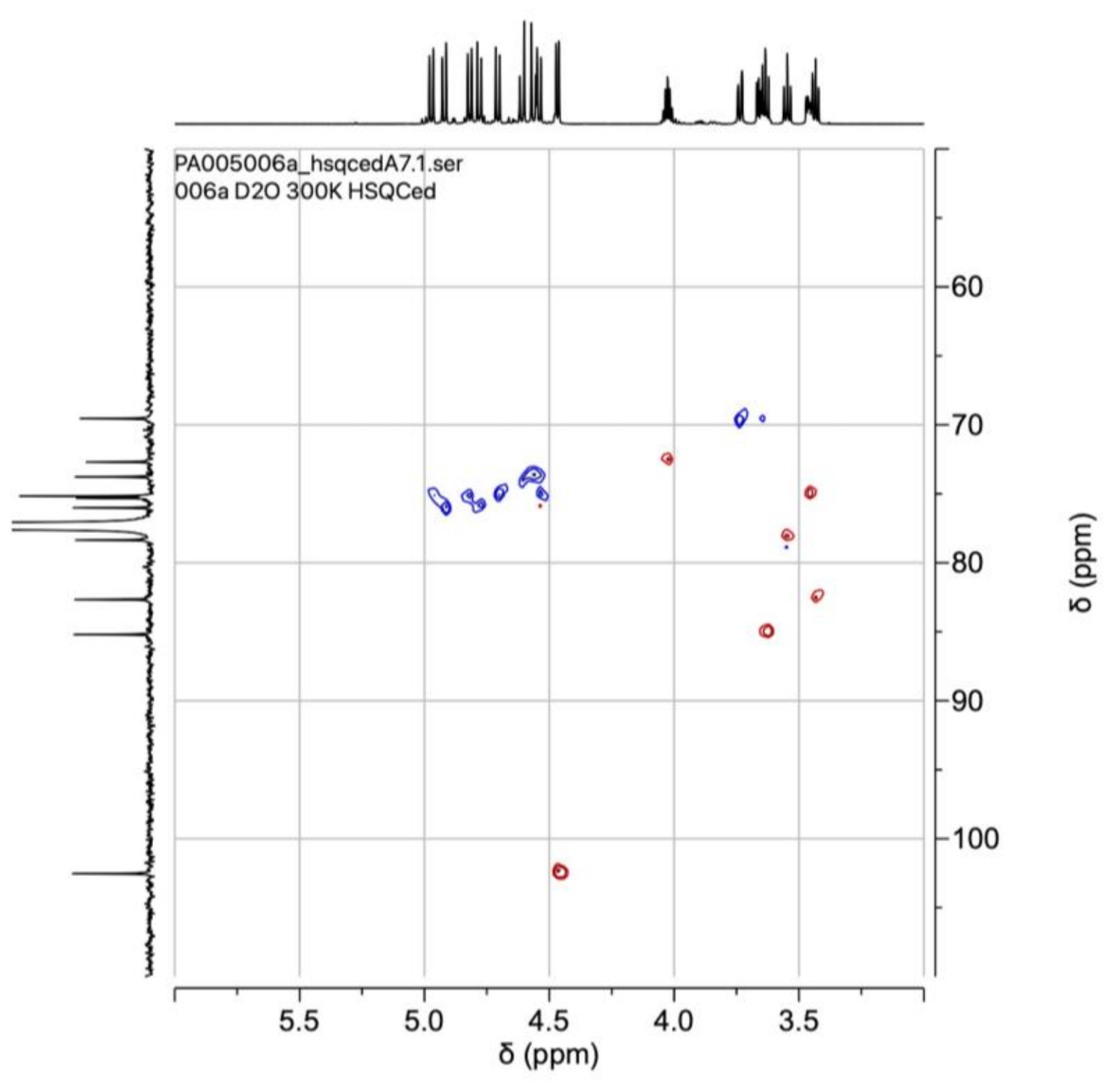

^{13}C NMR (151 MHz, $CDCl_3$) of ***β-anomer* of Glc-OPr**:

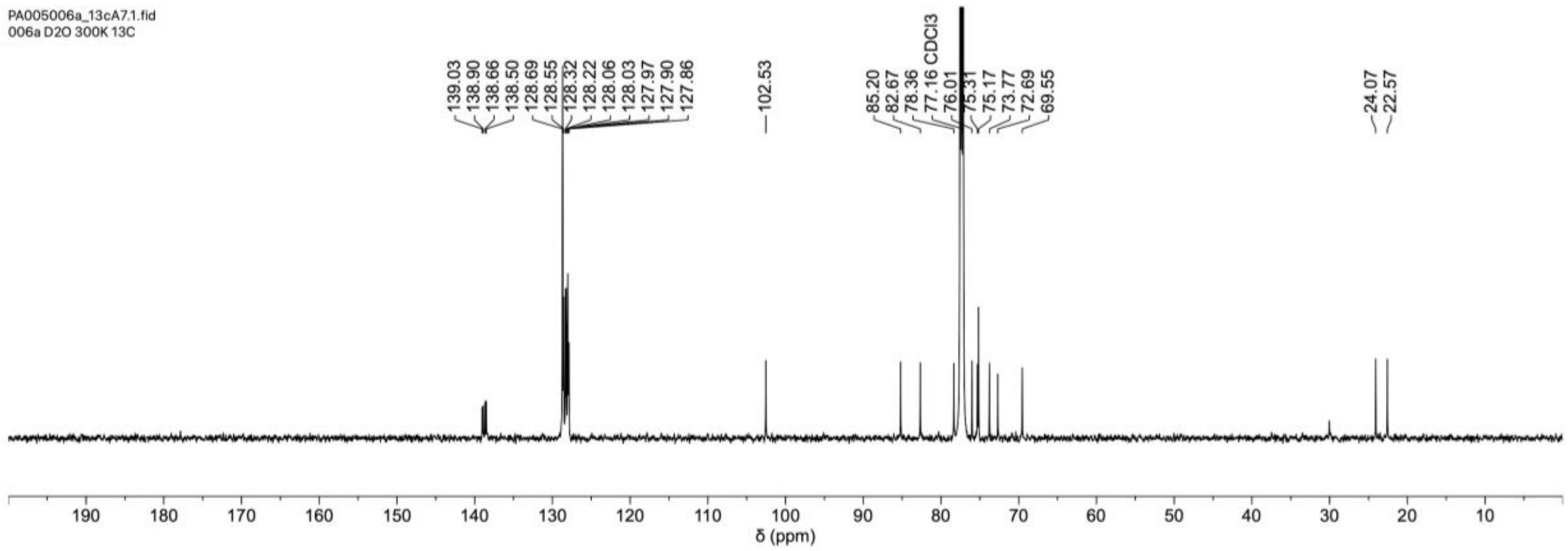

DEPT135 NMR (151 MHz, $CDCl_3$) of ***b*-anomer of Glc-OPr**:

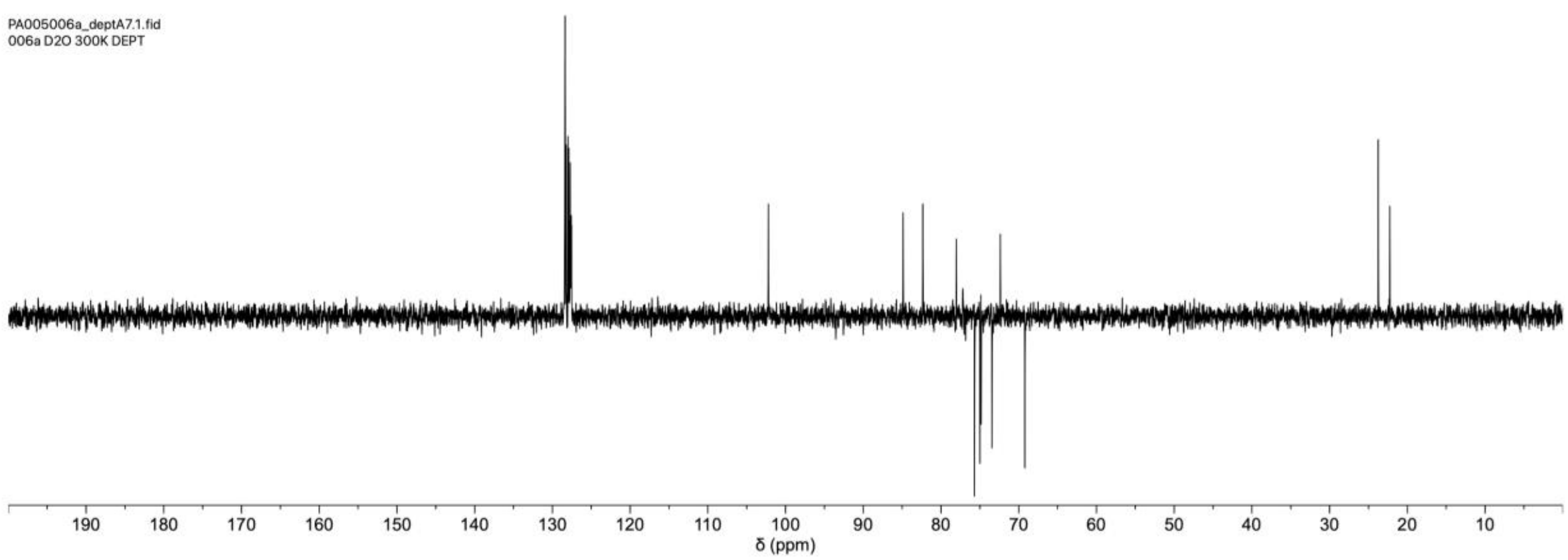

ESI-MS of ***β-anomer* of GlcOPr**:

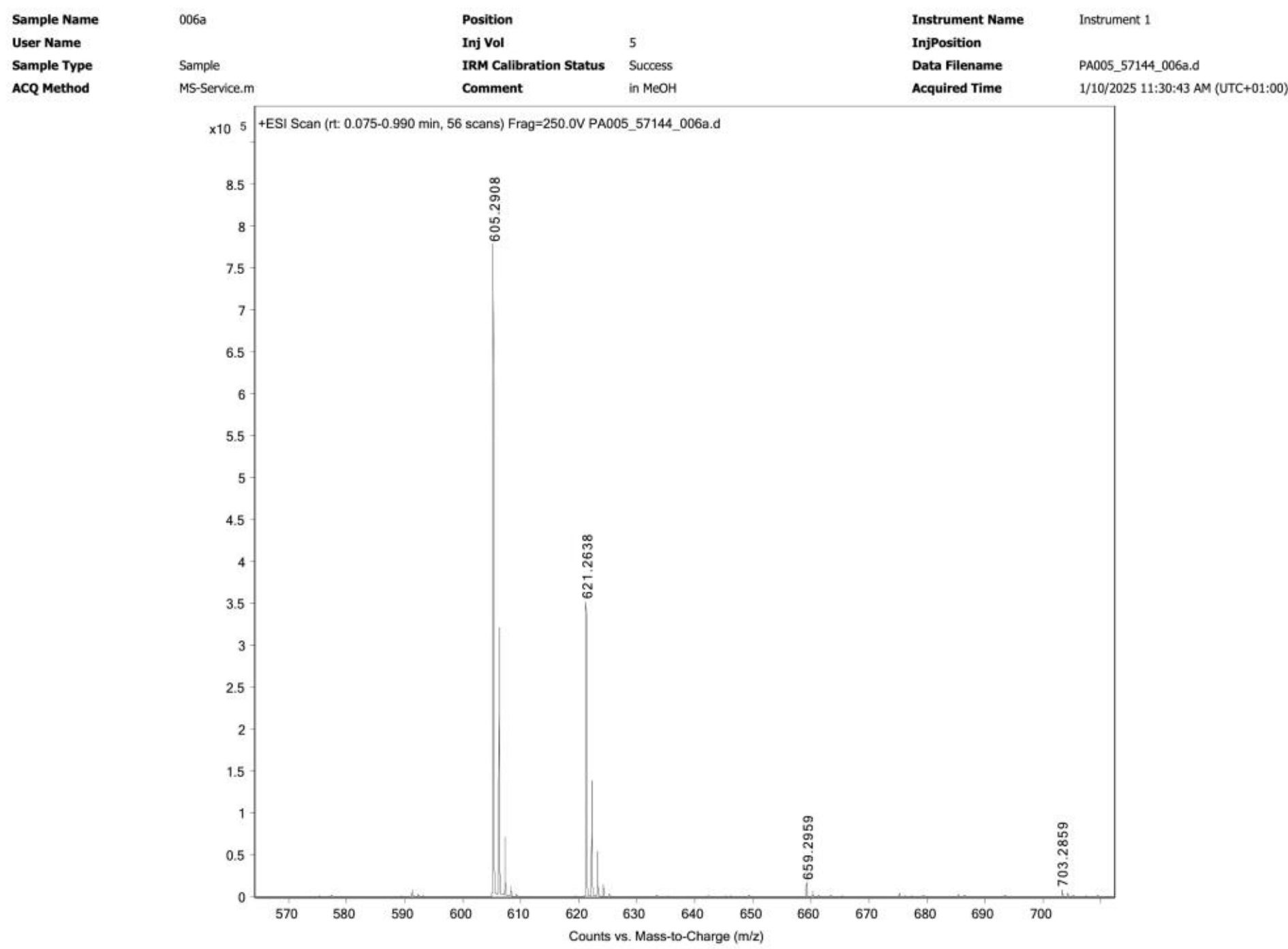

Ethyl 2,3,4,6-Tetra-O-benzyl-D-mannopyranoside

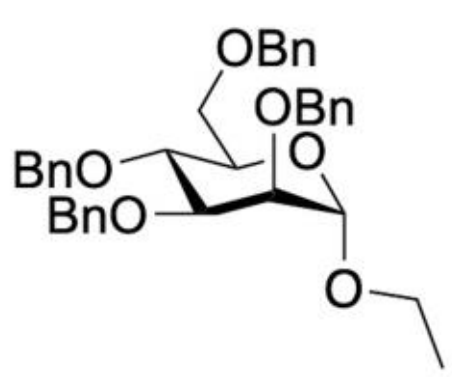

Man-OEt

***a*-anomer of Man-OEt:**

^{1}H NMR (700 MHz, CDCl$_3$) δ 7.40 – 7.24 (m, 18H, Ph-H), 7.18 – 7.14 (m, 2H, Ph-H), 4.89 (d, *J* = 1.9 Hz, 1H, H-1), 4.88 (d, *J* = 10.7 Hz, 1H, H-8a), 4.78 – 4.72 (m, 2H, H-10), 4.67 (d, *J* = 12.1 Hz, 1H, H-7a), 4.63 (s, 2H, H-9), 4.56 (d, *J* = 12.1 Hz, 1H, H-7b), 4.51 (d, *J* = 10.7 Hz, 1H, H-8b), 4.01 – 3.97 (m, 1H, H-4), 3.93 (dd, *J* = 9.4, 3.1 Hz, 1H, H-3), 3.80 (dd, *J* = 3.1, 1.9 Hz, 1H, H-2), 3.79 – 3.78 (m, 1H, H-6a), 3.78 – 3.77 (m, 1H, H-6b), 3.75 – 3.73 (m, 1H, H-5), 3.73 – 3.69 (m, 1H, ManOC*H*H), 3.44 (dq, *J* = 9.8, 7.0 Hz, 1H, ManOCH*H*), 1.16 (t, *J* = 7.1 Hz, 3H, CH$_3$). **^{13}C NMR (176 MHz, CDCl$_3$)** δ 138.6 (Ph-C), 138.4 (Ph-C), 138.4 (Ph-C), 128.5 (Ph-C), 128.4 (Ph-C), 128.3 (Ph-C), 128.3 (Ph-C), 128.2 (Ph-C), 128.0 (Ph-C), 127.9 (Ph-C), 127.9 (Ph-C), 127.8 (Ph-C), 127.7 (Ph-C), 127.7 (Ph-C), 127.6 (Ph-C), 127.5 (Ph-C), 127.5 (Ph-C), 127.4 (Ph-C), 127.4

(Ph-C), 97.6 (C-1), 80.3 (C-3), 75.1 (C-8), 75.0 (C-4), 74.8 (C-2), 73.3 (C-7), 72.5 (C-10), 72.1 (C-9), 71.7 (C-5), 69.3 (C-6), 62.9 (ManO*C*HH), 14.9 (CH_3). **HRMS (ESI)** calcd for $C_{36}H_{40}O_6$ $[M+Na]^+$: 591.2723, found: 591.2924

^1H NMR (700 MHz, $CDCl_3$) of ***a*-anomer of Man-OEt**:

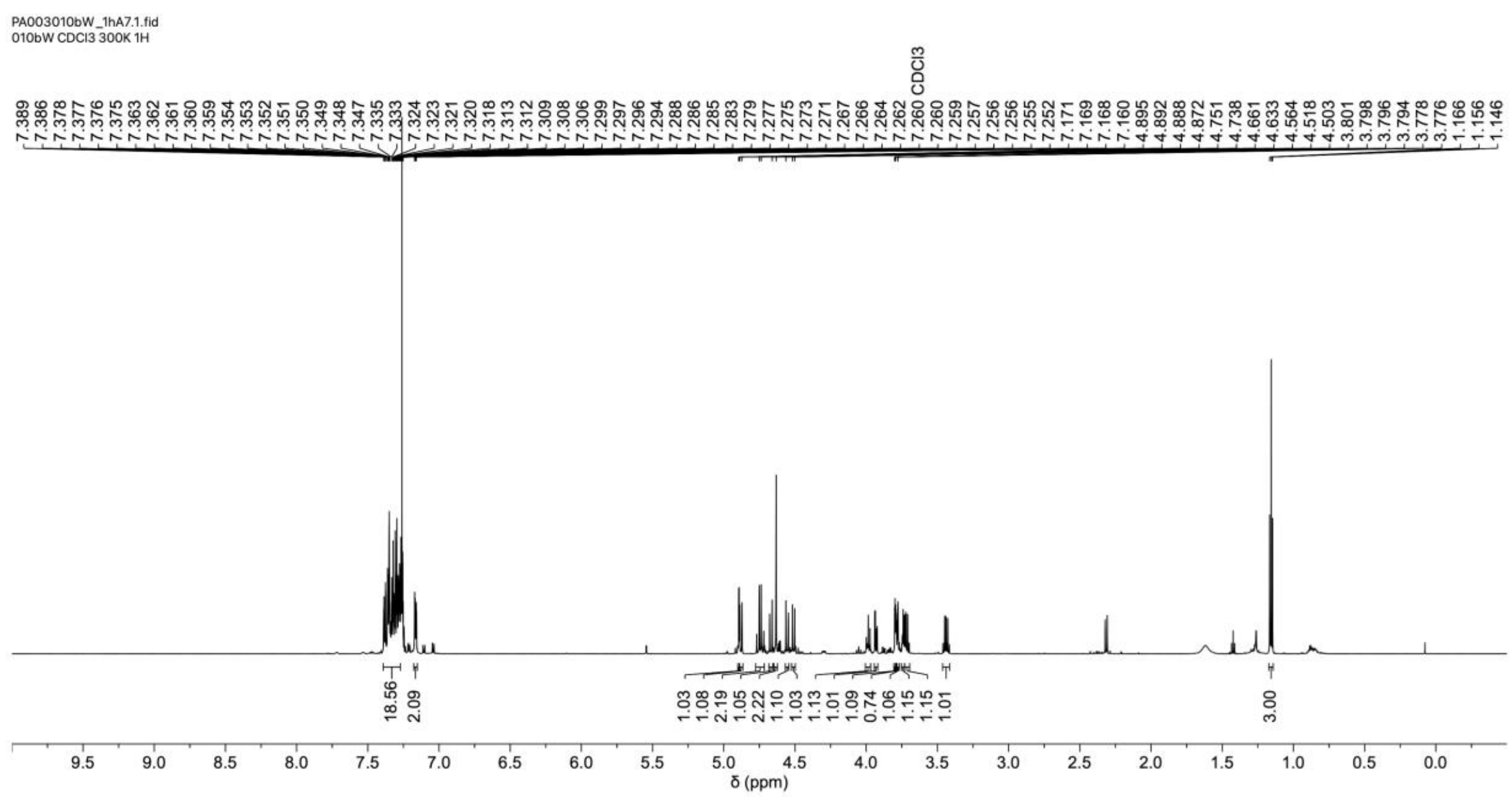

2D-HSQC (^1H, ^{13}C) (176 MHz, $CDCl_3$) of ***a*-anomer of Man-OEt**:

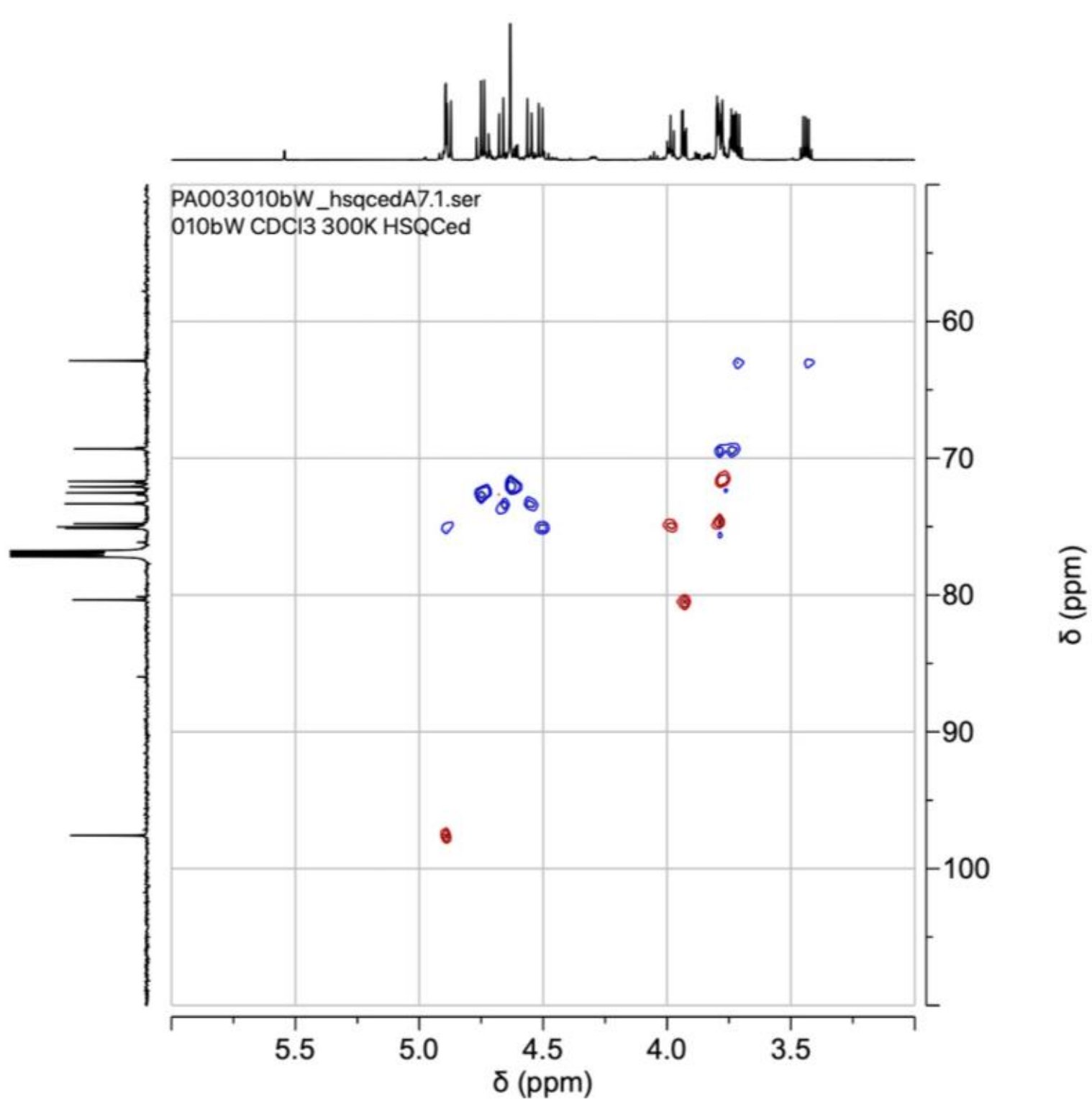

^{13}C NMR (176 MHz, $CDCl_3$) of ***a*-anomer of Man-OEt**:

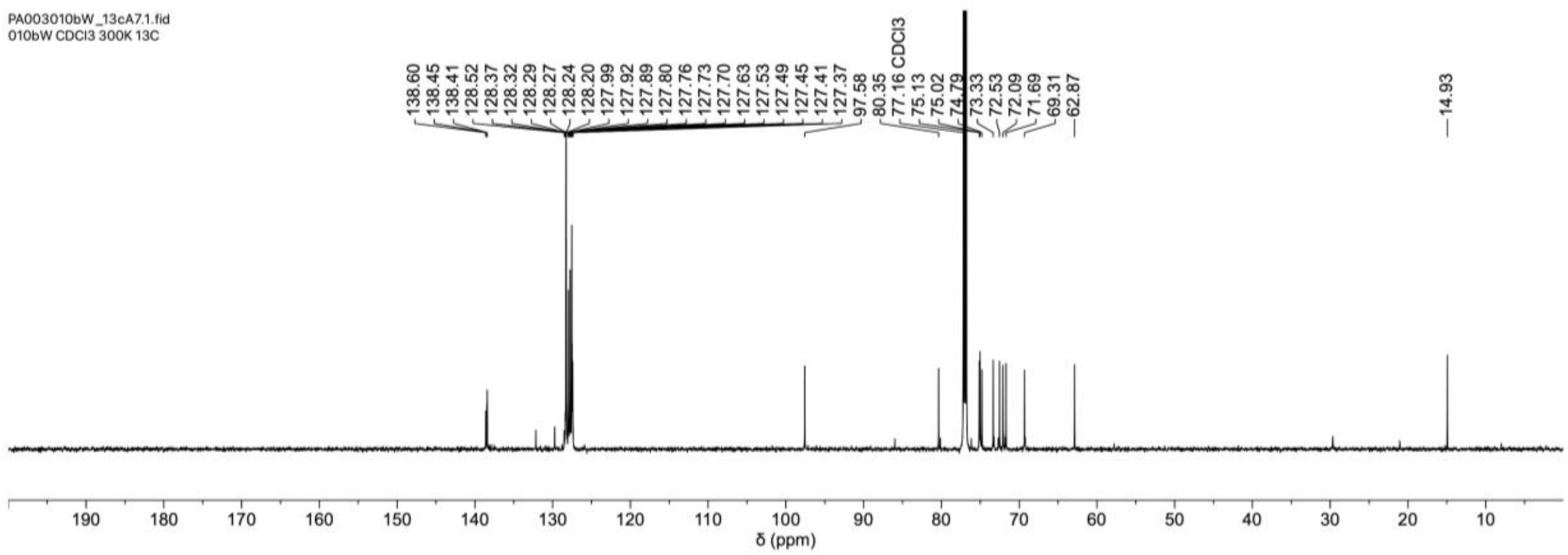

DEPT135 NMR (176 MHz, $CDCl_3$) of ***a*-anomer of Man-OEt**:

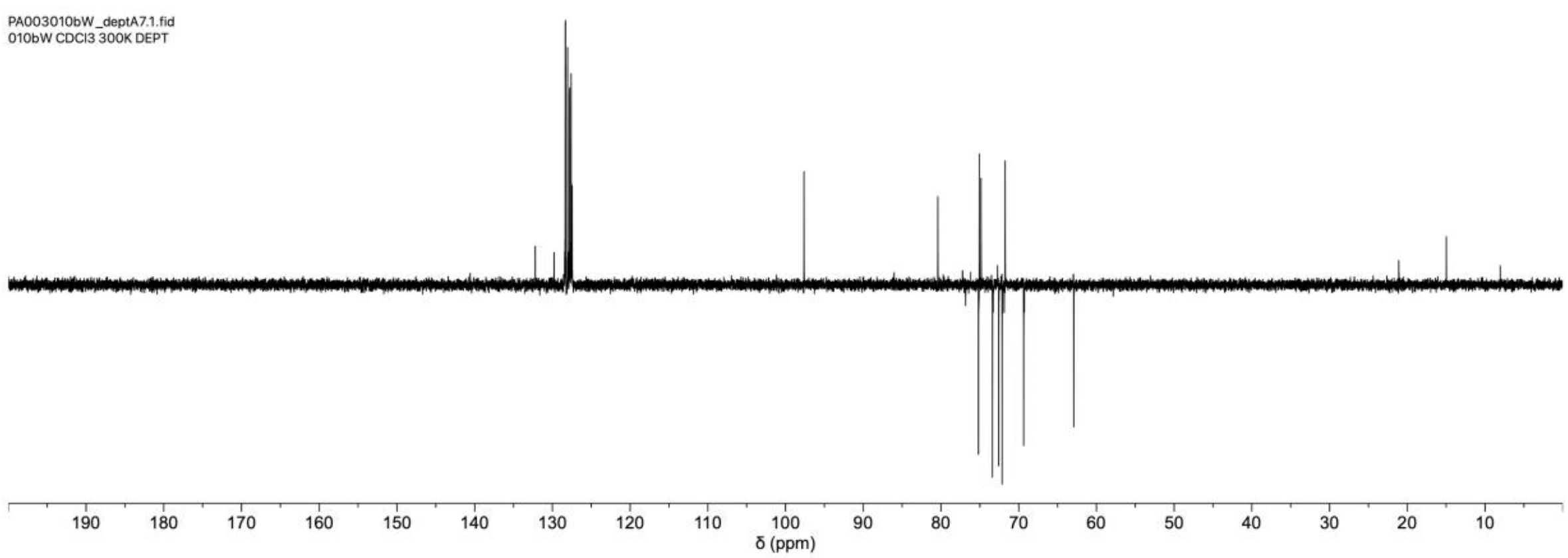

ESI-MS of ***a*-anomer of Man-OEt**:

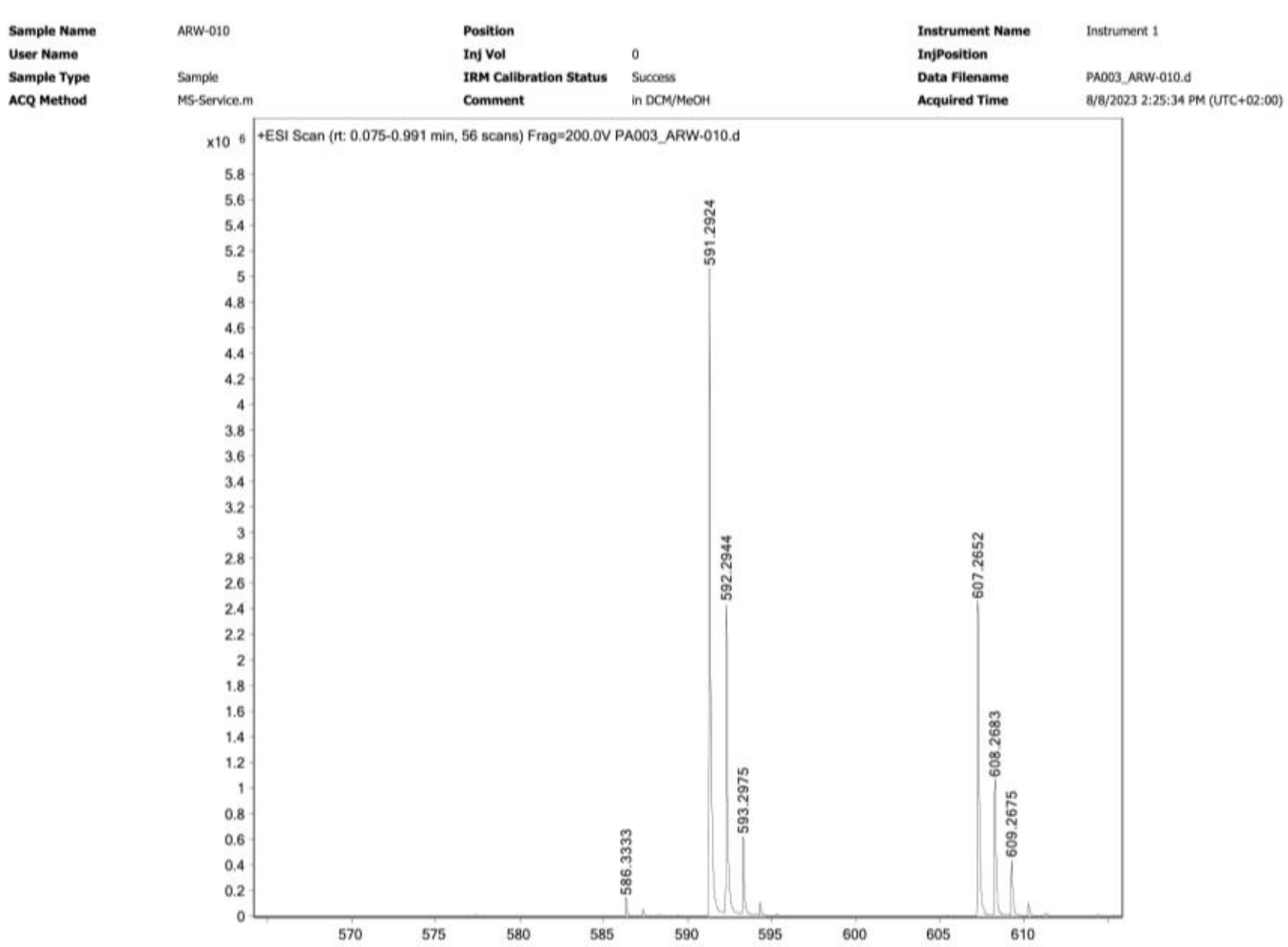

iso-Propyl 2,3,4,6-Tetra-O-benzyl-D-mannopyranoside

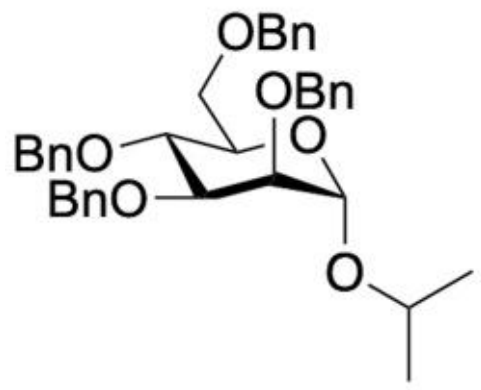

Man-O*i*Pr

***a*-anomer of Man-OPr:**

^{1}H NMR (700 MHz, CDCl$_3$) δ 7.40 – 7.22 (m, 18H, Ph-H), 7.20 – 7.13 (m, 2H, Ph-H), 4.96 (d, *J* = 1.9 Hz, 1H, H-1), 4.87 (d, *J* = 10.6 Hz, 1H, H-8a), 4.78 (d, *J* = 12.4 Hz, 1H, H-10a), 4.71 (d, *J* = 12.5 Hz, 1H, H-10b), 4.67 (d, *J* = 12.0 Hz, 1H, H-7a), 4.64 (s, 2H, H-9), 4.54 (d, *J* = 12.1 Hz, 1H, H-7b), 4.50 (d, *J* = 10.7 Hz, 1H, H-8b), 3.99 (t, *J* = 9.5 Hz, 1H, H-4), 3.92 (dd, *J* = 9.4, 3.1 Hz, 1H, H-3), 3.89 (p, *J* = 6.2 Hz, 1H, ManOC*H*), 3.84 – 3.81 (m, 1H, H-5), 3.79 (dd, *J* = 10.6, 5.0 Hz, 1H, H-6a), 3.74 (dd, *J* = 3.1, 1.9 Hz, 1H, H-2), 3.72 (dd, *J* = 10.6, 1.9 Hz, 1H, H-6b), 1.16 (d, *J* = 6.3 Hz, 3H, CH$_3$'), 1.06 (d, *J* = 6.1 Hz, 3H, CH$_3$''). **^{13}C NMR (176 MHz, CDCl$_3$)** δ 138.6 (Ph-C), 138.4 (Ph-C), 128.3 (Ph-C), 128.3 (Ph-C), 128.2 (Ph-C), 128.0 (Ph-C), 127.8 (Ph-C), 127.7 (Ph-C), 127.5 (Ph-C), 127.5 (Ph-C), 127.5 (Ph-C), 127.4 (Ph-C), 127.4 (Ph-C), 95.8 (C-1), 80.3 (C-3), 75.2 (C-2), 75.1 (C-8), 75.1 (C-4), 73.3 (C-10), 72.6 (C-7), 72.1 (C-9), 71.7 (C-5), 69.3 (C-6), 68.8 (ManO*C*H), 23.19 (CH$_3$'), 21.2 (CH$_3$''). **HRMS (ESI)** calcd for $C_{37}H_{42}O_6$ [M+Na]$^+$: 605.2879, found: 605.3096

^{1}H NMR (700 MHz, CDCl$_3$) of ***a*-anomer of Man-OPr**:

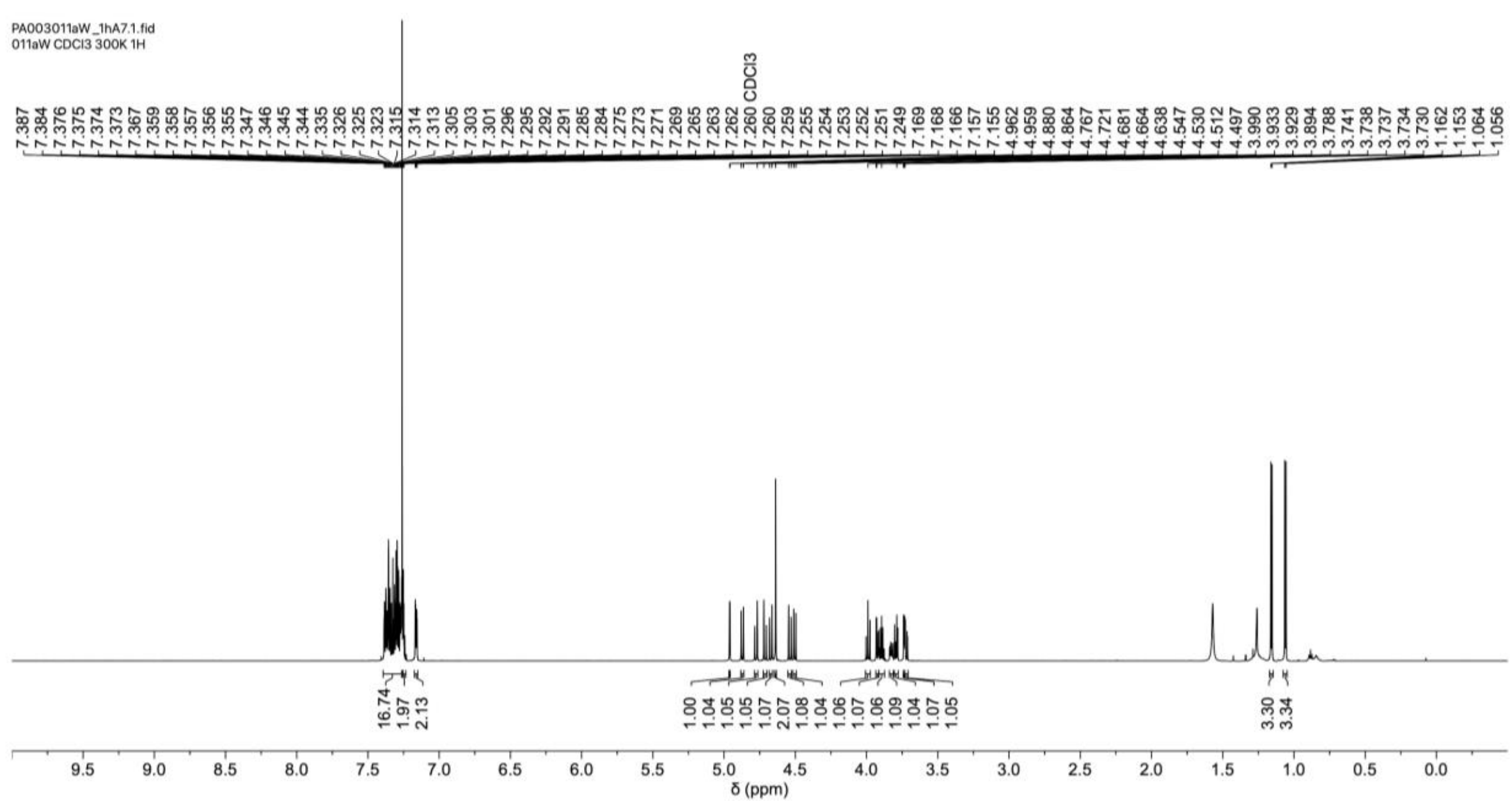

2D-HSQC (^{1}H, ^{13}C) (176 MHz, $CDCl_3$) of ***a*-anomer of Man-OPr**:

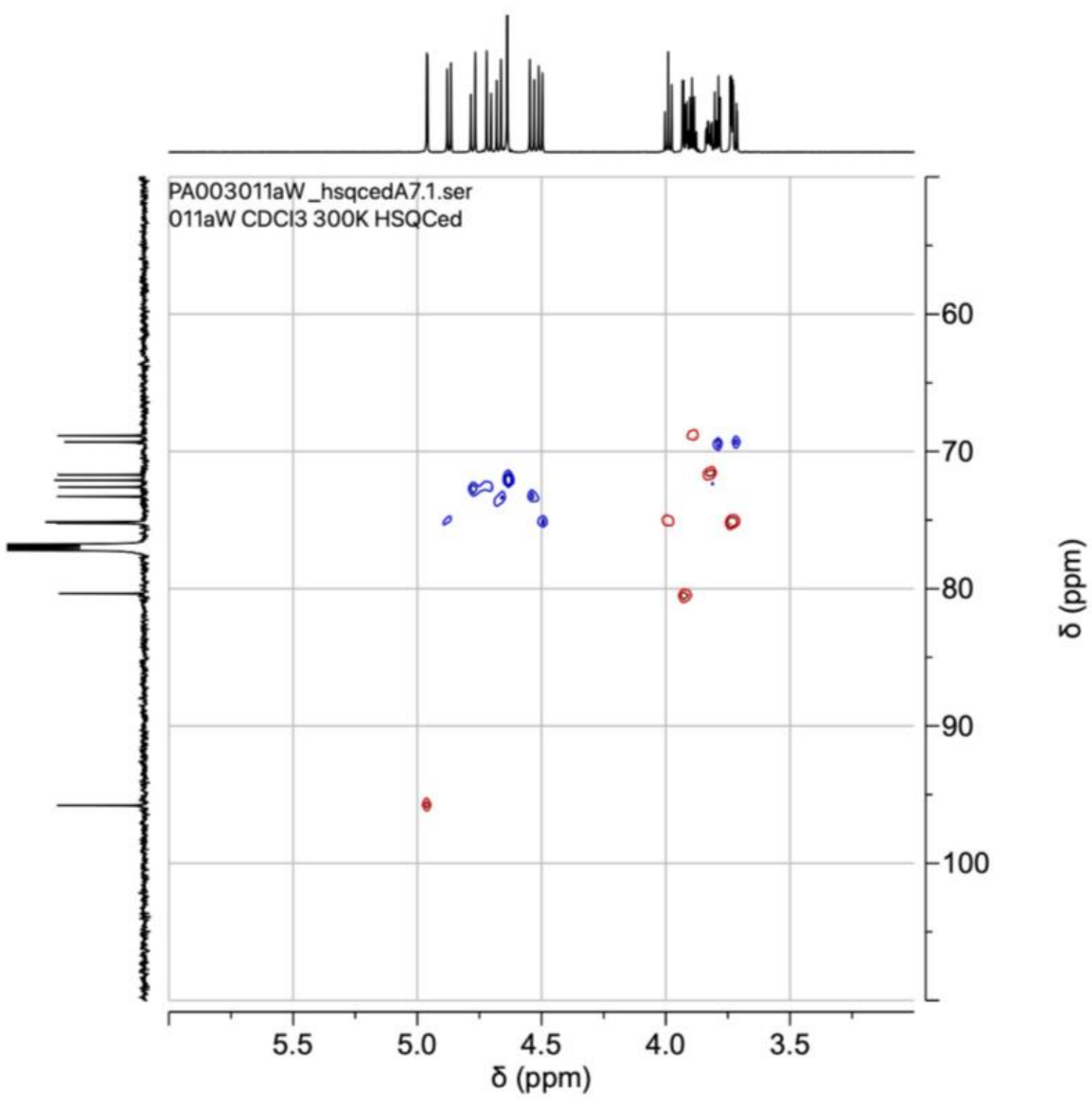

^{13}C NMR (176 MHz, $CDCl_3$) of ***a*-anomer of Man-OPr**:

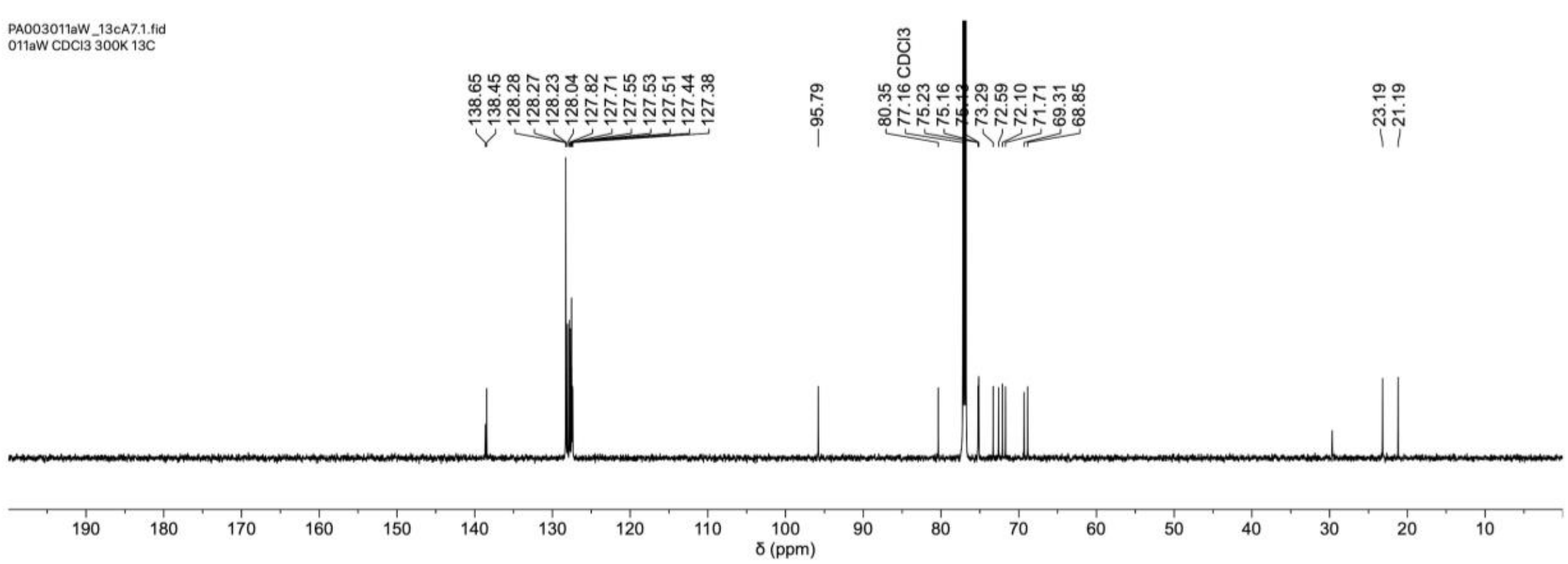

DEPT135 NMR (176 MHz, $CDCl_3$) of ***a*-anomer of Man-OPr**:

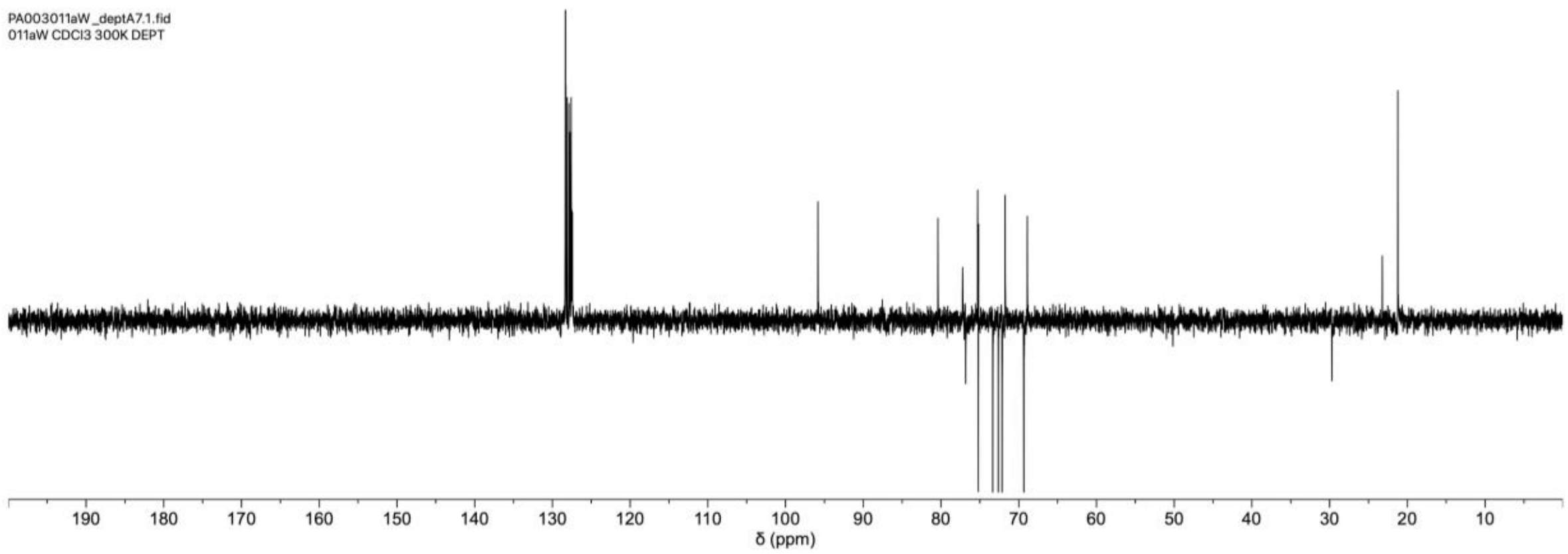

ESI-MS of ***a*-anomer of Man-OPr**:

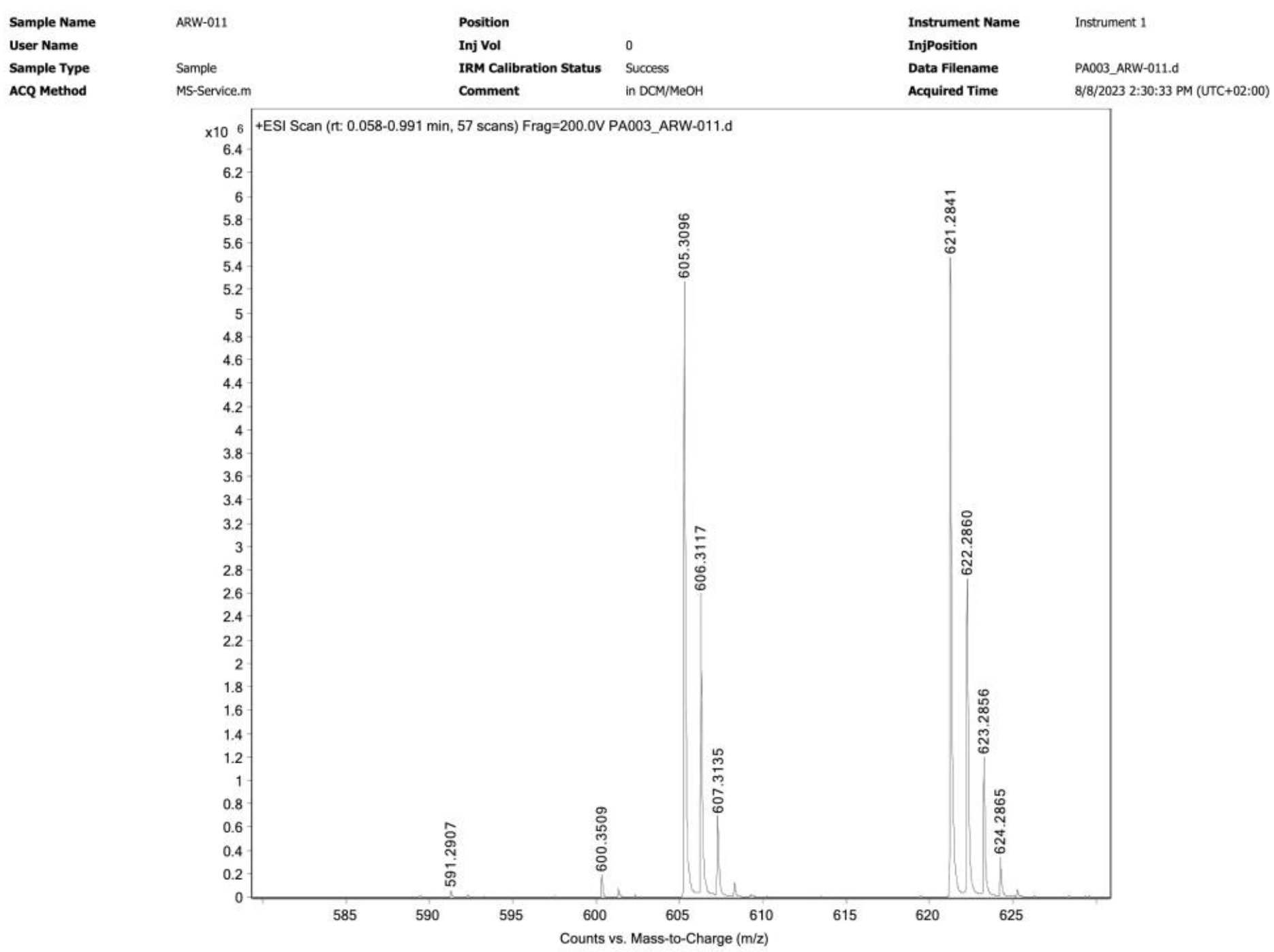

9. **Reference**

(1) Andreana, P. R.; Crich, D. *ACS Cent. Sci.* **2021**, *7*, 1454-1462.

(2) Demchenko, A. V.; Rousson, E.; Boons, G. J. *Tetrahedron Lett.* **1999**, *40*, 6523-6526.

(3) Satoh, H.; Hansen, H. S.; Manabe, S.; van Gunsteren, W. F.; Hünenberger, P. H. *J. Chem. Theory Comput.* **2010**, *6*, 1783-1797.

(4) Chang, C.-W.; Lin, M.-H.; Chiang, T.-Y.; Wu, C.-H.; Lin, T.-C.; Wang, C.-C. *Sci. Adv.* **2023**, *9*, eadk0531.

(5) Chang, C. W.; Lin, M. H.; Wu, C. H.; Chiang, T. Y.; Wang, C. C. *J. Org. Chem.* **2020**, *85*, 15945-15963.

(6) Bohé, L.; Crich, D. *Carbohydr. Res.* **2015**, *403*, 48-59.

(7) Crich, D. *J. Am. Chem. Soc.* **2020**, *143*, 17-34.

(8) Wen, P.; Crich, D. *J. Org. Chem.* **2015**, *80*, 12300-12310.

(9) Santana, A. G.; Montalvillo-Jimenez, L.; Diaz-Casado, L.; Corzana, F.; Merino, P.; Canada, F. J.; Jimenez-Oses, G.; Jimenez-Barbero, J.; Gomez, A. M.; Asensio, J. L. *J. Am. Chem. Soc.* **2020**, *142*, 12501-12514.

(10) de Kleijne, F. F. J.; Ter Braak, F.; Piperoudis, D.; Moons, P. H.; Moons, S. J.; Elferink, H.; White, P. B.; Boltje, T. J. *J. Am. Chem. Soc.* **2023**, *145*, 26190-26201.

(11) Chang, C.-W.; Wu, C.-H.; Lin, M.-H.; Liao, P.-H.; Chang, C.-C.; Chuang, H.-H.; Lin, S.-C.; Lam, S.; Verma, V. P.; Hsu, C.-P.; Wang, C.-C. *Angew. Chem. Int. Ed.* **2019**, *58*, 16775-16779.

(12) Kopysov, V.; Makarov, A.; Boyarkin, O. V. *Anal. Chem.* **2015**, *87*, 4607-4611.

(13) Kopysov, V.; Makarov, A.; Boyarkin, O. V. *Anal. Chem.* **2017**, *89*, 544-547.

(14) Kopysov, V.; Gorshkov, M. V.; Boyarkin, O. V. *Analyst* **2018**, *143*, 833-836.

(15) Saparbaev, E.; Kopysov, V.; Aladinskaia, V.; Ferrieres, V.; Legentil, L.; Boyarkin, O. V. *Anal. Chem.* **2020**, *92*, 14624-14632.

(16) Lobas, A. A.; Solovyeva, E. M.; Saparbaev, E.; Gorshkov, M. V.; Boyarkin, O. V. *Talanta* **2021**, *232*, 122412.

(17) Kopysov, V.; Yamaletdinov, R.; Boyarkin, O. V. *Analyst* **2024**, *149*, 4600-4604.

(18) Saparbaev, E.; Yamaletdinov, R.; Boyarkin, O. V. *Anal. Chem.* **2021**, *93*, 12822-12826.

(19) Saparbaev, E.; Zviagin, A.; Boyarkin, O. V. *Anal. Chem.* **2022**, *94*, 9514-9518.

(20) Habibi, S. C.; Nagy, G. *Anal. Chem.* **2023**, *95*, 8028-8035.

(21) Cui, H.; Zheng, D.; Chu, H.; Li, Y.; Li, G. *J. Phys. Chem. B* **2026**, *130*, 1054-1064.

(22) Pellegrinelli, R. P.; Yue, L.; Carrascosa, E.; Warnke, S.; Ben Faleh, A.; Rizzo, T. R. *J. Am. Chem. Soc.* **2020**, *142*, 5948-5951.
(23) Greis, K.; Mucha, E.; Lettow, M.; Thomas, D. A.; Kirschbaum, C.; Moon, S.; Pardo-Vargas, A.; von Helden, G.; Meijer, G.; Gilmore, K.; Seeberger, P. H.; Pagel, K. *ChemPhysChem* **2020**, *21*, 1905-1907.
(24) van der Vorm, S.; Hansen, T.; van Hengst, J. M. A.; Overkleeft, H. S.; van der Marel, G. A.; Codée, J. D. C. *Chem. Soc. Rev.* **2019**, *48*, 4688-4706.
(25) Hettikankanamalage, A. A.; Lassfolk, R.; Ekholm, F. S.; Leino, R.; Crich, D. *Chem. Rev.* **2020**, *120*, 7104-7151.
(26) **2016**.
(27) Pracht, P.; Bohle, F.; Grimme, S. *Phys. Chem. Chem. Phys.* **2020**, *22*, 7169-7192.
(28) Bannwarth, C.; Ehlert, S.; Grimme, S. *J. Chem. Theory Comput.* **2019**, *15*, 1652-1671.
(29) Adamo, C.; Barone, V. *J. Chem. Phys.* **1999**, *110*, 6158-6170.
(30) Grimme, S.; Antony, J.; Ehrlich, S.; Krieg, H. *J. Chem. Phys.* **2010**, *132*, 154104.
(31) Chang, C.-W.; Greis, K.; Prabhu, G. R. D.; Wehner, D.; Kirschbaum, C.; Ober, K.; Torres-Boy, A. Y.; Leichnitz, S.; Meijer, G.; von Helden, G.; Seeberger, P. H.; Pagel, K. *Nat. Synth.* **2024**, *3*, 1377-1384.
(32) Marianski, M.; Mucha, E.; Greis, K.; Moon, S.; Pardo, A.; Kirschbaum, C.; Thomas, D. A.; Meijer, G.; von Helden, G.; Gilmore, K.; Seeberger, P. H.; Pagel, K. *Angew. Chem. Int. Ed.* **2020**, *59*, 6166-6171.
(33) Siyabalapitiya Arachchige, S.; Crich, D. *J. Org. Chem.* **2021**, *87*, 316-339.
(34) Dharuman, S.; Amarasekara, H.; Crich, D. *J. Org. Chem.* **2018**, *83*, 10334-10351.
(35) Zhang, Z.; Ollmann, I. R.; Ye, X.-S.; Wischnat, R.; Baasov, T.; Wong, C.-H. *J. Am. Chem. Soc.* **1999**, *121*, 734-753.
(36) Zhou, S.; Prebyl, B. S.; Cook, K. D. *Anal. Chem.* **2002**, *74*, 4885-4888.
(37) Gadzuk-Shea, M. M.; Hubbard, E. E.; Gozzo, T. A.; Bush, M. F. *J. Am. Soc. Mass Spectrom.* **2023**, *34*, 1675-1684.
(38) Konermann, L.; Liu, Z.; Haidar, Y.; Willans, M. J.; Bainbridge, N. A. *Anal. Chem.* **2023**, *95*, 13957-13966.
(39) Chang, C.-W.; Lin, M.-H.; Wang, C.-C. *Chem. Eur. J.* **2021**, *27*, 2556-2568.

(40) van Hengst, J. M. A.; Hellemons, R. J. C.; Remmerswaal, W. A.; van de Vrande, K. N. A.; Hansen, T.; van der Vorm, S.; Overkleeft, H. S.; van der Marel, G. A.; Codée, J. D. C. *Chem. Sci.* **2023**, *14*, 1532-1542.

(41) Huang, M.; Garrett, G. E.; Birlirakis, N.; Bohé, L.; Pratt, D. A.; Crich, D. *Nat. Chem.* **2012**, *4*, 663-667.

(42) de Kleijne, F. F. J.; Ter Braak, F.; Moons, P. H.; Rutjes, F. P. J. T.; White, P. B.; Boltje, T. J. *Nat. Commun.* **2026**, *17*, 2987.

(43) Chang, C.-W.; Lin, M.-H.; Chan, C.-K.; Su, K.-Y.; Wu, C.-H.; Lo, W.-C.; Lam, S.; Cheng, Y.-T.; Liao, P.-H.; Wong, C.-H.; Wang, C.-C. *Angew. Chem. Int. Ed.* **2021**, *60*, 12413-12423.
